# Oxidative coupling of *sp*^2^ and *sp*^3^ carbon–hydrogen bonds to construct dihydrobenzofurans

**DOI:** 10.1038/s41467-017-00078-6

**Published:** 2017-08-10

**Authors:** Jiang-Ling Shi, Ding Wang, Xi-Sha Zhang, Xiao-Lei Li, Yu-Qin Chen, Yu-Xue Li, Zhang-Jie Shi

**Affiliations:** 1 Beijing National Laboratory of Molecule Sciences and Key Laboratory of Bioorganic Chemistry and Molecular Engineering of Ministry of Education, College of Chemistry and Molecular Engineering, Peking University, Beijing, 100871 China; 2 College of Chemistry and Material Science, Sichuan Normal University, Chengdu, 610068 China; 3 Institute of Chemistry, Chinese Academy of Sciences, Beijing, 100872 China; 4 Department of Chemistry, Fudan University, Shanghai, 200433 China; 5 State Key Laboratory of Organometallic Chemistry, Chinese Academy of Sciences, Shanghai, 200032 China

## Abstract

Metal-catalyzed cross-couplings provide powerful, concise, and accurate methods to construct carbon–carbon bonds from organohalides and organometallic reagents. Recent developments extended cross-couplings to reactions where one of the two partners connects with an aryl or alkyl carbon–hydrogen bond. From an economic and environmental point of view, oxidative couplings between two carbon–hydrogen bonds would be ideal. Oxidative coupling between phenyl and “inert” alkyl carbon–hydrogen bonds still awaits realization. It is very difficult to develop successful strategies for oxidative coupling of two carbon–hydrogen bonds owning different chemical properties. This article provides a solution to this challenge in a convenient preparation of dihydrobenzofurans from substituted phenyl alkyl ethers. For the phenyl carbon–hydrogen bond activation, our choice falls on the carboxylic acid fragment to form the palladacycle as a key intermediate. Through careful manipulation of an additional ligand, the second “inert” alkyl carbon–hydrogen bond activation takes place to facilitate the formation of structurally diversified dihydrobenzofurans.

## Abstract (web summary)

Cross-dehydrogenative coupling is finding increasing application in synthesis, but coupling two chemically distinct sites remains a challenge. Here, the authors report an oxidative coupling between *sp* ^2^ and *sp* ^3^ carbons by sequentially activating the more active aryl site followed by the alkyl position.

## Introduction

Carbon–hydrogen bonds are ubiquitous in organic compounds. With fast developments in the field of C–H functionalization during the past three decades, the understanding of the reactivity of carbon–hydrogen bonds is continuously updated. Many transformations involving carbon–hydrogen bonds as at least one of the partners have been developed to construct carbon–carbon bonds^1^. For example, both aryl and alkyl carbon–hydrogen bonds take part in cross-couplings with aryl/alkyl halides^2, 3^. Vice versa, with or without directing groups, aryl carbon–hydrogen bonds play key roles as surrogates of aryl halides in couplings with various organometallic reagents under oxidative conditions^4, 5^. Arguably, however, the most efficient and ideal method to construct carbon–carbon bonds is to use carbon–hydrogen bonds exclusively as precursors in a single chemical operation.

Stimulated by this idea, the concept of cross-dehydrogenative coupling (CDC) was conceived and applied to construct different types of carbon–carbon bonds^6^. However, in most of these cases, one of the carbon–hydrogen bonds must exhibit extraordinary reactivity (for example, carbon–hydrogen bonds adjacent to heteroatoms, benzyl/allyl carbon–hydrogen bonds, as well as carbon–hydrogen bonds at α-position of a carbonyl group)^7–10^. Cross-couplings between two “inert” carbon–hydrogen bonds face a number of challenges: the requirement of high chemo- and regioselectivity in precursors containing multiple carbon–hydrogen bonds, the need to find conditions to activate two carbon–hydrogen bonds of different reactivities in a one-pot process, and the requirement to control cross-coupling between two partners over that of homocoupling.

Oxidative couplings between two aryl carbon–hydrogen bonds have been well developed in both intra- and intermolecular manner over the past years^11–13^. The next goal is to develop efficient oxidative coupling protocols to construct carbon–carbon bonds between both “inert” aromatic and aliphatic carbon–hydrogen bonds. Indeed, this would be the most straightforward method for the alkylation of aromatic compounds.

**Fig. 1:**
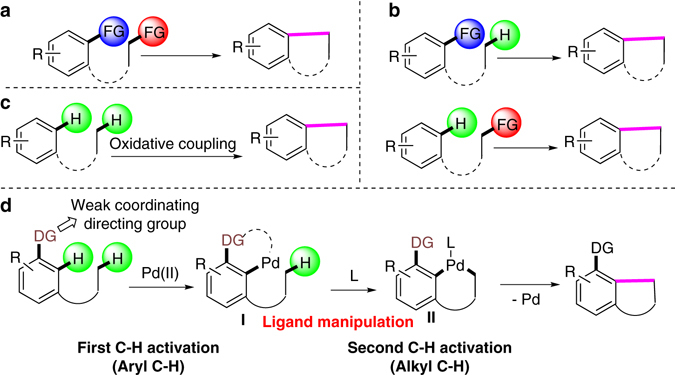
Efficient method to construct benzo-fused rings by cross-coupling. **a** Conventional method of cross-coupling. **b** Recent coupling based on carbon–hydrogen activation. **c** Ideal approach through oxidative coupling. **d** Design on oxidative coupling by ligand-manipulated tandem C–H activations

We hypothesized that benzo-fused ring systems may be accessible from substituted benzene with alkyl groups tethered with different linkers. Indeed, benzo-fused scaffolds playing important functions in life science and material chemistry, which can be constructed through intramolecular cross-couplings or reductive couplings from two functionalized motifs (Fig. 1a)^14, 15^. Recent advances in carbon–hydrogen activation provided efficient and competitive pathways to approach benzo-fused rings from monofunctionalized starting materials (Fig. 1b)^2, 16^. Obviously, the ideal intramolecular oxidative coupling from simple precursors would do away with a prefunctionalization as shown in Fig. 1c. Unfortunately, current chemistry, for example, via the direct carbon–hydrogen bond activation based on radical chemistry and directing strategy, has not been successful to reach this goal^17, 18^. To date, only two excellent examples have been reported for the intramolecular cross-coupling between *sp* ^2^ and *sp* ^3^ carbon–hydrogen bonds to construct fused rings. Both were initiated from electron-rich heterocylces^19, 20^. Examples with benzene derivatives are not literature-known. We aimed to meet this challenge through a demonstration of the synthesis of versatile dihydrobenzofuran derivatives from readily available and simple phenyl alkyl ethers.

As the directing strategy has proven efficient for single carbon–hydrogen bond activation^21, 22^ and the intramolecular aliphatic carbon–hydrogen bond activations have been shown effective from in-situ-generated Pd(II) complexes from an oxidative addition of aryl halides with Pd(0) catalysts^23^, we conceived that a new strategy for reaching our target may be through ligand-manipulated tandem carbon–hydrogen activations (Fig. 1d). Based on this design, a versatile directing group would facilitate the first aryl carbon–hydrogen activation to form a palladacycle. It is then essential that an external ligand enters to assist the generation of the key intermediate **II** through the second aliphatic carbon–hydrogen activation.

The carboxylate group has been shown to be a weak yet efficient coordinating group in numerous directed carbon–hydrogen transformations^24–26^. Upon ligand manipulation, the intermediate **I** provides Pd complex **II**, which may undergo strain release of the palladacycle and Pd insertion into the aliphatic carbon–hydrogen bond. Inspired by recent successes in the use of ligands to promote efficiency^27^, accelerate the rate^28^, and even control stereoselectivity in carbon–hydrogen activations^29, 30^, we envisaged that the judicious choice of a ligand may give access to the targeted catalytic oxidative coupling between phenyl- and “inert” alkyl carbon–hydrogen bonds.

## Results

### Oxidative coupling of *sp*^2^ and *sp*^3^ C–H bonds

Based on this design, ether **1a** was readily synthesized from the commercial 3-hydroxy-6-methylbenzoic acid and isobutene. The aryl C(6) position, prone to be a competitive site for palladation, was blocked by a methyl group. Pd(OAc)_2_ was selected as the catalyst, which plays crucial roles in C–H activations. We evaluated various types of ligands to promote oxidative coupling (Supplementary Fig. 65)^27–29^. Finally, to our delight, we found that the addition of 1,4- benzoquinone and acridine led to success. To efficiently isolate the desired product, esterification with MeI was carried out and this provided ester **2a** with 85% yield.

**Fig. 2:**
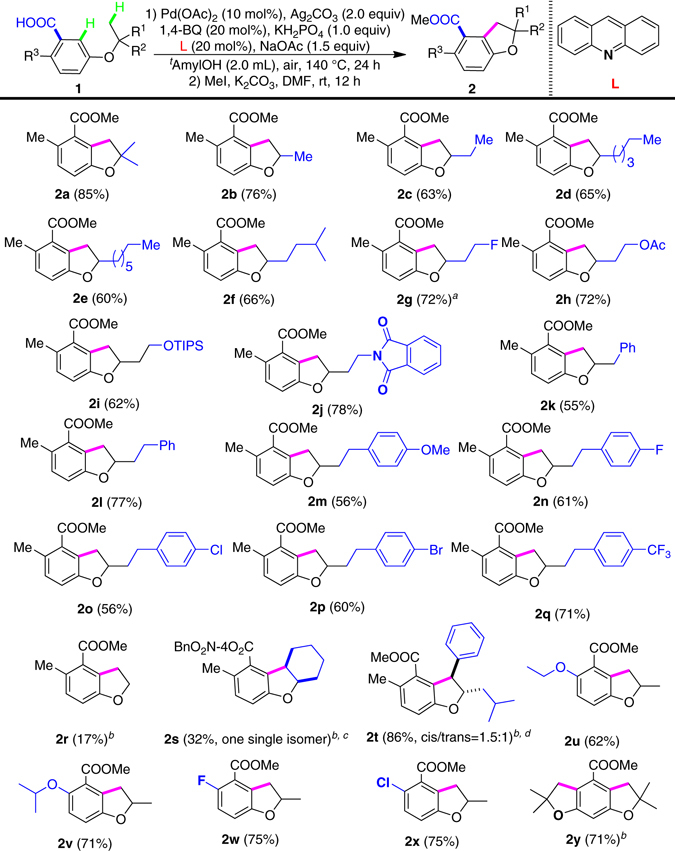
Scope of phenyl alkyl ether substrates. Unless otherwise noted, the reaction conditions were as follows: **1** (0.3 mmol), Pd(OAc)_2_ (0.03 mmol), 1,4-BQ (0.06 mmol), Ag_2_CO_3_ (0.6 mmol), Acridine (0.06 mmol), KH_2_PO_4_ (0.3 mmol), NaOAc (0.45 mmol), and ^*t*^AmylOH (2.0 mL), 140 °C, 24 h. Then the solvent was removed, and MeI (1.5 mmol), K_2_CO_3_ (0.6 mmol), and DMF (3.0 mL) were added at 50 °C for 12 h. ^a^15 mol% Pd(OAc)_2_; ^b^20 mol% Pd(OAc)_2_; ^c^4-Nitrobenzyl bromide (1.5 mmol) instead of MeI; ^d^Selectivity was determined by crude ^1^H NMR spectroscopy

The *tert-*butyl ether function is structurally special and its derivatives are relatively difficult to synthesize. In addition, it can only deliver 2,2-dimethyl dihydydrobenzofuran derivatives in the present application, thereby highly limiting potential applications. We therefore sought to further expand this chemistry to secondary alkyl ethers, which can be readily produced from the secondary alcohols in a single step through either Buchwald–Hartwig etherification^31^ or SN_2_ substitution (for example, Mitsunobu reaction)^32^. We were pleased to find that the isopropyl ether **1b** reacted smoothly to give **2b** with 76% yield (Fig. 2). With this success, we tested a number of different secondary ethers with various chain lengths and found all of them to be successful substrates. The longer chains only slightly decreased yields (**2c**-**2f**). It is important to note that the second carbon–hydrogen activation only took place at the primary position, leaving the secondary carbon–hydrogen bond untouched. The oxidative coupling showed excellent compatibility of functionalities. Thus, C-F (**2g**), acetate (-OAc, **2h**) and silyl ether (-OTIPS, **2i**), and phthalidyl-protected amine (**2j**) functions are compatible. This bodes well for further product manipulation. Although in many transformations benzylic carbon–hydrogen bonds show better reactivity than Me, in our case, Me won over CH_2_Ph. Interestingly, oxidative couplings between two aryl carbon–hydrogen bonds were not observed under current conditions, perhaps indicating the second carbon–hydrogen activation to be mostly controlled by steric hindrance (**2k**).

Unsubstituted and substituted phenyl groups could be attached to the side chain with a two-carbon linker. All of these substrates showed good reactivity to give the desired products (**2l**-**2q**), with unsubstituted phenyl group giving the best yield (**2l**, 77%). Increasing the catalyst loading, in most cases, gave better yield. Gratifyingly, transformable functionalities, including -OMe (**2m**), -F (**2n**), -Cl (**2o**), and -Br (**2p**), are suitable, providing further opportunities to make diversified libraries of dihydrobenzofurans. To confirm the formation of the dihydrobenzofuran scaffolds, the structure of **2ma** (the hydrolysis product of **2m**) was unambiguously confirmed by X-ray structure of its single crystal (Supplementary Fig. 66). Under the standard conditions, ethyl ether (**2r**) was also workable albeit in a low yield.

It is important to note that secondary carbon–hydrogen bonds could also react in the absence of primary carbon–hydrogen bonds. When cyclohexyl ether was subjected, the desired product **2s** was isolated with an excellent distereoselectivity albeit in a relatively low yield. If two different methylene carbon–hydrogen bonds are present, the benzylic one showed dominant reactivity over the aliphatic secondary one (**2t**). However, in this case, both diastereoisomers were obtained in a nearly 1.5:1 ratio. These results provide another possibility to expand this chemistry to a wider substrate scope to form 2,3-disubstiuted dihydrobenzofuran derivatives.

We also tested the motive of different benzoic acids in ether. The *ortho*-aryl substituents were varied first. Other than methyl group, alkoxyl groups also showed good reactivity while the *ortho*- isopropoxyl group gave a much better yield (**2v**) than the *ortho*-ethoxyl group (**2u**). It is interesting to note that both *ortho* *-*F- and Cl- substituents led to successful reactions (**2w** and **2x**). Not only do these results extend the substrate scope but also offer the possibility to functionalize the products. We additionally tested 3,5-disubstituted benzoic acids and observed oxidative coupling to proceed smoothly. When 3,5-di-*tert*-butoxybenzoic acid were submitted, dual oxidative couplings took place to afford tricyclic scaffolds in an excellent yield (**2y**).

### Transformations of the dihydrobenzofuran scaffolds

**Fig. 3:**
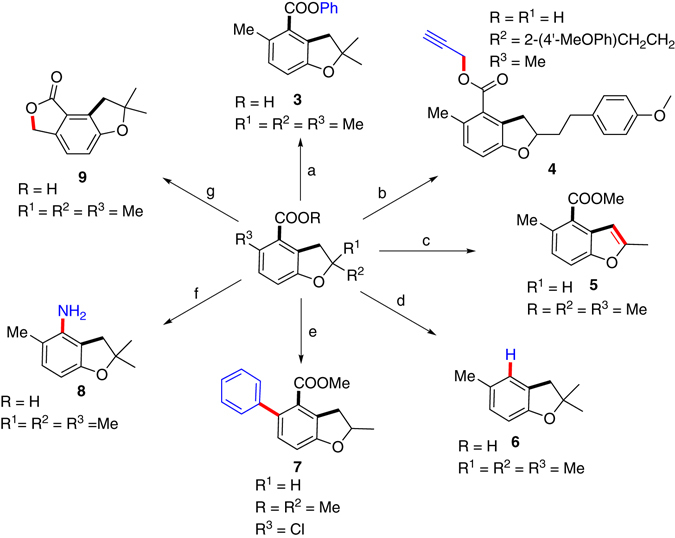
Diversified transformations to produce different substituted benzofurans and dihydrobenzofurans. **a 2aa** (the hydrolysis product of **2a**, 0.2 mmol), PhB(OH)_2_ (0.4 mmol), Cu(OTf)_2_ (0.04 mmol), Ag_2_CO_3_ (2.0 equiv), DMSO (1.0 mL), 120 °C, 2 h, air, **3** was obtained as 67% yield. **b 2ma** (the hydrolysis product of **2m**, 0.3 mmol), 3-Bromopropyne (1.5 mmol), K_2_CO_3_ (0.6 mmol), DMF (3.0 mL), 12 h, air, **4** was obtained as 98% yield. **c 2b** (0.3 mmol), DDQ (0.36 mmol), Toluene (3.0 mL), reflux, N_2_, 48 h, **5** was obtained as 74% yield. **d 2aa** (0.3 mmol), Cu_2_O (0.3 mmol), 1,10-Phen (0.6 mmol), NMP, 160 °C, **6** was obtained as 73% yield. **e 2x** (0.3 mmol), PhB(OH)_2_ (0.45 mmol), Pd(OAc)_2_ (0.2 mol%), dicyclohexyl(2’,6’-dimethoxy-[1,1’-biphenyl]-2-yl)phosphane (0.5 mol%), K_3_PO_4_ (0.6 mmol), Toluene (2.0 mL), 100 °C, 12 h, N_2_, **7** was obtained as 86% yield. **f** (1) **2aa** (0.3 mmol), DPPA (0.315 mmol), Et_3_N (0.9 mmol), THF (2.0 mL), 25 °C, 3 h; (2) H_2_O, reflux, overnight, **8** was obtained as 81% yield. **g 2aa** (0.2 mmol), Pd(OAc)_2_ (5 mol%), Ac-Leu-OH (30 mol%), Ag_2_CO_3_ (0.6 mmol), K_2_HPO_4_ (0.5 mmol), **9** was obtained as 52% yield

In order to demonstrate the potential of applications of this method, we explored the transformations of the carboxylic acid/ester function in dihydrobenzofuran scaffolds (Fig. 3). Obviously, different esters (**3** and **4**) could be obtained using the phenylboronic acid or propargyl bromide. As benzofuran is an important scaffold with multiple bioreactivities, aromatization of **2b** was conducted and benzofuran **5** was obtained in a good yield. This offers an alternative to syntheses of benzofuran derivatives^33^. Next, decarboxylation of the product took place smoothly^34^. Cross-coupling of the *ortho*-chloro substituent to give phenylated dihydrobenzofuran proceeded in excellent yield^35^. The carboxylic group could be transformed into an NH_2_ group (**8)** through Curtis rearrangement^36^. Last but not the least, with methyl substituents, further benzylic C–H activation and lactonization took place to produce a tricyclic compound (**9**) in a moderate yield^37^.

**Fig 4:**
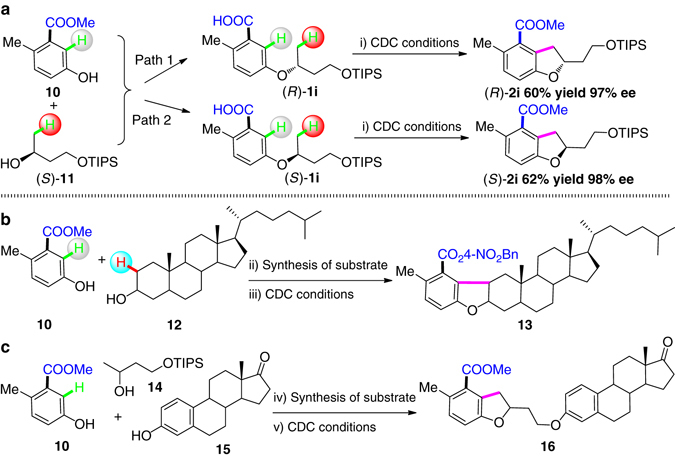
Applications of the oxidative coupling reaction protocol. **a** Path 1. Synthesis of (*R*)-**1i**: **10**, (S)-**11**, PPh_3_, Et_3_N, DIAD, THF, N_2_, 25 °C, 16 h, 85% isolated yield; LiOH·H2O, THF/H2O, 80 °C, 12 h, 93% isolated yield. i) CDC conditions: Pd(OAc)_2_, 1,4-BQ, Ag_2_CO_3_, Acridine, KH_2_PO_4_, NaOAc, ^*t*^AmylOH, air, 140 °C, 24 h; MeI, K_2_CO_3_, DMF, 50 °C, 12 h. 60% isolated yield over two steps, 97% ee. Path 2. Synthesis of (*S*)-**1i**: Benzoic Acid, (*S*)-**11**, PPh_3_, Et_3_N, DIAD, THF, N_2_, 25 °C, 12 h, 90% isolated yield; NaOH, MeOH, reflux, 12 h, afford (*R*)-**11**, 95% isolated yield; 10, (*R*)-**11**, PPh_3_, Et_3_N, DIAD, THF, N_2_, 25 °C, 16 h, 85% isolated yield; LiOH·H2O, THF/H2O, 80 °C, 12 h, 95% isolated yield. i) CDC conditions: Pd(OAc)_2_, 1,4-BQ, Ag_2_CO_3_, Acridine, KH_2_PO_4_, NaOAc, ^*t*^AmylOH, air, 140 °C, 24 h; MeI, K_2_CO_3_, DMF, 50 °C, 12 h. 62% isolated yield over two steps, 98% ee. **b** ii) **10**, **12**, PPh_3_, Et_3_N, DIAD, THF, N_2_, 25 °C, 16 h, 60% isolated yield; LiOH·H2O, THF/H2O, 80 °C, 12 h, 73% isolated yield. iii) CDC conditions: Pd(OAc)_2_, 1,4-BQ, Ag_2_CO_3_, Acridine, KH_2_PO_4_, NaOAc, ^*t*^AmylOH, air, 140 °C, 24 h; 4-Nitrobenzyl bromide, K_2_CO_3_, DMF, 50 °C, 12 h. 44% NMR yield over two steps. **c** iv) **10**, **14**, PPh_3_, DIAD, THF, 25 °C, N_2_, 16 h, 74% isolated yield; TBAF, THF, rt, 80% isolated yield; **15**, PPh_3_, DIAD, THF, 25 °C, N_2_, 16 h, 70% isolated yield; LiOH·H_2_O, THF/H_2_O, 80 °C, 12 h, 86% isolated yield. v) CDC conditions: Pd(OAc)_2_, 1,4-BQ, Ag_2_CO_3_, Acridine, KH_2_PO_4_, NaOAc, ^*t*^AmylOH, air, 140 °C, 24 h. c. MeI, K_2_CO_3_, DMF, 50 °C, 12 h. 52% NMR yield over two steps

Enantiopure dihydrobenzofurans are important as core structures of natural products and drug candidates. Efficient methods to approach single enantiomers of products are very important. Enantiopure secondary alcohols are easily produced, broadly commercially available, and methods for enantiomer inversion are well documented. With this background, we expanded the coupling protocol to the synthesis of a pair of stereoisomers of dihydrobenzofuran (*R*)/(*S*)-**2i** from the same enantiomerically pure secondary alcohol (*S*)-**11**. Through double Mitsunobu reactions, the configuration of stereogenic center was retained and (*S*)-**2i** was obtained in a good yield. By one step Mitsunobu reaction, the stereogenic center was inverted and the other stereoisomer ((*R*)-**2i**) was obtained. Both products were produced with high ee (>97%) as shown by chiral HPLC after desilylation. Therefore, this chemistry provides an economical protocol to produce optically pure compounds containing the dihydrobenzofuran structural unit (Fig. 4a).

As alcohols are widely present in natural products, through the simple etheration/oxidative coupling, a wide range of functionalized compounds can be built up with high potential for drug discovery. With this in mind, we conducted the etheration of sterol **12** and the requisite phenol **10** to produce the ether. Oxidative coupling and esterification produced product **13** in 44% NMR yield. By combining three components, **10**, **14**, and estrone **15**, compound **16** was constructed in three simple steps (Fig. 4b). This method efficiently builds up the complexity from natural and existing molecules for material chemistry and druggable scaffolds.

In summary, a new strategy was developed to carry out the intramolecular oxidative coupling between phenyl- and “inert” aliphatic carbon–hydrogen bonds with a broad functional group compatibility. The weakly coordinated carboxylate was found to be an effective directing group, and the proper ligand was essential for the success of this cross-coupling protocol yielding dihydrobenzofuran products from easily synthesized phenyl alkyl ethers. The carboxylic acid group can be transformed into a wide range of diverse functionalities, thus expanding the range of application of this method. Starting from the same commercially available chiral alcohols and readily synthesized phenols, both stereoisomers of corresponding dihydrobenzofurans were produced, thereby providing an economic route to make valuable complex molecules. With the developed strategy, the oxidative coupling between different carbon–hydrogen bonds shall have a great future to construct different carbon–carbon bonds in organic synthesis.

## Methods

### General procedure for oxidative coupling

To a 20 mL oven-dried glass tube, **1** (0.3 mmol), Pd(OAc)_2_ (10 mol%), 1,4-BQ (20 mol%), Ag_2_CO_3_ (2.0 equiv), Acridine (20 mol%), KH_2_PO_4_ (1.0 equiv), NaOAc (1.5 equiv), and ^*t*^AmylOH (2.0 mL) were added. The tube was sealed and the reaction mixture was stirred at 140 °C for 24 h under an air atmosphere. The mixture was cooled to rt. After removal of the solvent, MeI (5.0 equiv), K_2_CO_3_ (2.0 equiv), and DMF (3.0 mL) were added into the Schlenk tube. The mixture was stirred at 50 °C for 12 h. Then the suspension was filtered through a celite pad and washed with EtOAc (3 × 10 mL). The solvent was then removed in vacuo, and the residue was purified by flash chromatography on silica gel (PE/EtOAc = 100:1 to PE/EtOAc = 30:1) to afford the desired product **2**.

For more specific procedures of the reaction treatment and compounds’ characterization method information, please refer to the Supplementary Methods. For NMR spectra of the compounds in this article, see Supplementary Figures 1–64.

### Data availability

Accession codes: The X-ray crystallographic structures for **2ma** reported in this article have been deposited at the Cambridge Crystallographic Data Centre (CCDC), under deposition number CCDC 1489220. These data can be obtained free of charge from the Cambridge Crystallographic Data Centre via http://www.ccdc.cam.ac.uk/. The authors declare that all other data supporting the findings of this study are available within the article and its Supplementary Information files.

## Electronic supplementary material

Supplementary Information

Peer Review File
