# Supplementary material for: Oxidative coupling of sp2 and sp3 carbon–hydrogen bonds to construct dihydrobenzofurans
Source: Nat Commun. 2017 Aug 10;8:238. doi: 10.1038/s41467-017-00078-6 (PMC5552783; doi:10.1038/s41467-017-00078-6)
Supplement: Supplementary file 1 — Supplementary Information [file 41467_2017_78_MOESM1_ESM.pdf]

SI GUIDE

File Name: Supplementary Information
Description: Supplementary Figures, Supplementary Tables, Supplementary Methods and Supplementary References.

File Name: Peer Review File
Description:

Supplementary Figures

1. NMR spectra of Starting Material

Supplementary Figure 1. ^{1}H, ^{13}C-NMR spectra of product **1a**

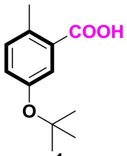

1a

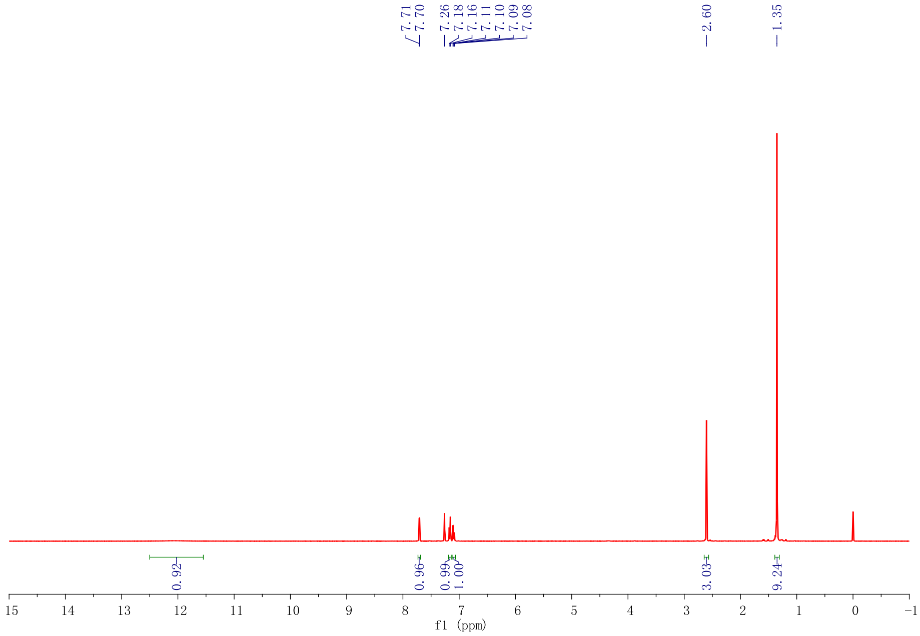

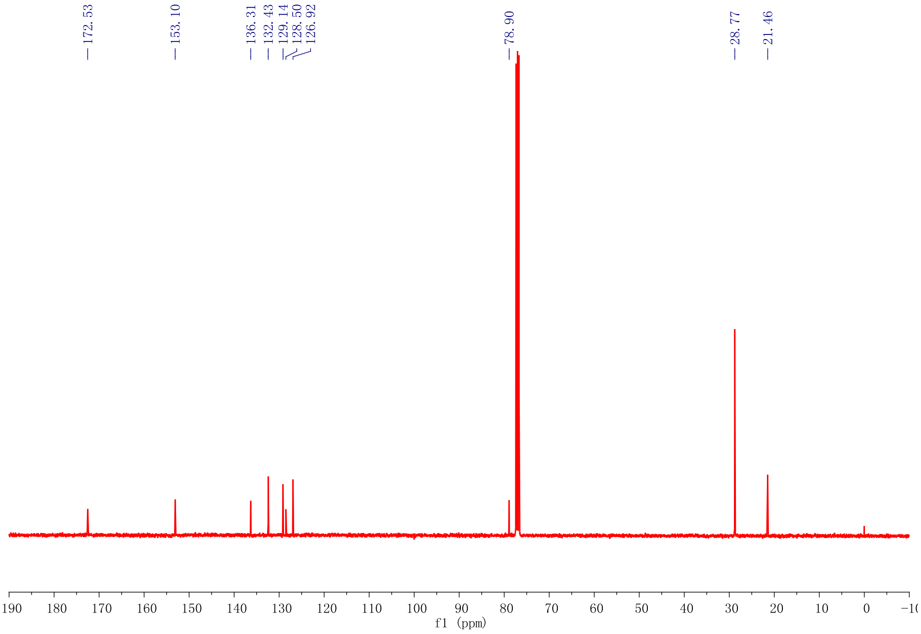

Supplementary Figure 2. ^{1}H, ^{13}C-NMR spectra of product **1b**

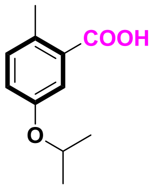

1b

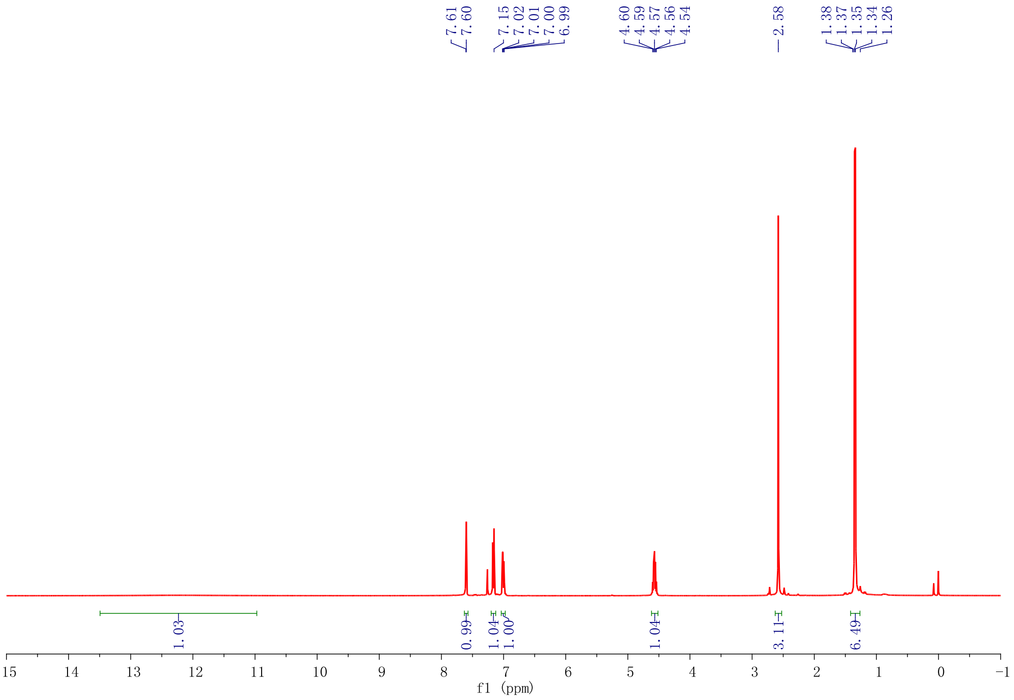

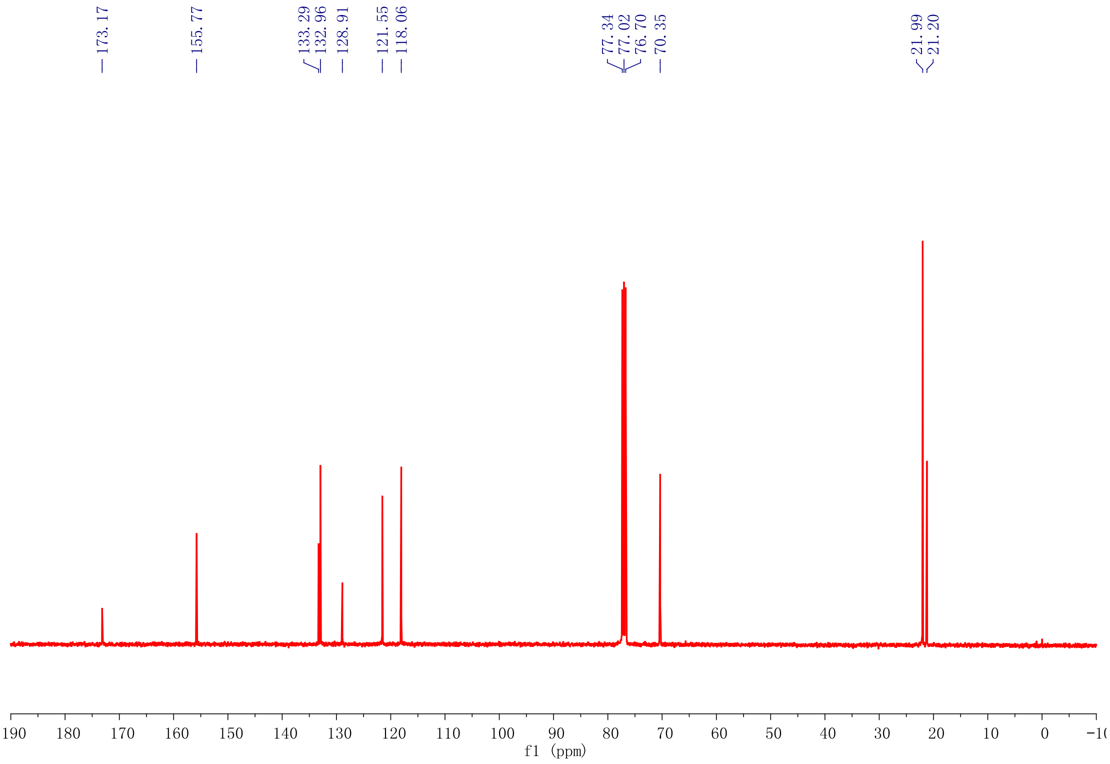

Supplementary Figure 3. ^{1}H, ^{13}C-NMR spectra of product **1c**

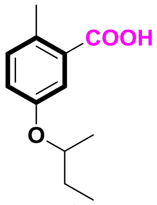

1c

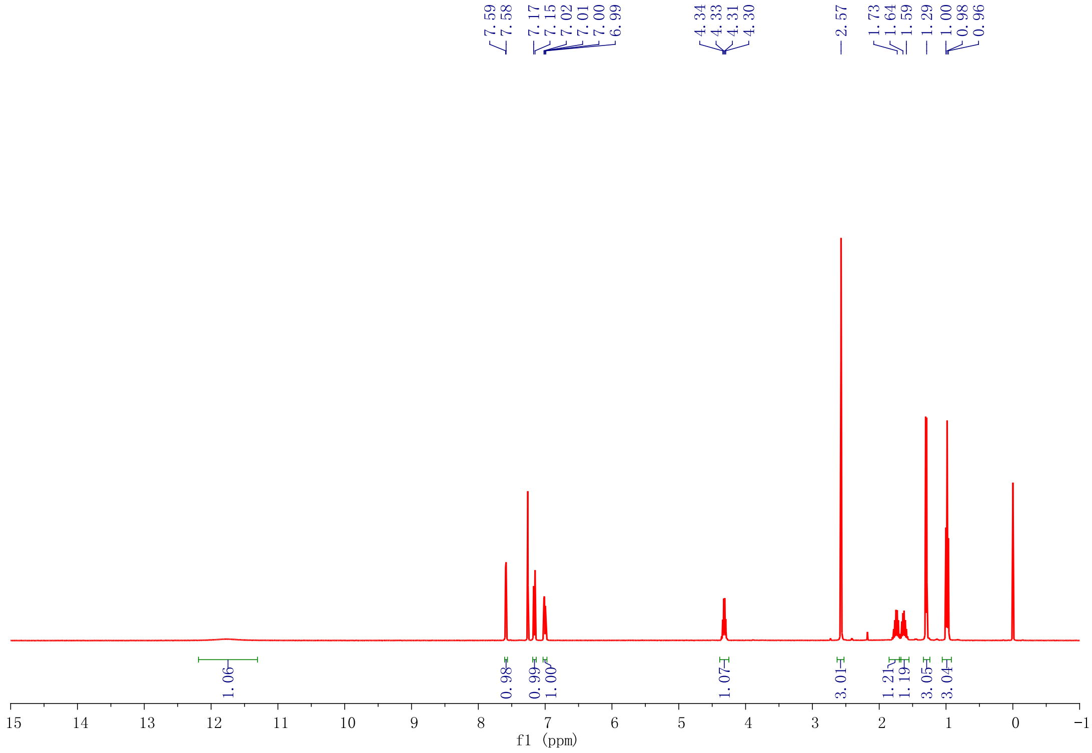

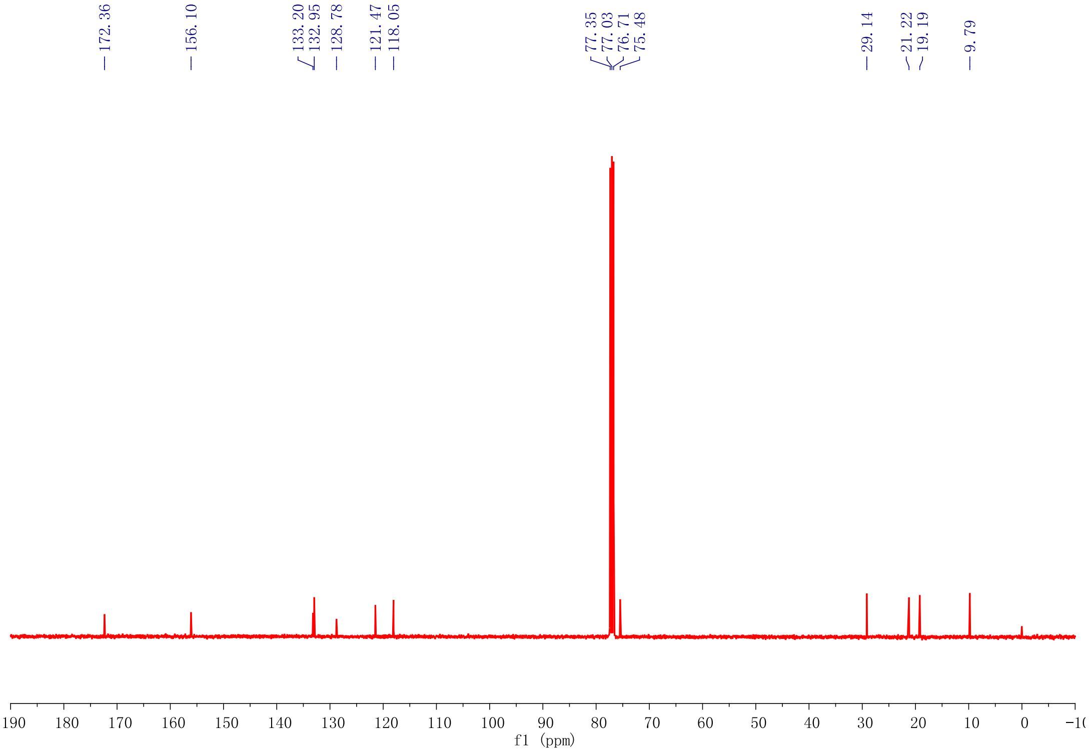

Supplementary Figure 4. ^{1}H, ^{13}C-NMR spectra of product **1d**

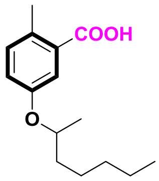

1d

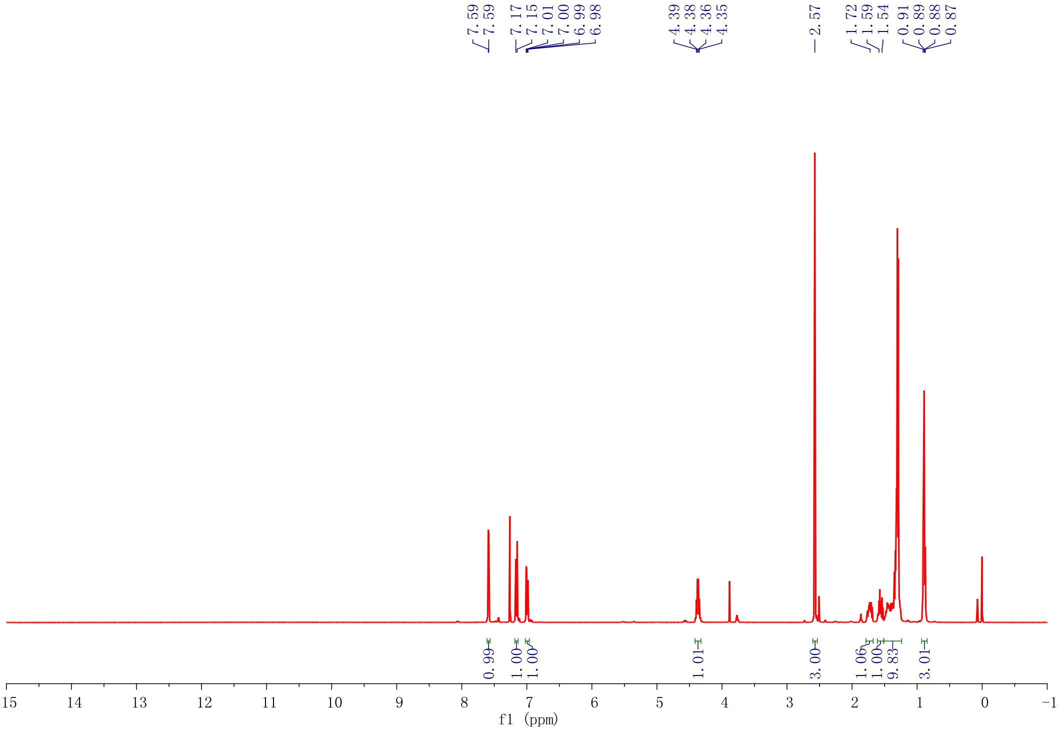

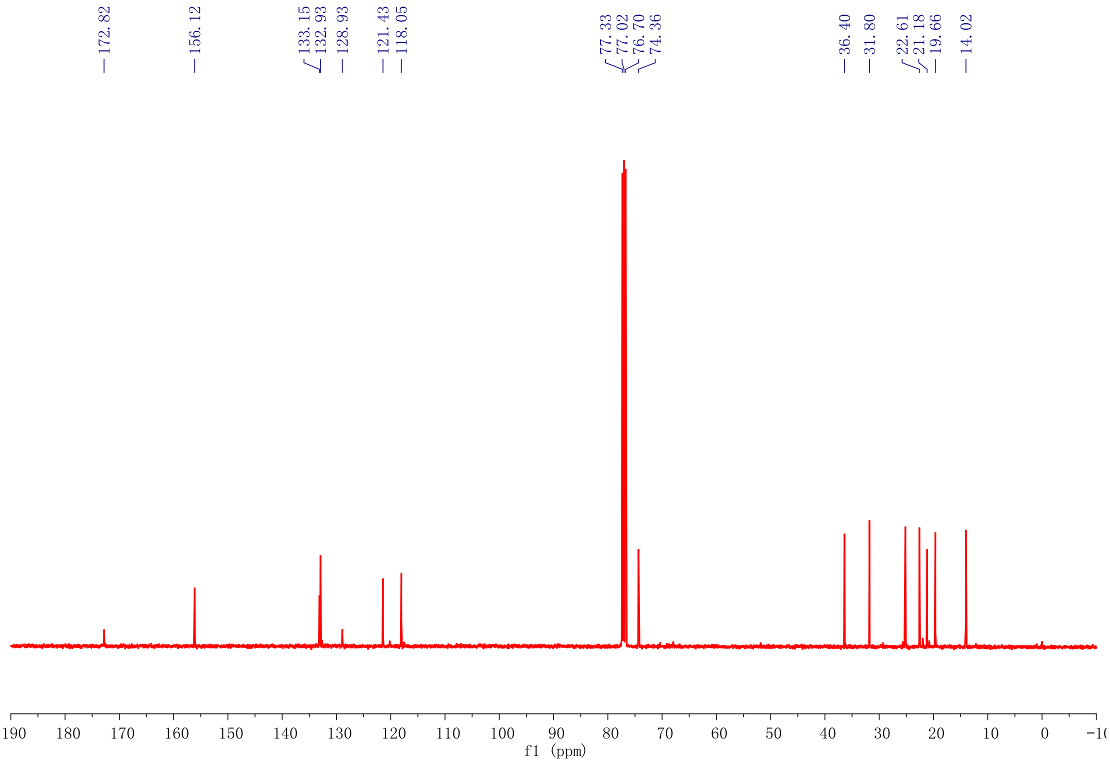

Supplementary Figure 5. ^{1}H, ^{13}C-NMR spectra of product **1e**

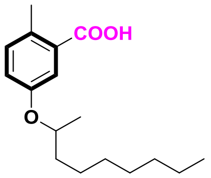

1e

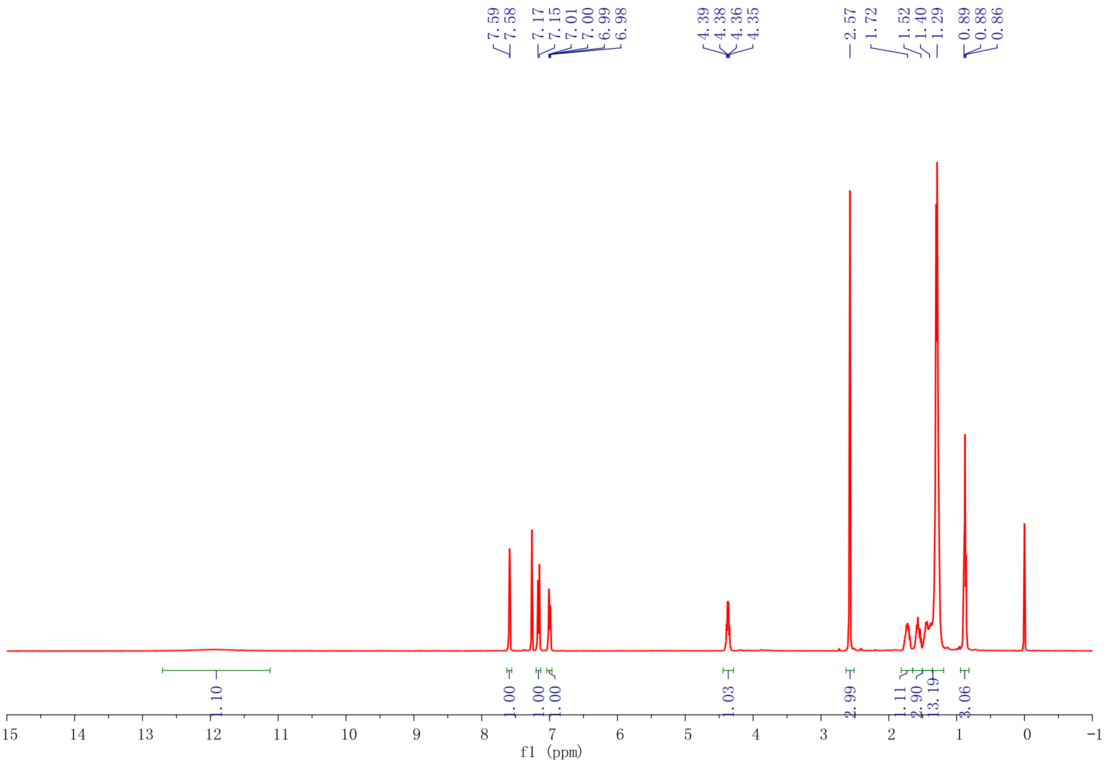

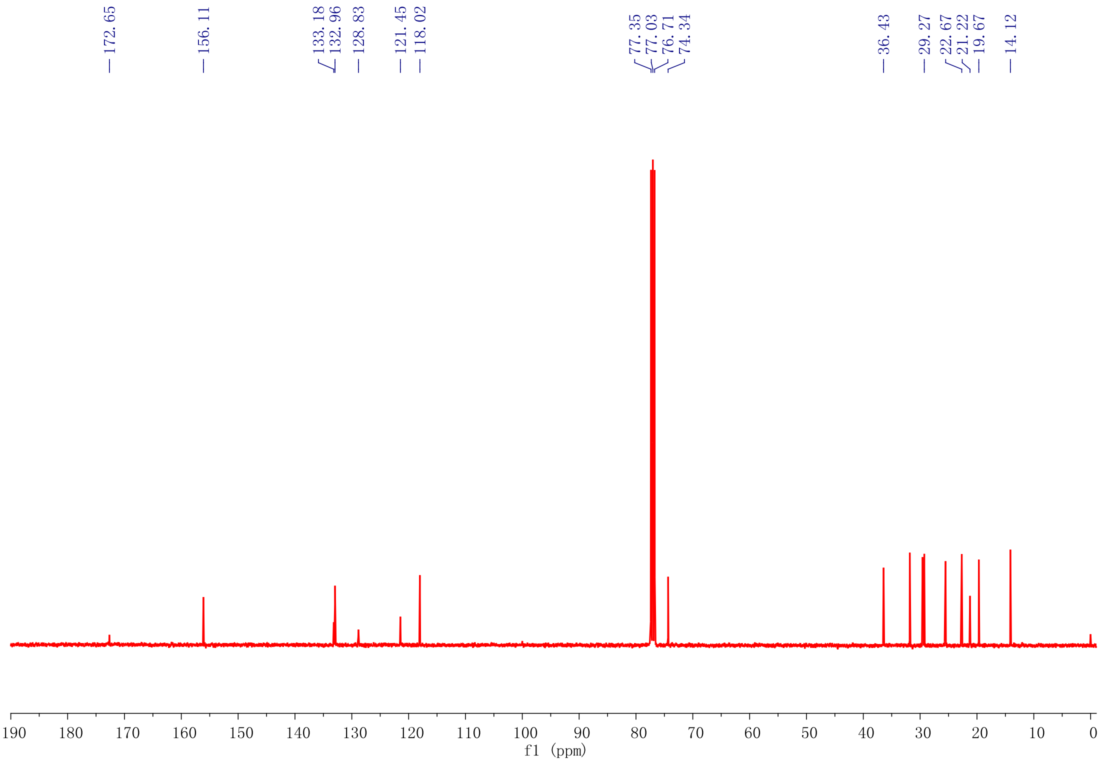

Supplementary Figure 6. ^{1}H, ^{13}C-NMR spectra of product **1f**

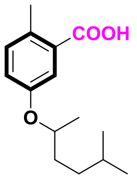

1f

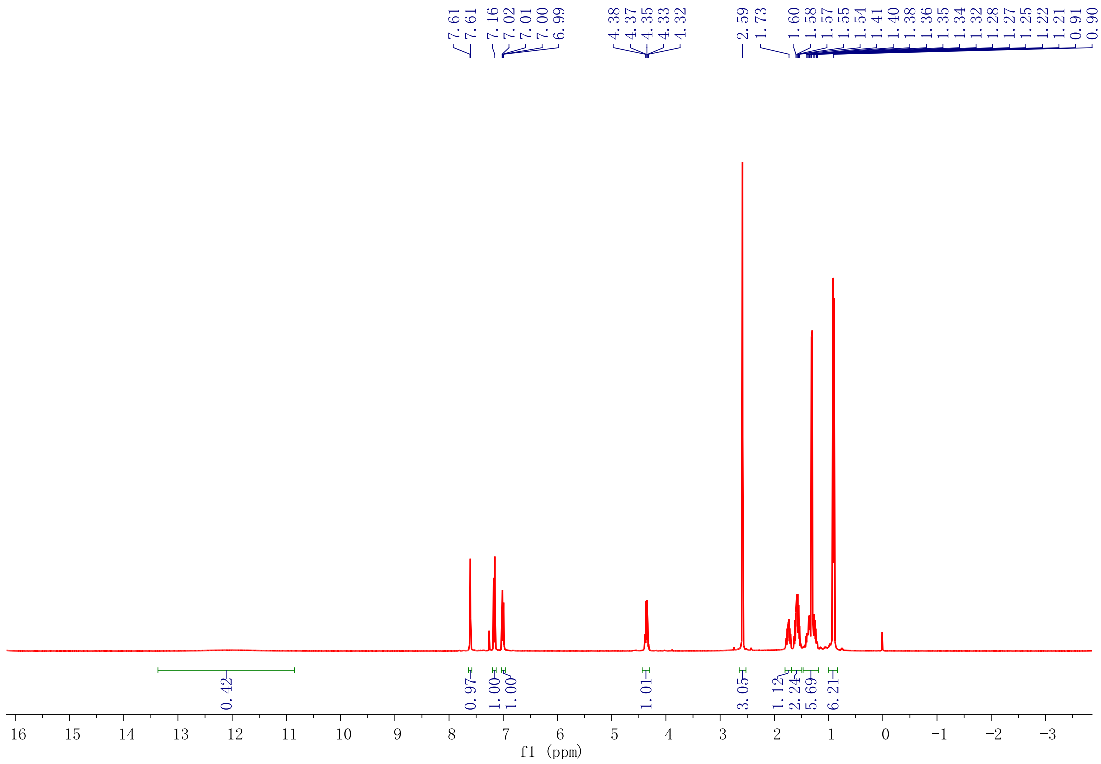

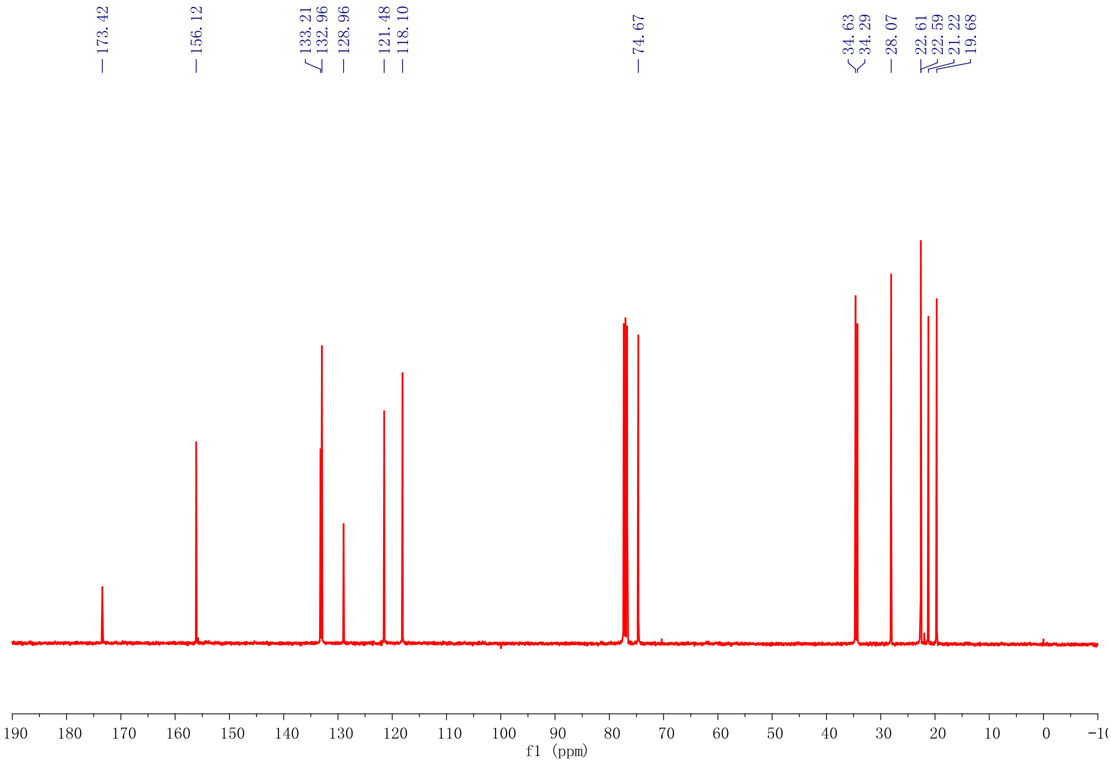

Supplementary Figure 7. ¹H, ¹³C-NMR spectra of product **1g**

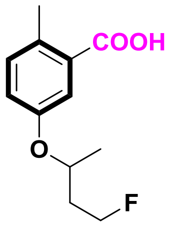

1g

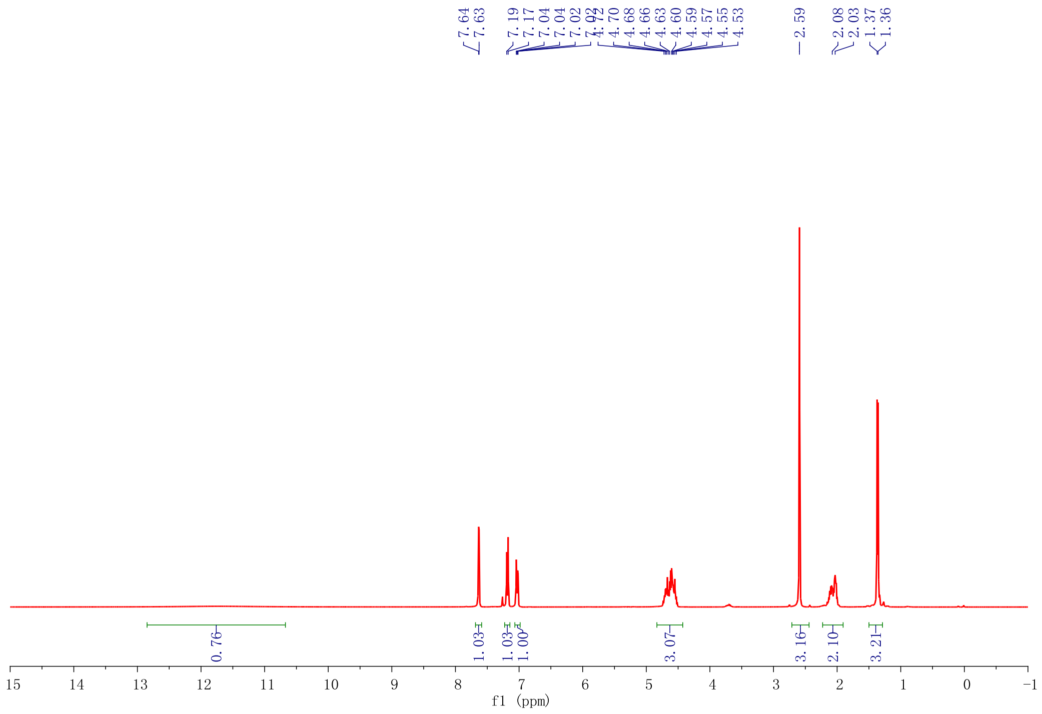

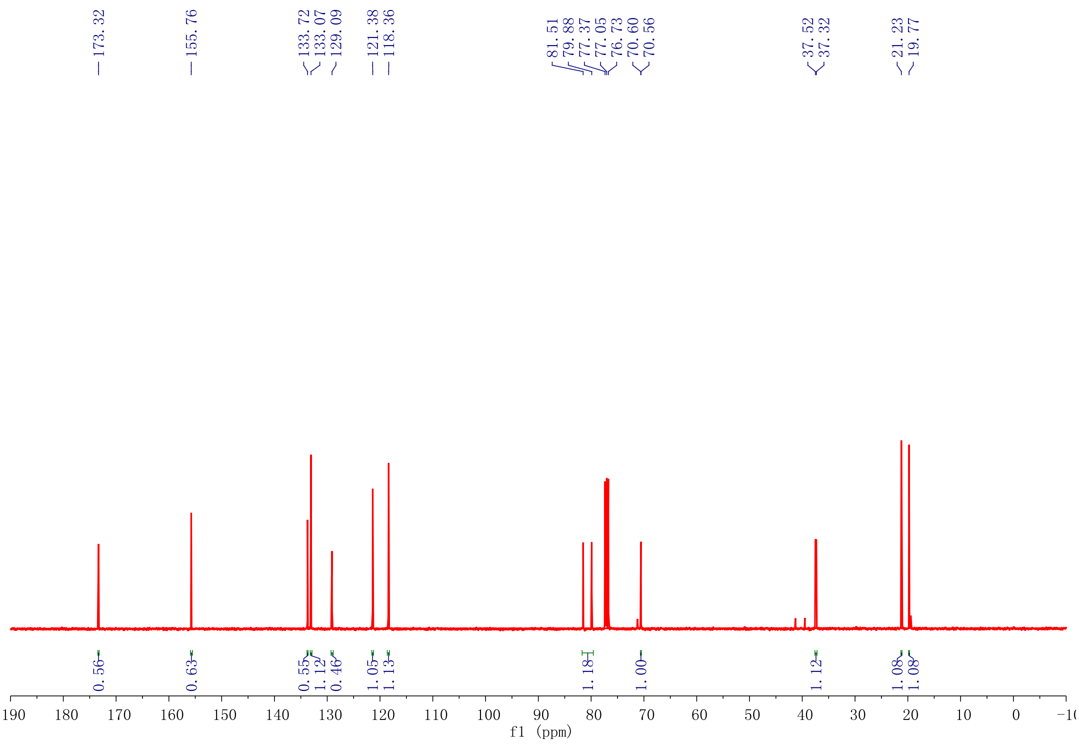

Supplementary Figure 8. ^{1}H, ^{13}C-NMR spectra of product **1h**

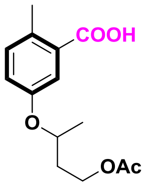

1h

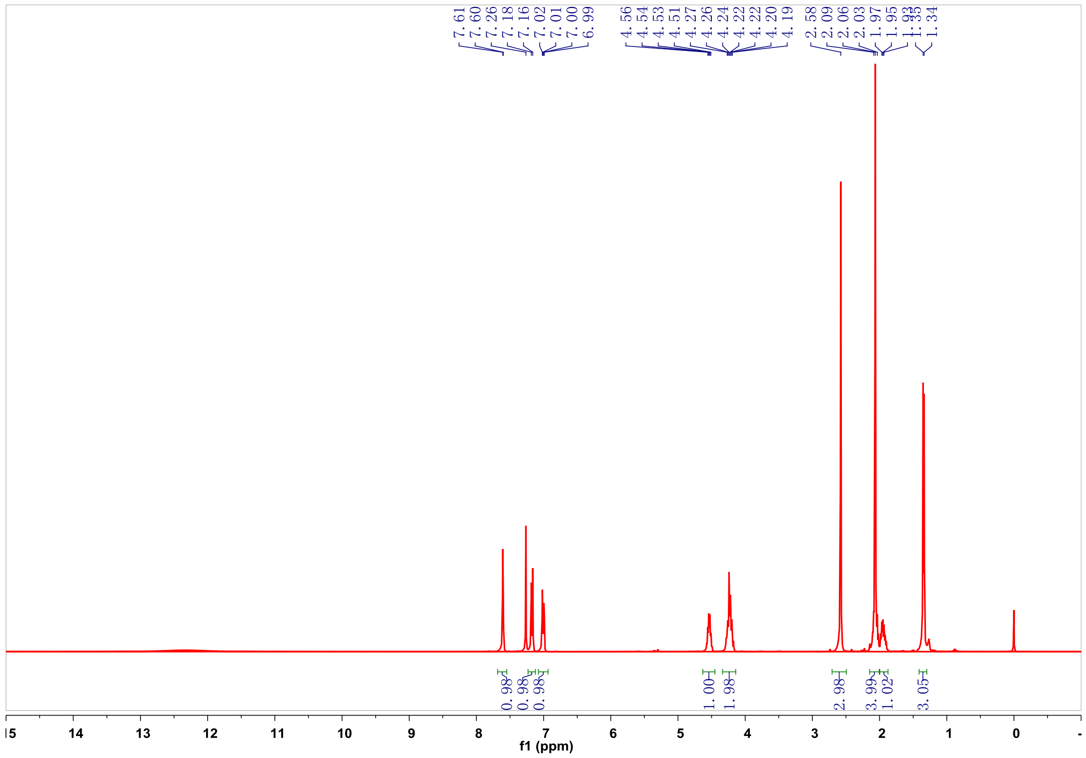

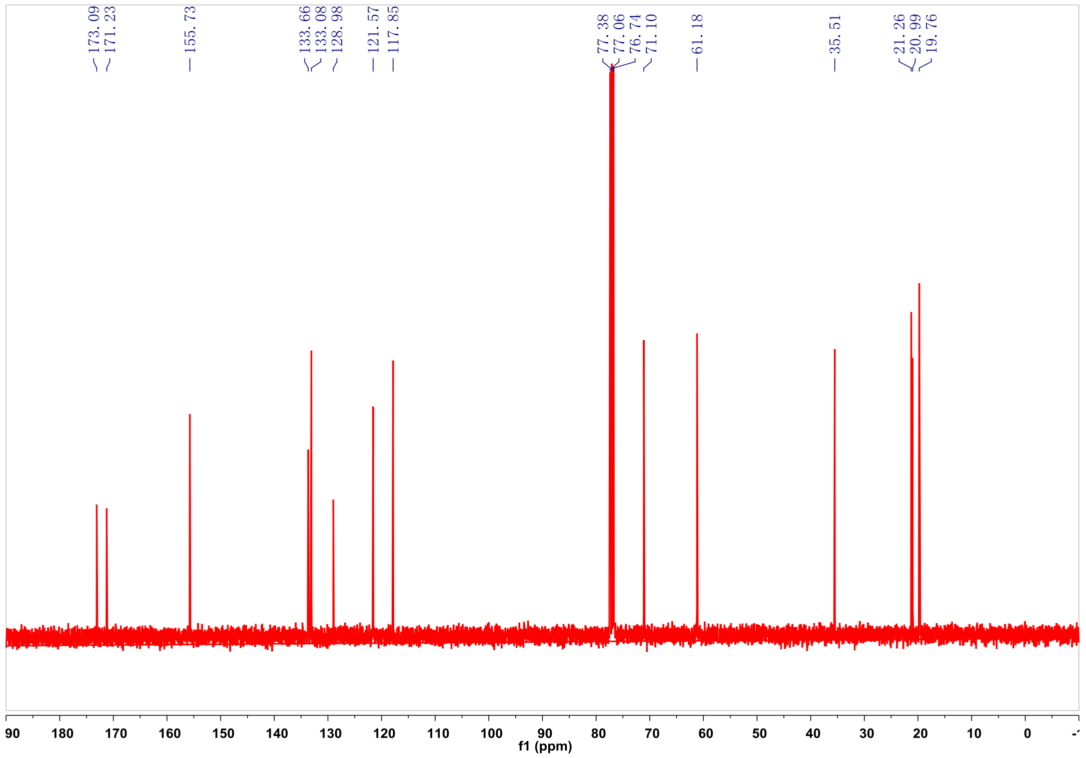

Supplementary Figure 9. ^{1}H, ^{13}C-NMR spectra of product **1i**

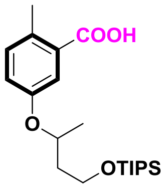

1i

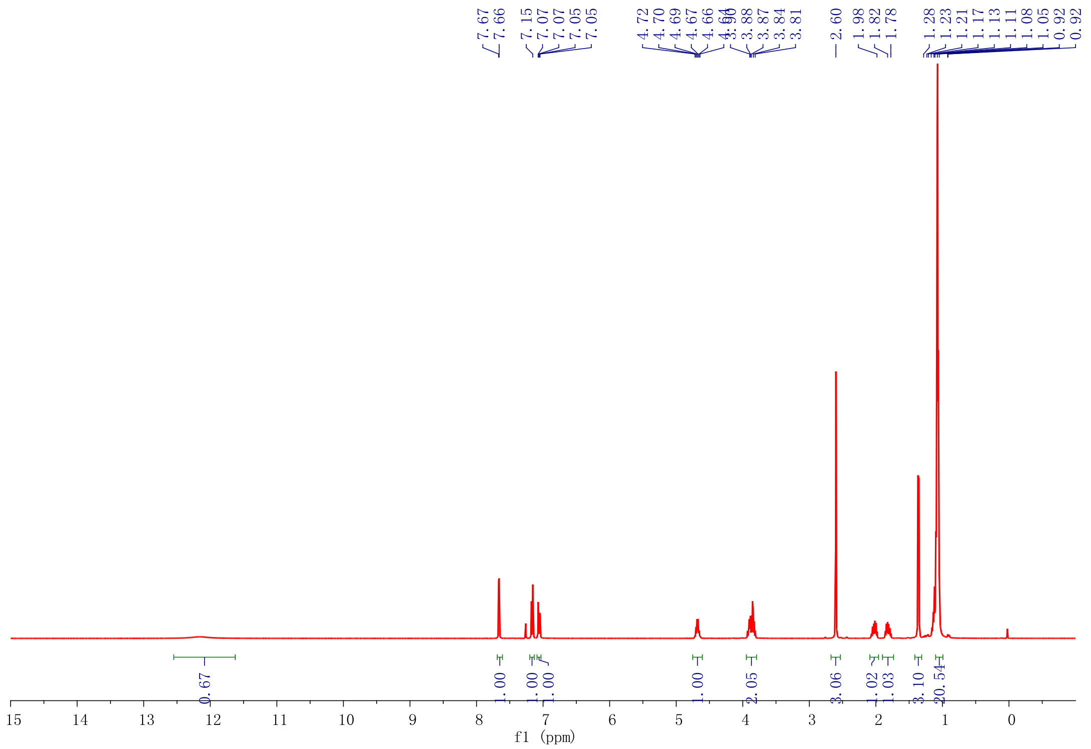

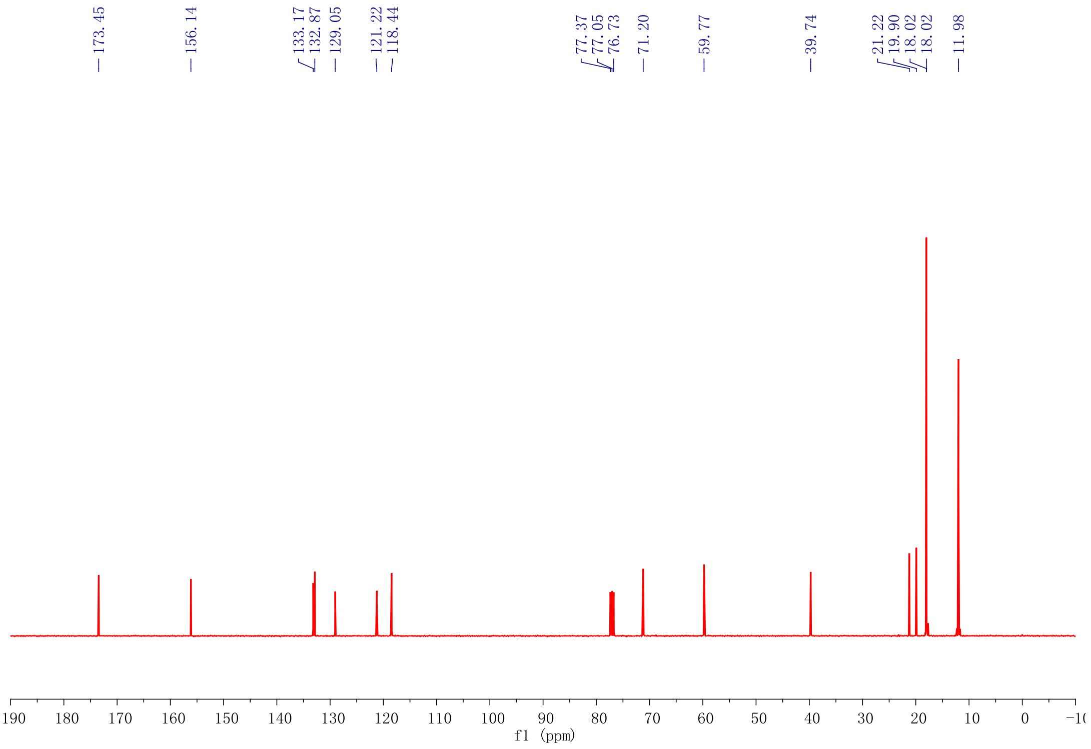

Supplementary Figure 10. ^{1}H, ^{13}C-NMR spectra of product **1j**

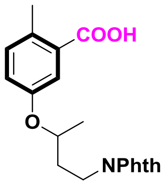

1j

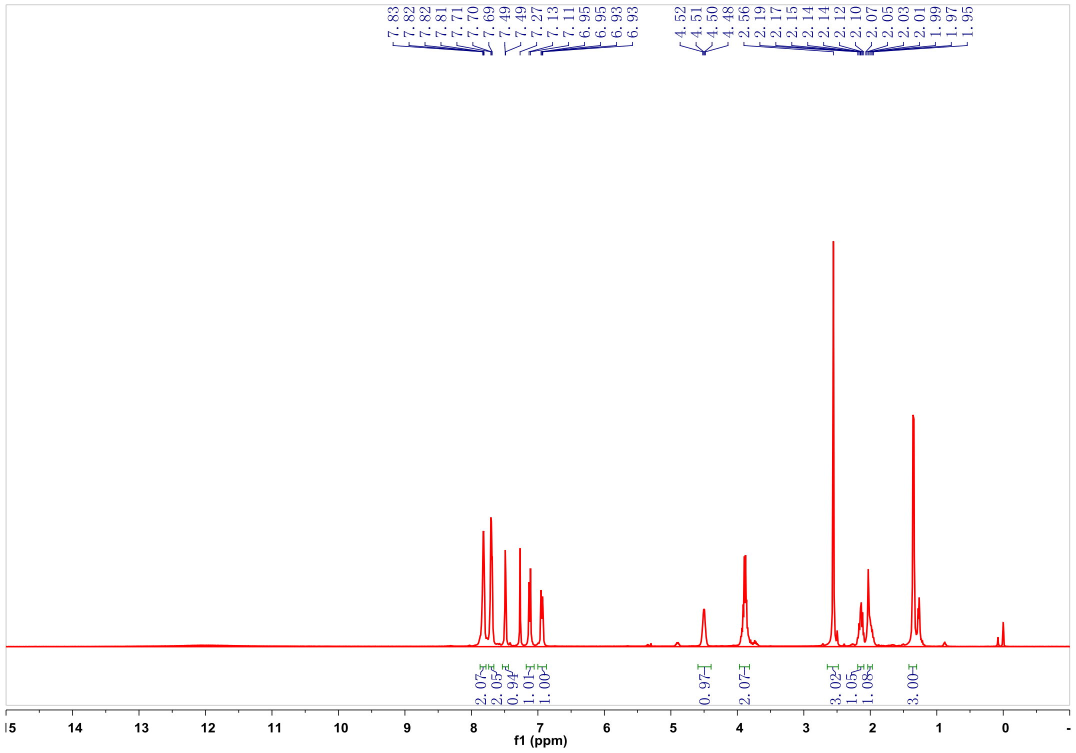

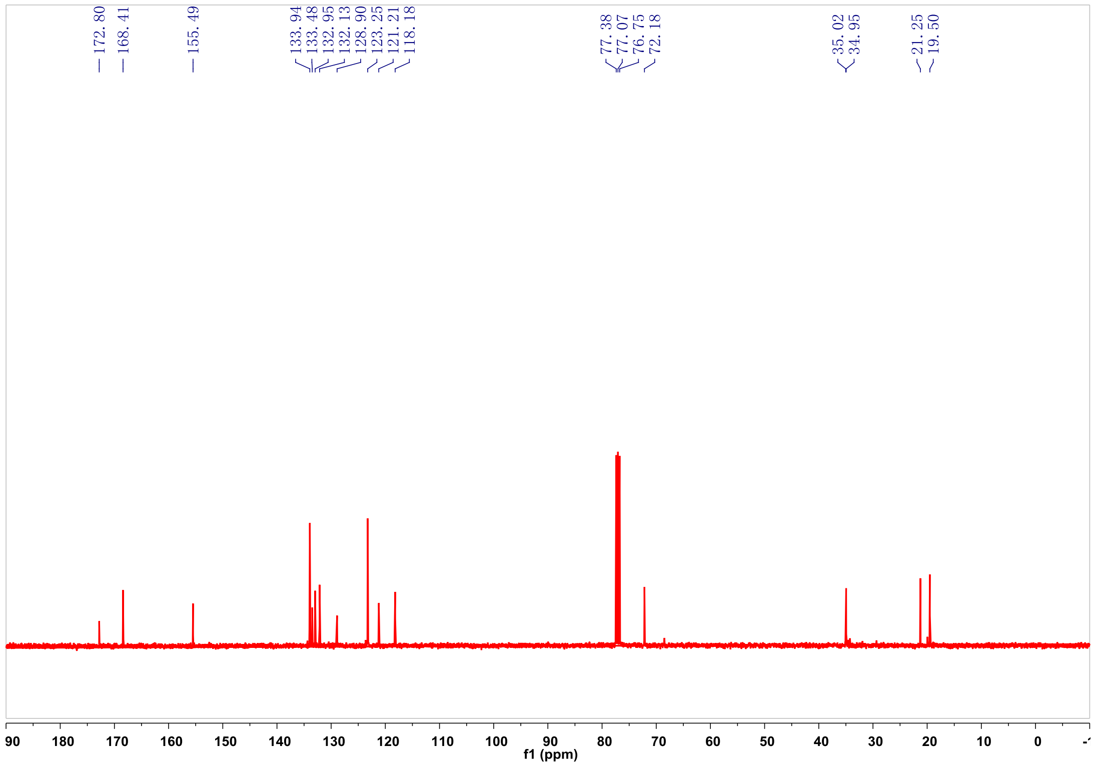

Supplementary Figure 11. ^{1}H, ^{13}C-NMR spectra of product **1k**

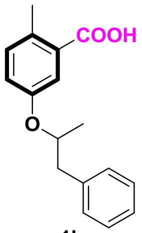

1k

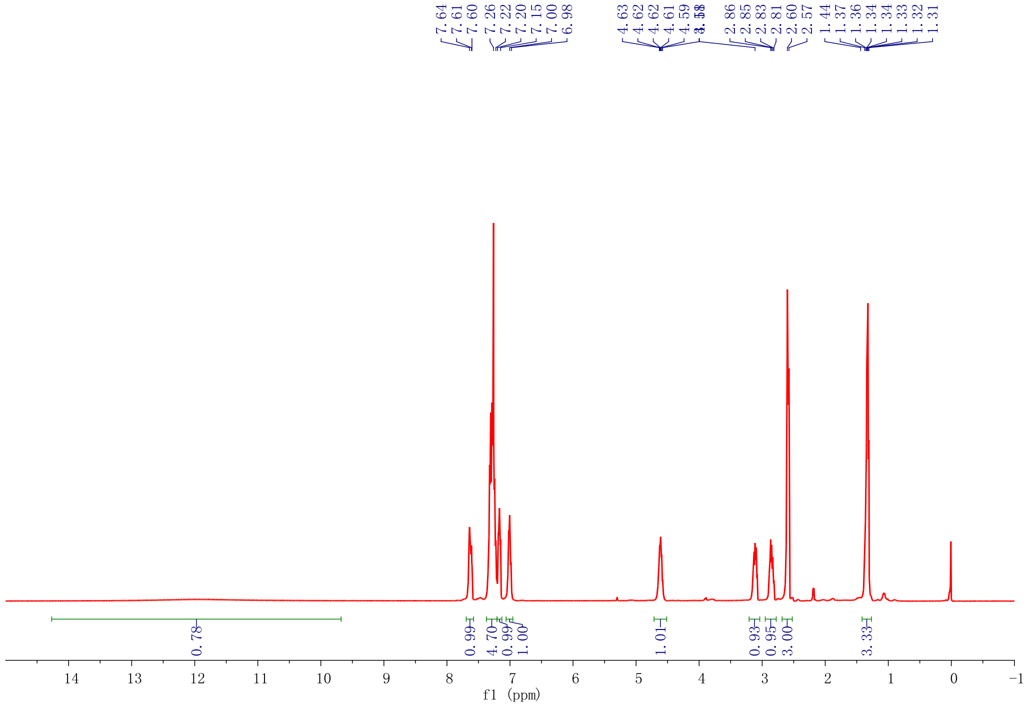

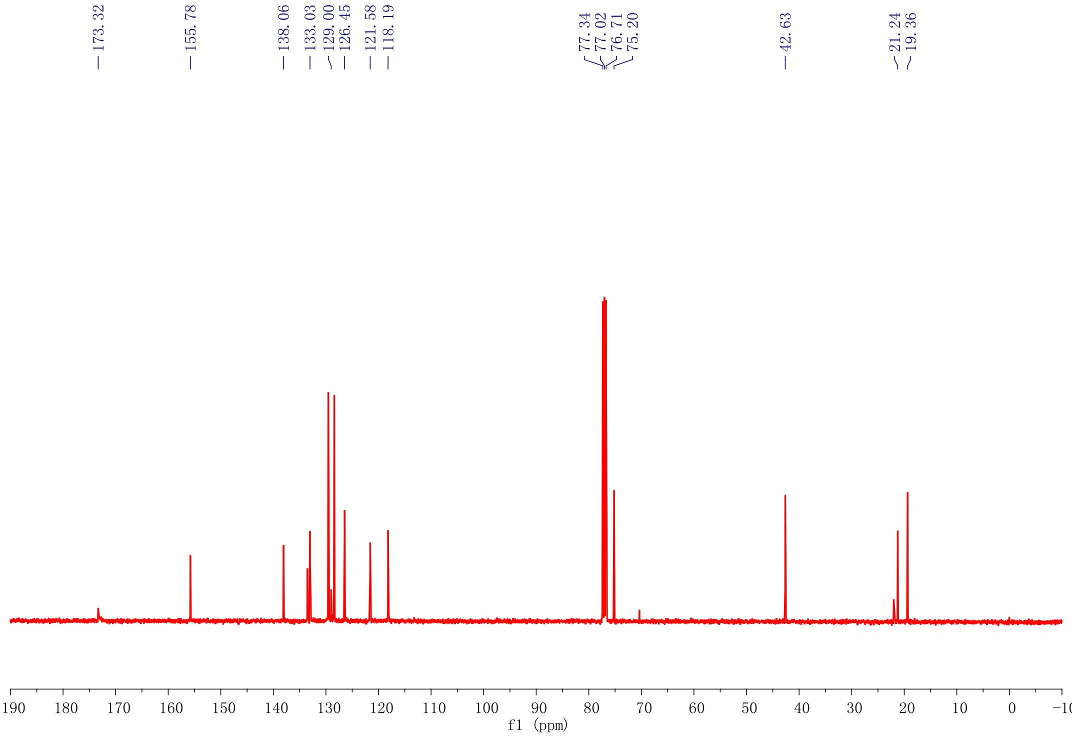

Supplementary Figure 12. ^{1}H, ^{13}C-NMR spectra of product **1l**

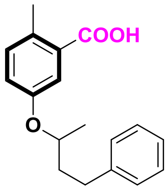

1l

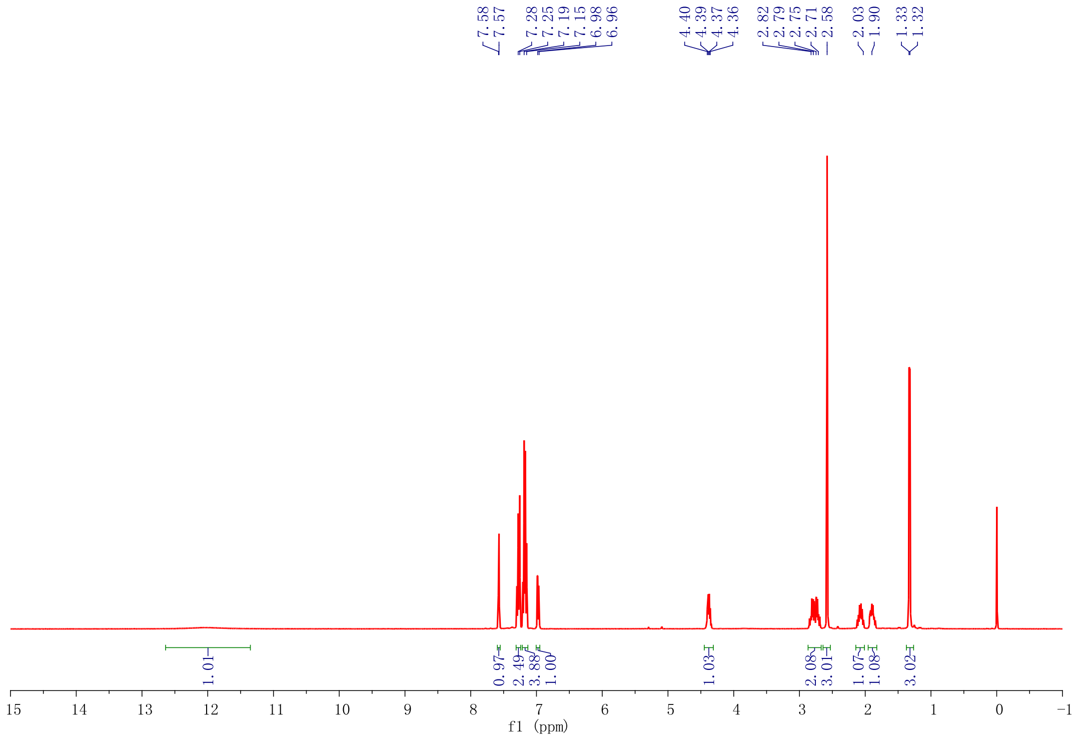

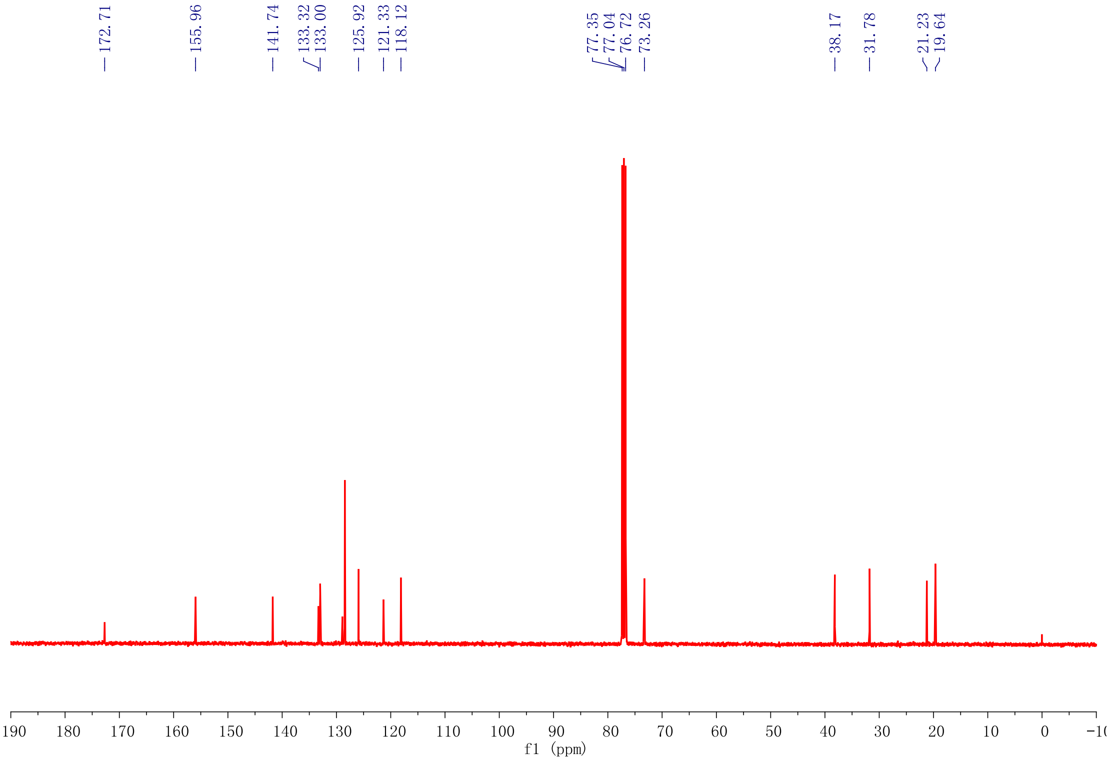

Supplementary Figure 13. ^{1}H, ^{13}C-NMR spectra of product **1m**

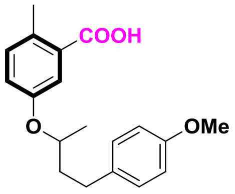

1m

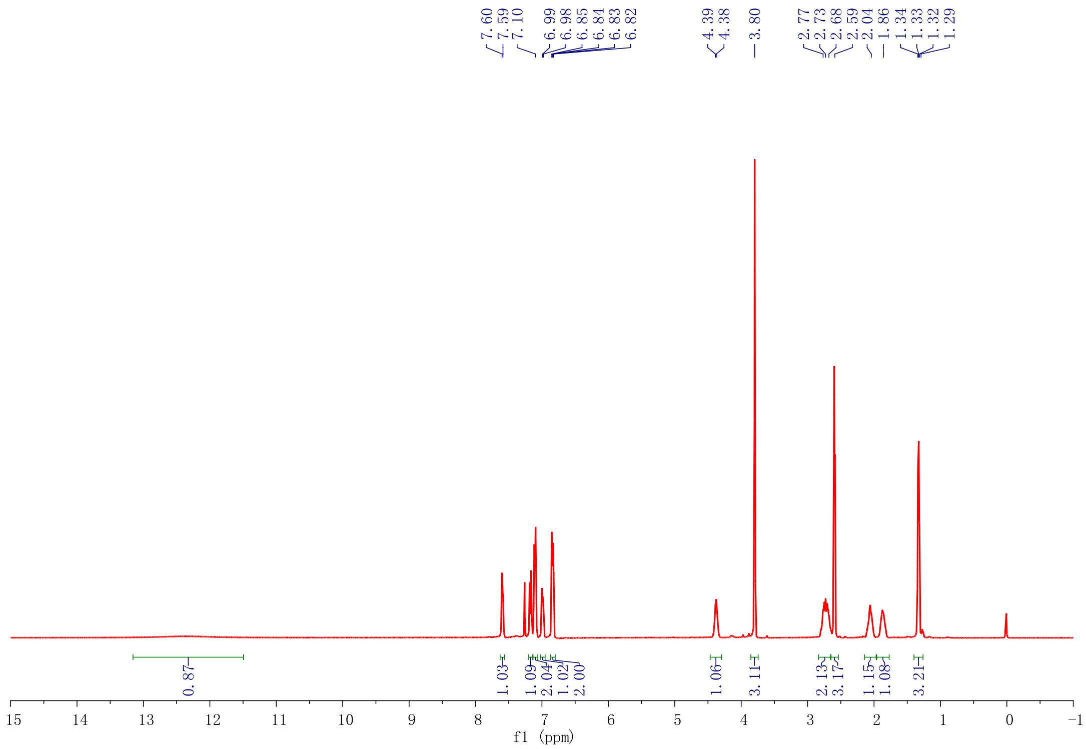

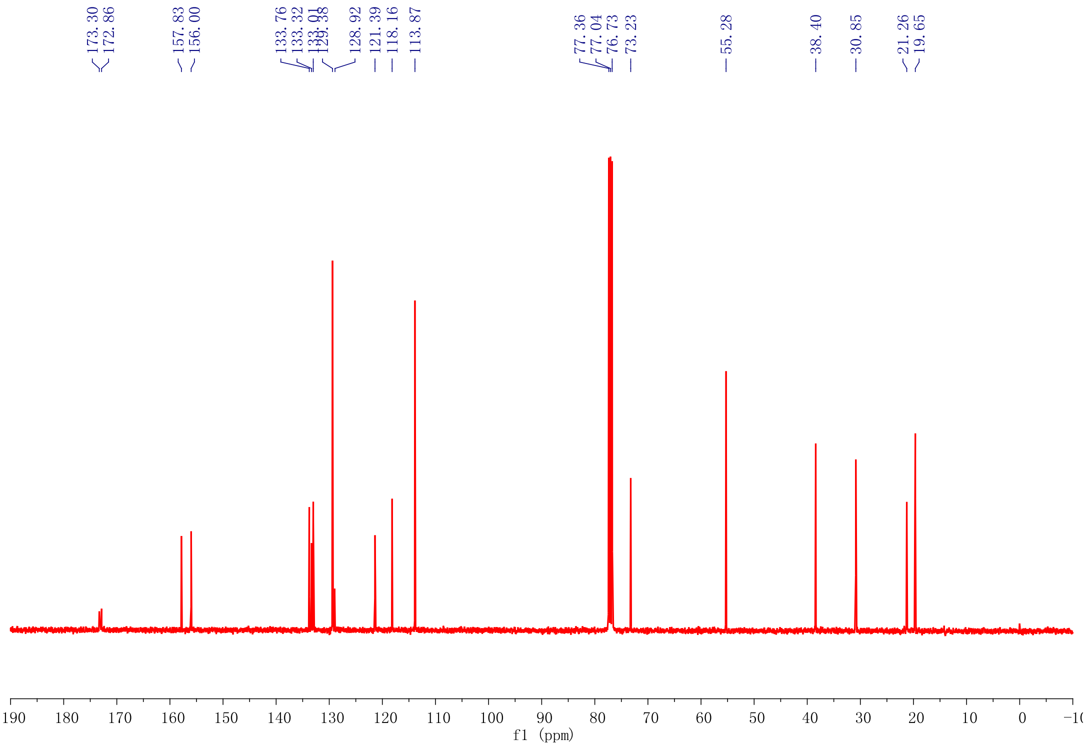

Supplementary Figure 14. ^{1}H, ^{13}C-NMR spectra of product **1n**

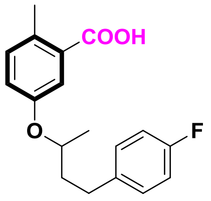

1n

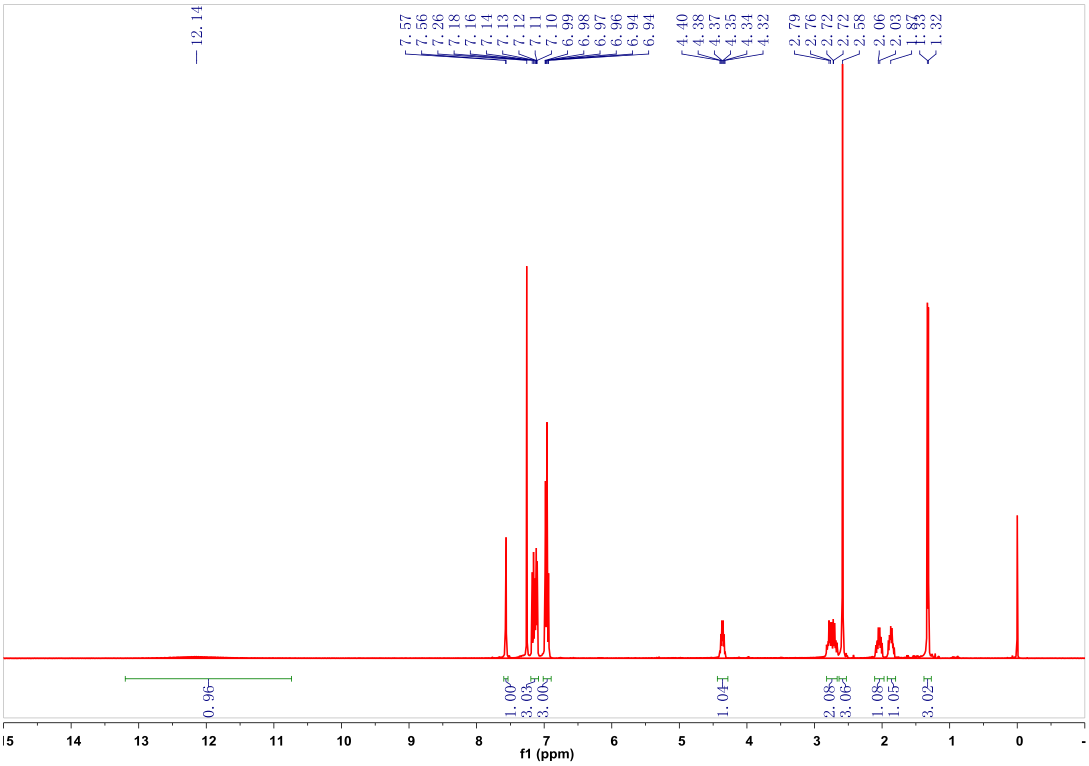

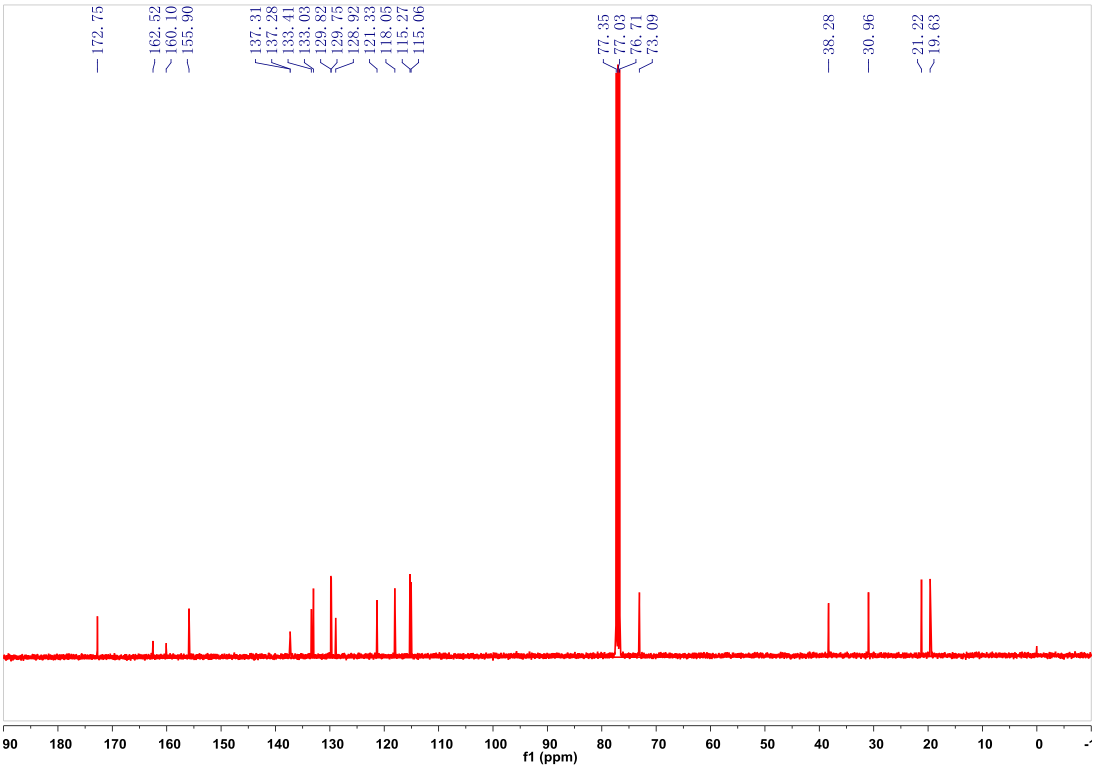

Supplementary Figure 15. ^{1}H, ^{13}C-NMR spectra of product **1o**

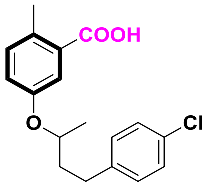

1o

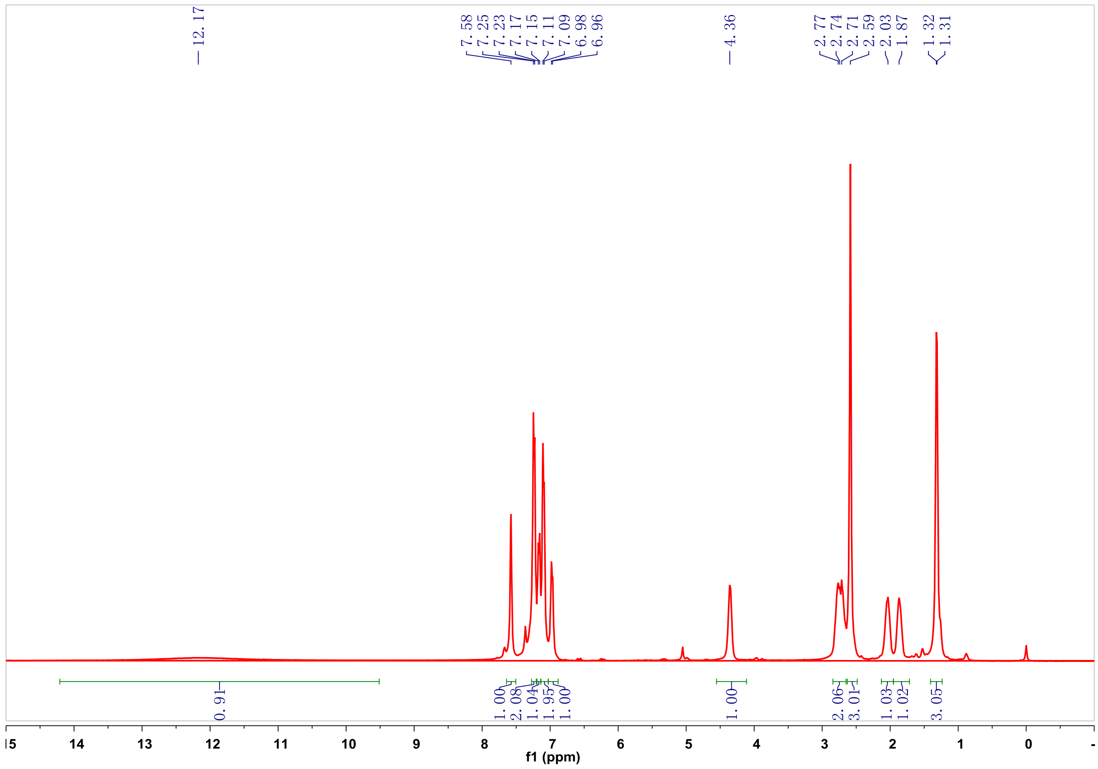

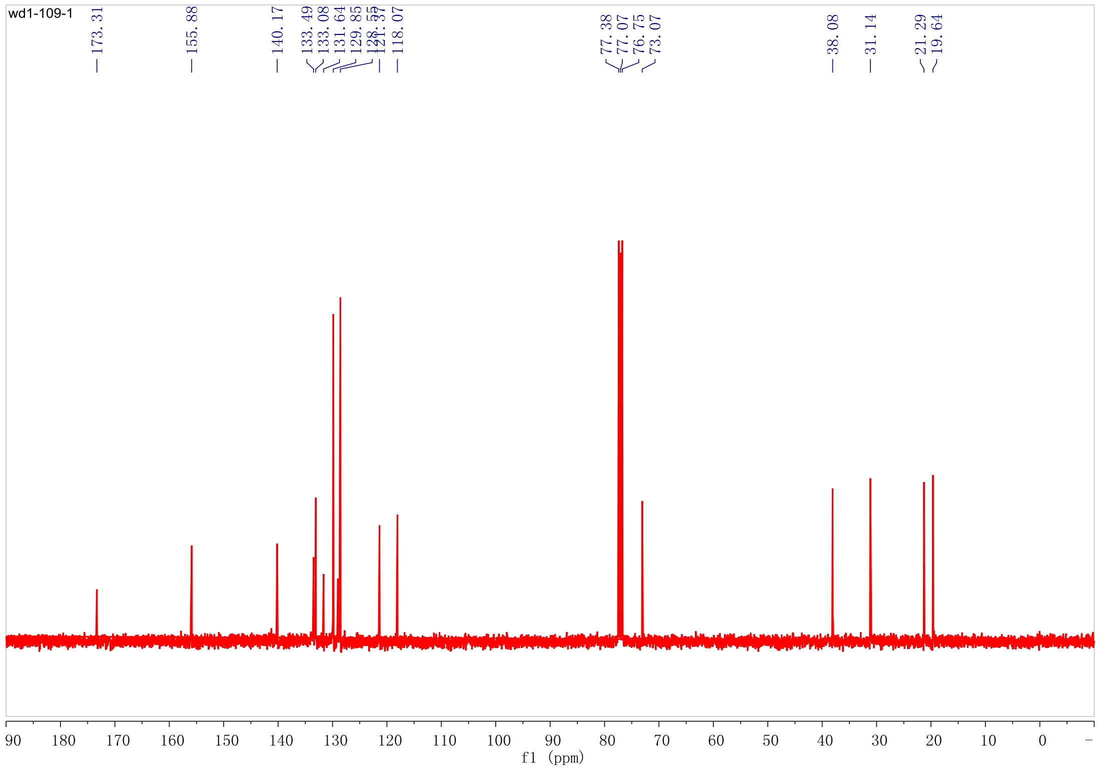

Supplementary Figure 16. ^{1}H, ^{13}C-NMR spectra of product **1p**

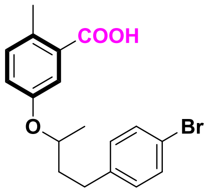

1p

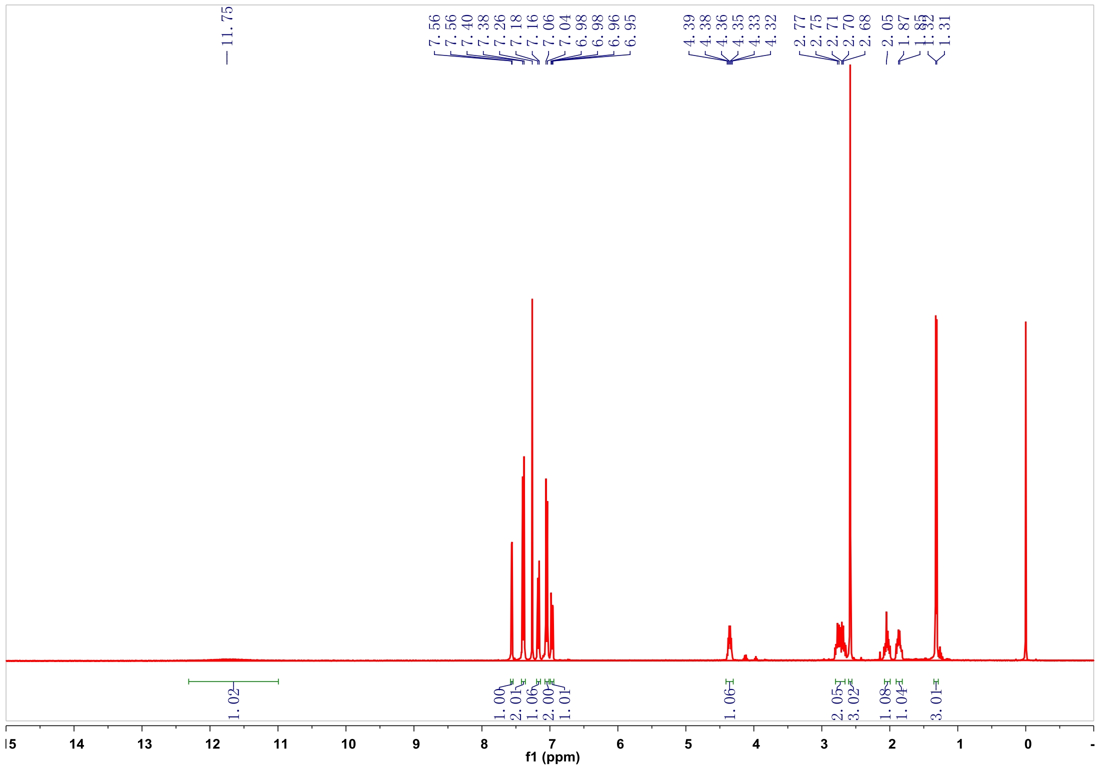

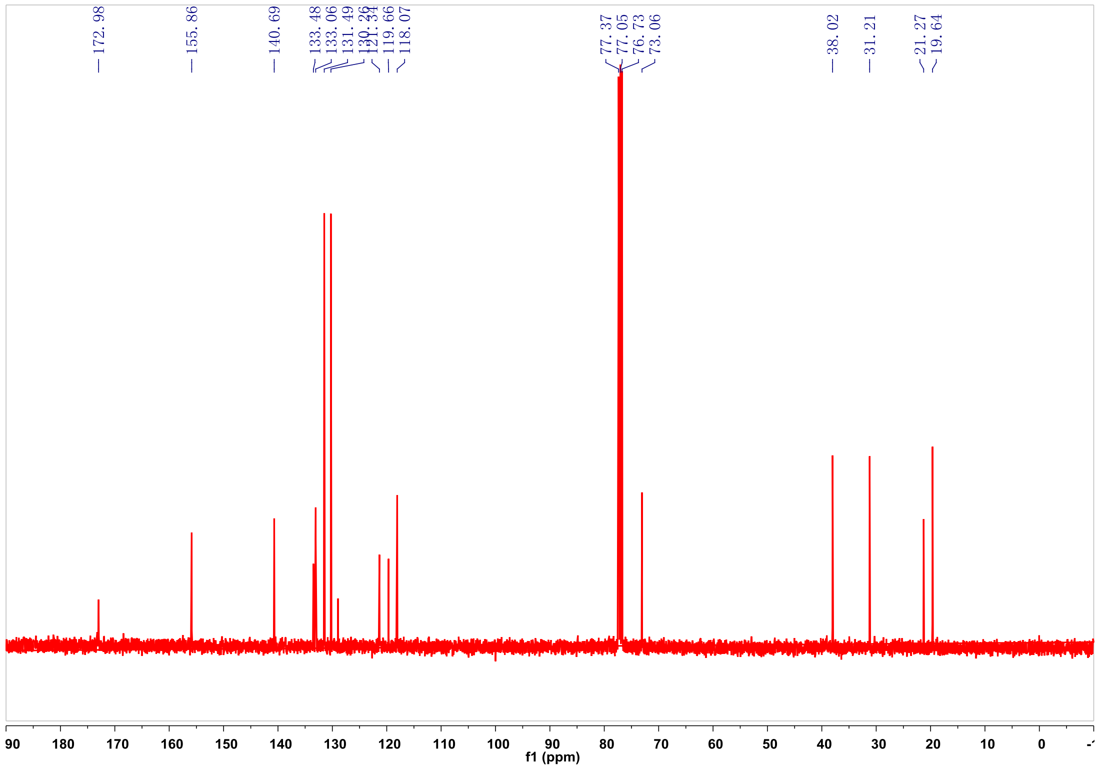

Supplementary Figure 17. ^{1}H, ^{13}C-NMR spectra of product **1q**

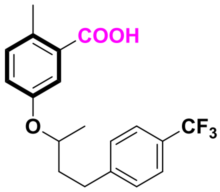

1q

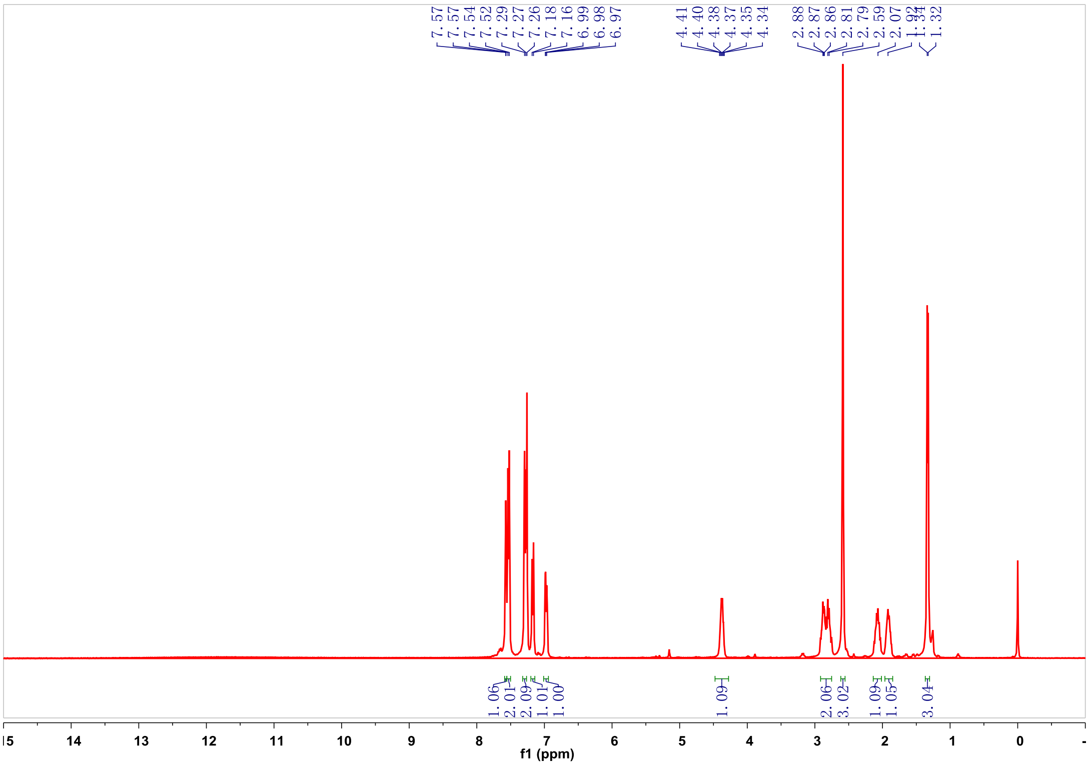

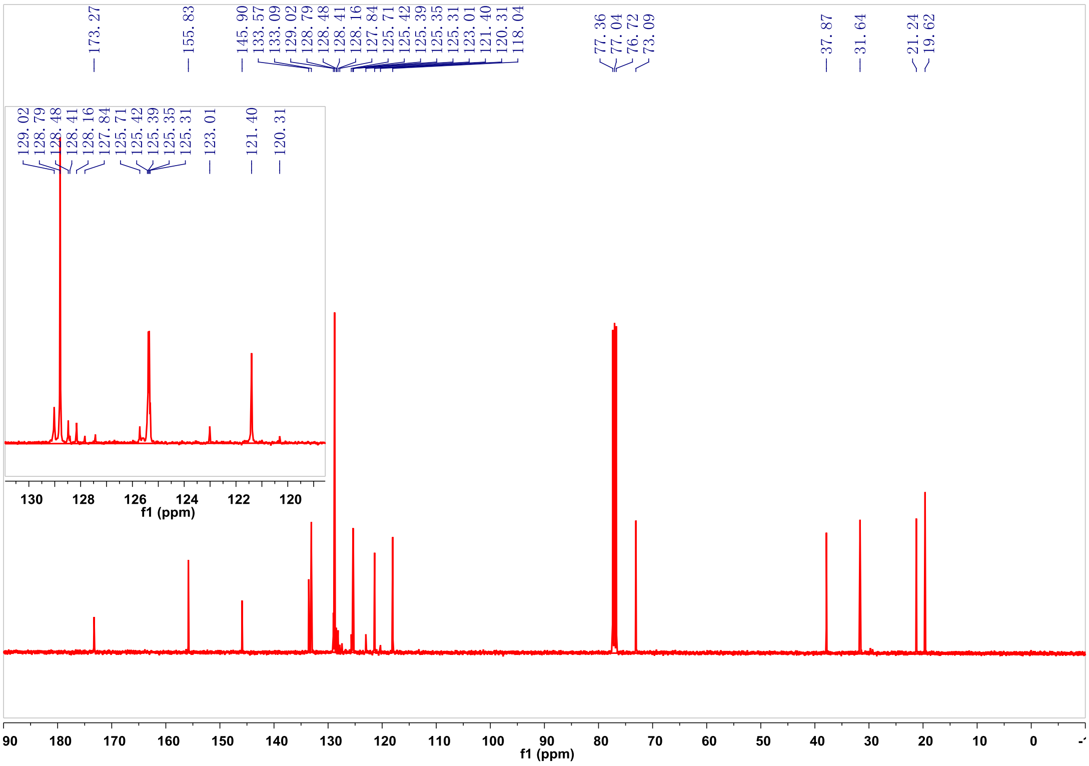

Supplementary Figure 18. ^{1}H, ^{13}C-NMR spectra of product **1r**

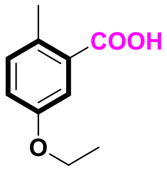

1r

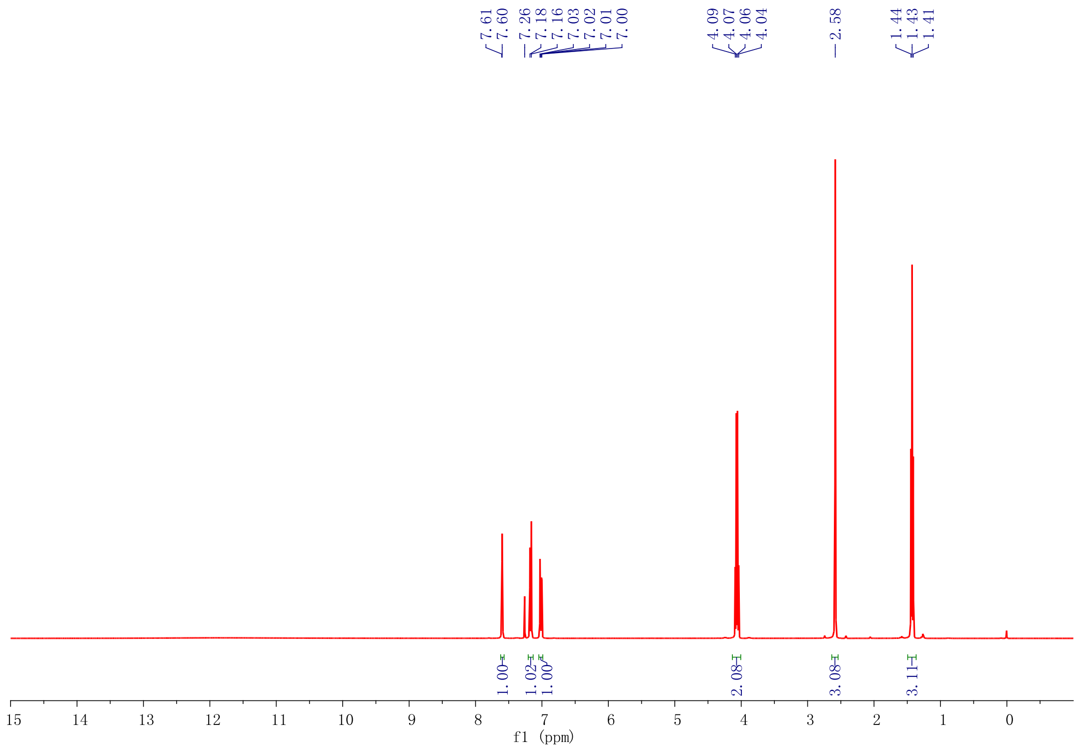

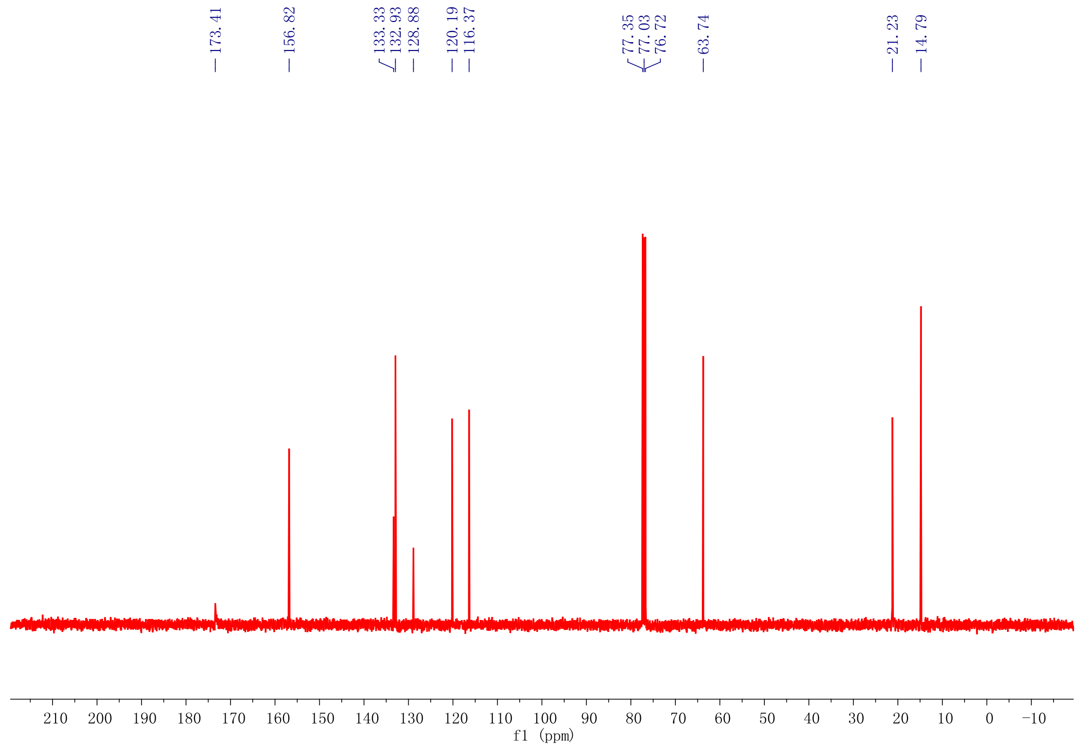

Supplementary Figure 19. ^{1}H, ^{13}C-NMR spectra of product **1s**

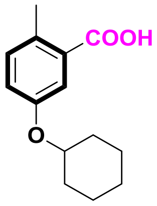

1s

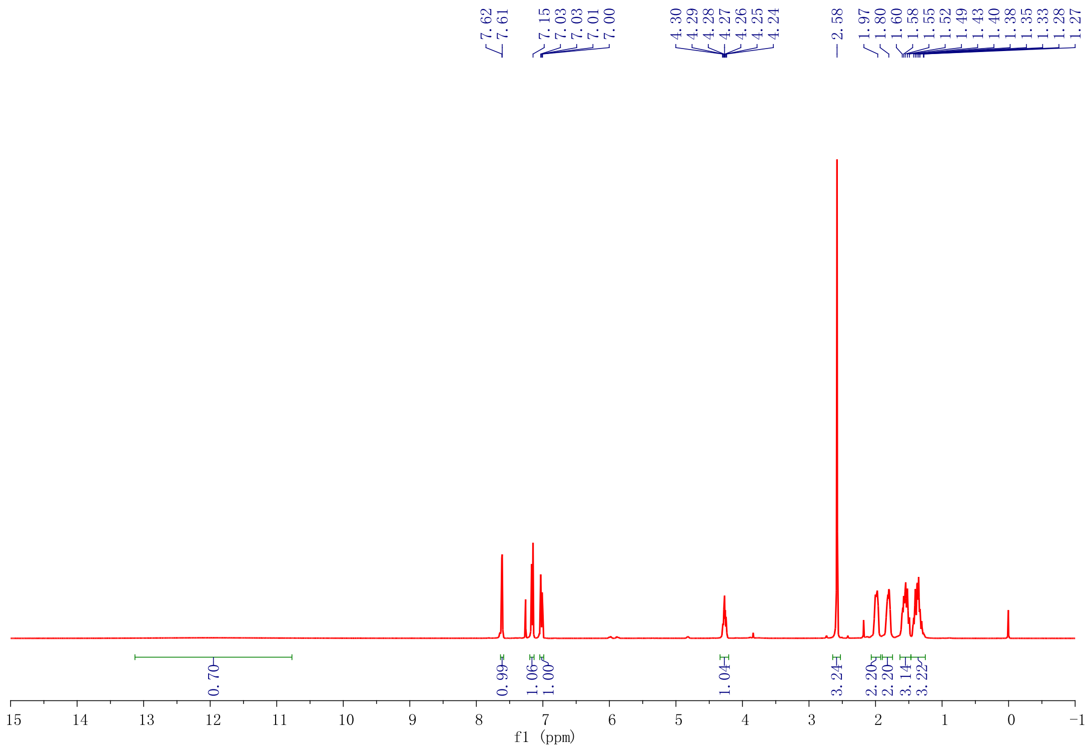

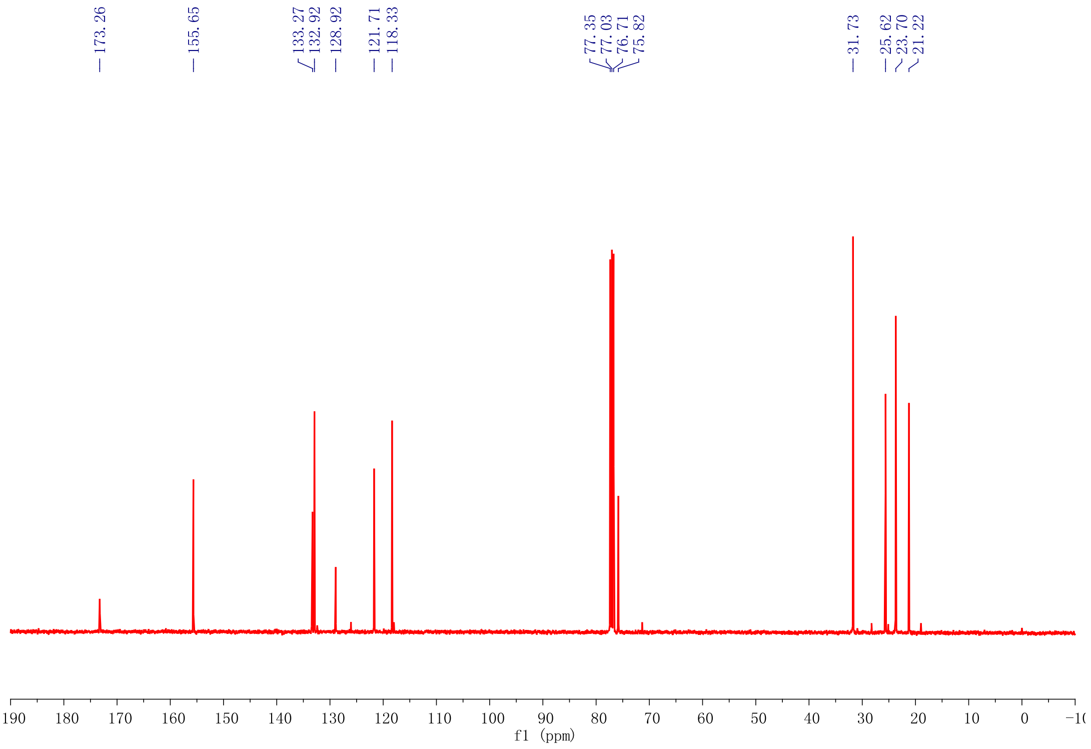

Supplementary Figure 20. ^{1}H, ^{13}C-NMR spectra of product **1t**

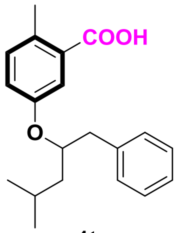

1t

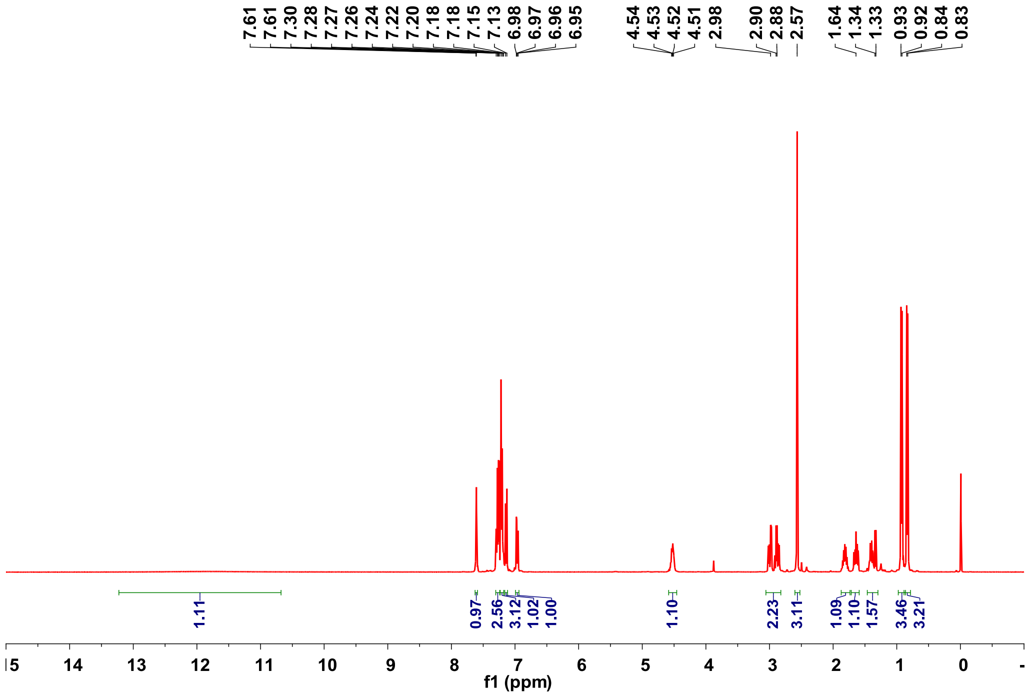

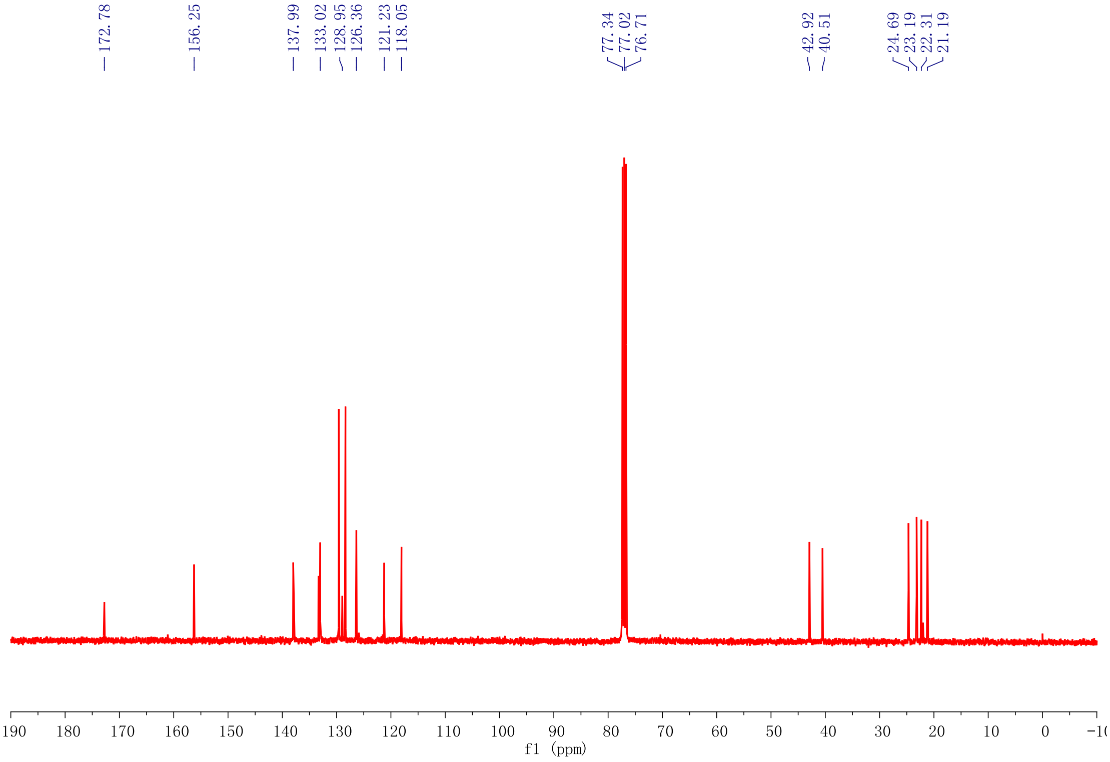

Supplementary Figure 21. ^{1}H, ^{13}C-NMR spectra of product **1u**

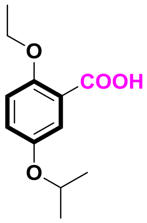

1u

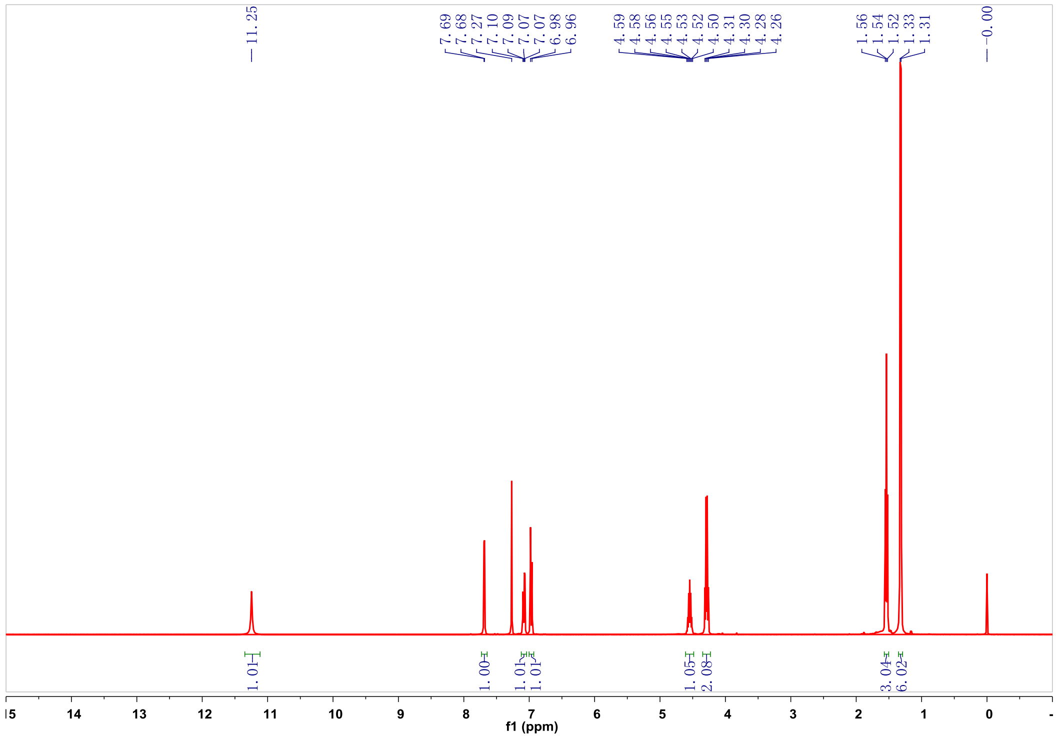

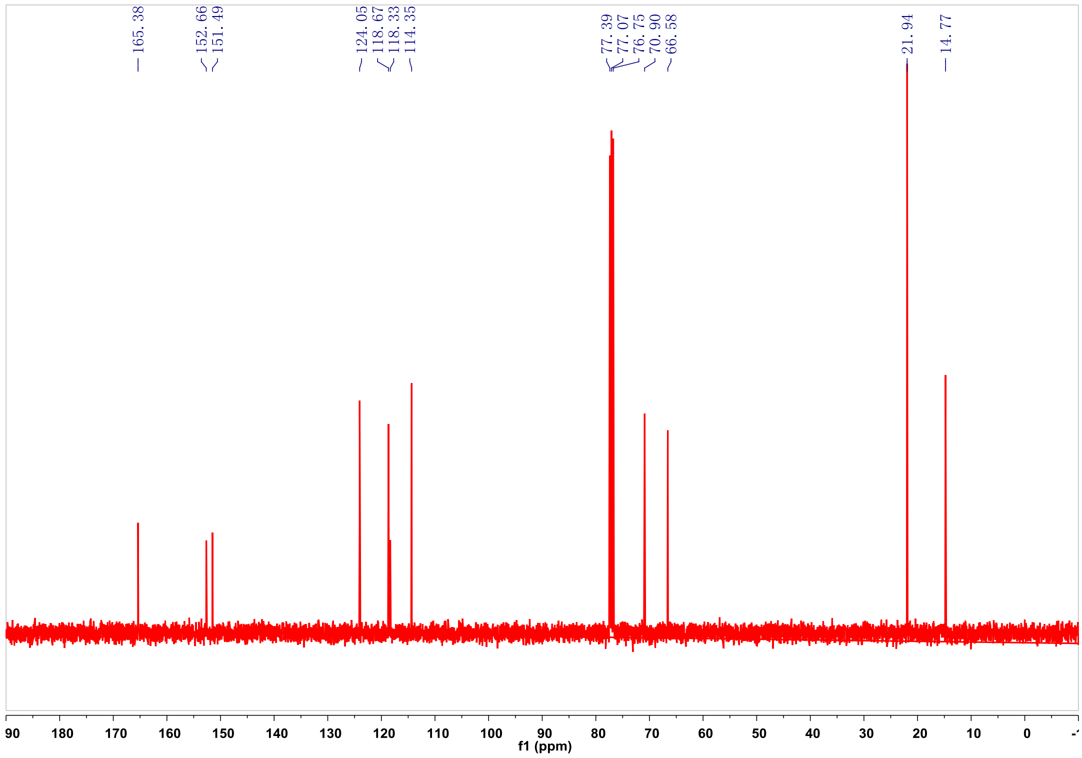

Supplementary Figure 22. ^{1}H, ^{13}C-NMR spectra of product **1v**

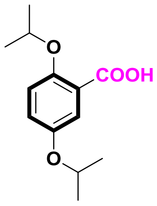

1v

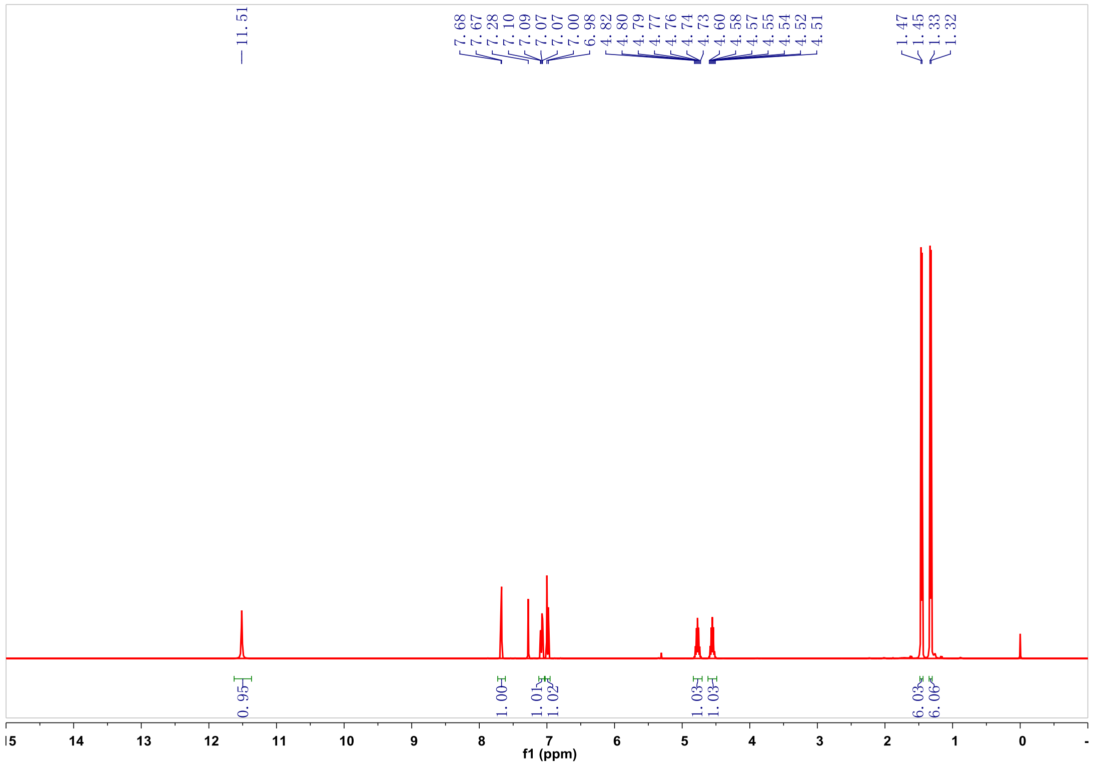

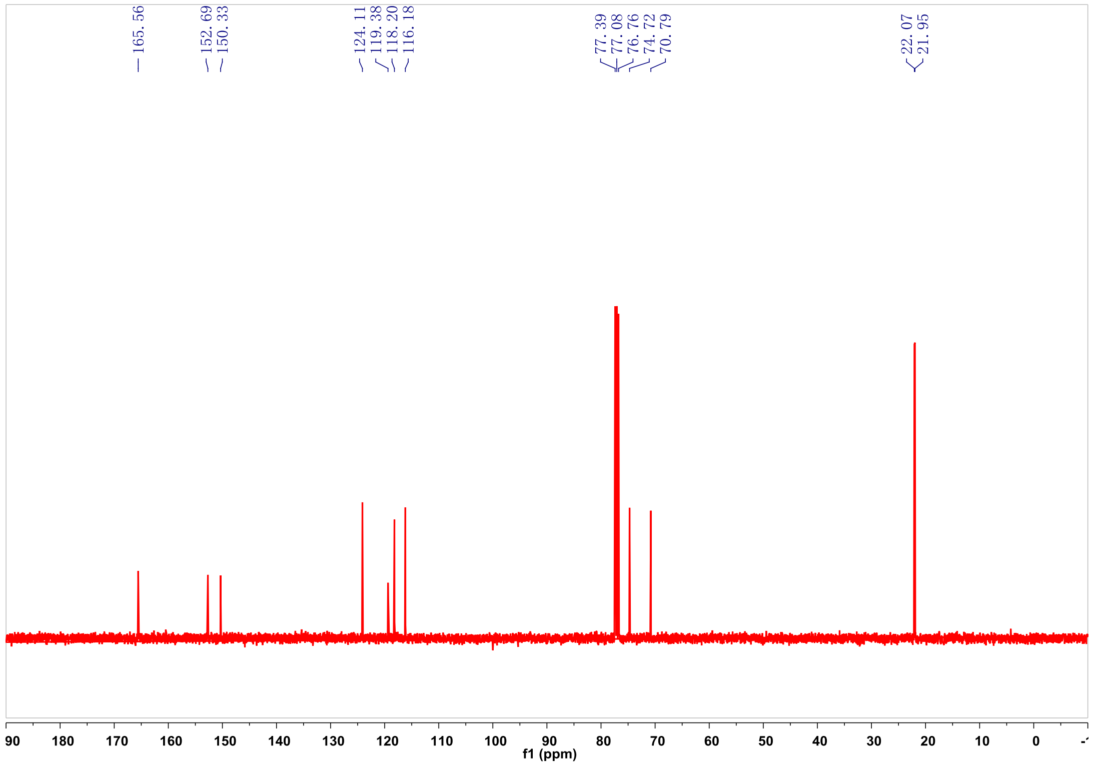

Supplementary Figure 23. ^{1}H, ^{13}C-NMR spectra of product **1w**

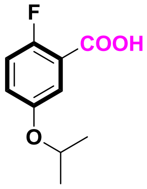

1w

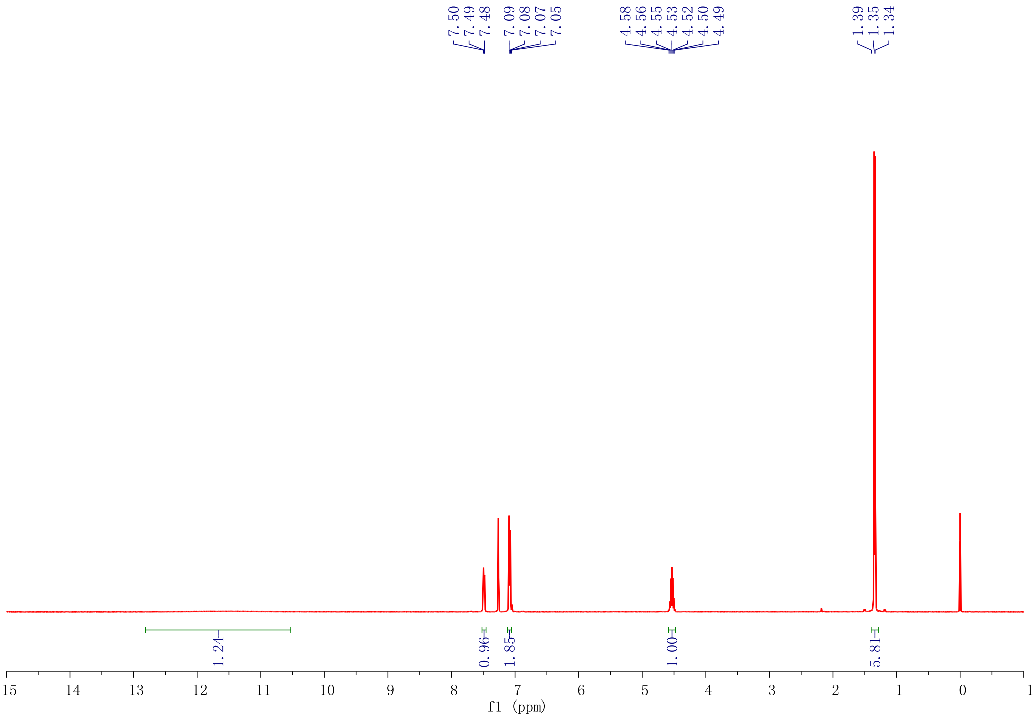

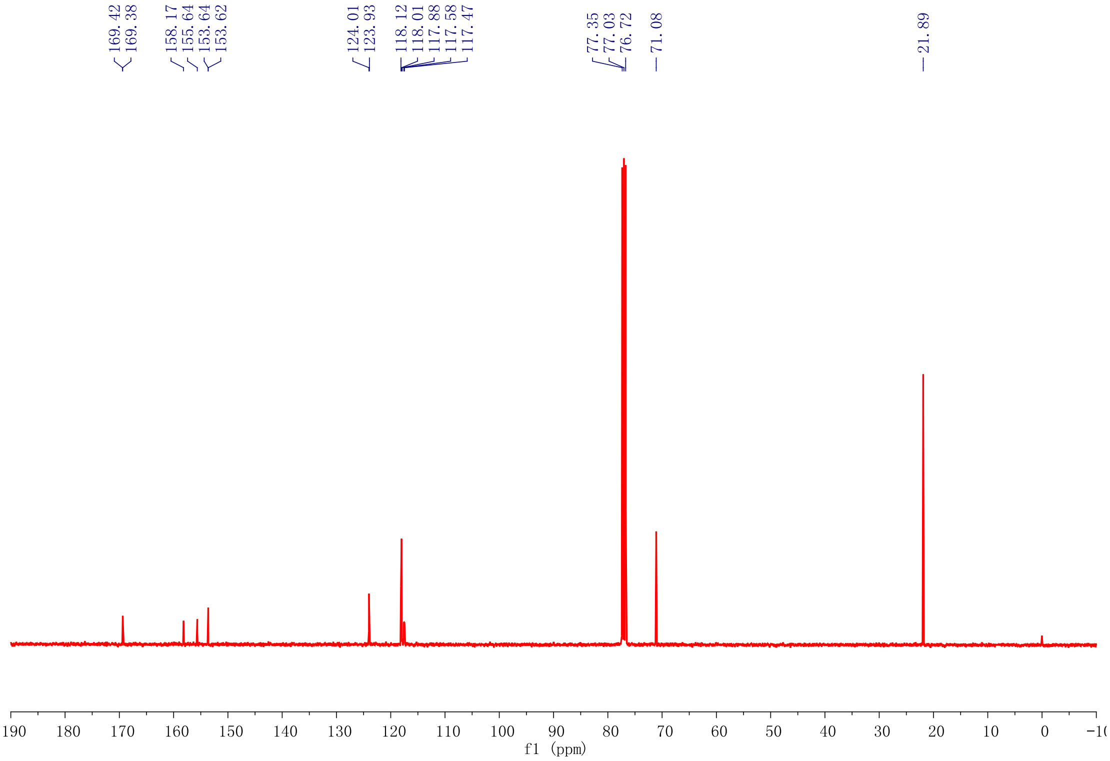

Supplementary Figure 24. ^{1}H, ^{13}C-NMR spectra of product **1x**

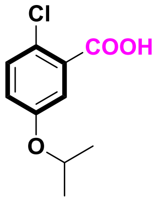

1x

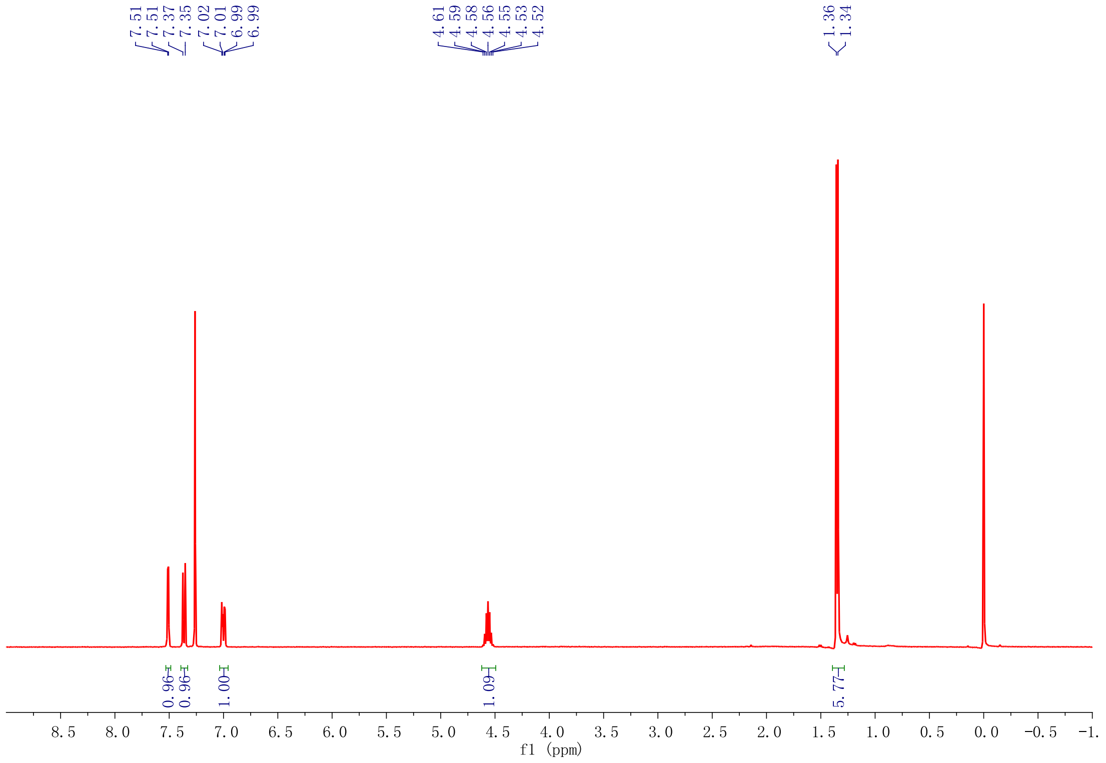

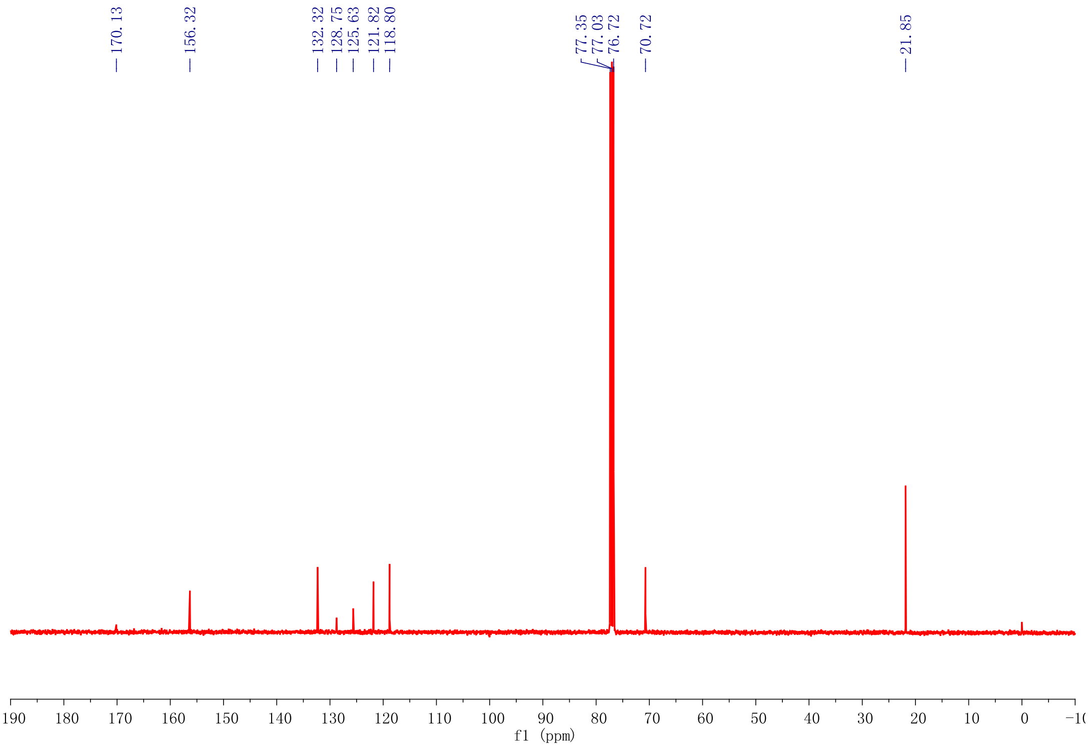

Supplementary Figure 25. ^{1}H, ^{13}C-NMR spectra of product **1y**

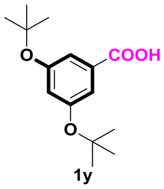

1y

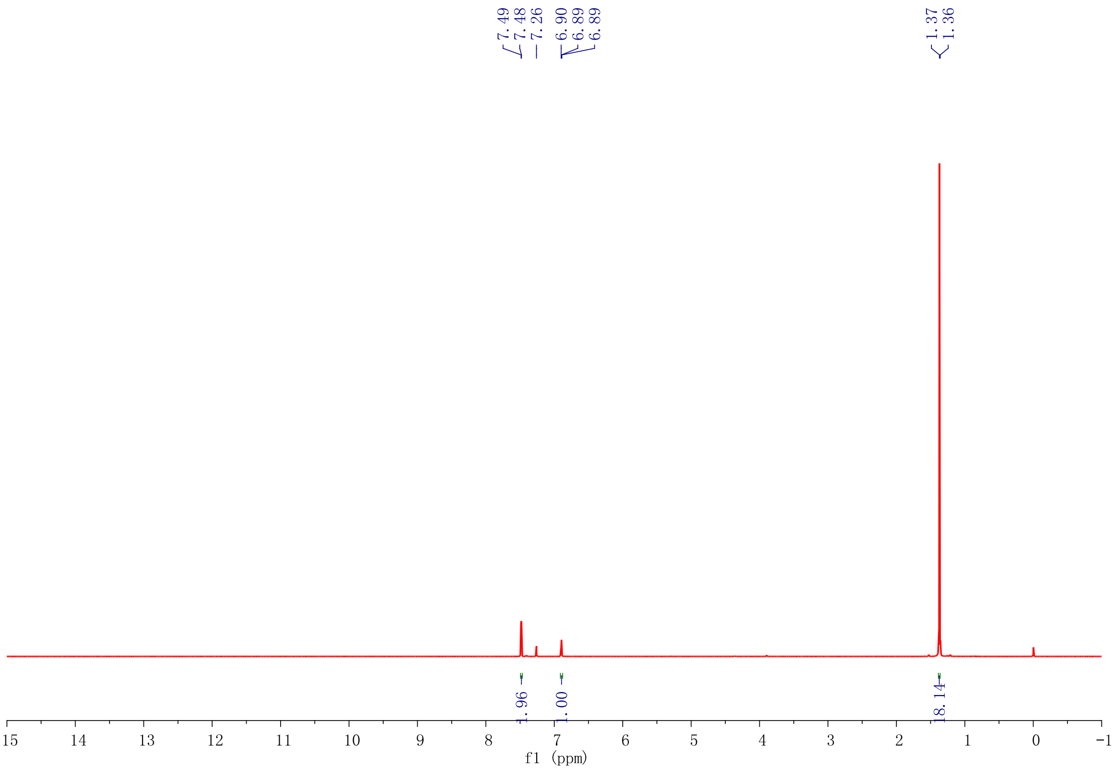

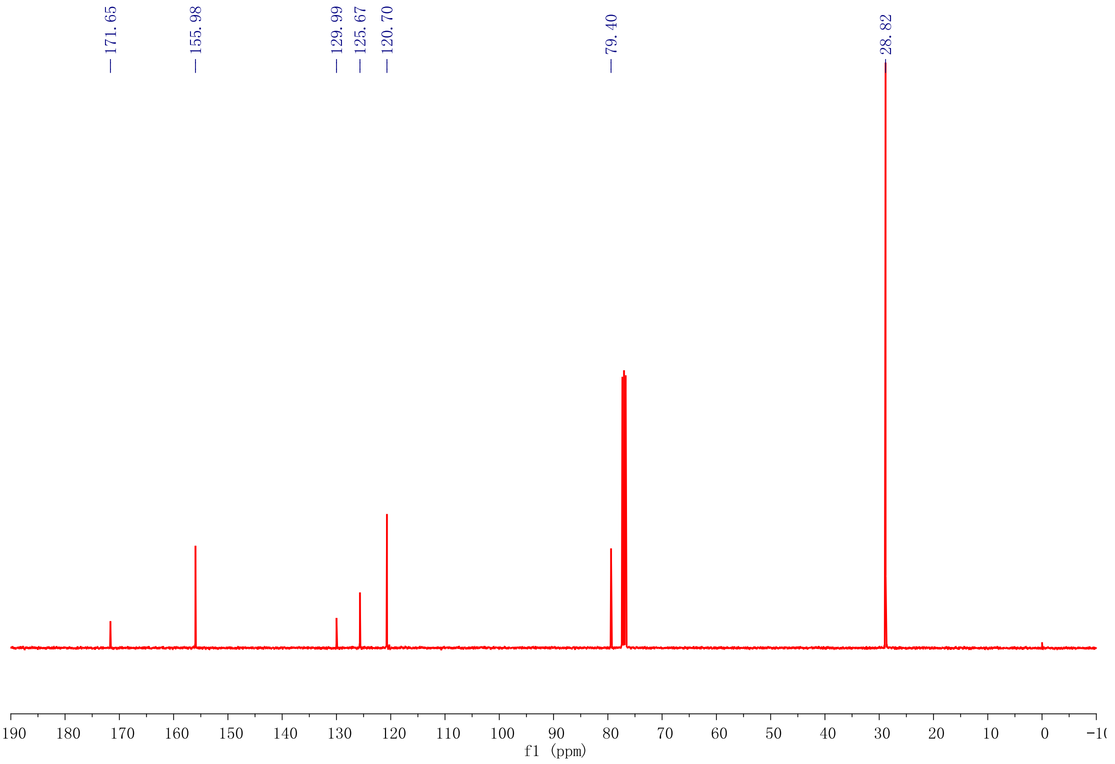

Supplementary Figure 26. ^{1}H, ^{13}C-NMR spectra of product **1z**

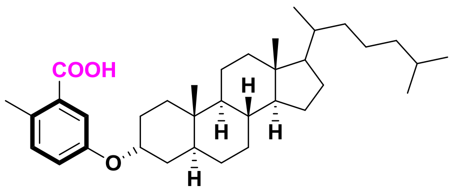

1z

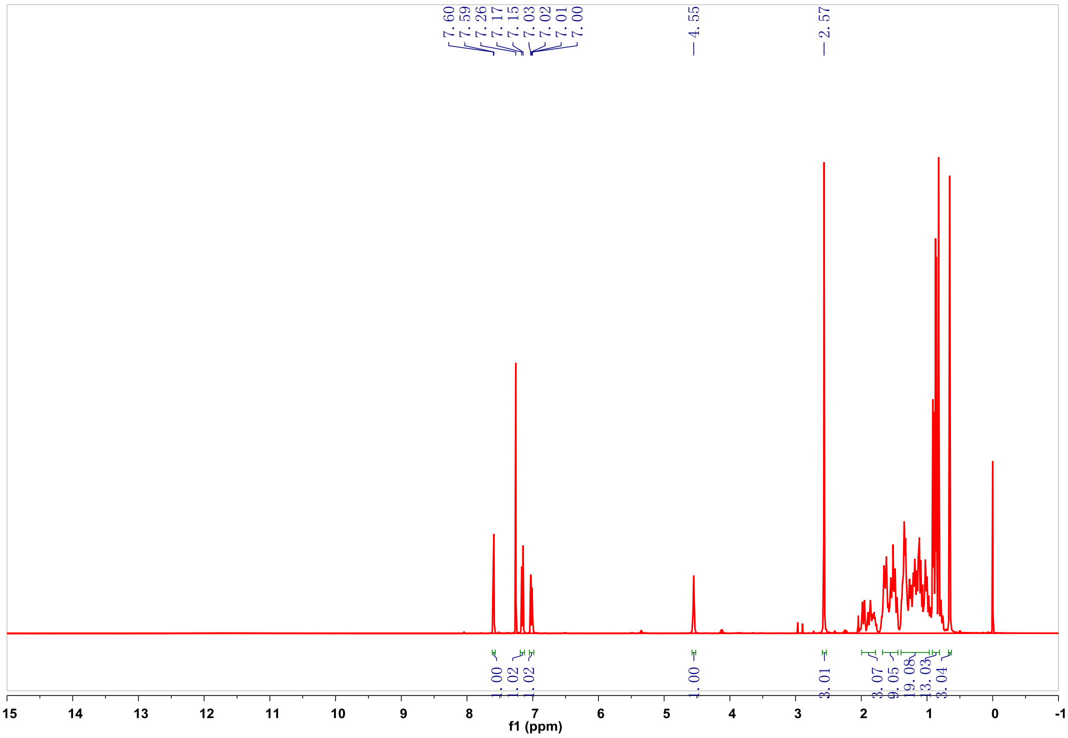

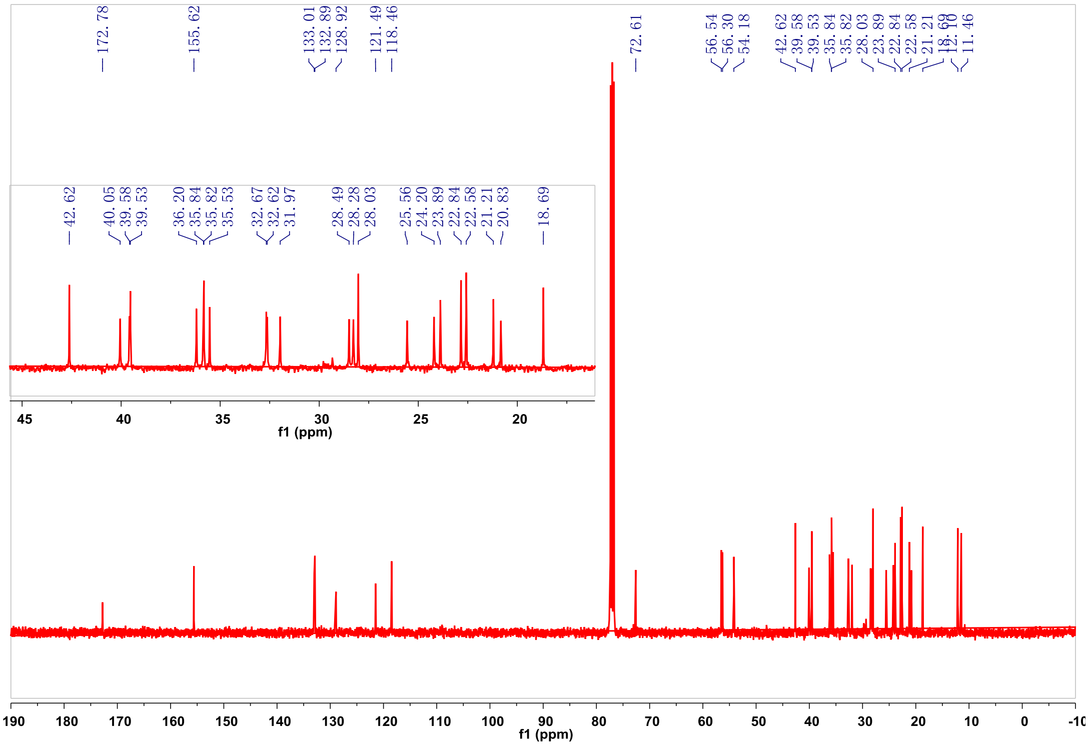

Supplementary Figure 27. ^{1}H, ^{13}C-NMR spectra of product **15a**

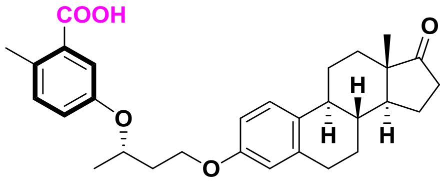

15a

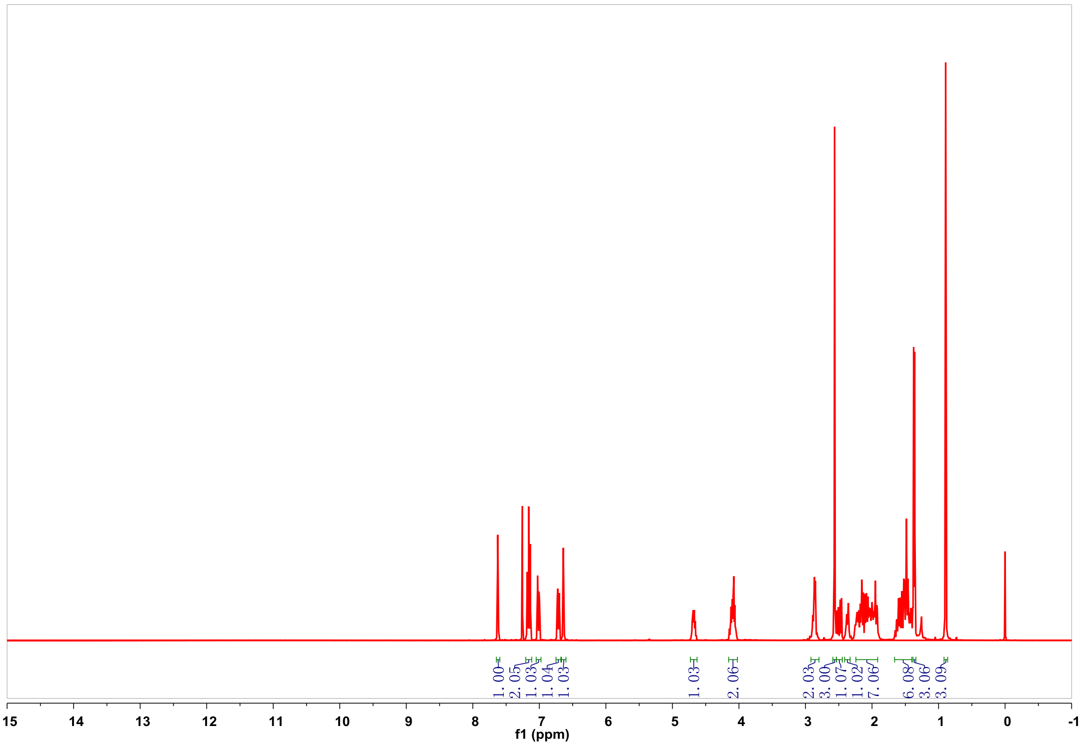

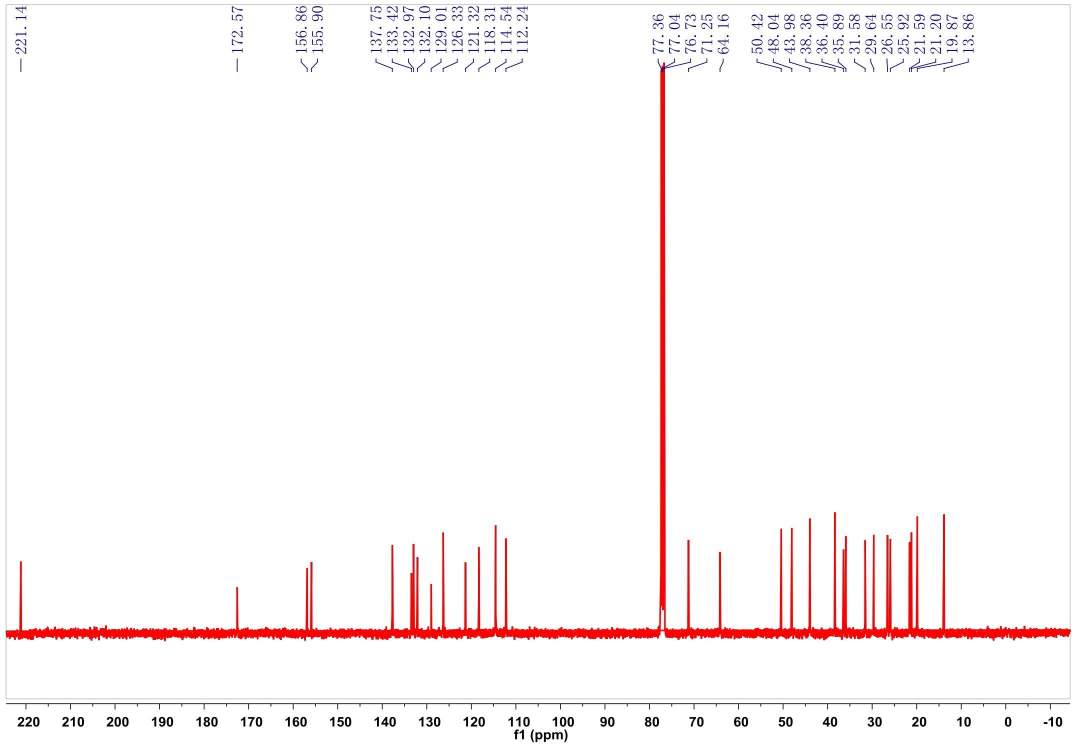

2.NMR Spectra of Corresponding products

Supplementary Figure 28. ^{1}H, ^{13}C-NMR spectra of product **2aa**

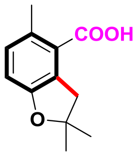

2aa

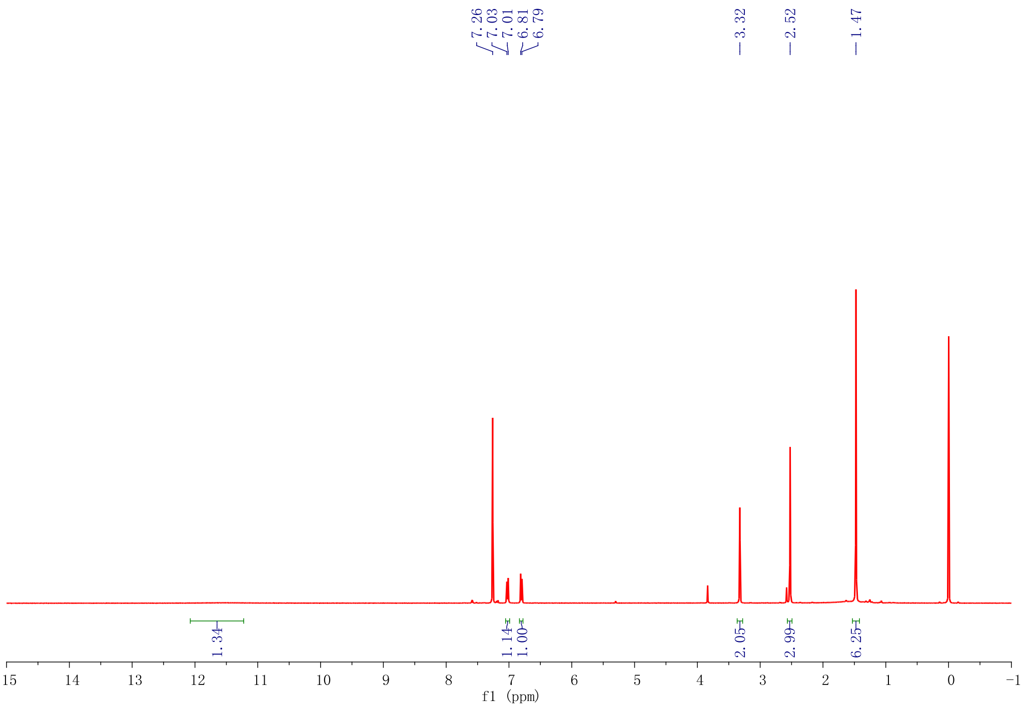

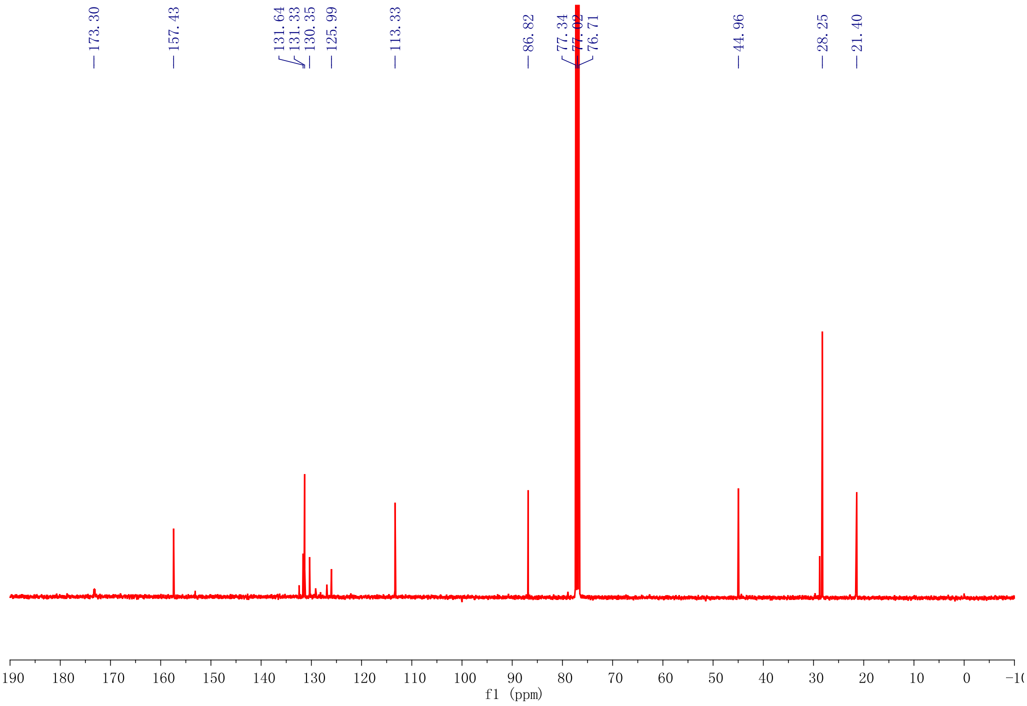

Supplementary Figure 29. ^{1}H, ^{13}C-NMR spectra of product **2a**

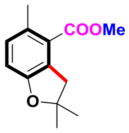

2a

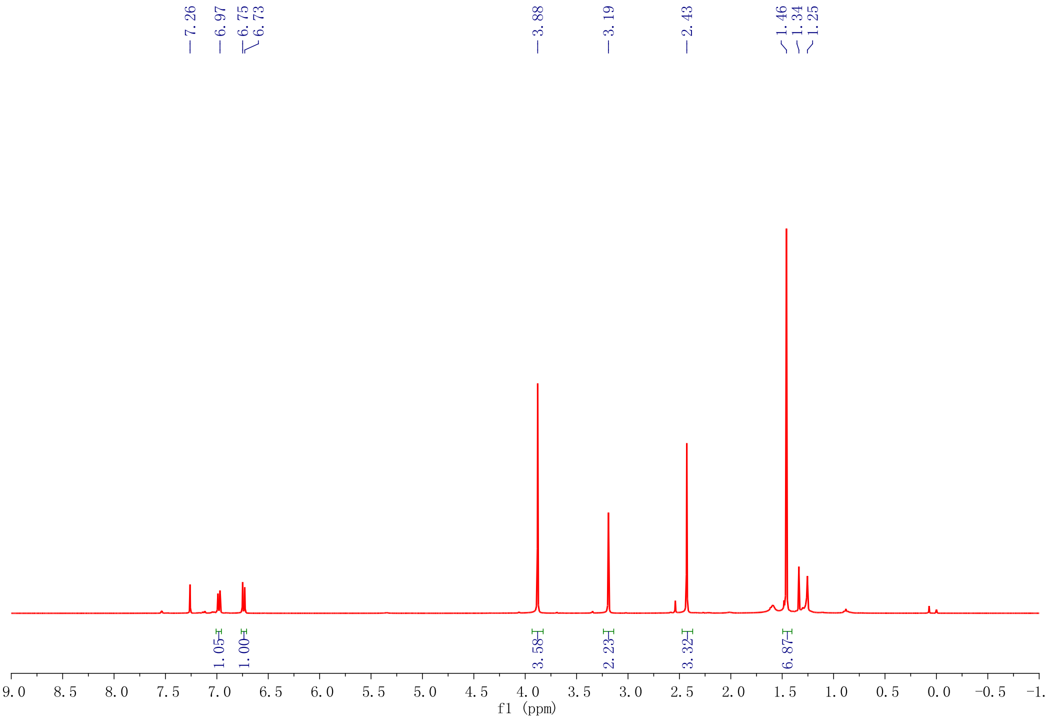

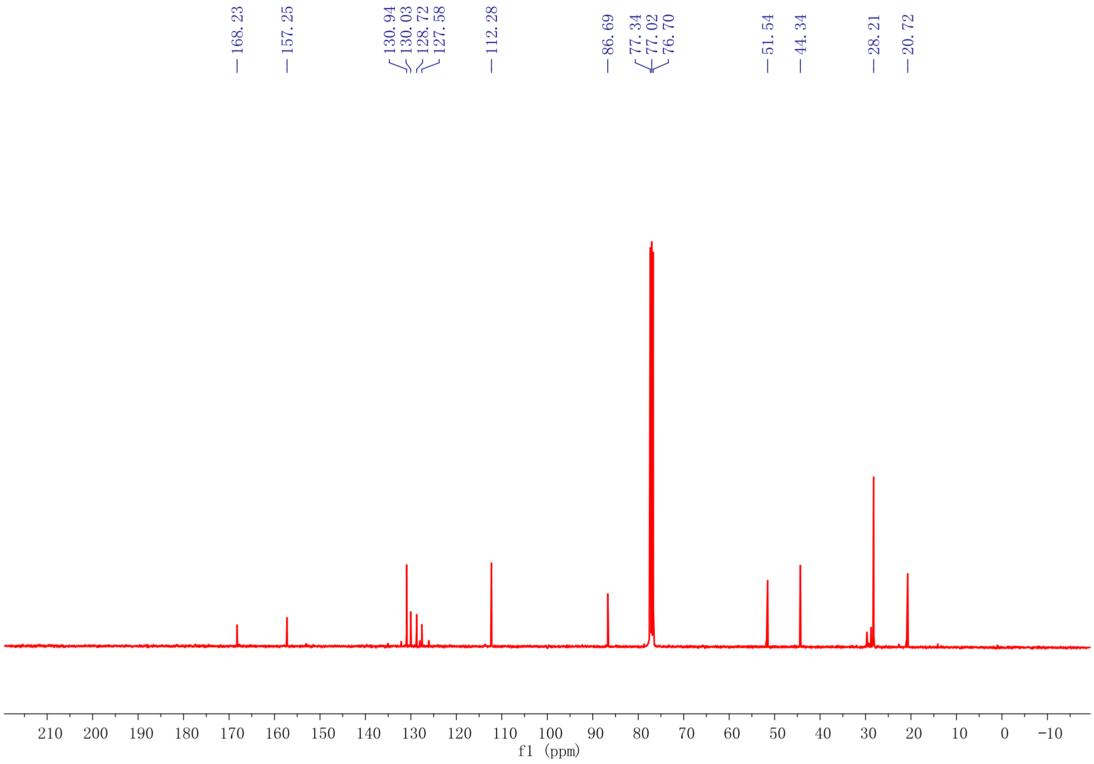

Supplementary Figure 30. ^{1}H, ^{13}C-NMR spectra of product **2b**

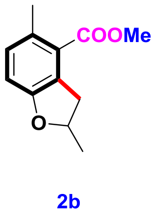

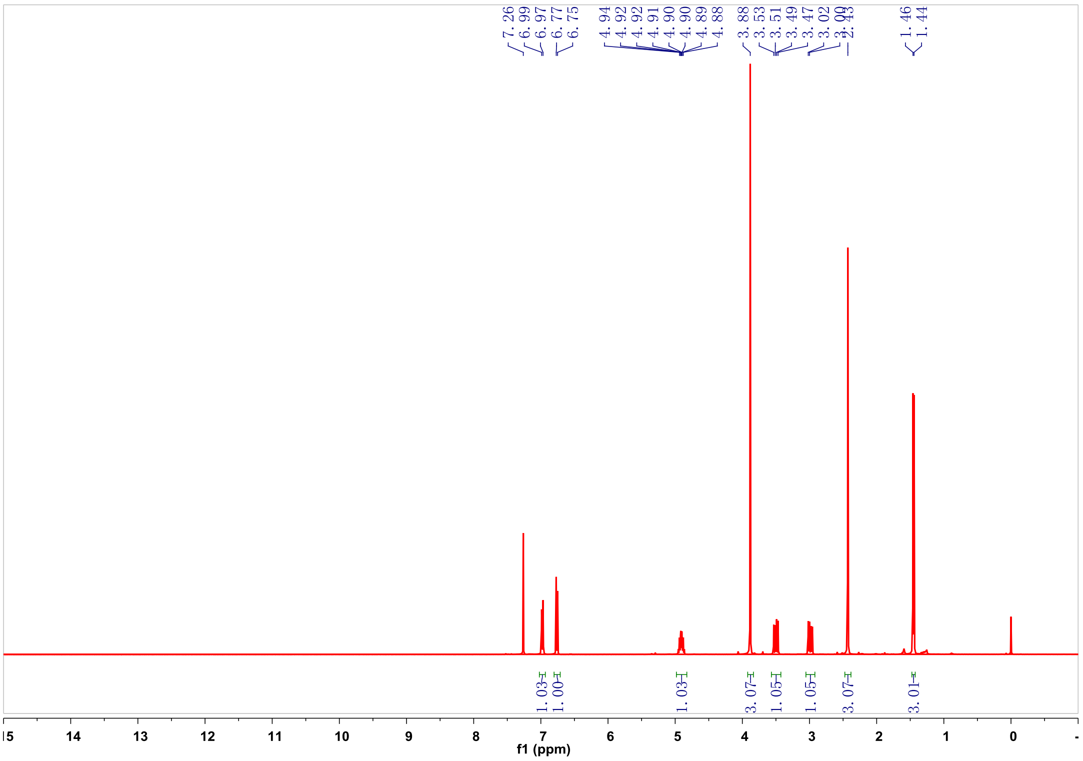

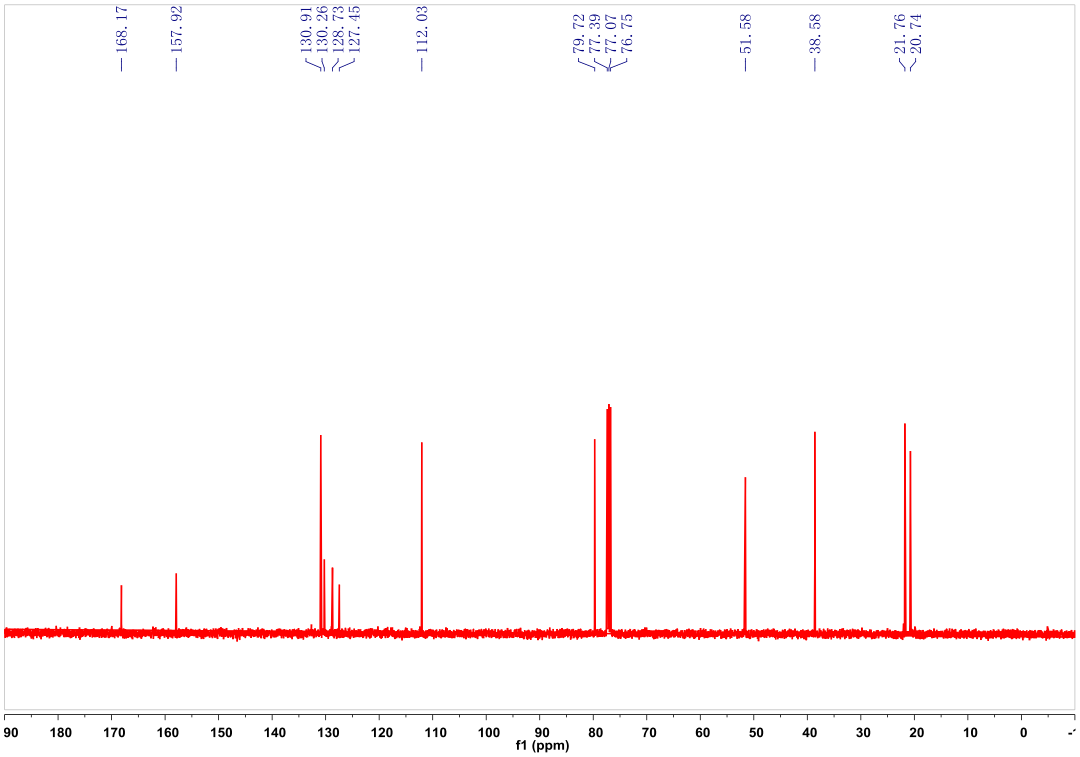

Supplementary Figure 31. ^{1}H, ^{13}C-NMR spectra of product **2c**

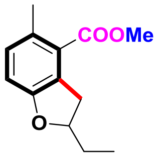

2c

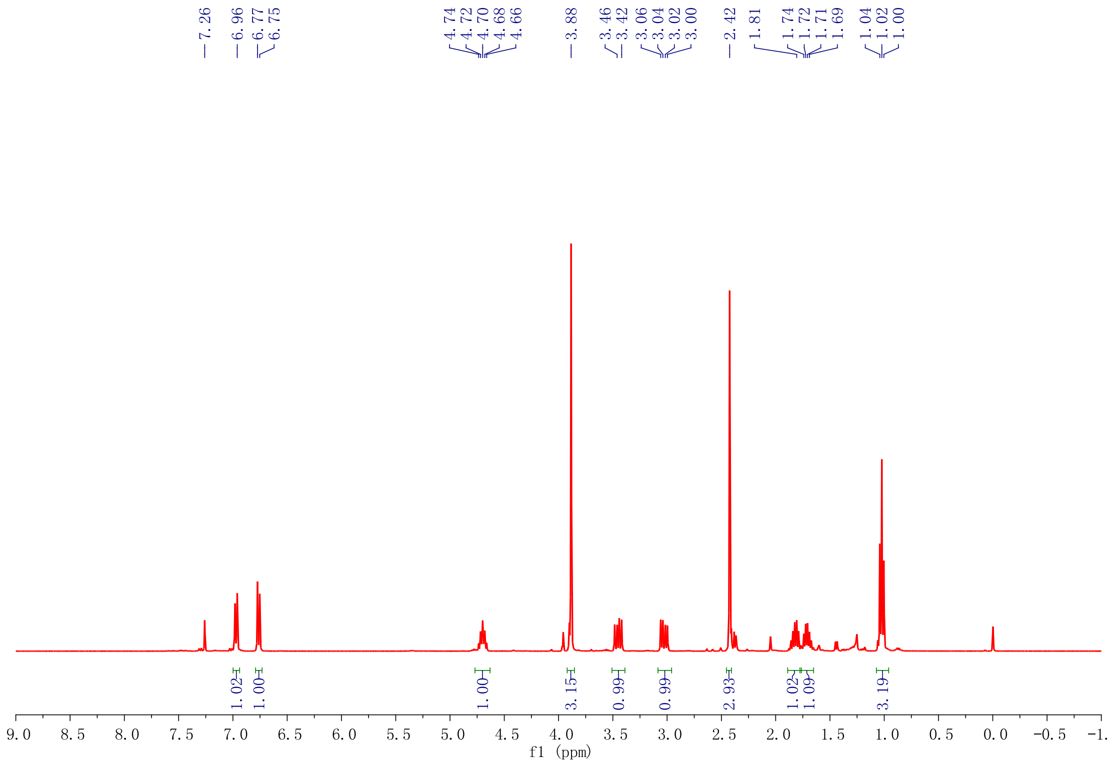

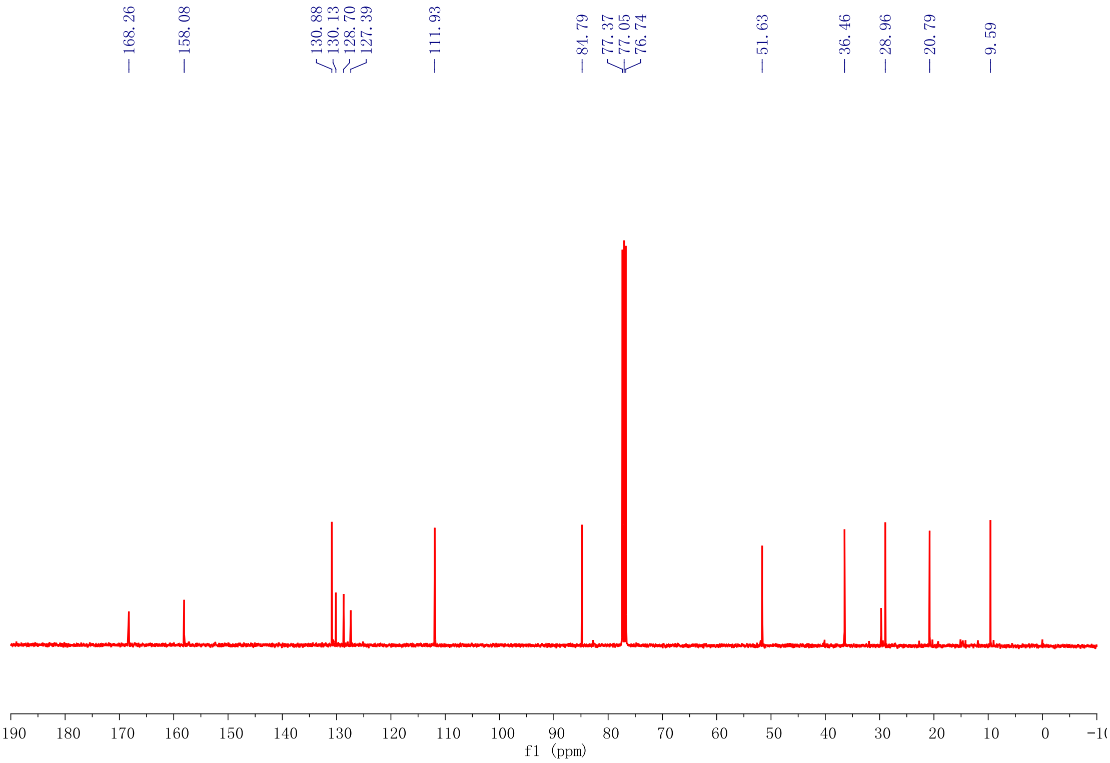

Supplementary Figure 32. ^{1}H, ^{13}C-NMR spectra of product **2d**

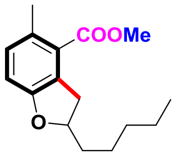

2d

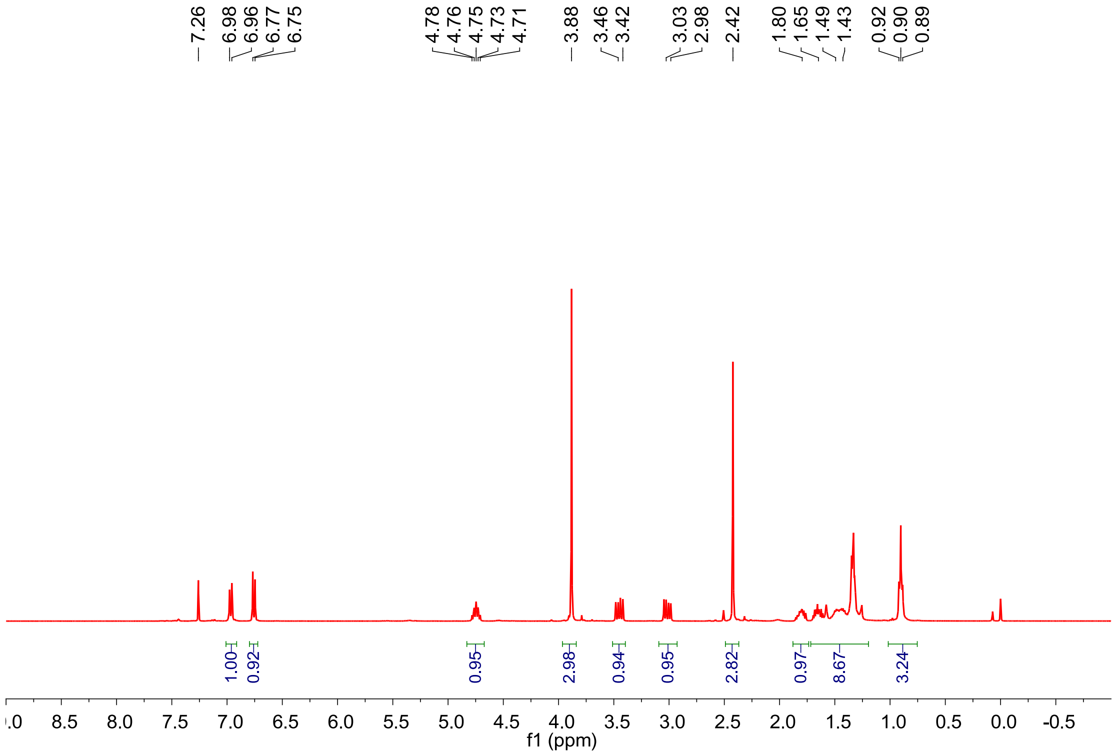

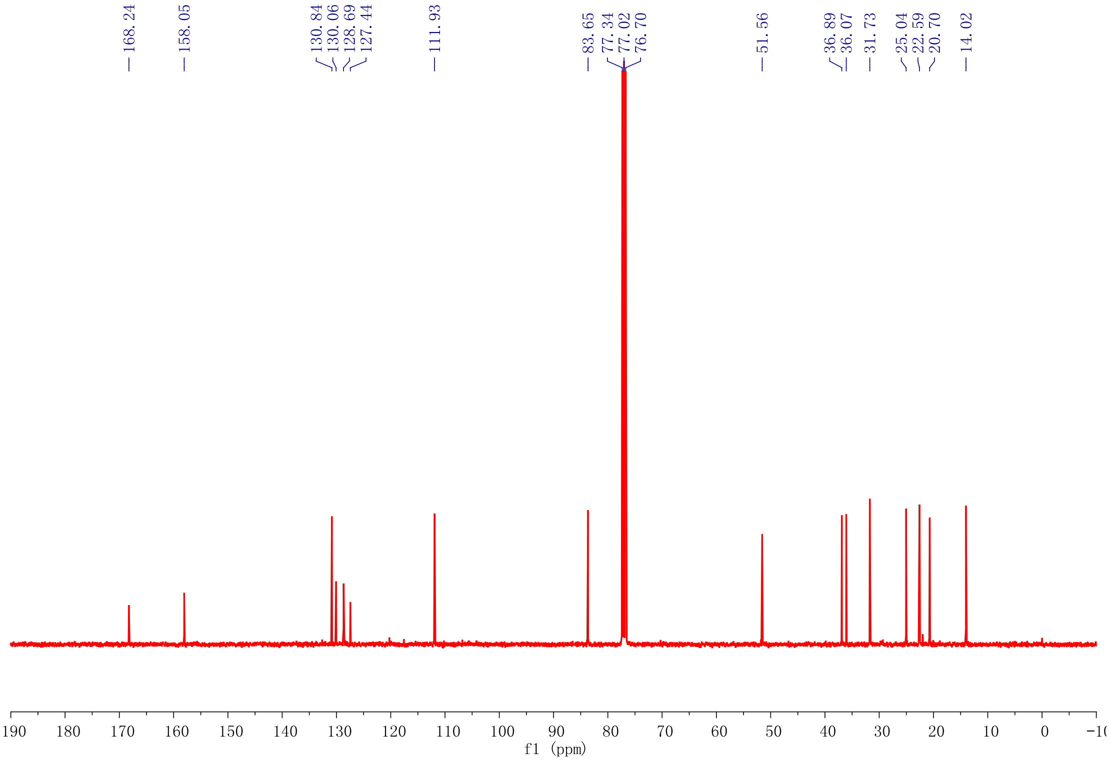

Supplementary Figure 33. ^{1}H, ^{13}C-NMR spectra of product **2e**

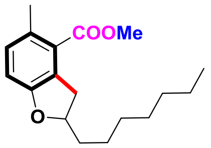

2e

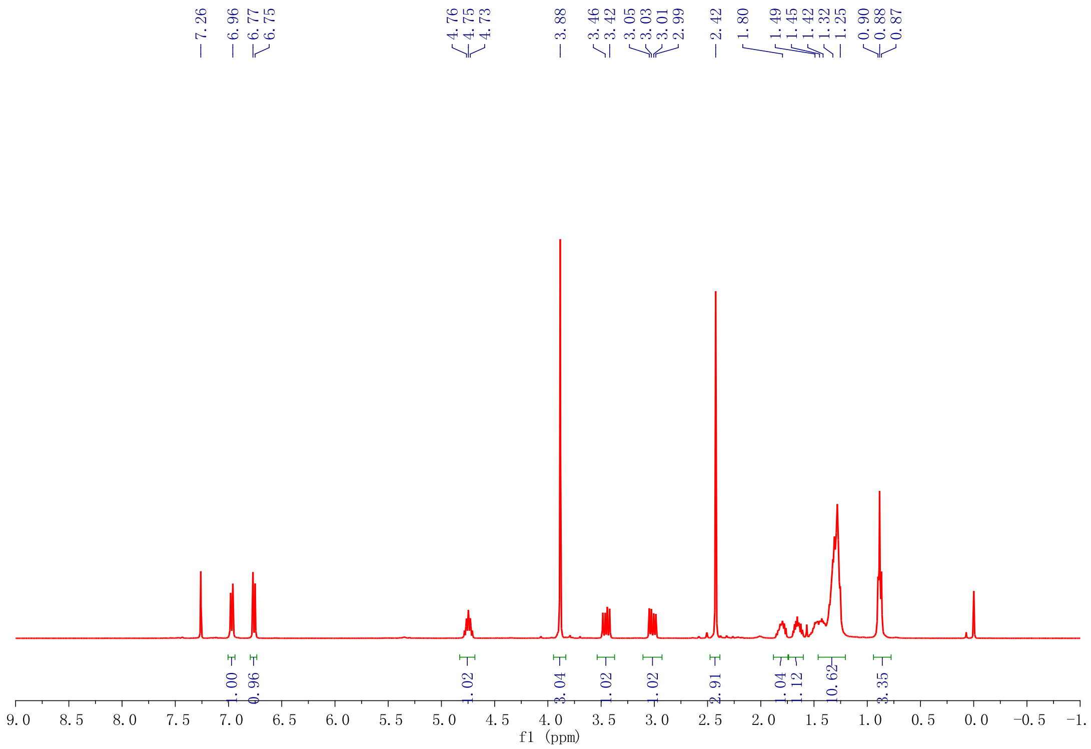

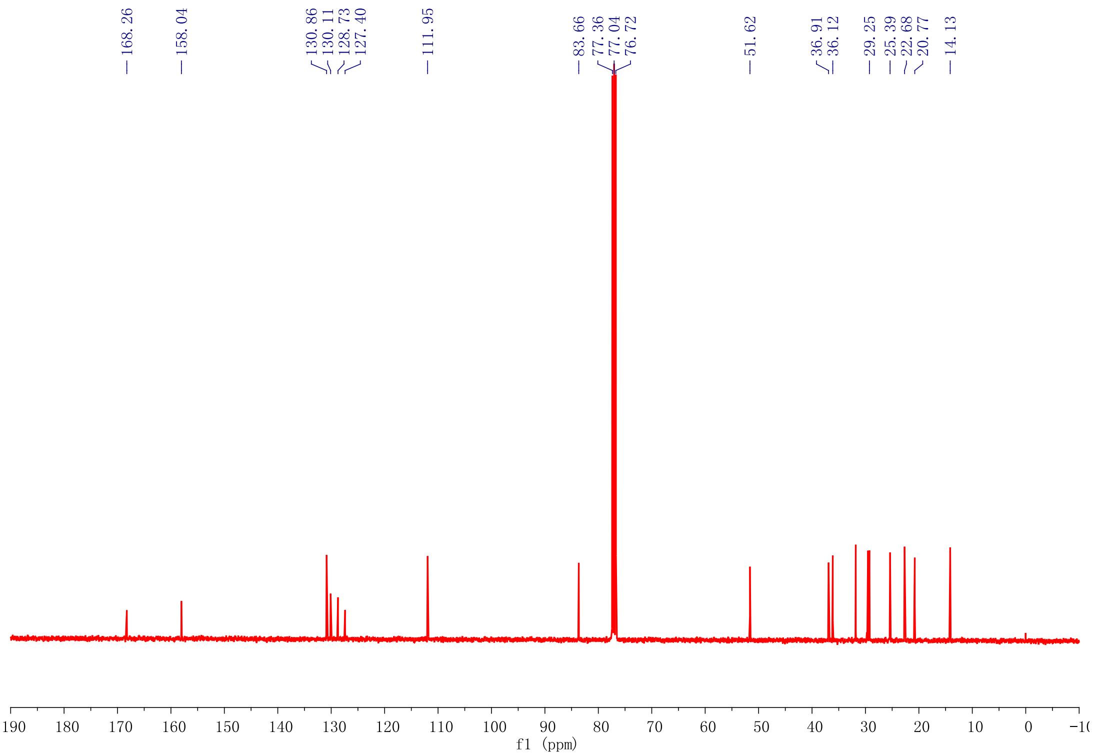

Supplementary Figure 34. ^{1}H, ^{13}C-NMR spectra of product **2f**

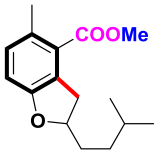

2f

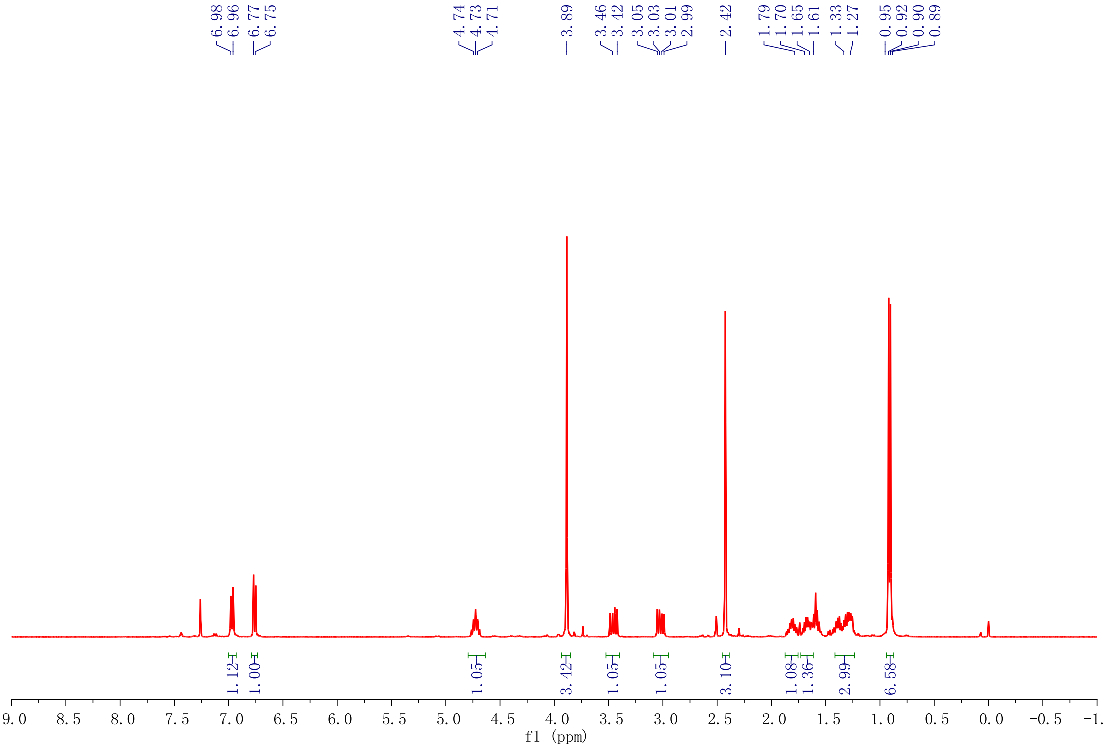

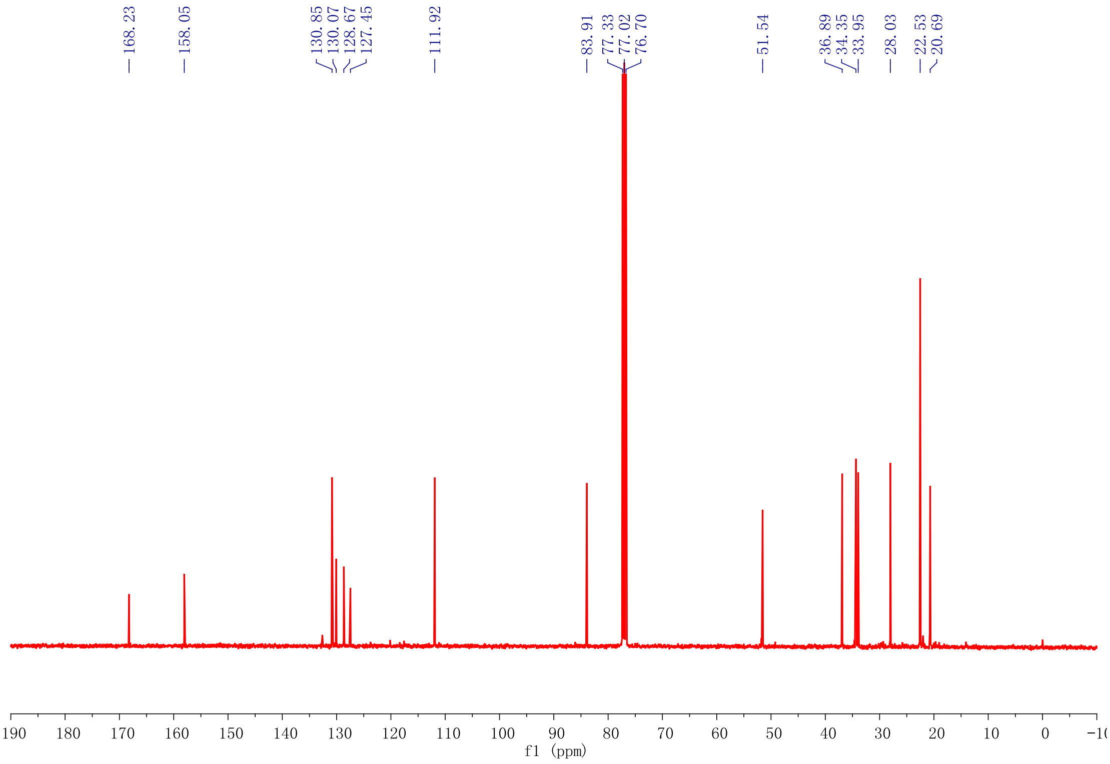

Supplementary Figure 35. ^{1}H, ^{13}C-NMR spectra of product **2g**

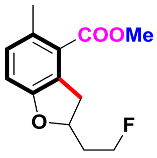

2g

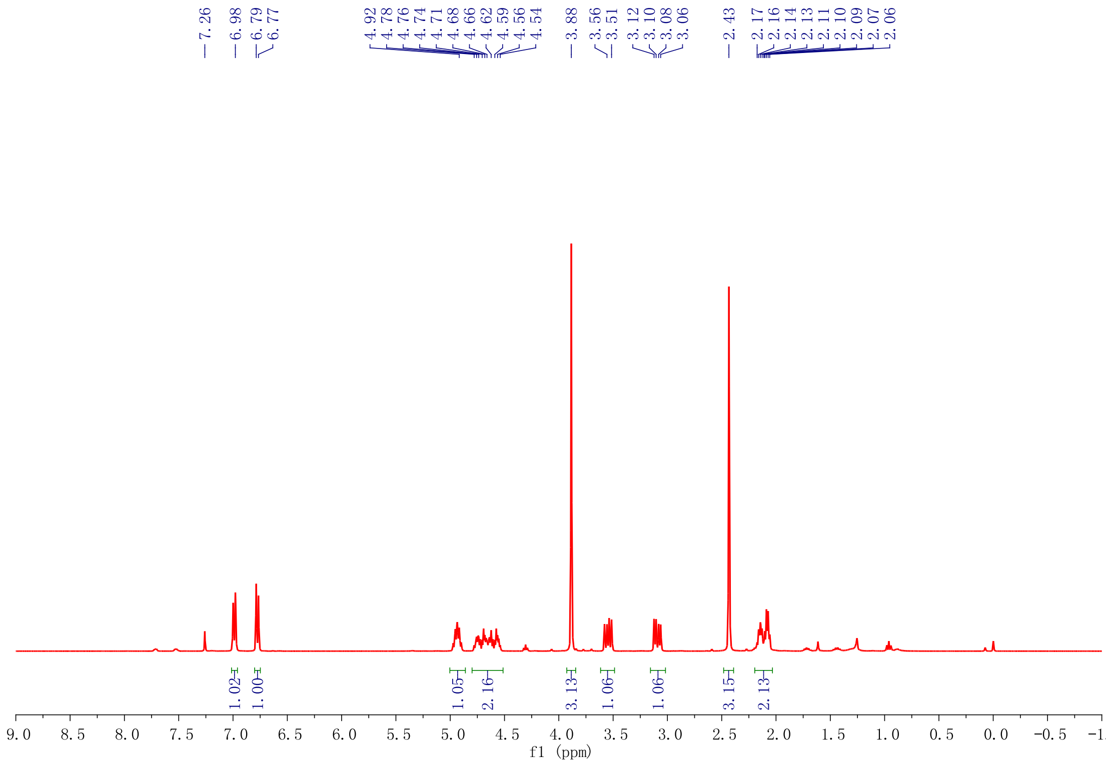

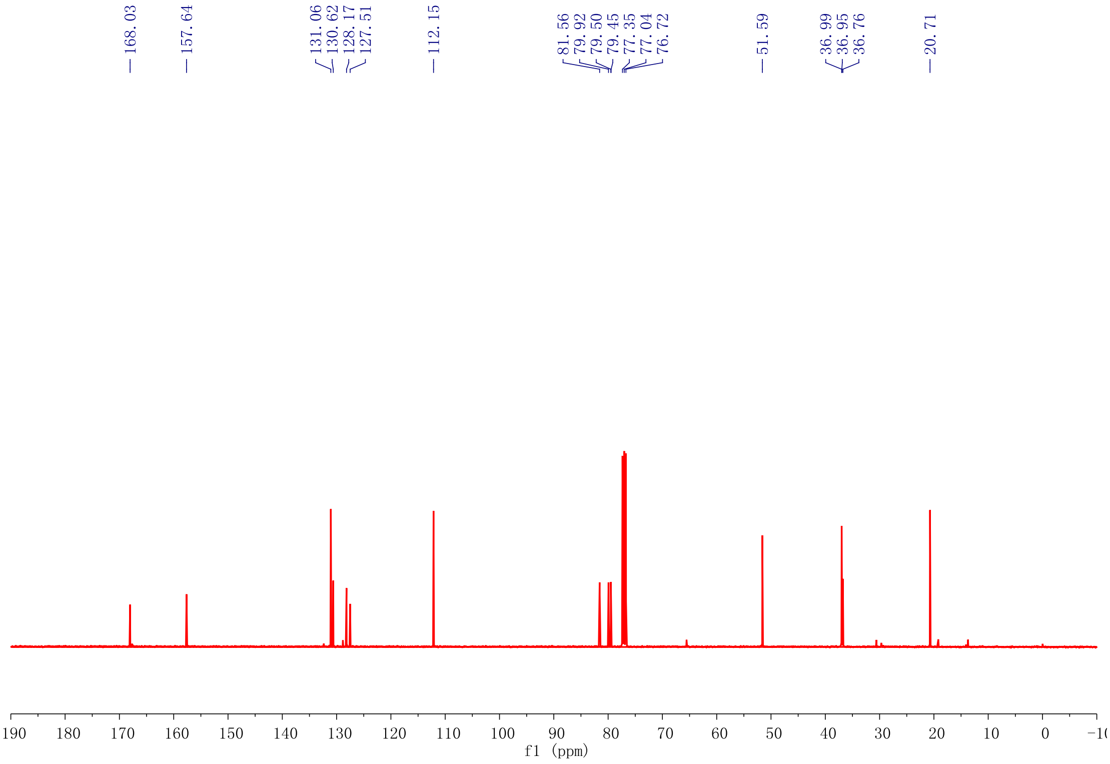

Supplementary Figure 36. ^{1}H, ^{13}C-NMR spectra of product **2h**

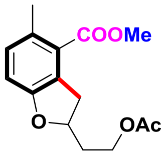

2h

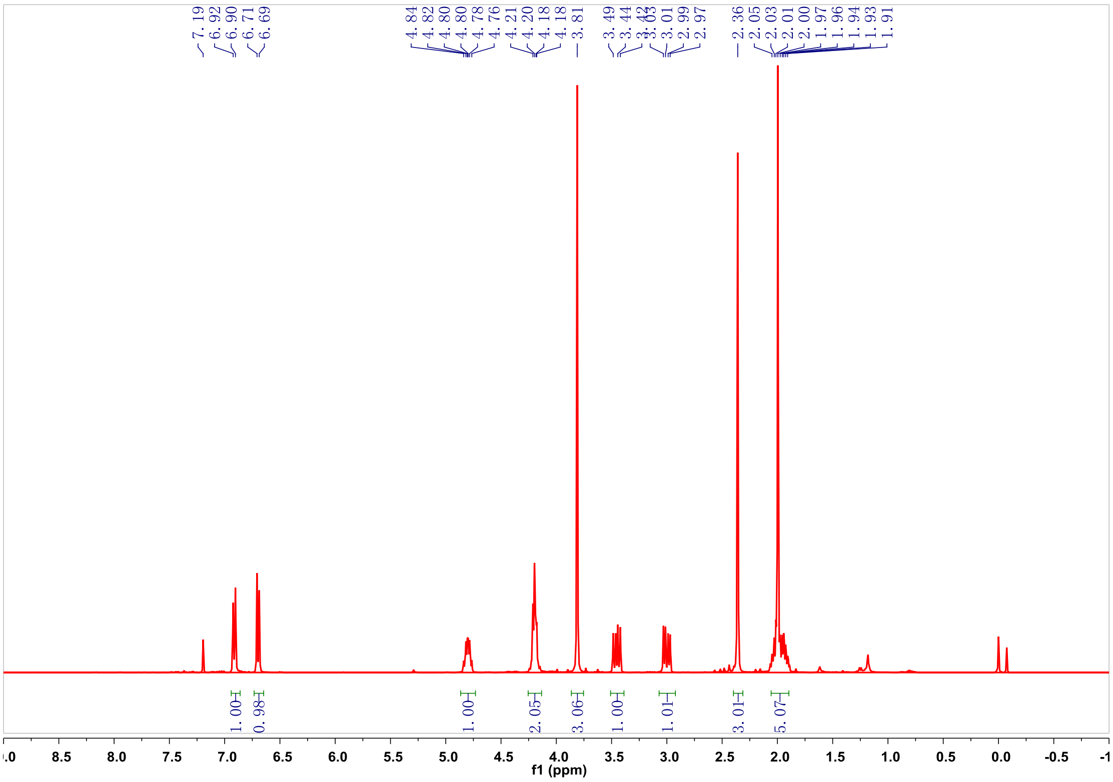

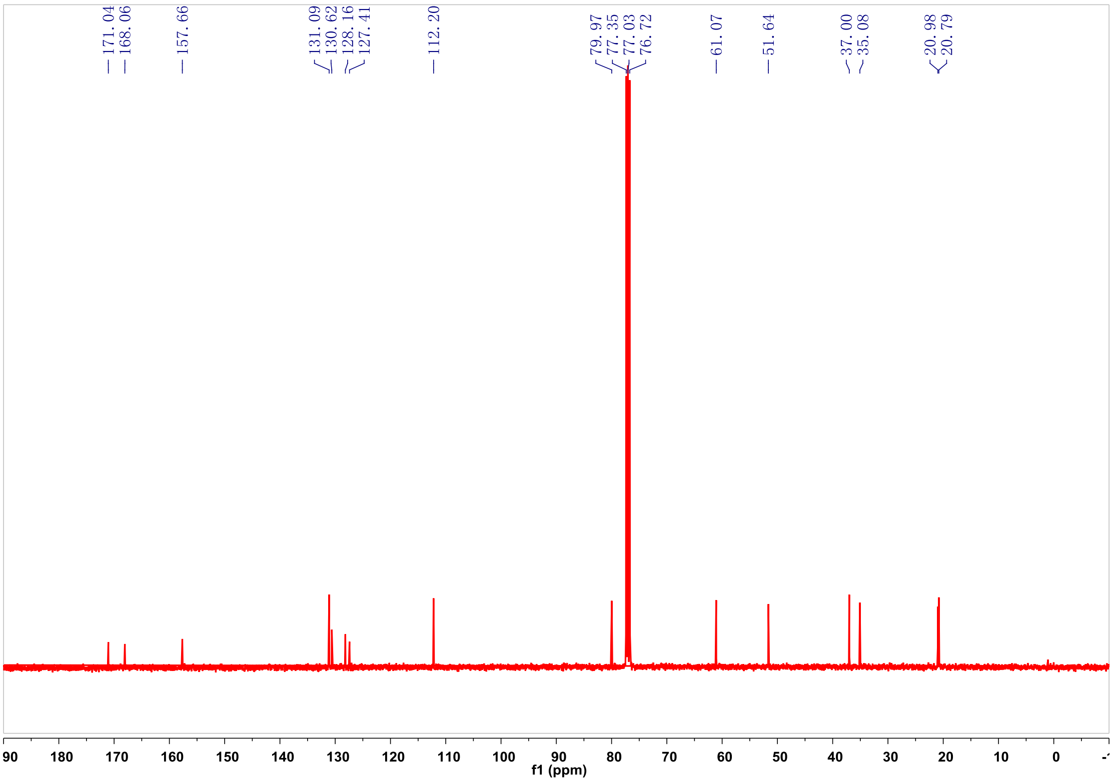

Supplementary Figure 37. ^{1}H, ^{13}C-NMR spectra of product **2i**

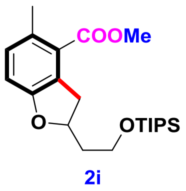

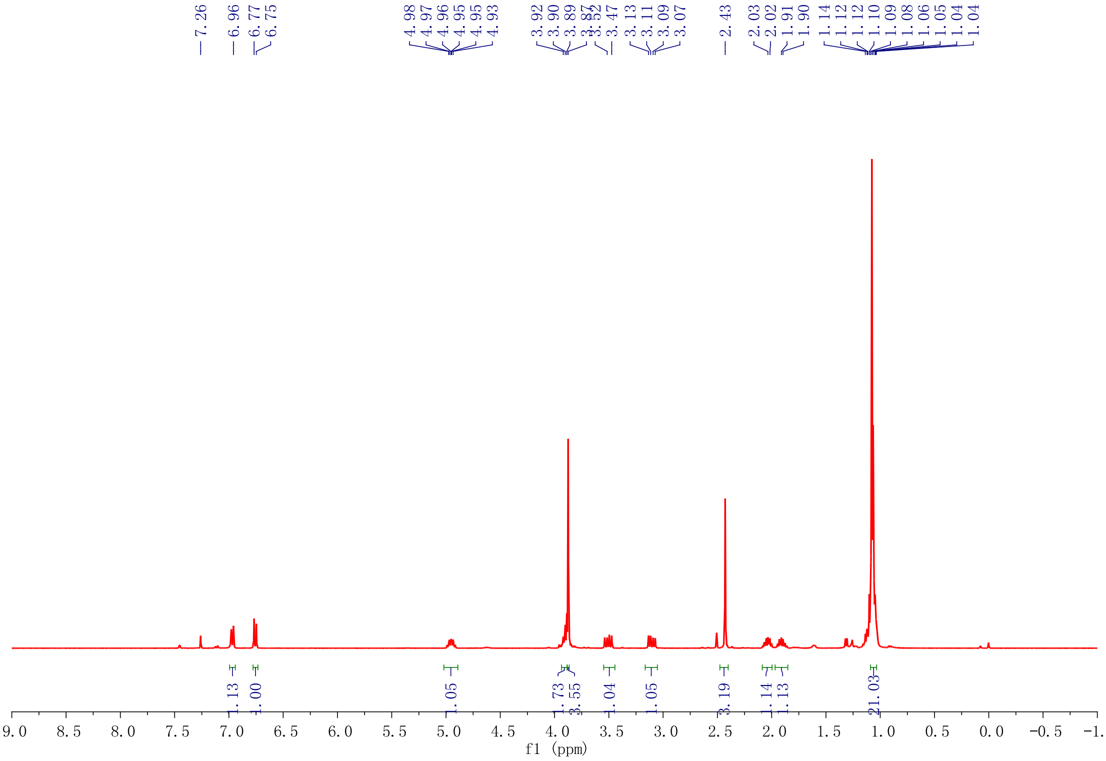

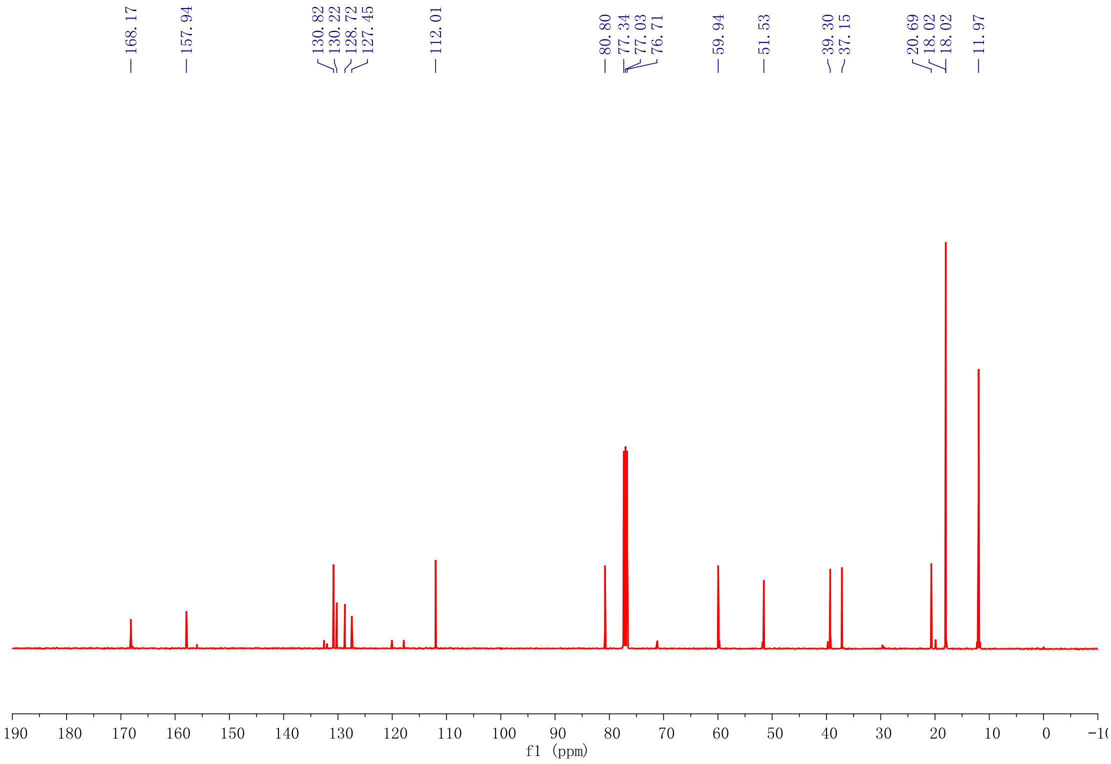

Supplementary Figure 38. ^{1}H, ^{13}C-NMR spectra of product **2j**

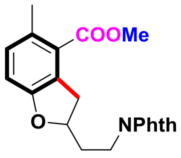

2j

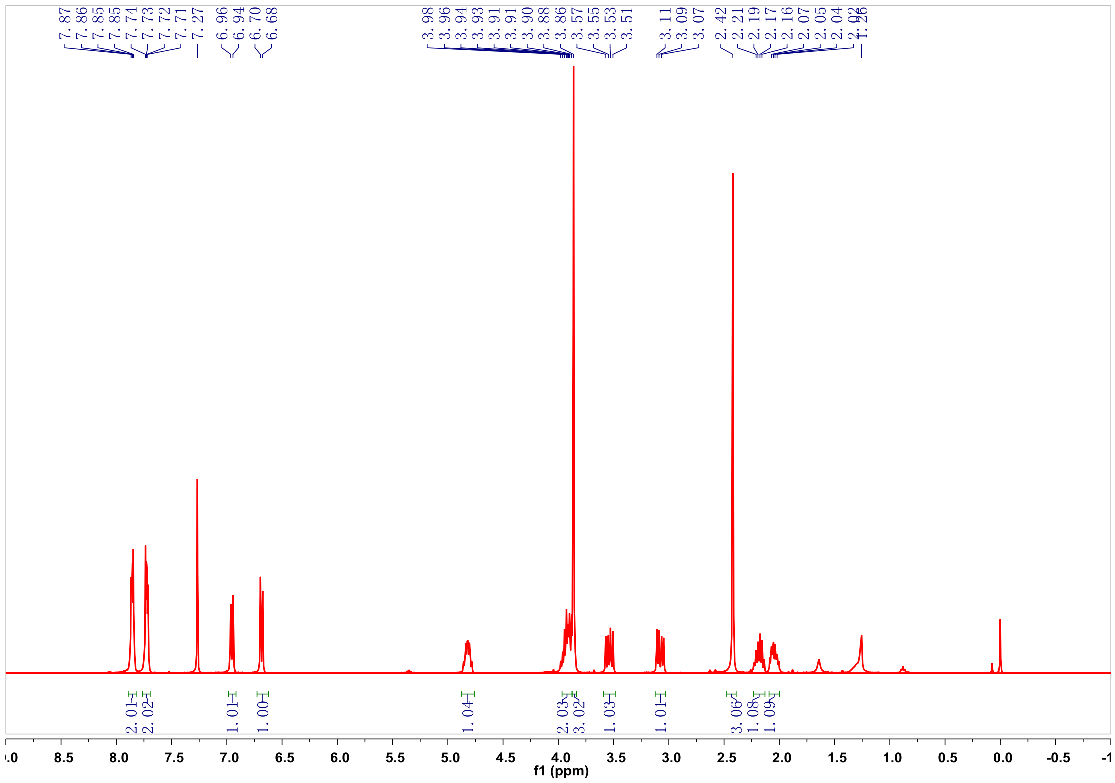

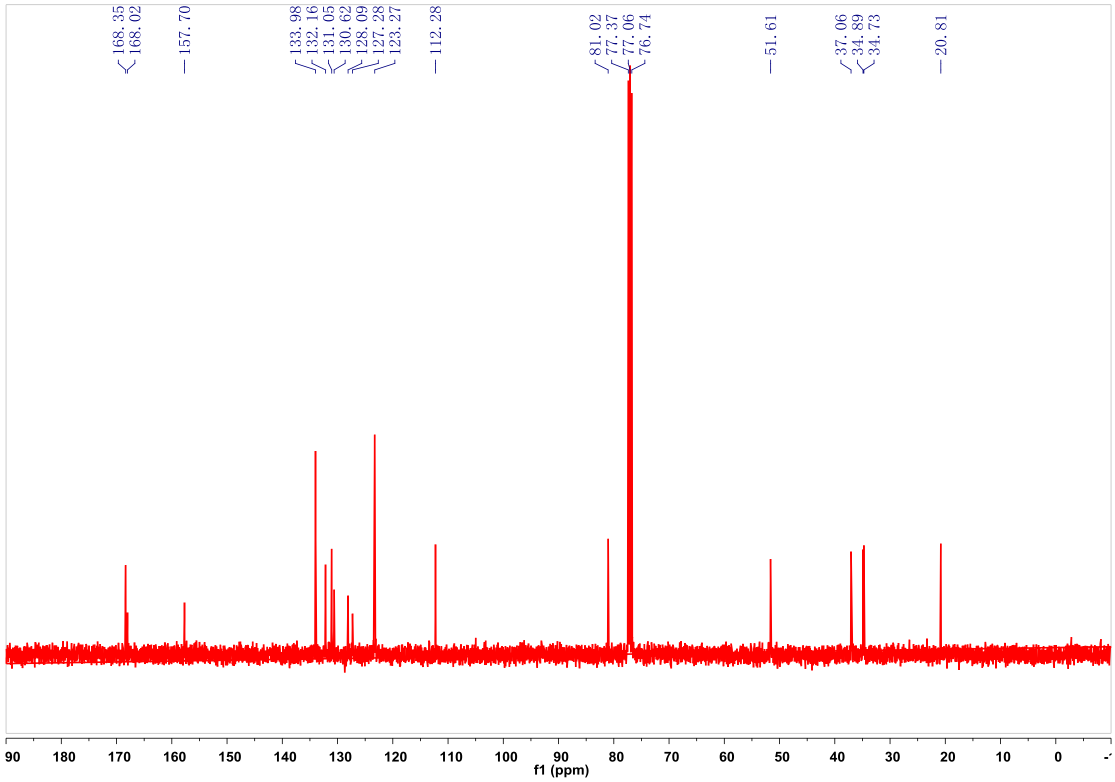

Supplementary Figure 39. ^{1}H, ^{13}C-NMR spectra of product **2k**

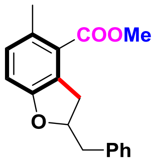

2k

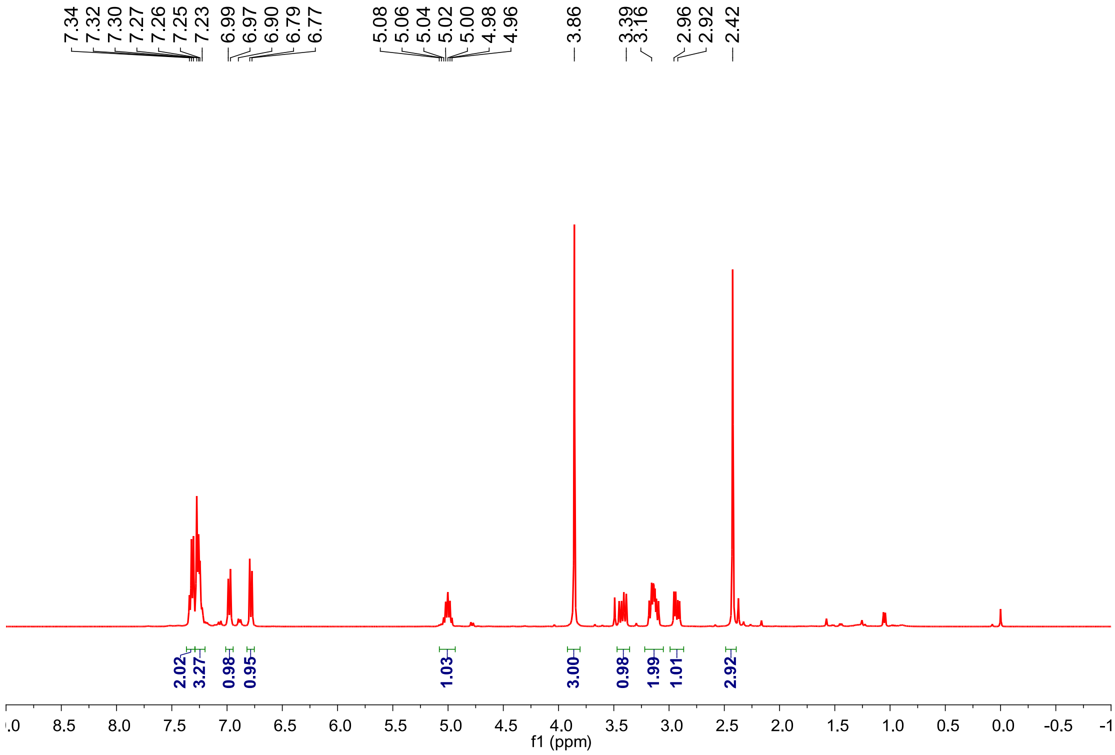

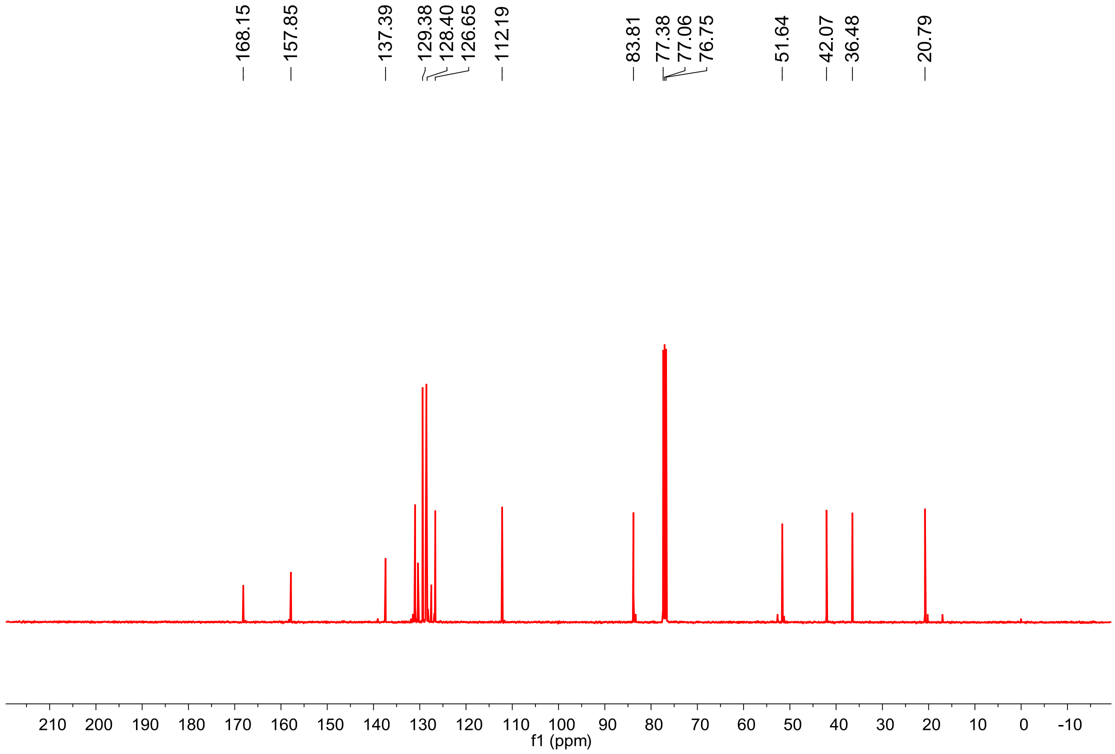

Supplementary Figure 40. ^{1}H, ^{13}C-NMR spectra of product **2l**

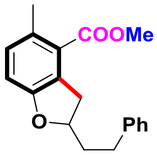

2l

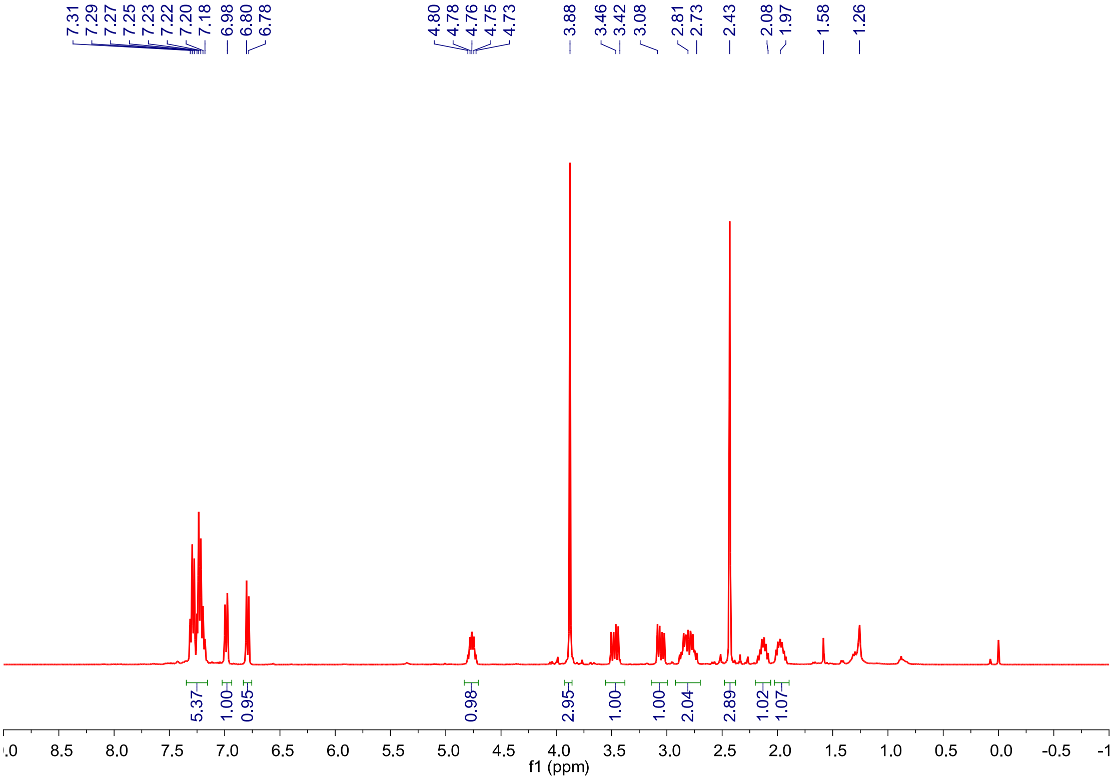

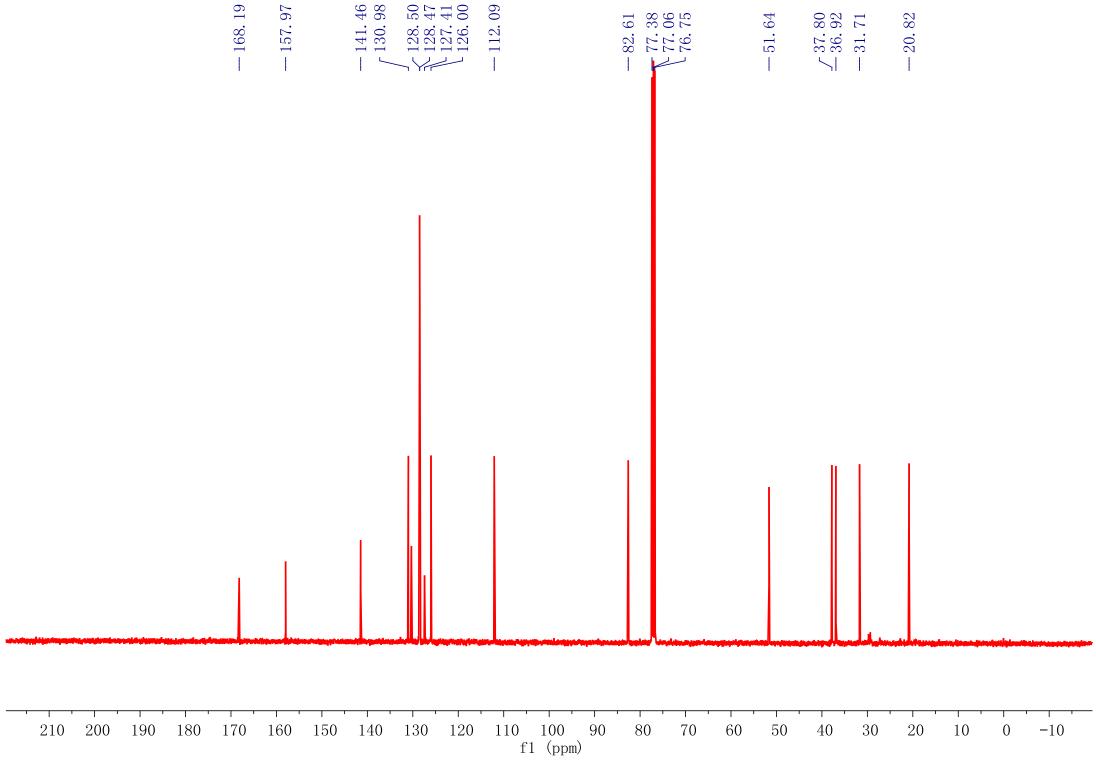

Supplementary Figure 41. ^{1}H, ^{13}C-NMR spectra of product **2m**

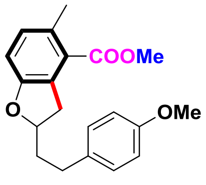

2m

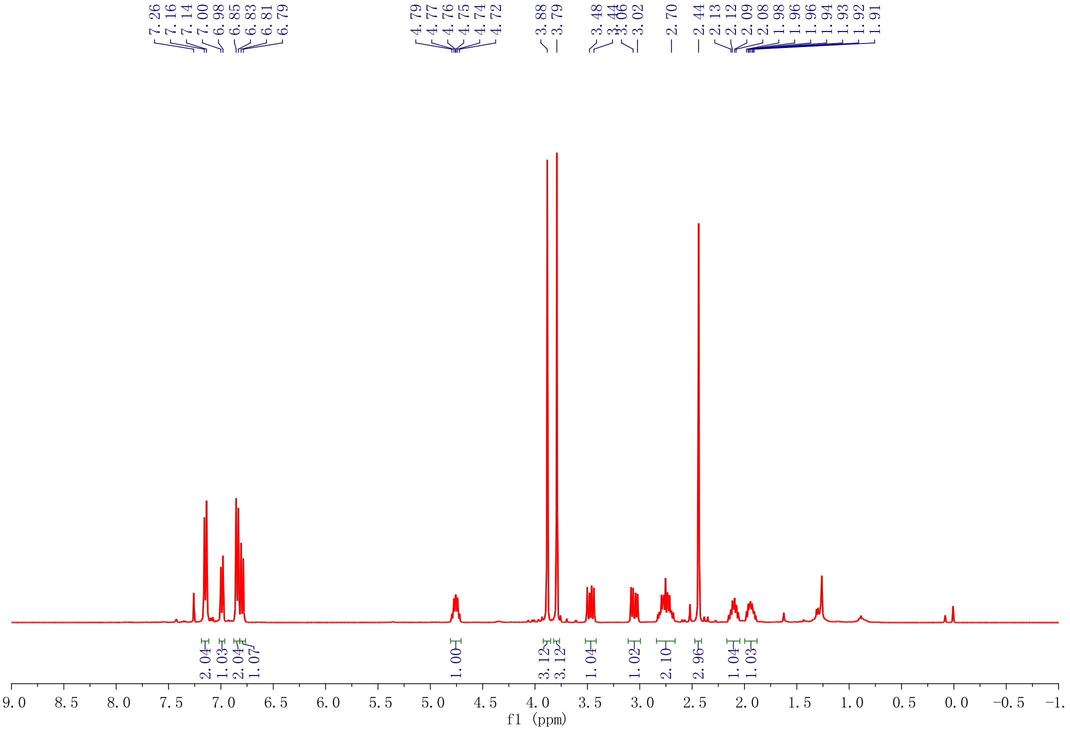

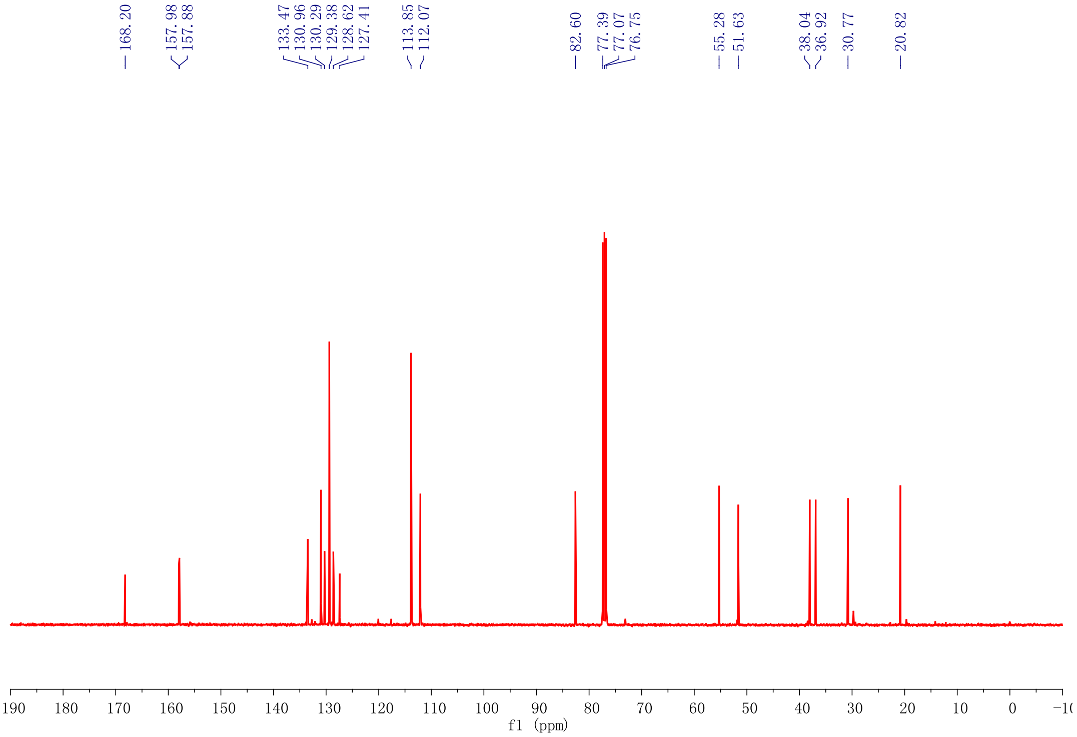

Supplementary Figure 42. ^{1}H, ^{13}C-NMR spectra of product **2ma**

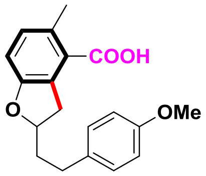

2ma

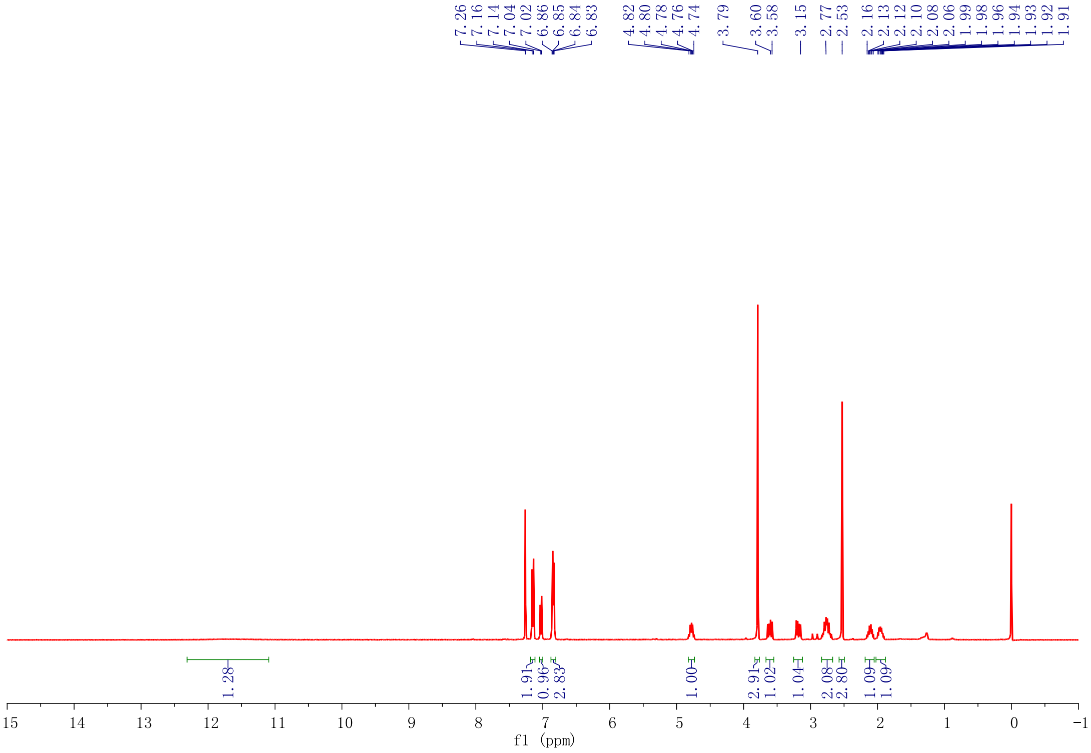

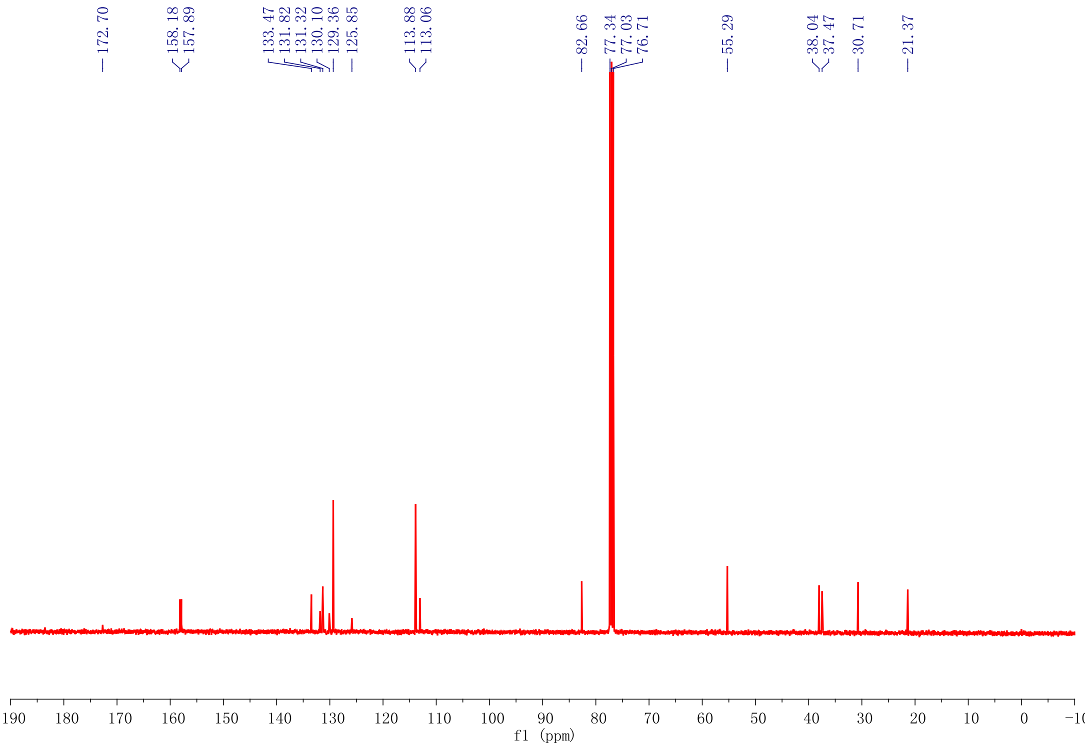

Supplementary Figure 43. ^{1}H, ^{13}C-NMR spectra of product **2n**

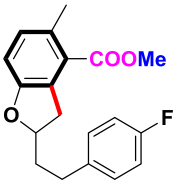

2n

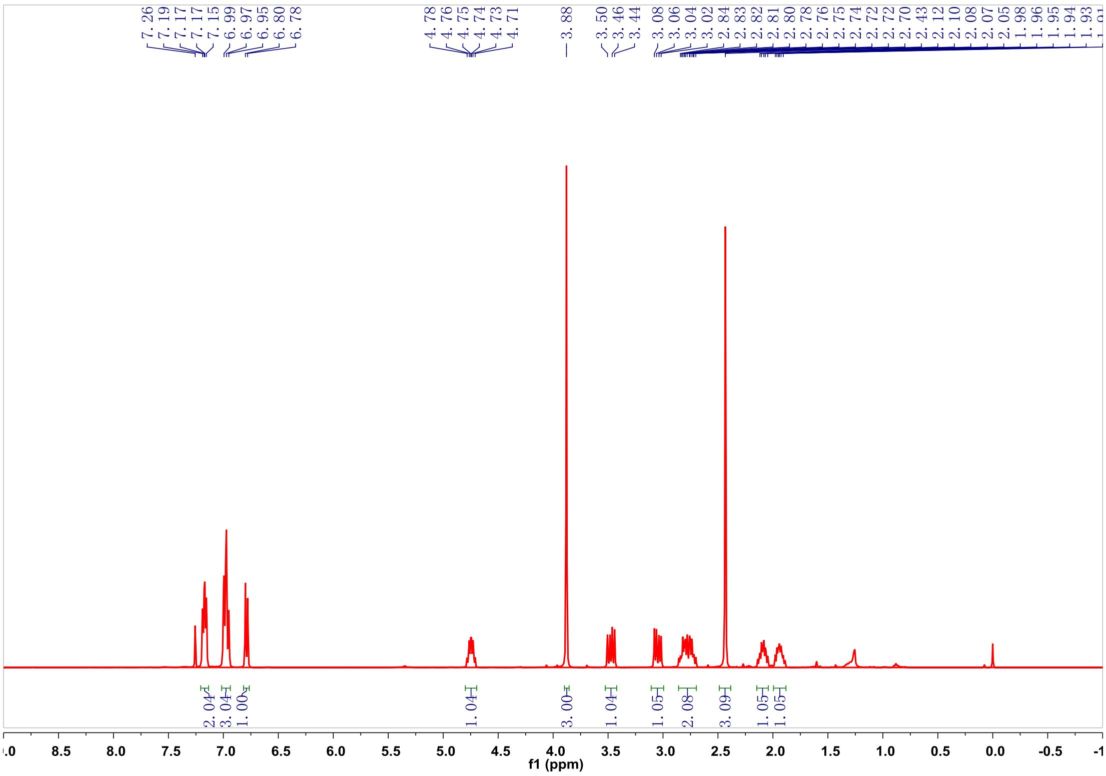

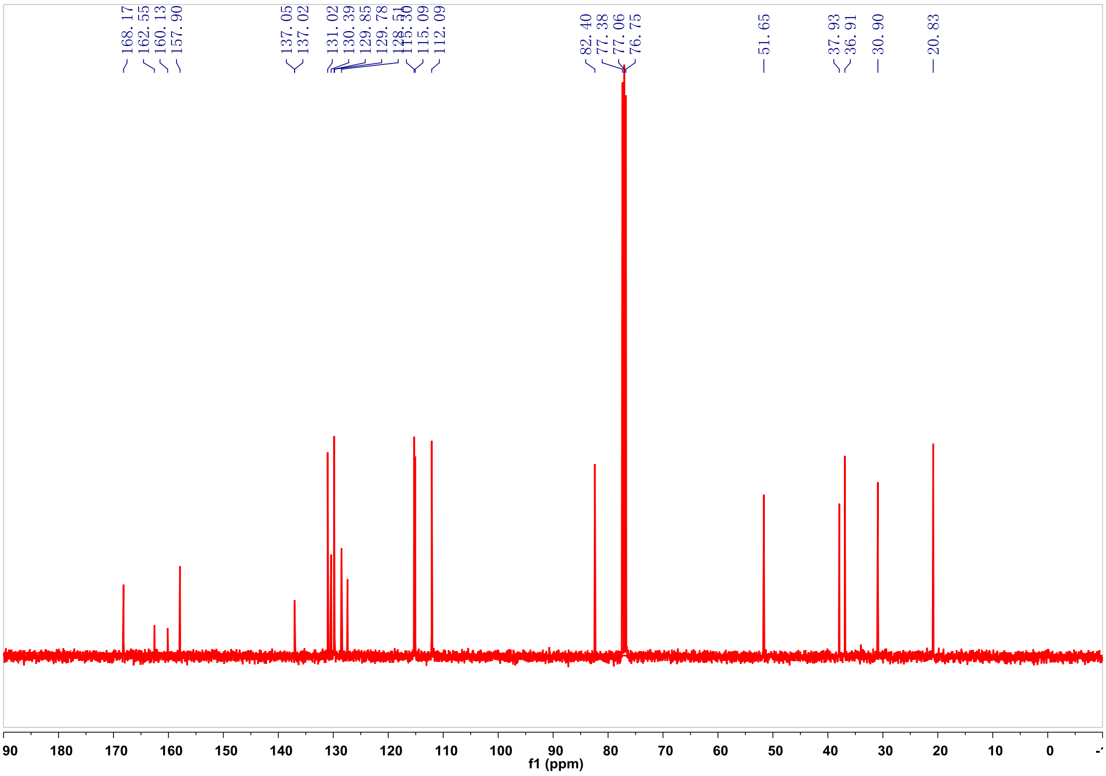

Supplementary Figure 44. ^{1}H, ^{13}C-NMR spectra of product **2o**

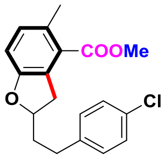

2o

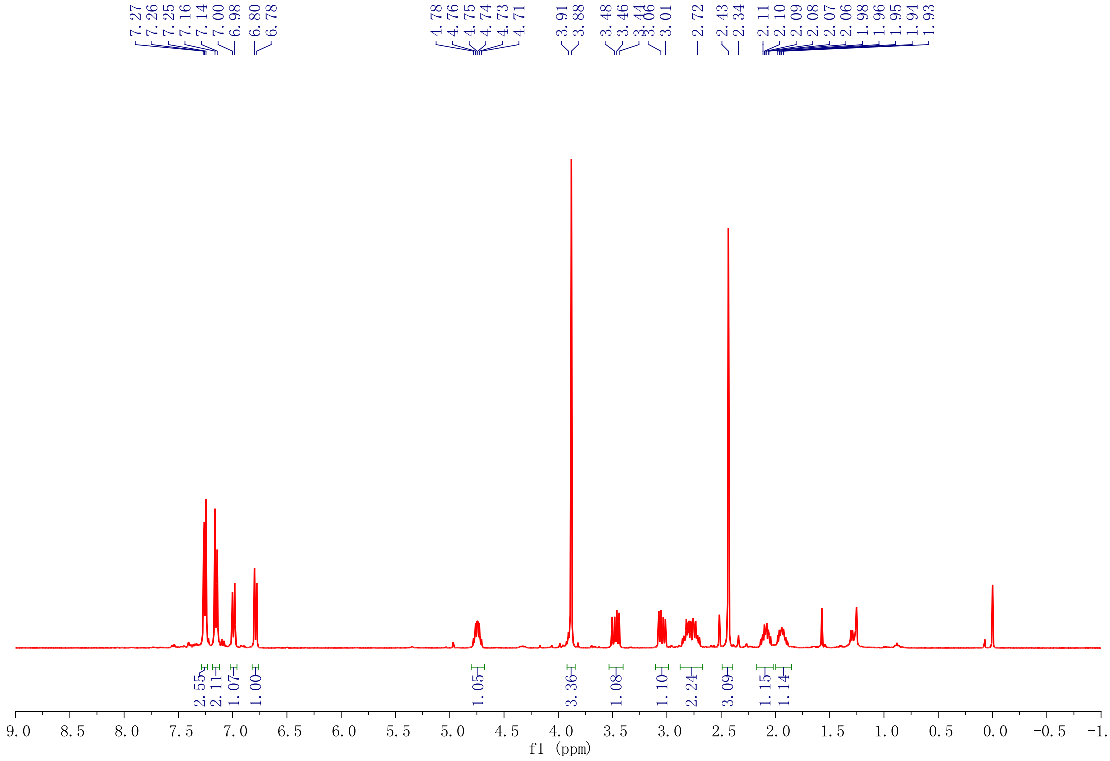

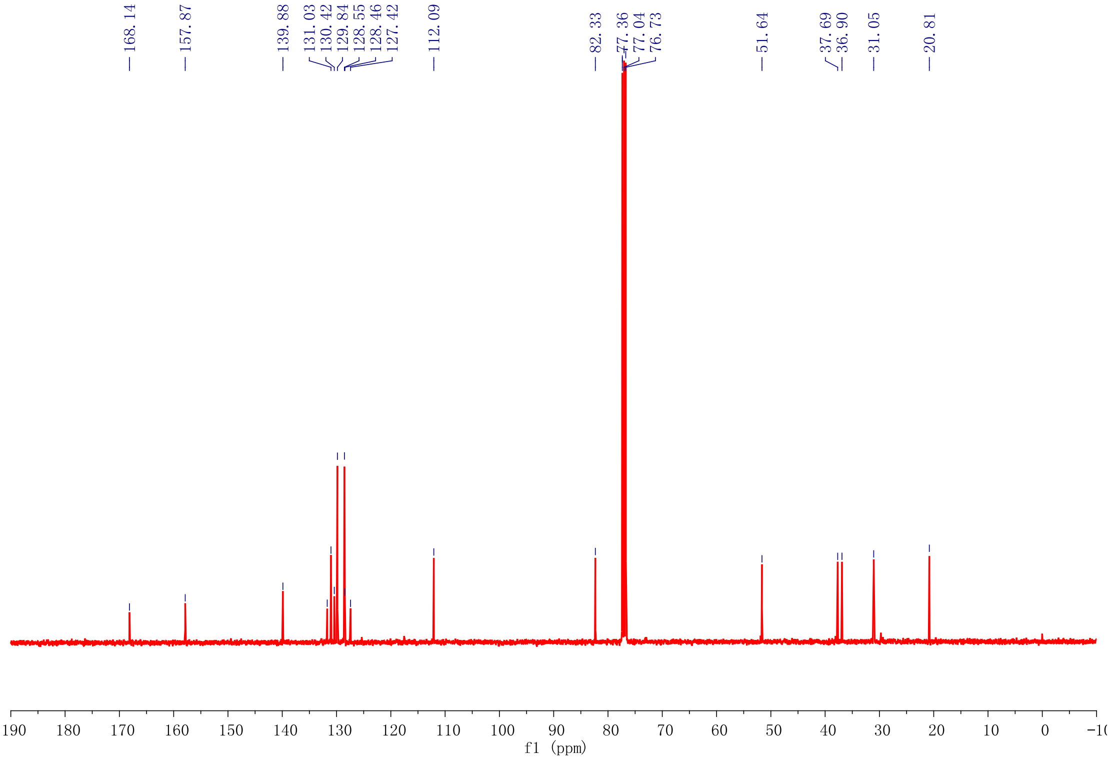

Supplementary Figure 45. ^{1}H, ^{13}C-NMR spectra of product **2p**

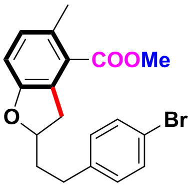

2p

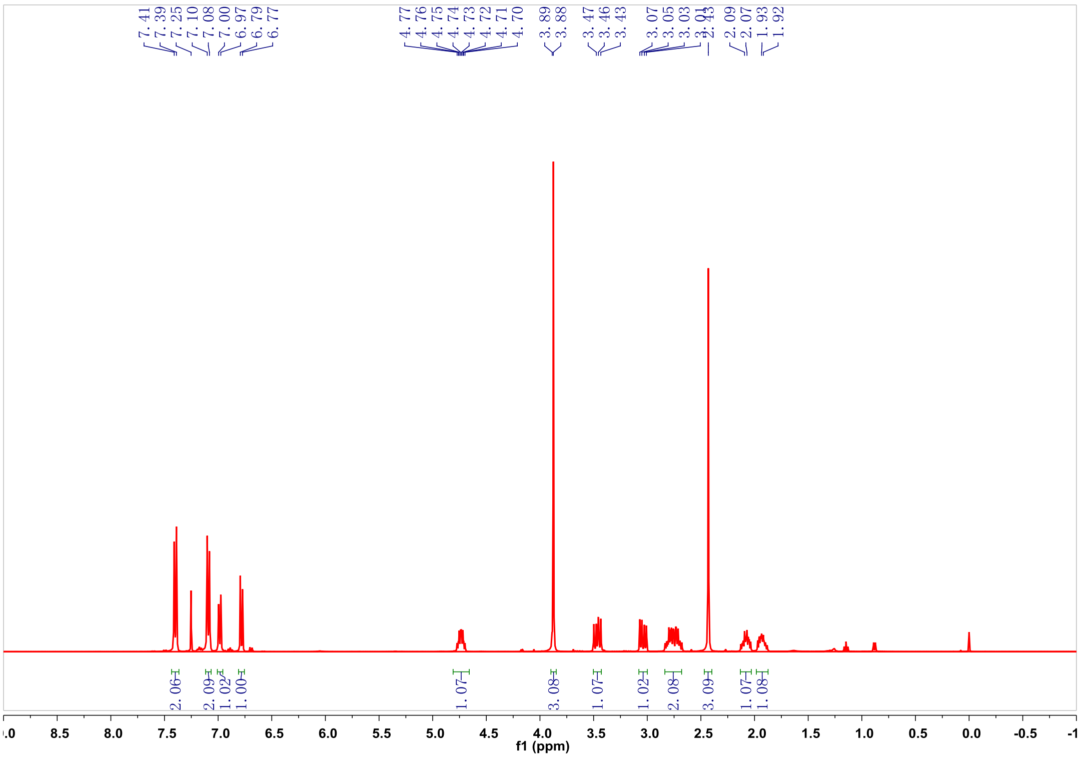

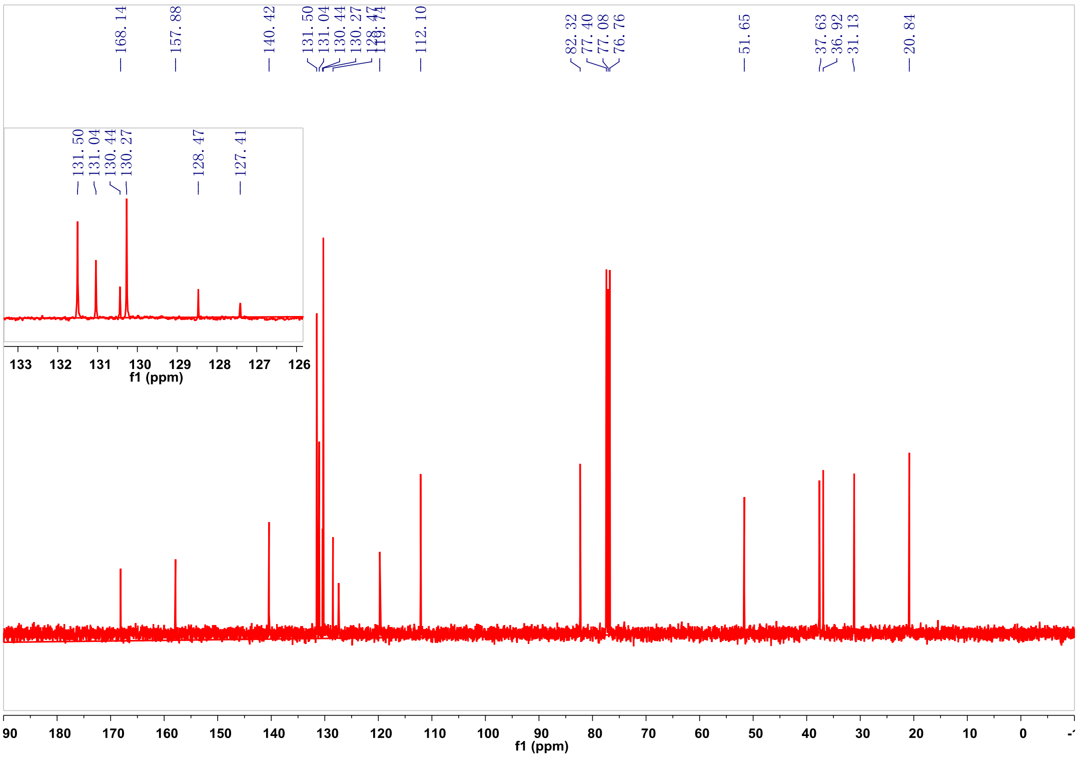

Supplementary Figure 46. ^{1}H, ^{13}C-NMR spectra of product **2q**

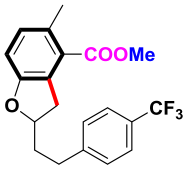

2q

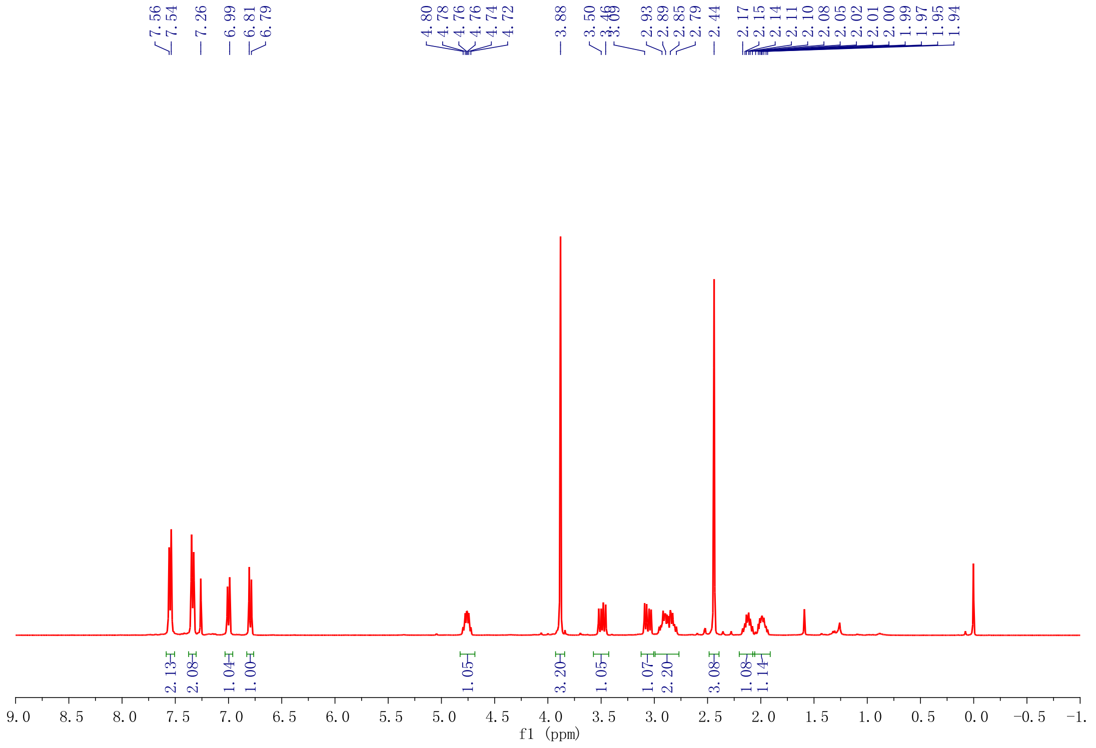

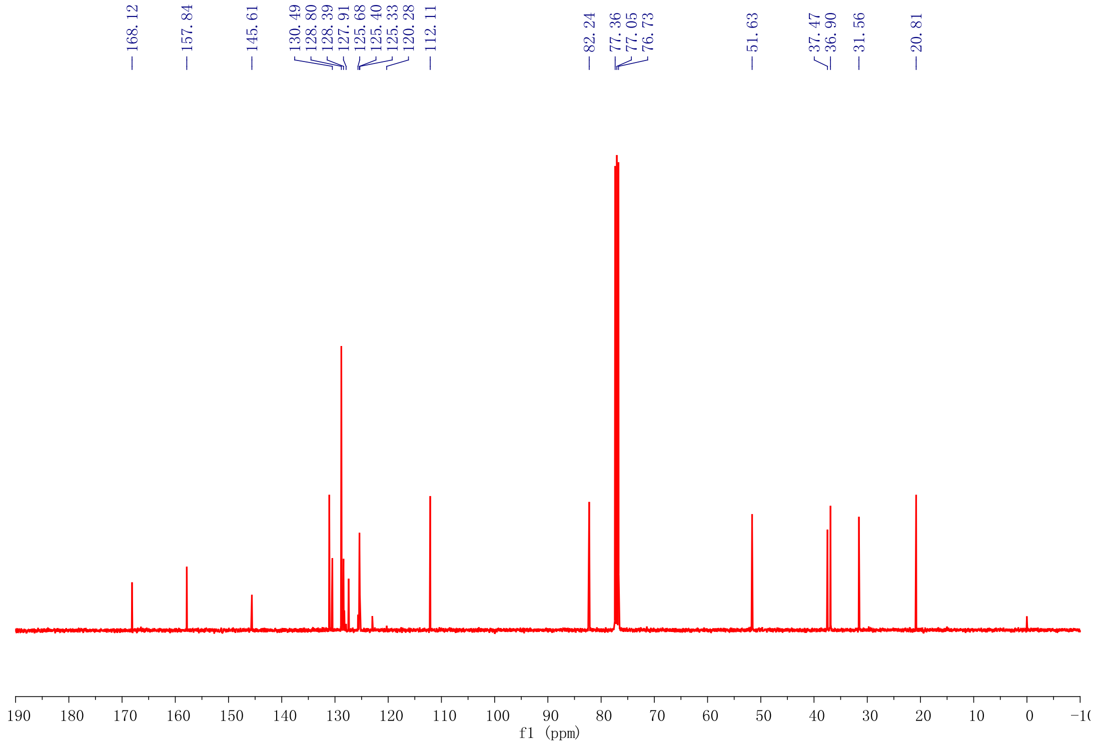

Supplementary Figure 47. ^{1}H, ^{13}C-NMR spectra of product **2r**

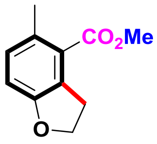

2r

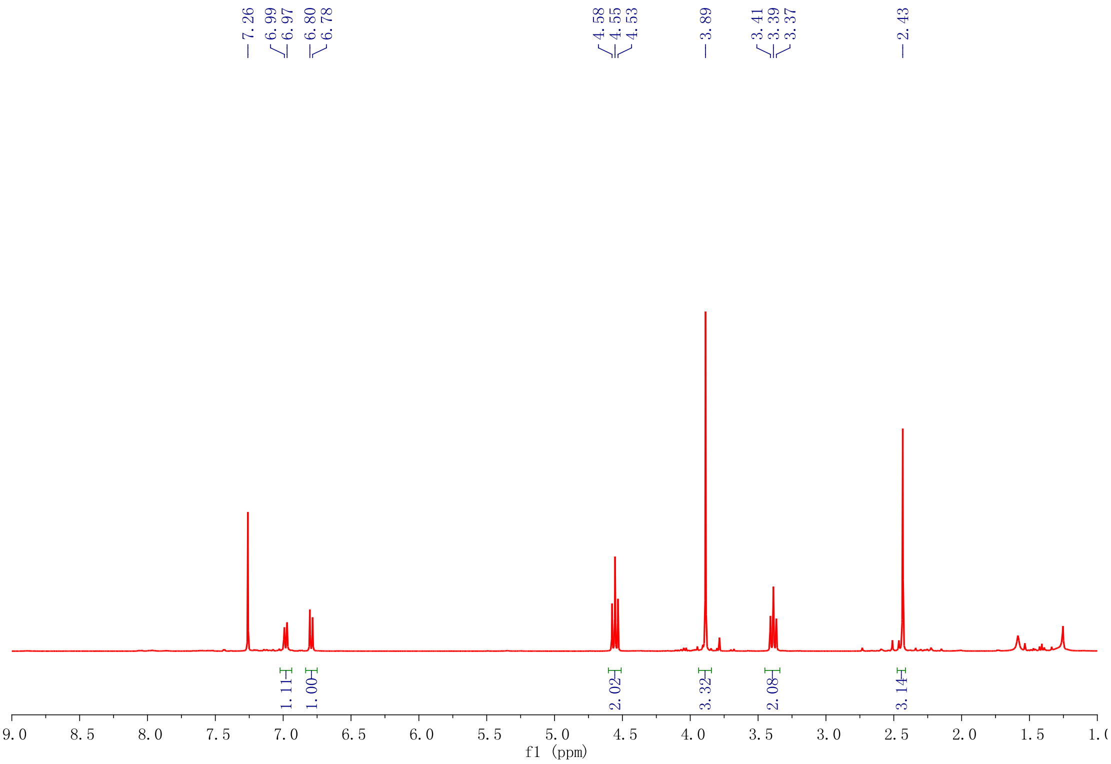

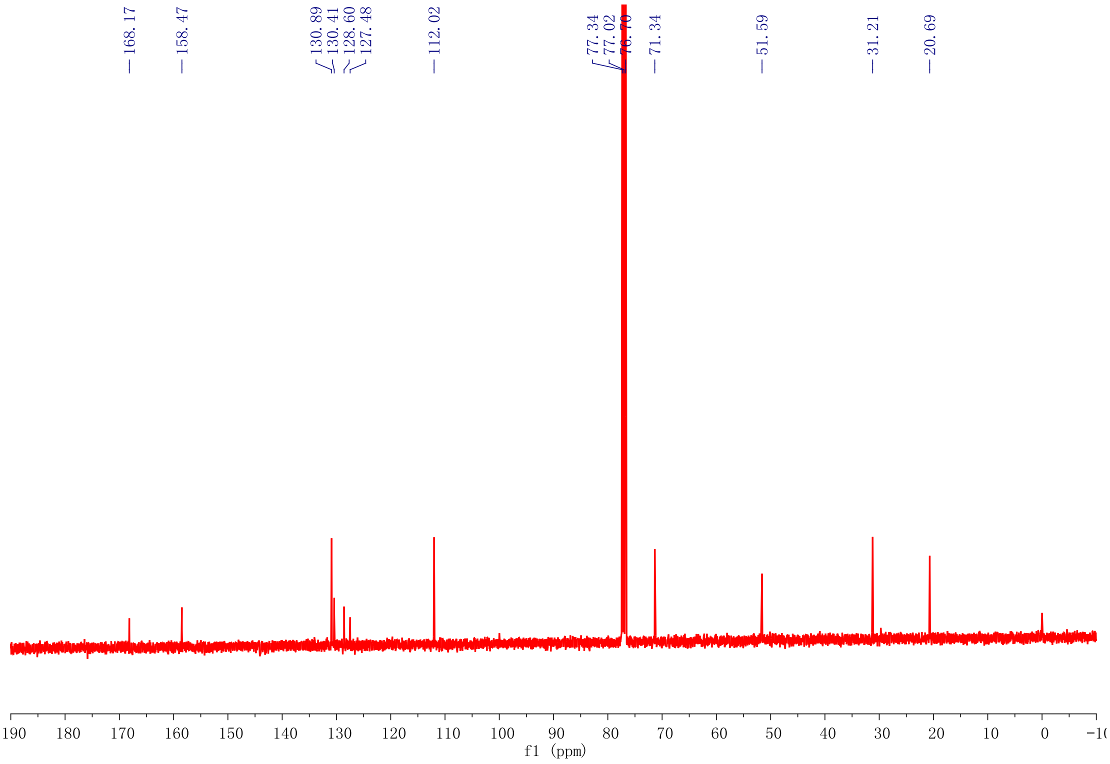

Supplementary Figure 48. ^{1}H, ^{13}C-NMR spectra of product **2s**

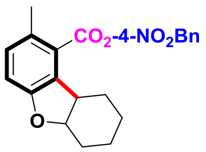

2s

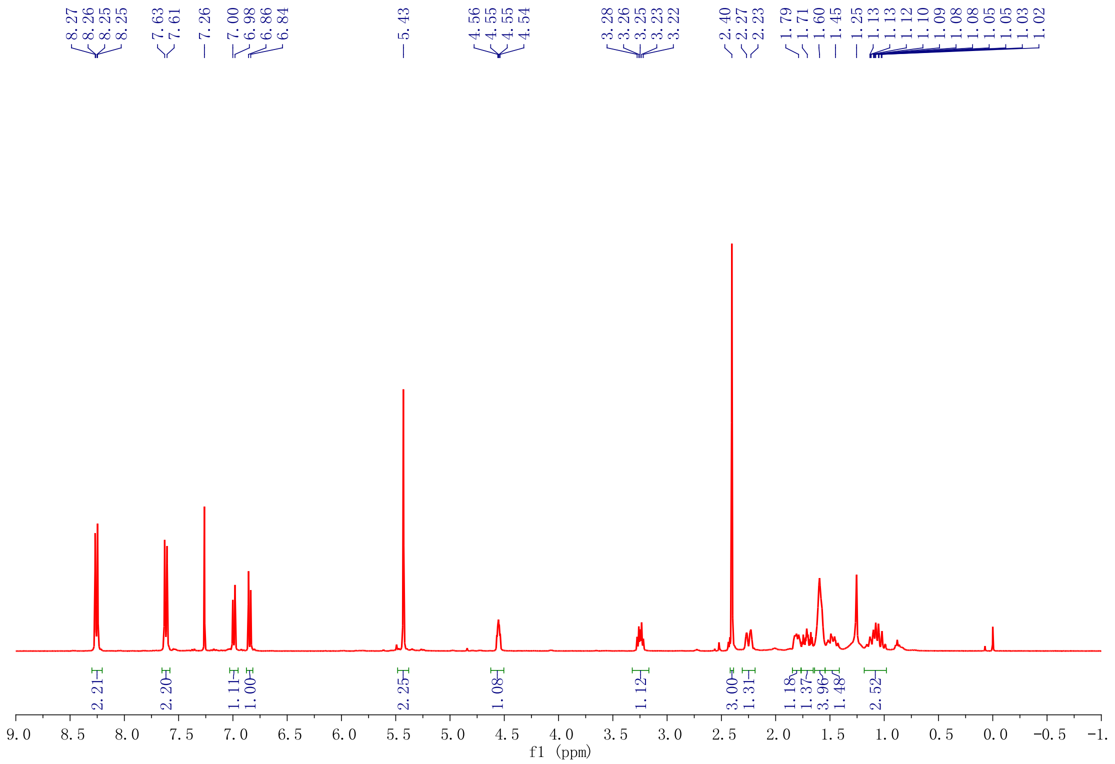

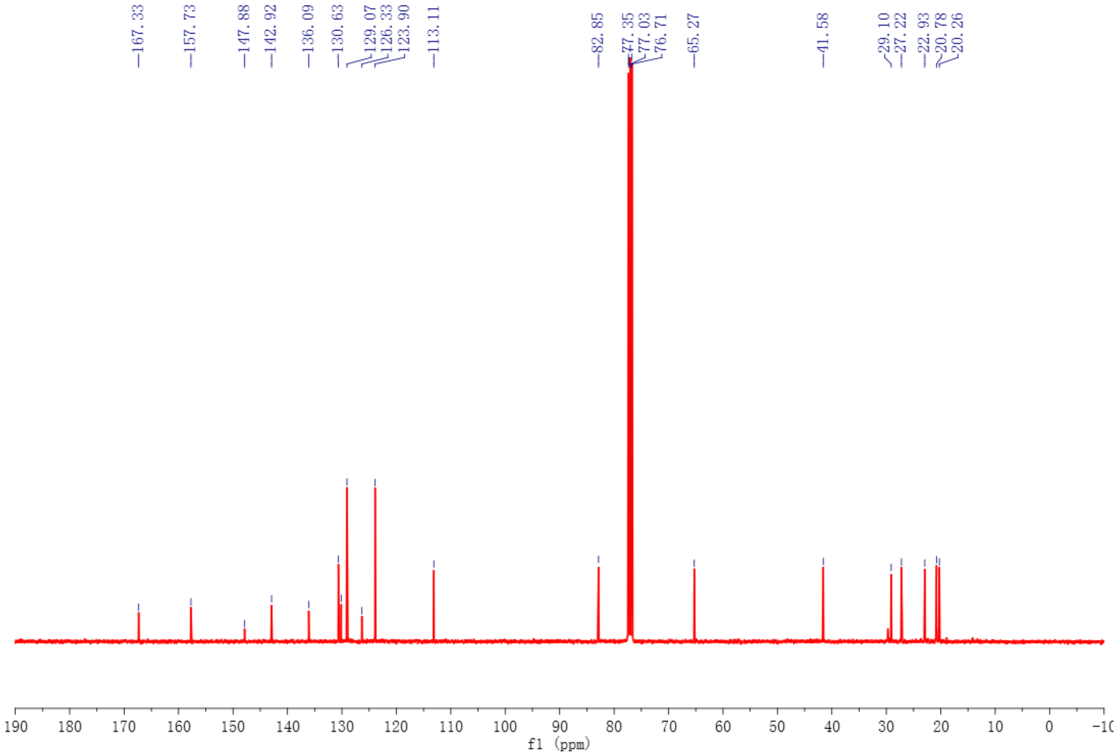

Supplementary Figure 49. ^{1}H, ^{13}C-NMR spectra of product **2t**

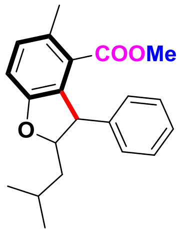

2t

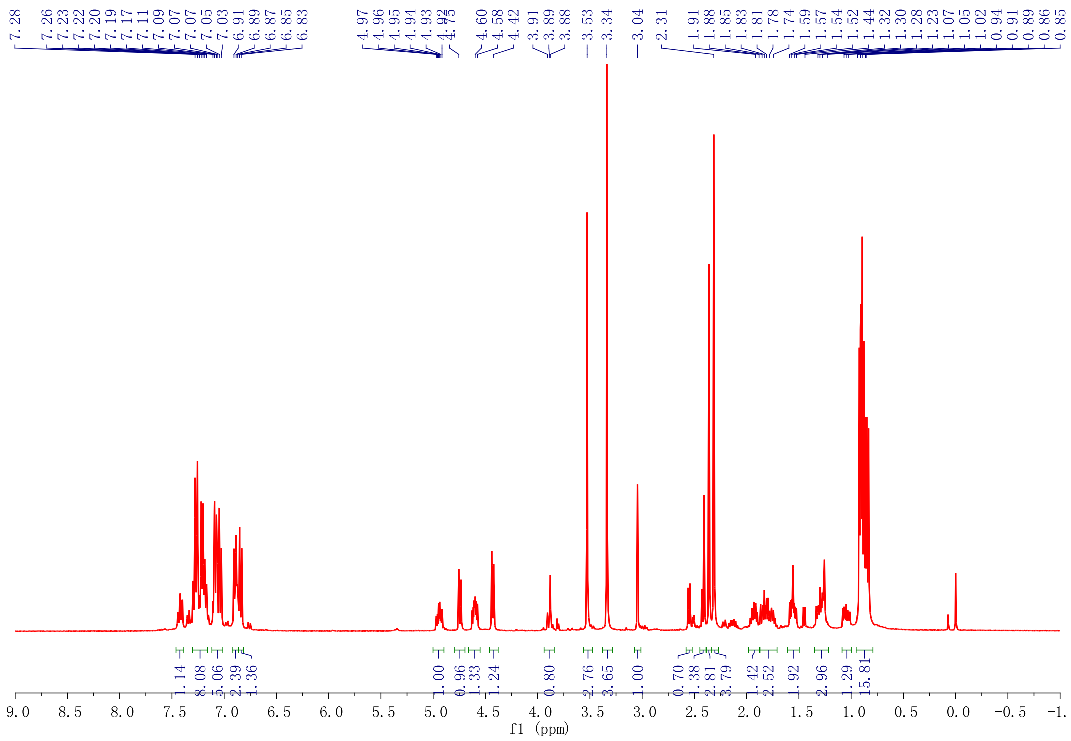

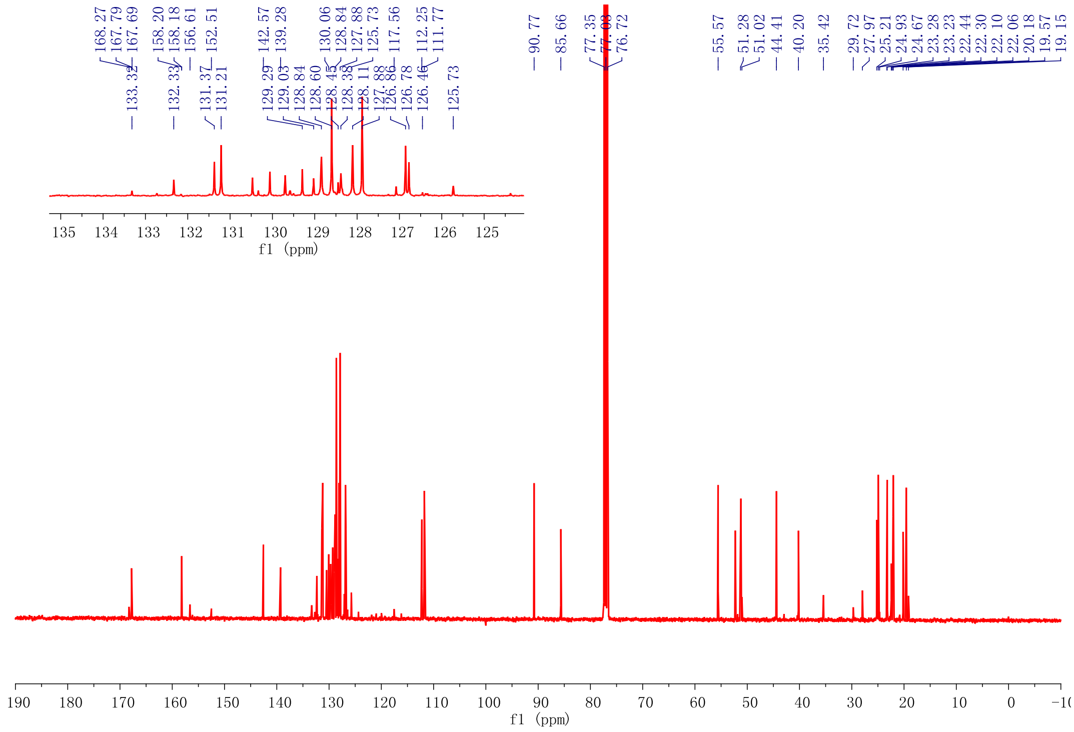

Supplementary Figure 50. ^{1}H, ^{13}C-NMR spectra of product **2u**

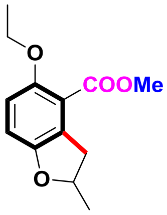

2u

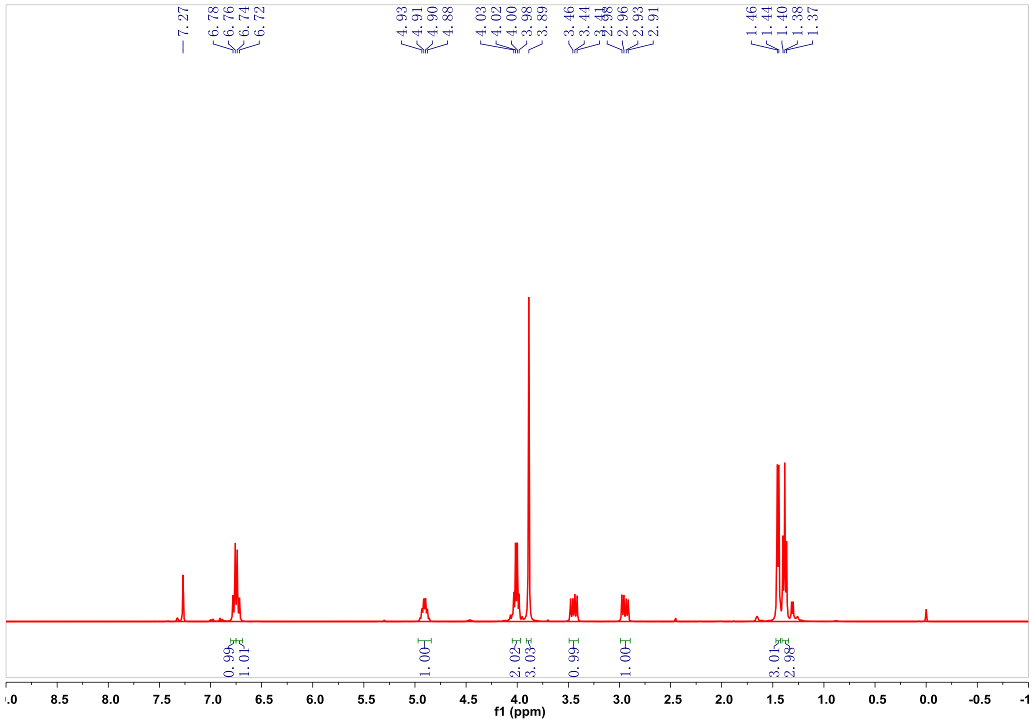

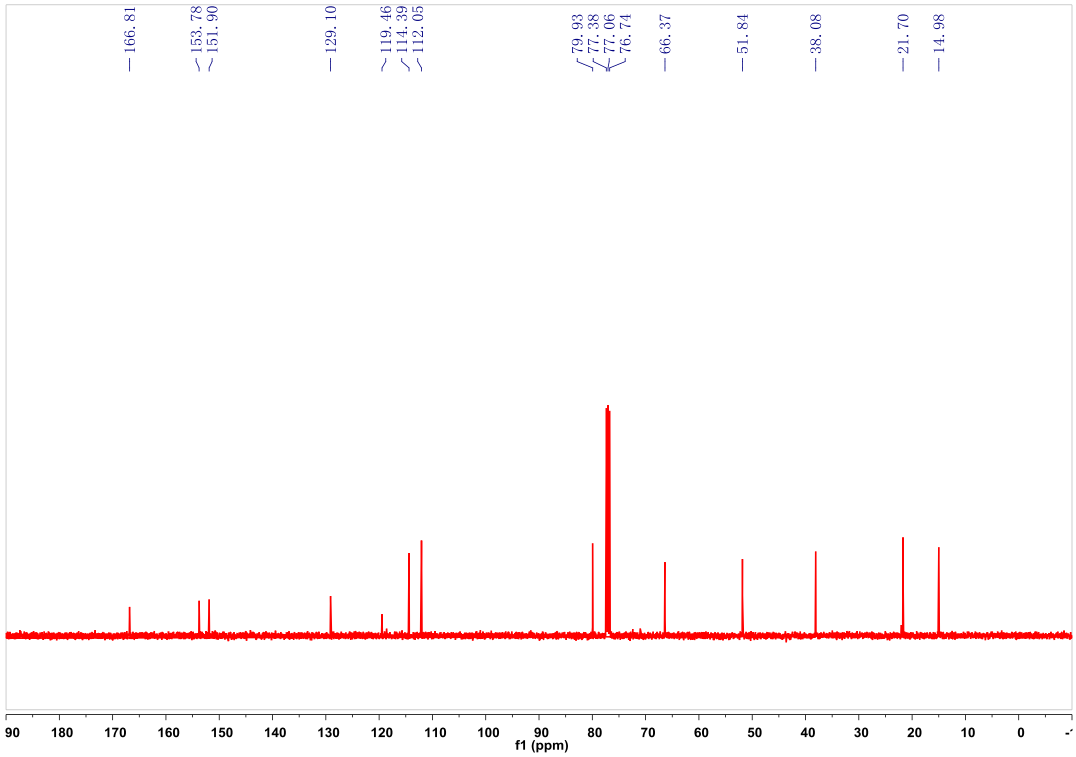

Supplementary Figure 51. ^{1}H, ^{13}C-NMR spectra of product **2v**

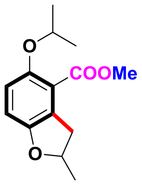

2v

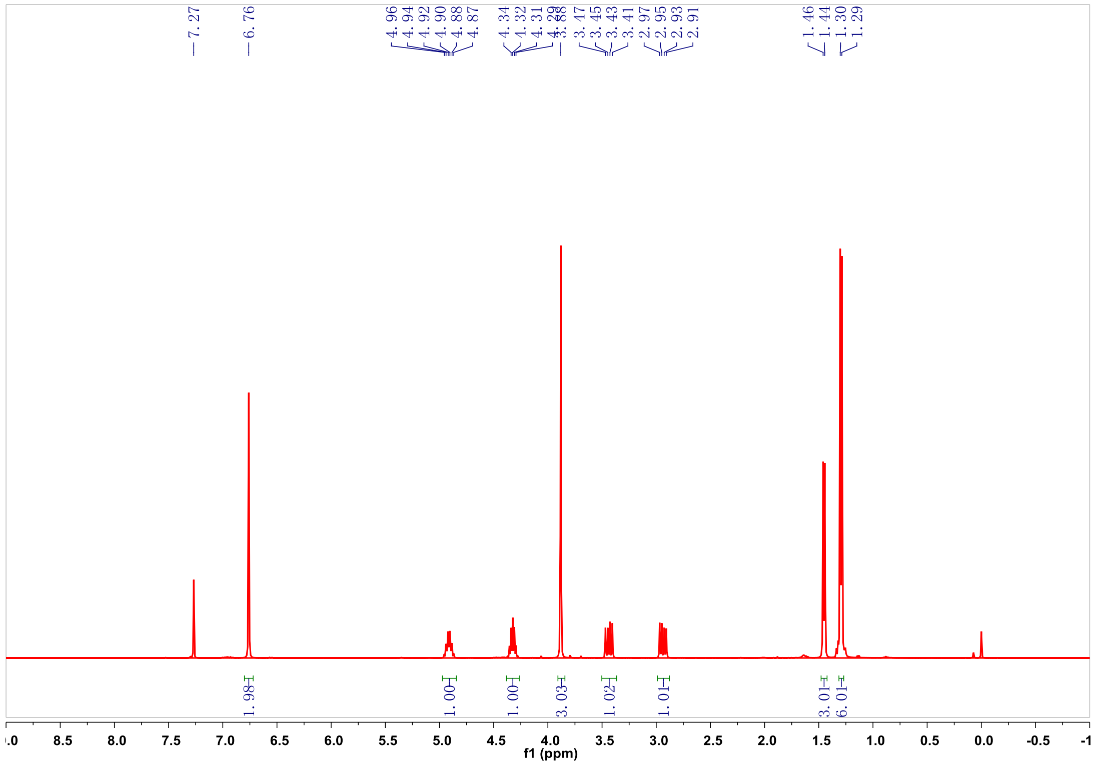

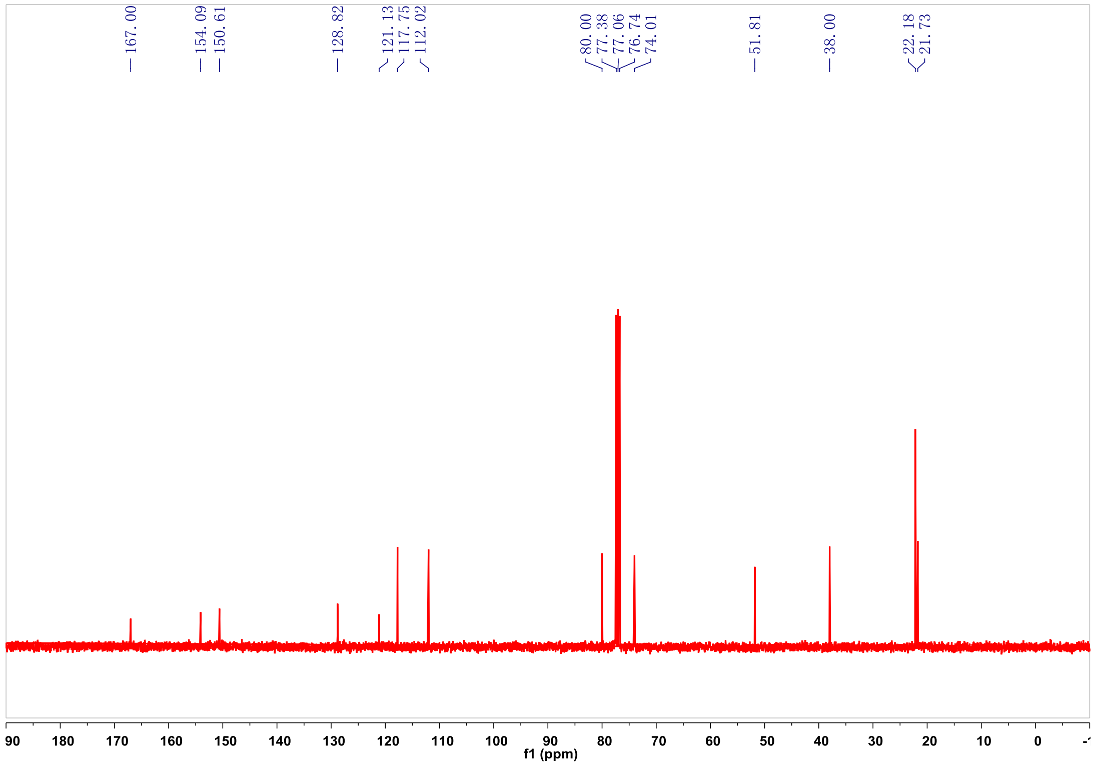

Supplementary Figure 52. ^{1}H, ^{13}C-NMR spectra of product **2w**

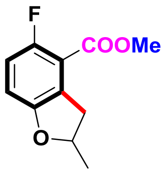

2w

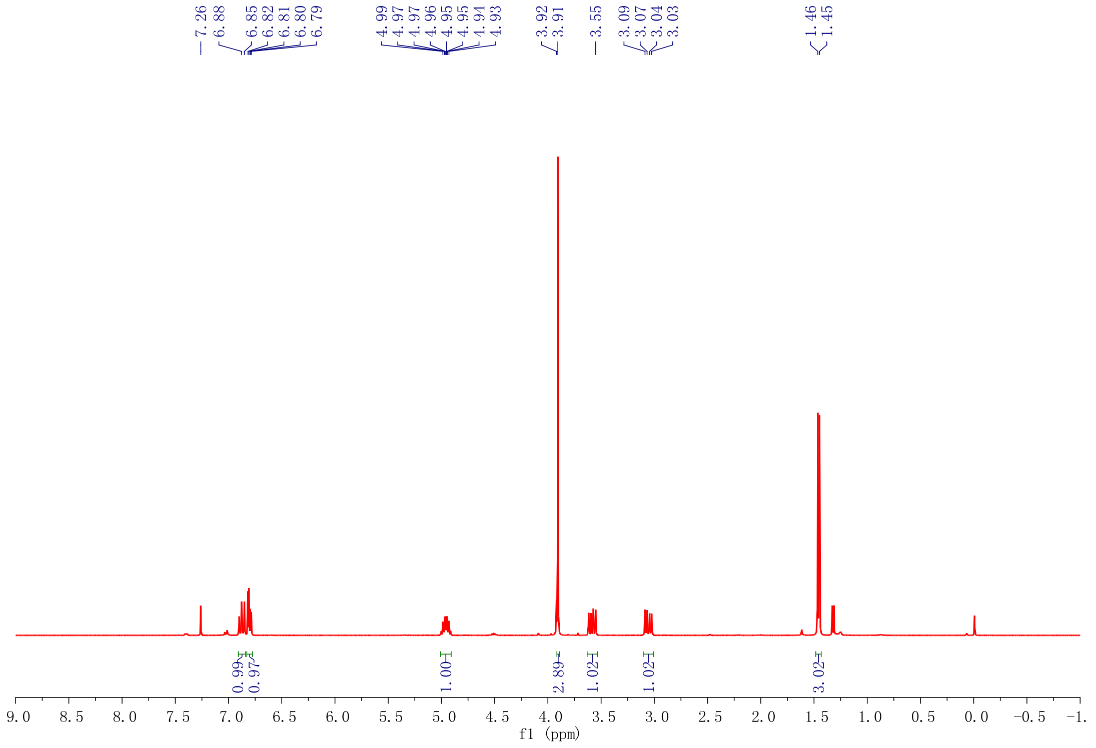

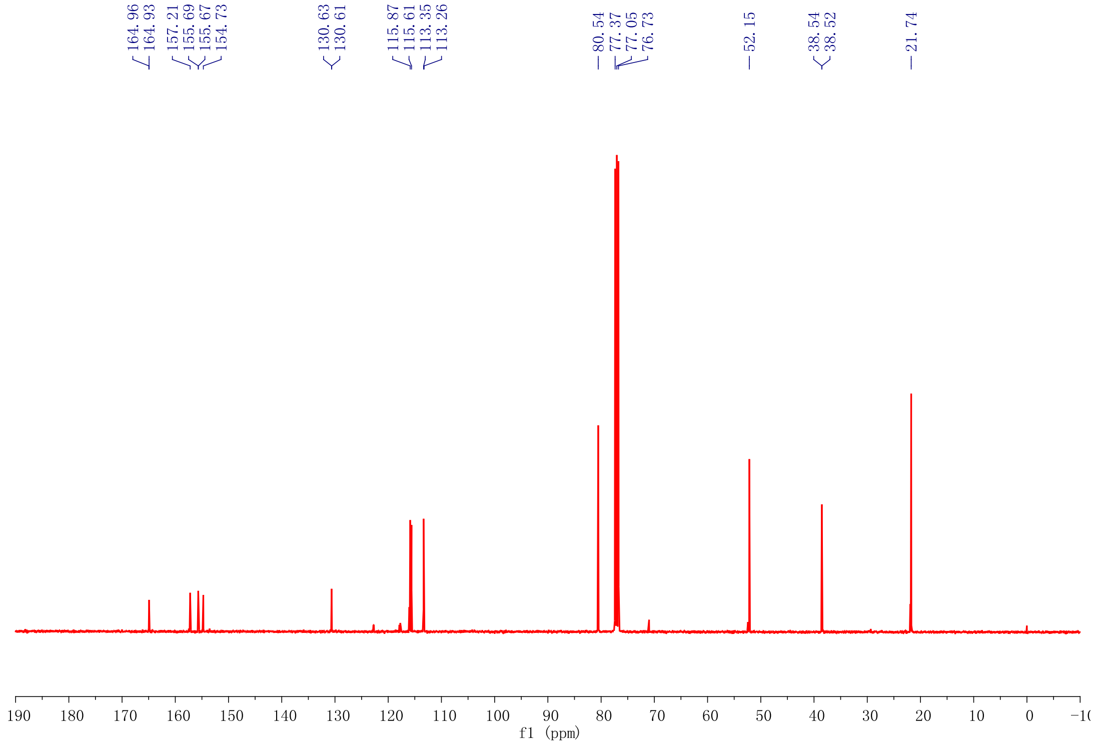

Supplementary Figure 53. ^{1}H, ^{13}C-NMR spectra of product **2x**

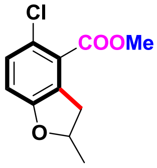

2x

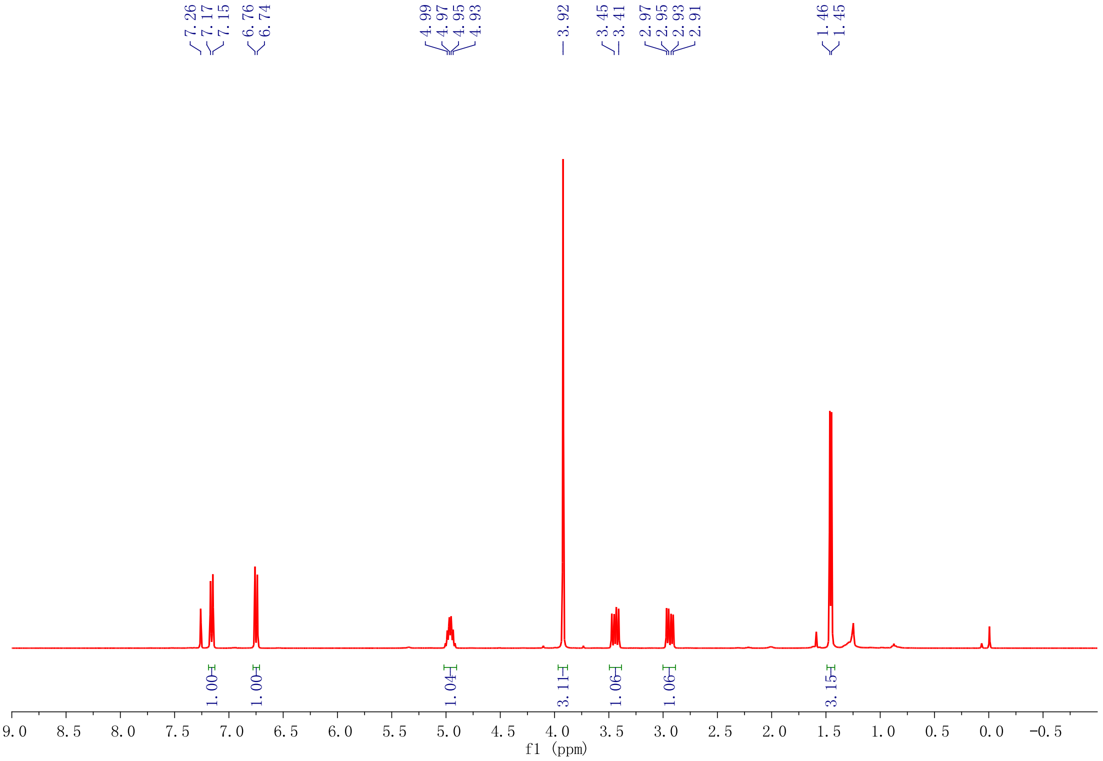

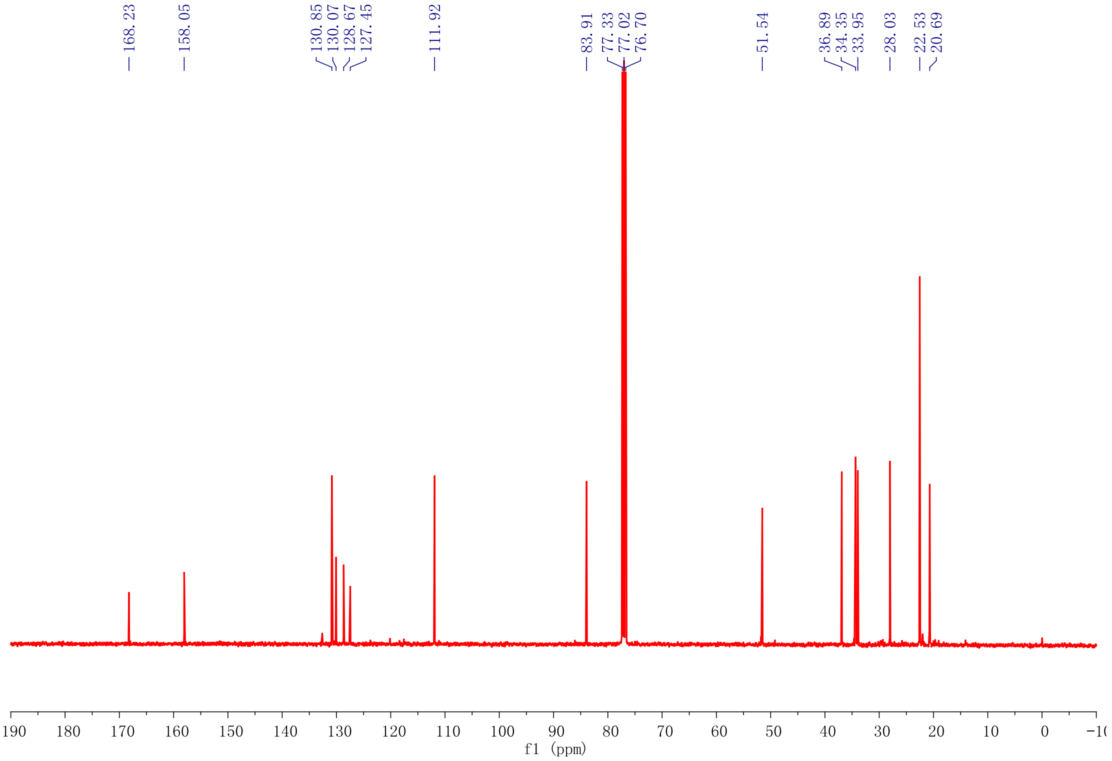

Supplementary Figure 54. ^{1}H, ^{13}C-NMR spectra of product **2y**

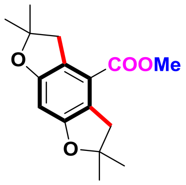

2y

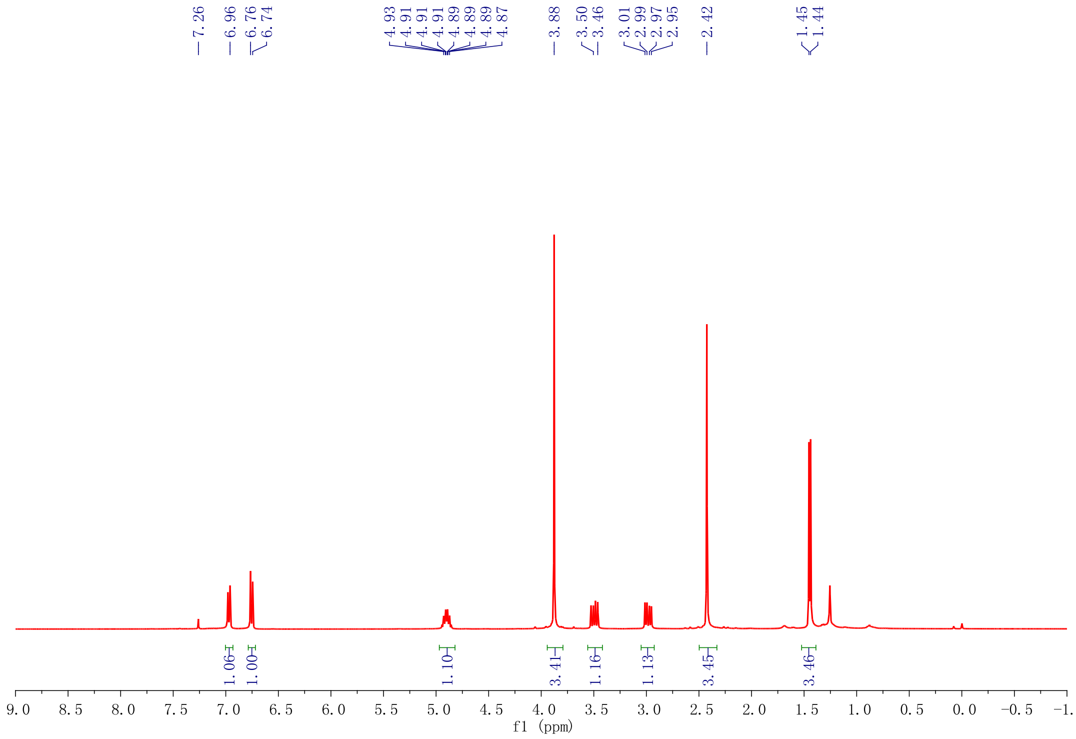

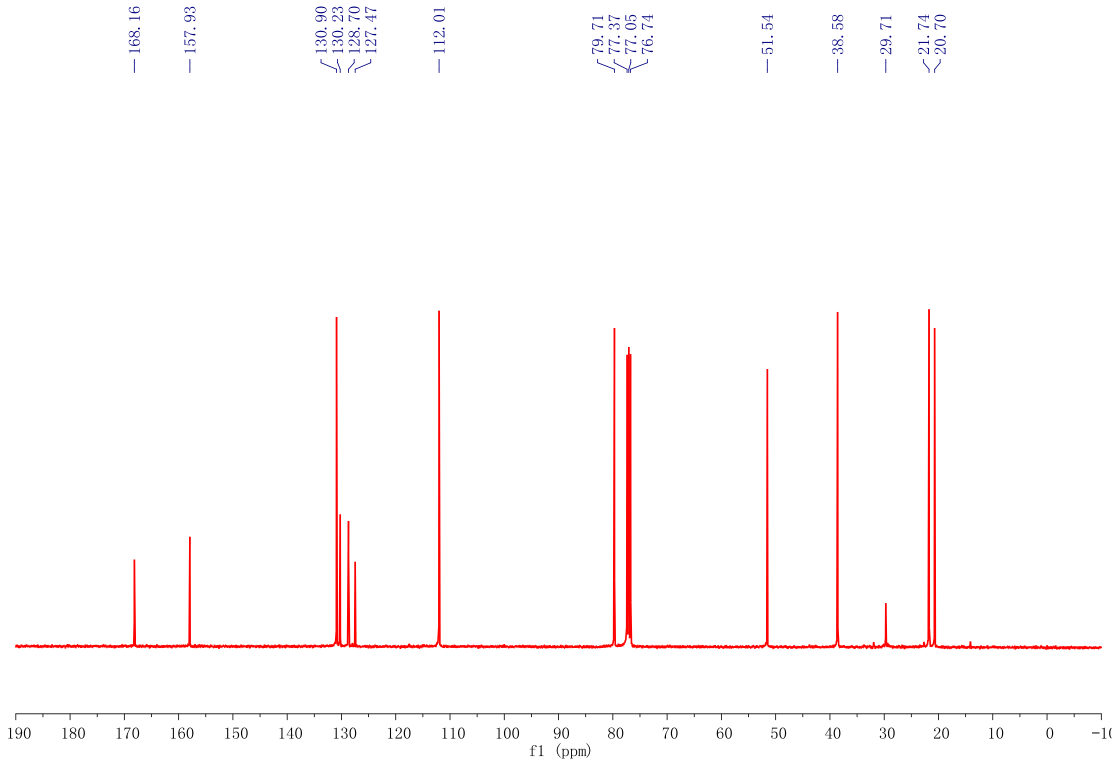

NMR Spectra of Corresponding Transformation Products

Supplementary Figure 55. ^{1}H, ^{13}C-NMR spectra of product **3**

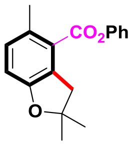

3

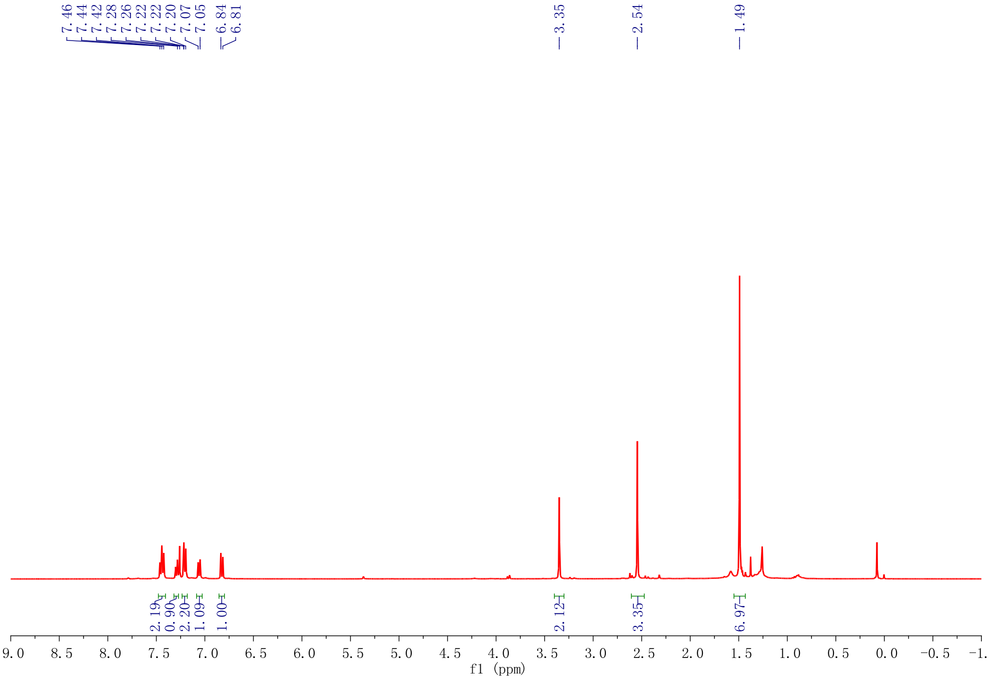

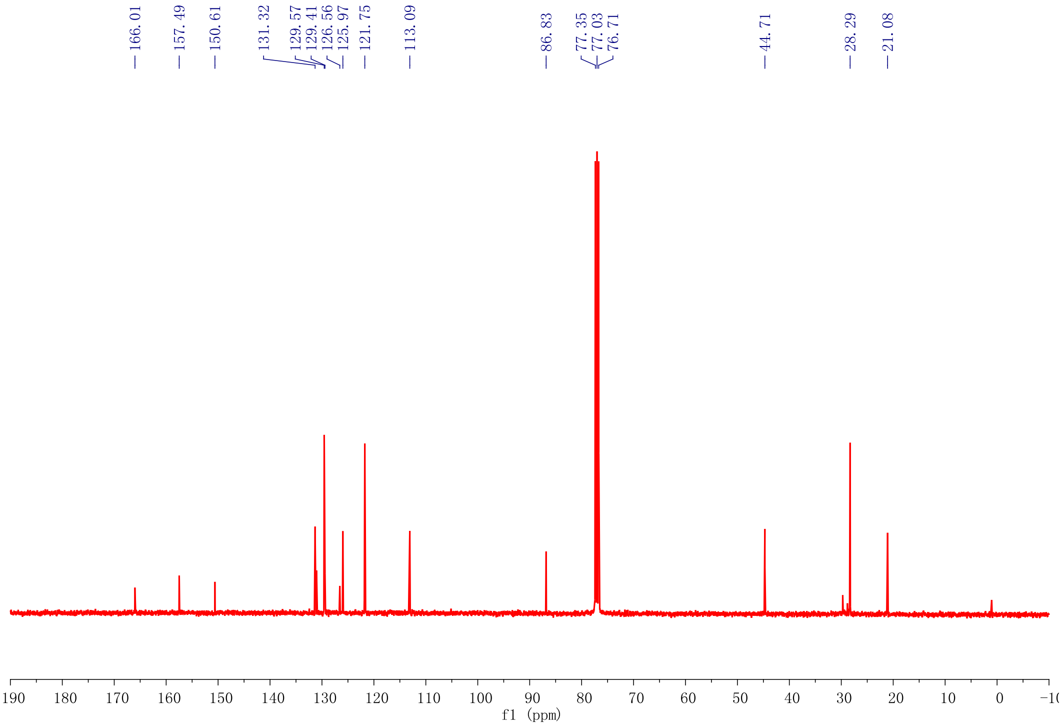

Supplementary Figure 56. ^{1}H, ^{13}C-NMR spectra of product **4**

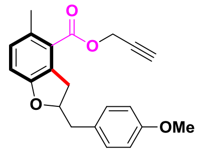
4

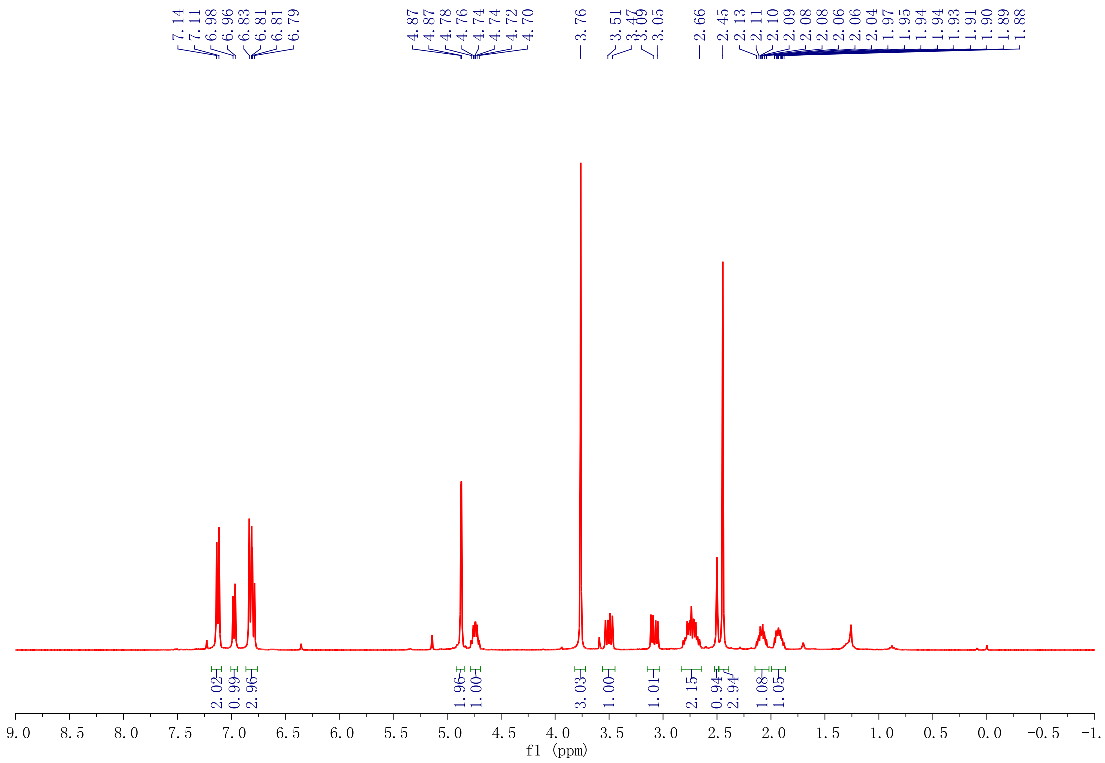

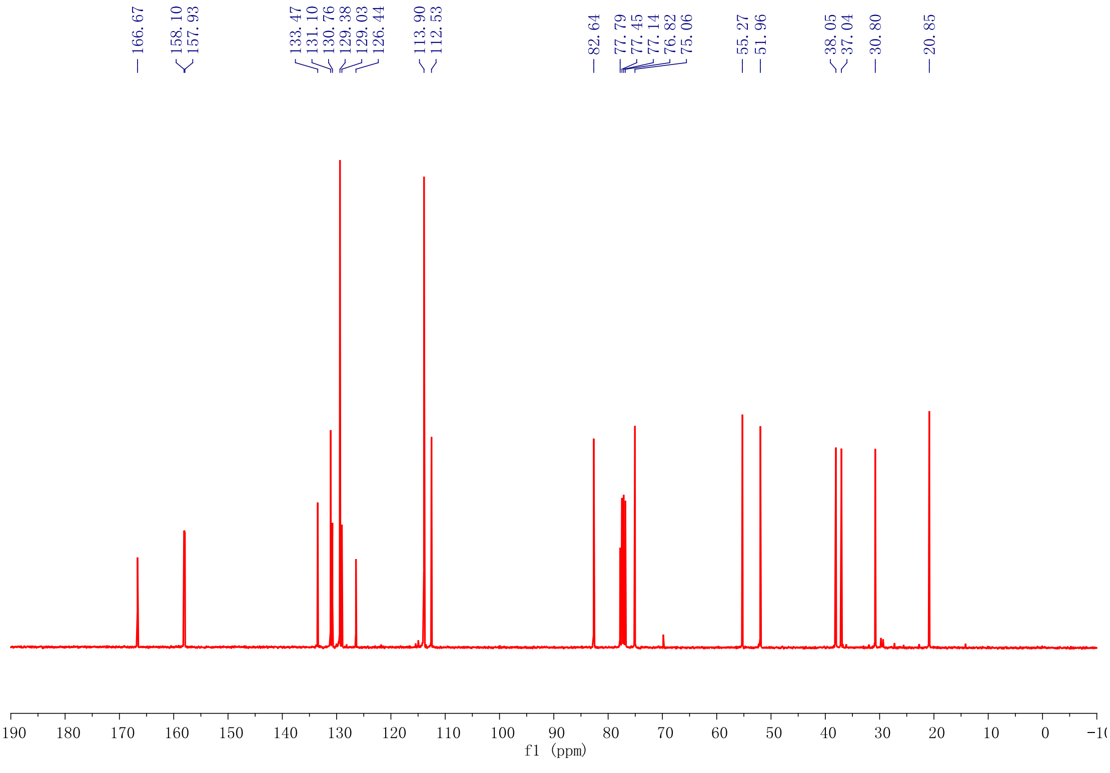

Supplementary Figure 57. ^{1}H, ^{13}C-NMR spectra of product **5**

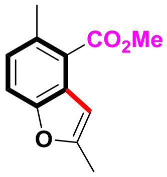

5

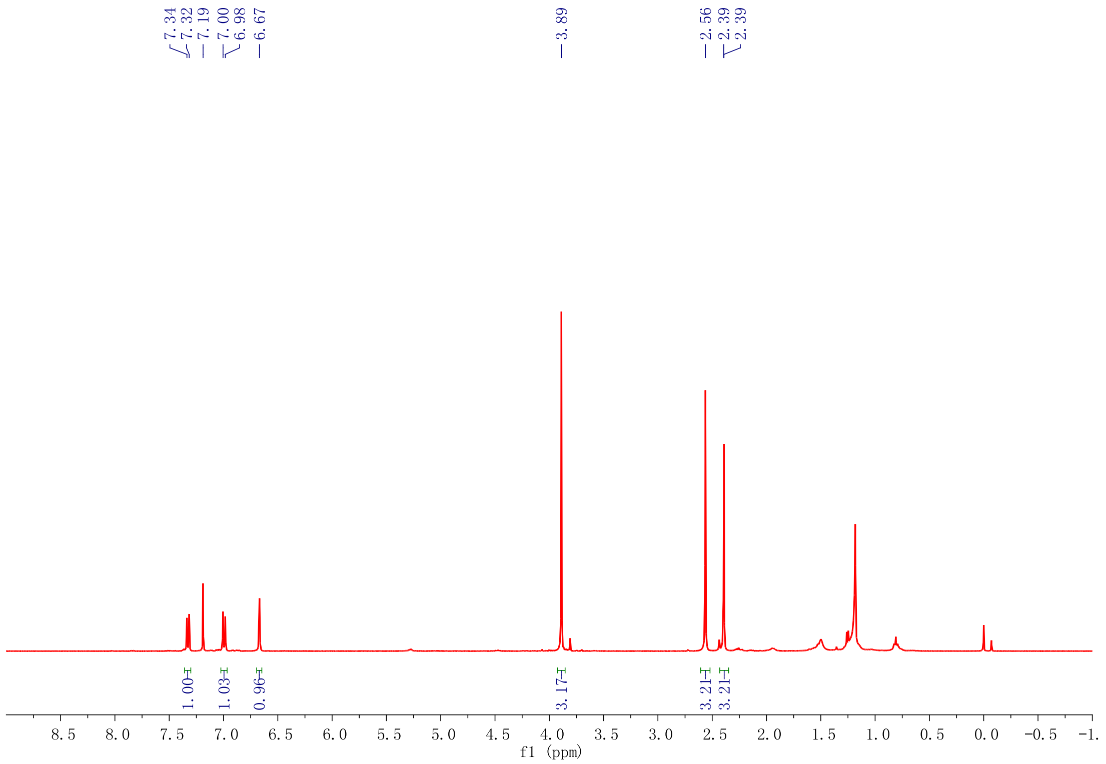

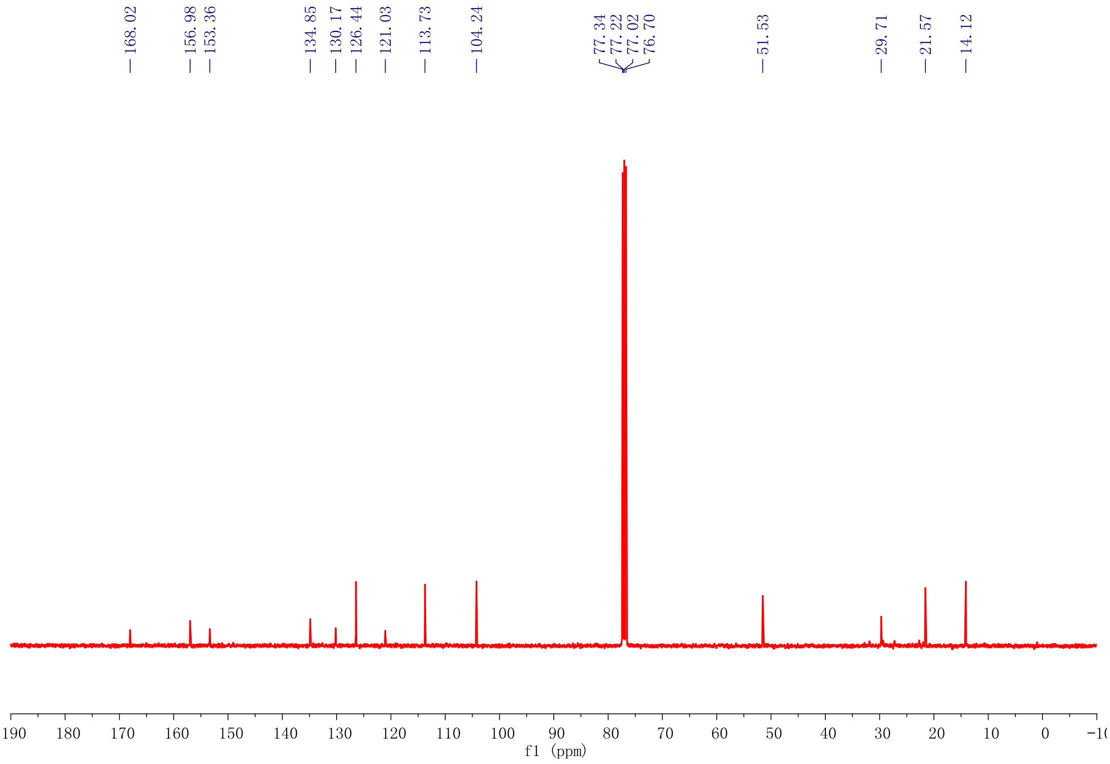

Supplementary Figure 58. ^{1}H, ^{13}C-NMR spectra of product **6**

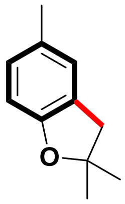

6

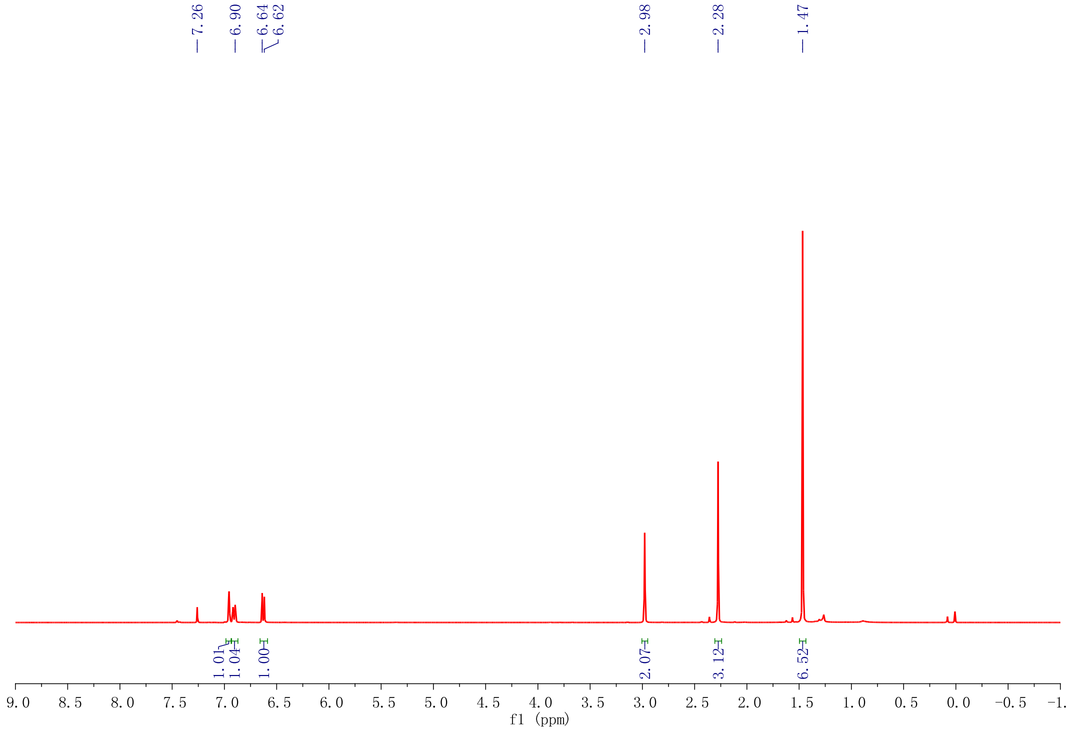

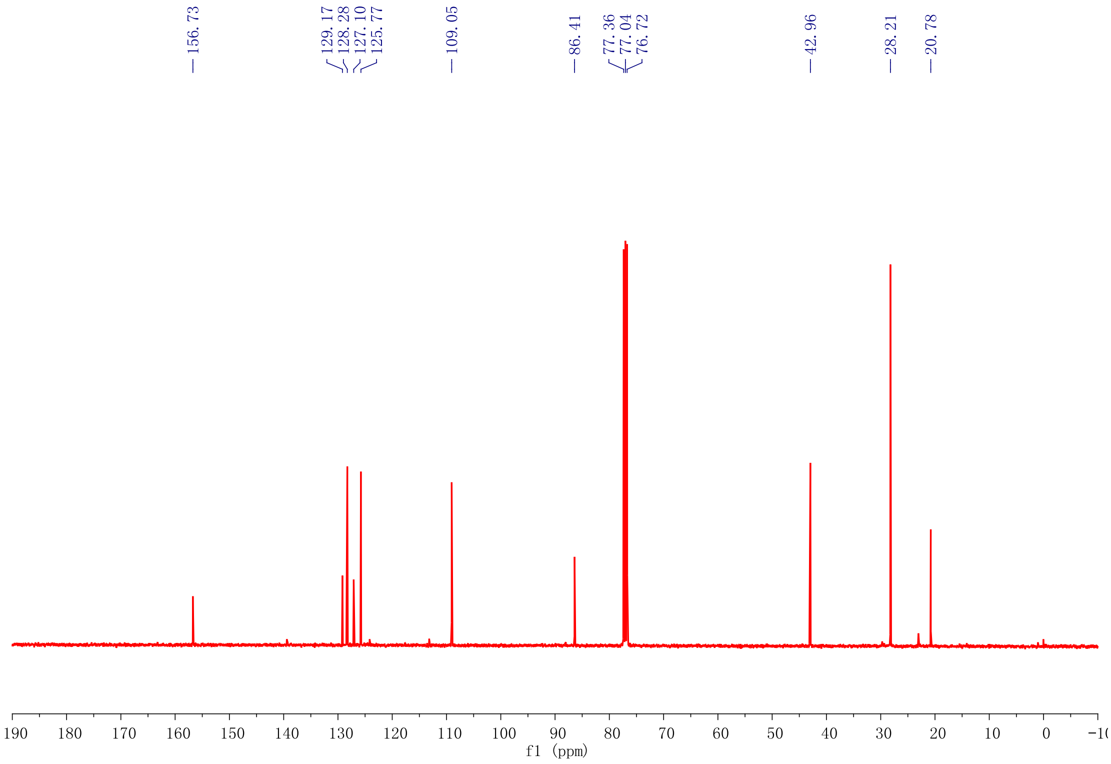

Supplementary Figure 59. ^{1}H, ^{13}C-NMR spectra of product **7**

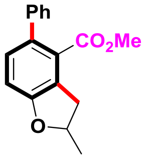

7

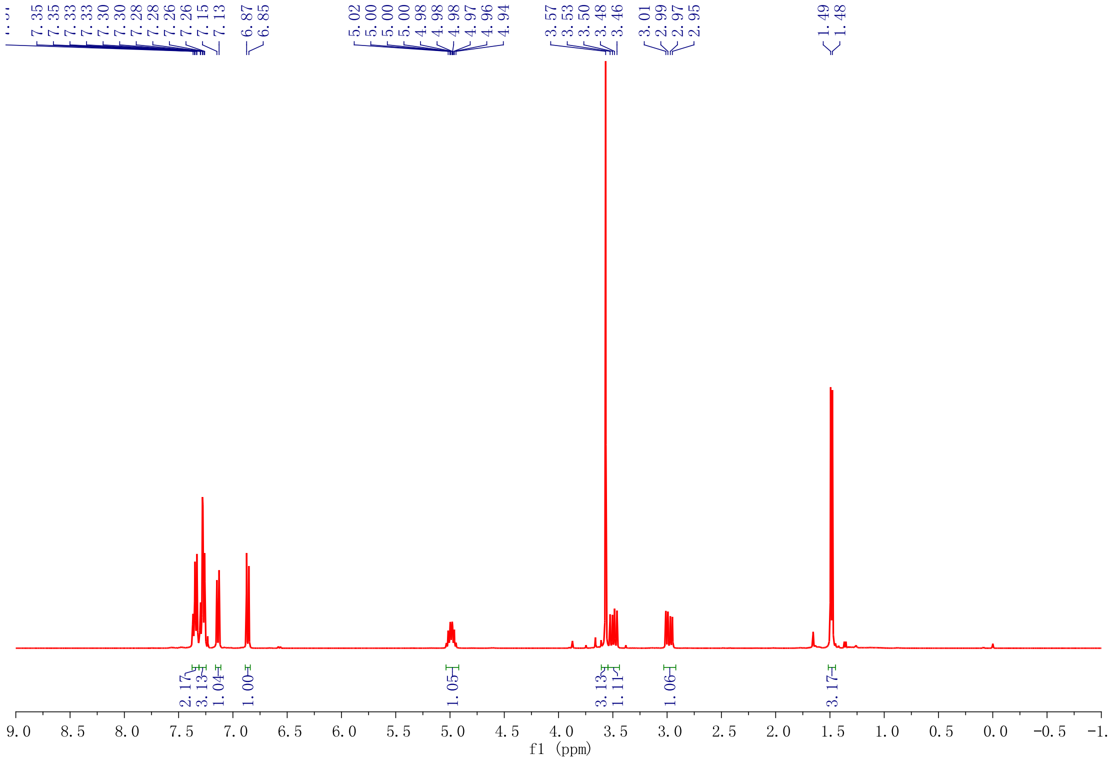

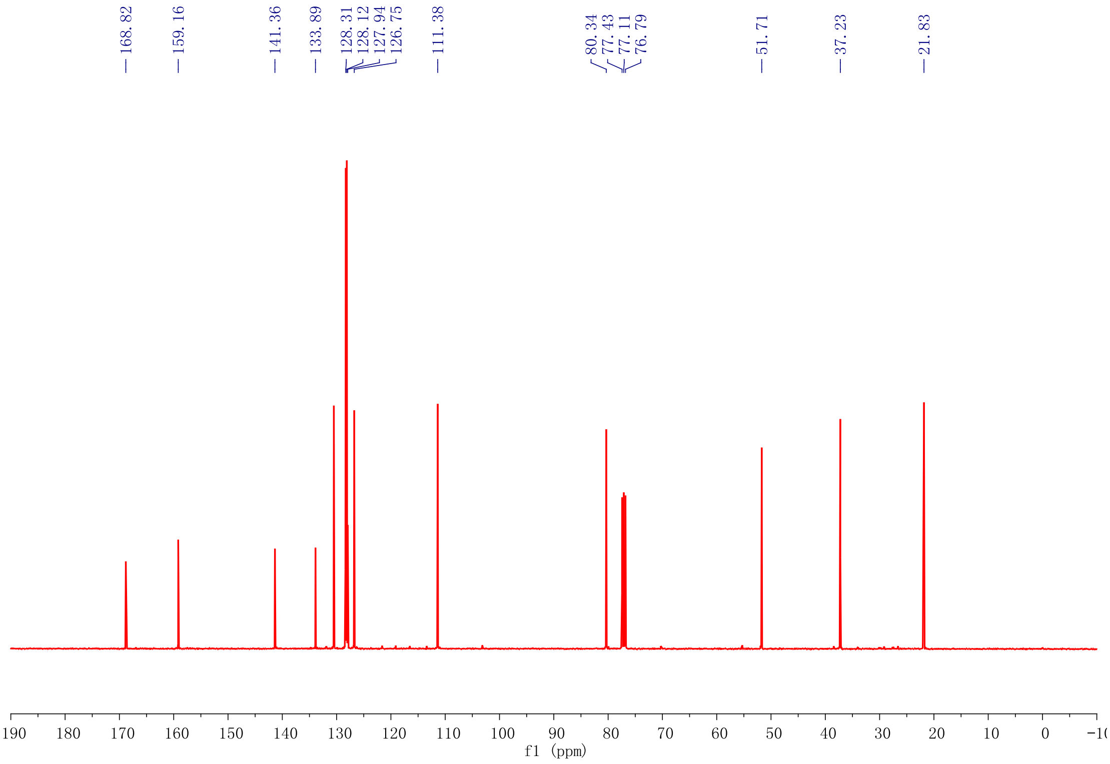

Supplementary Figure 60. ^{1}H, ^{13}C-NMR spectra of product **8**

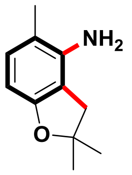

8

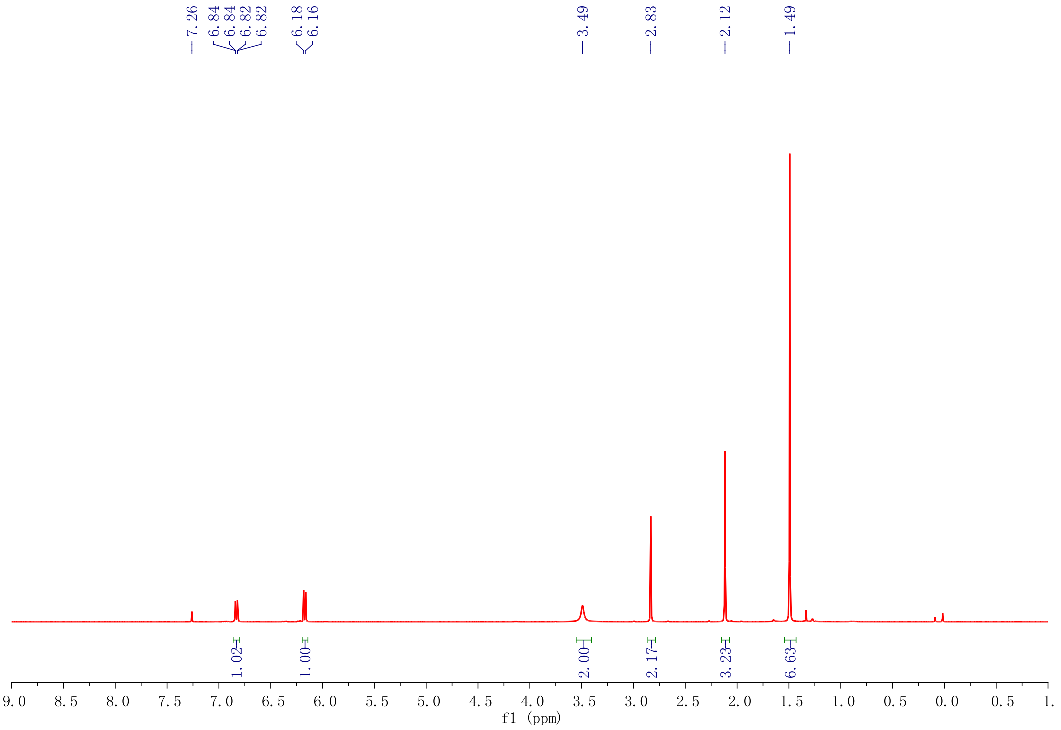

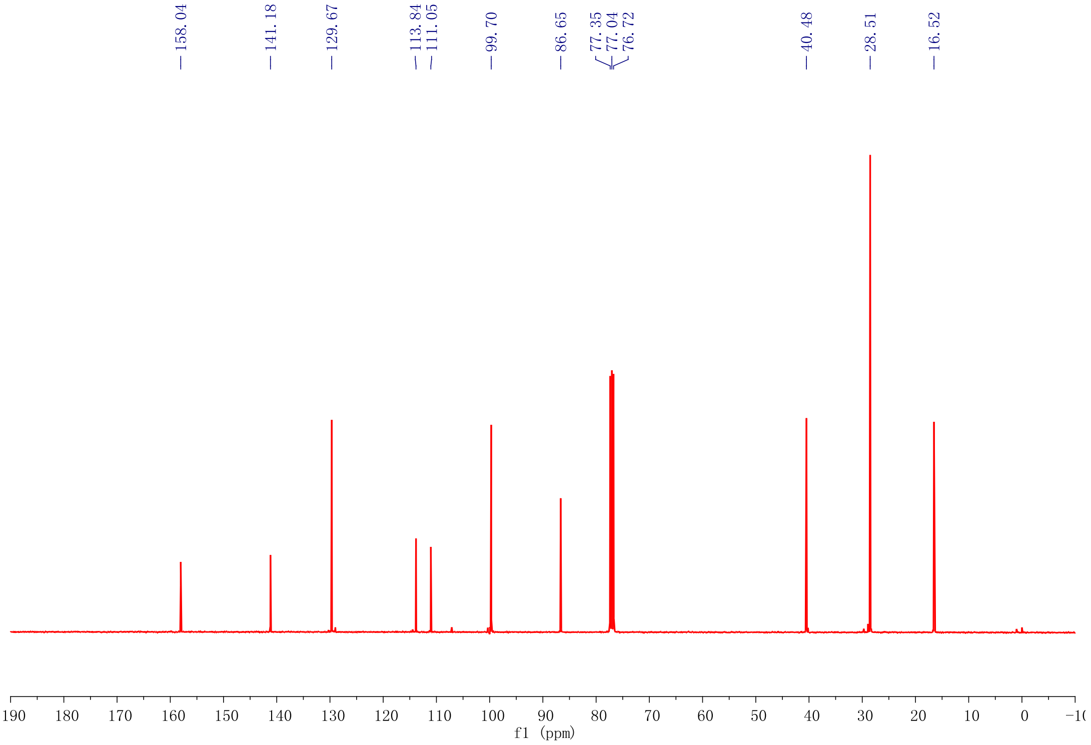

Supplementary Figure 61. ^{1}H, ^{13}C-NMR spectra of product **9**

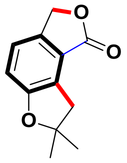

9

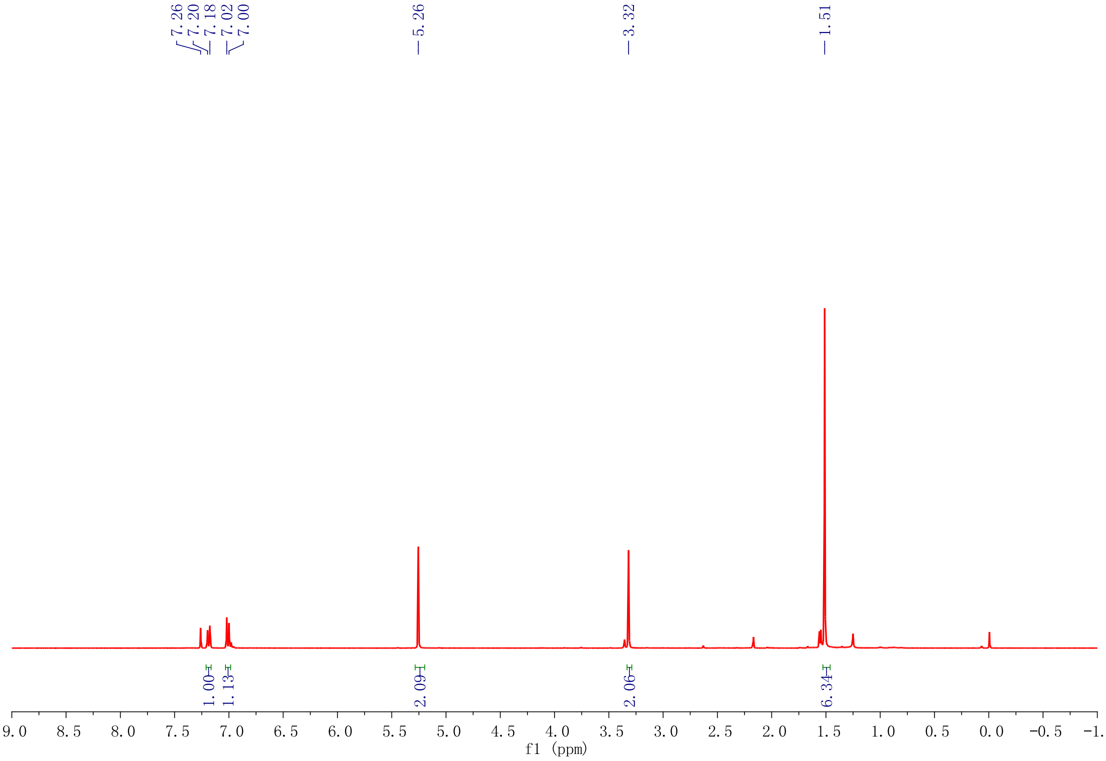

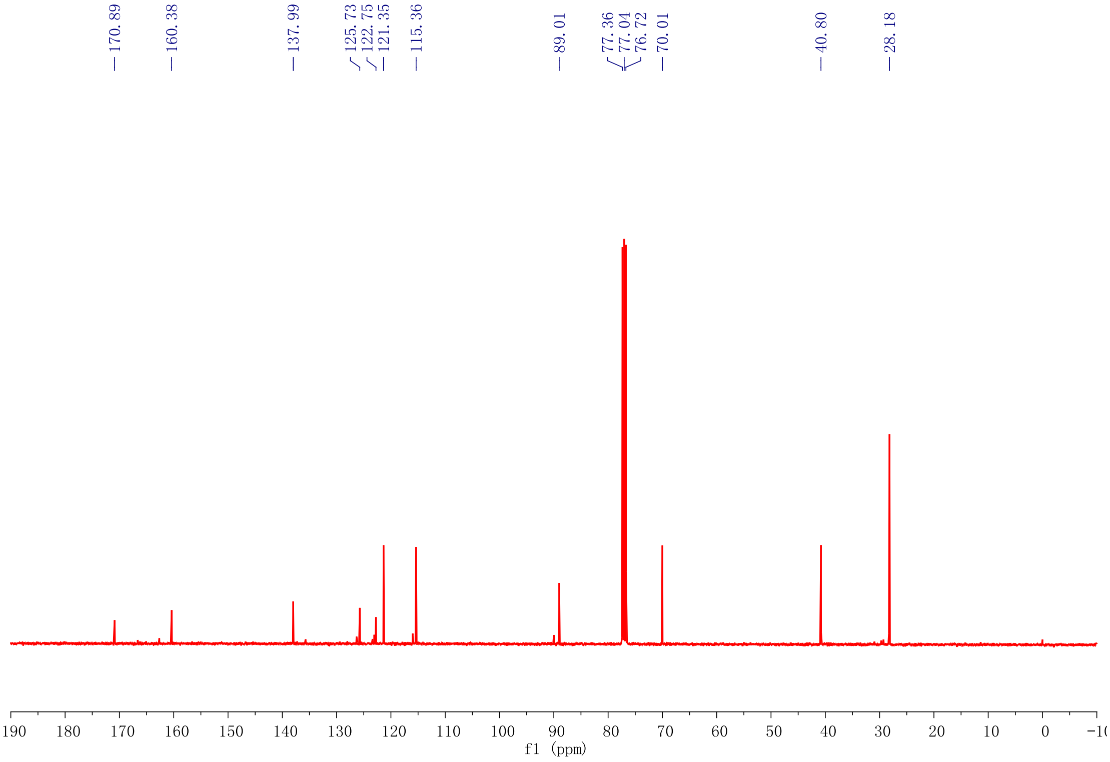

Supplementary Figure 62. ^{1}H, ^{13}C-NMR spectra of product **2i-1**

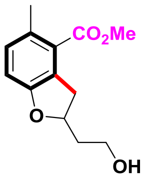

2i-1

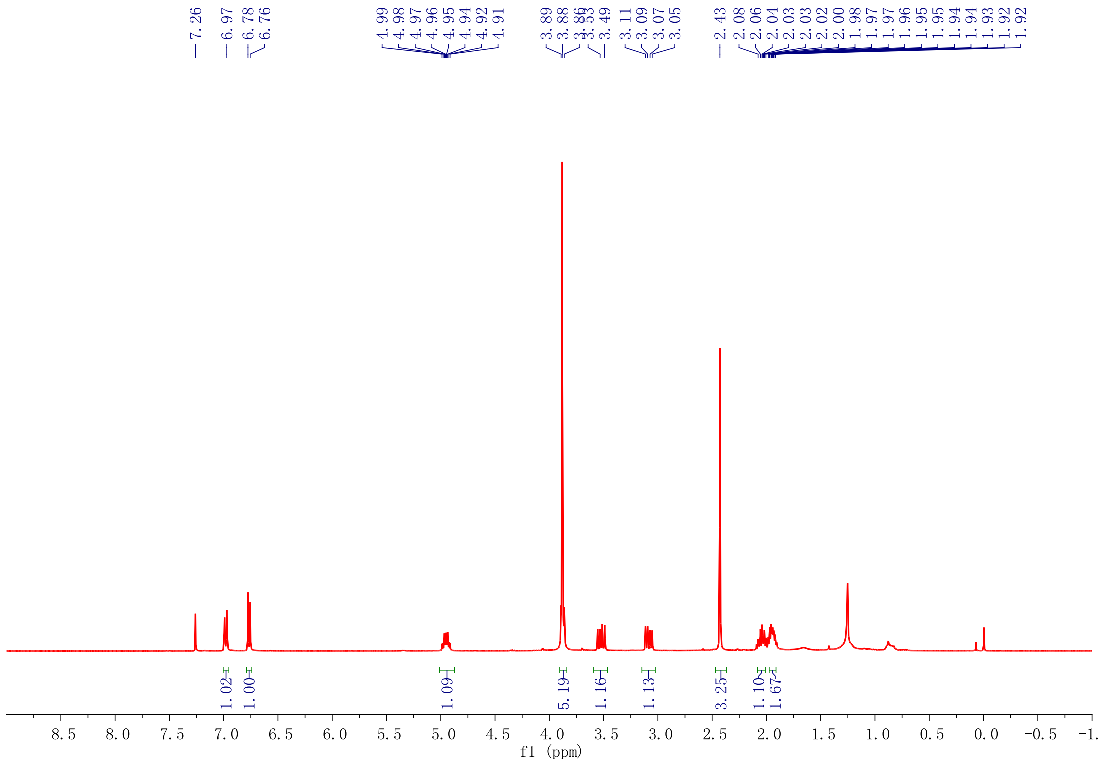

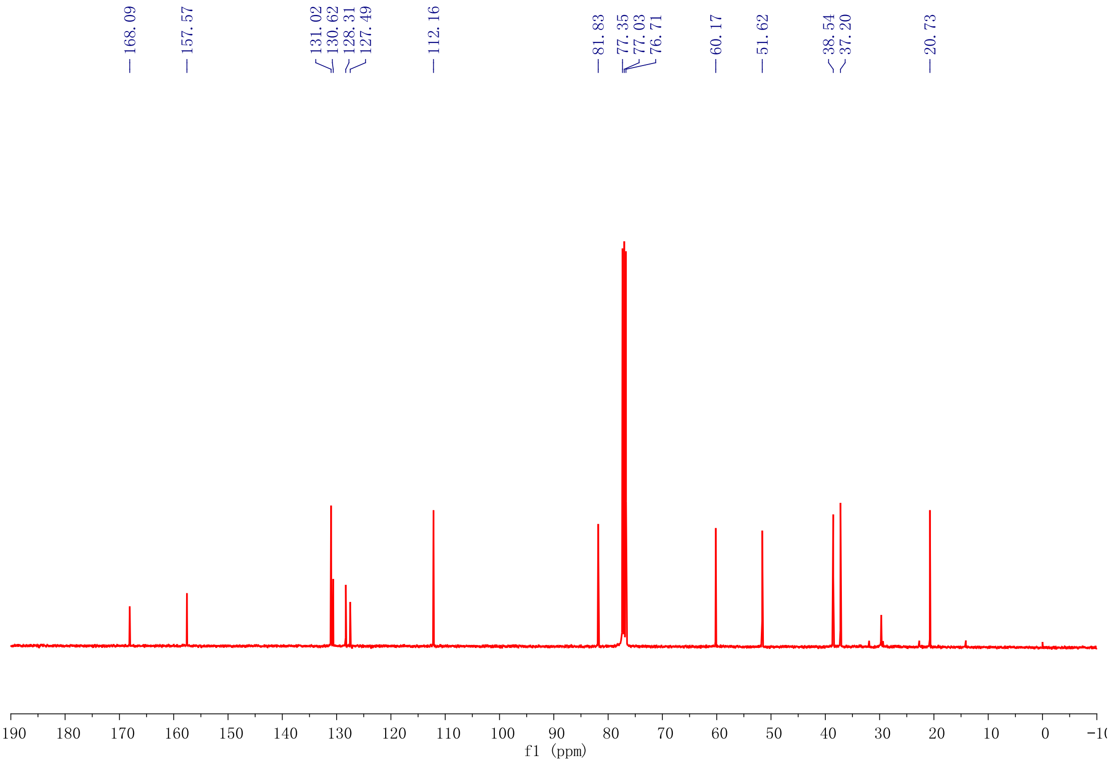

Supplementary Figure 63. ^{1}H, ^{13}C-NMR spectra of product **13**

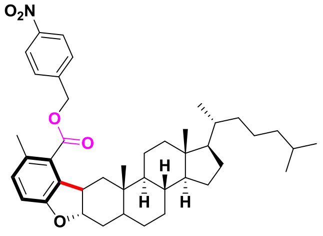

13

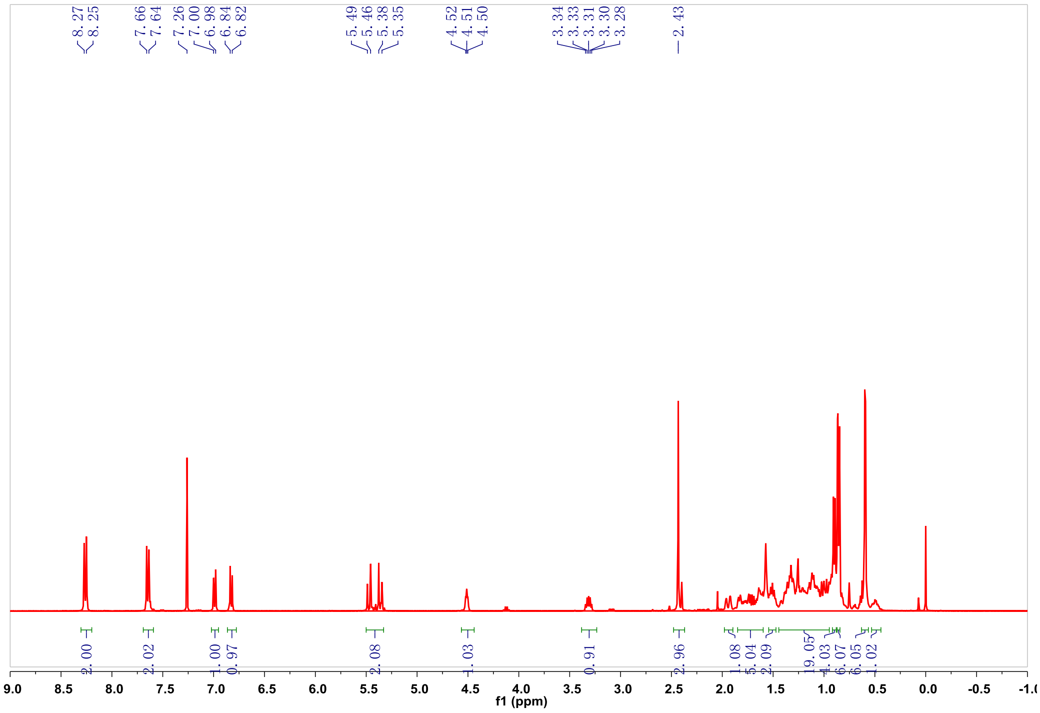

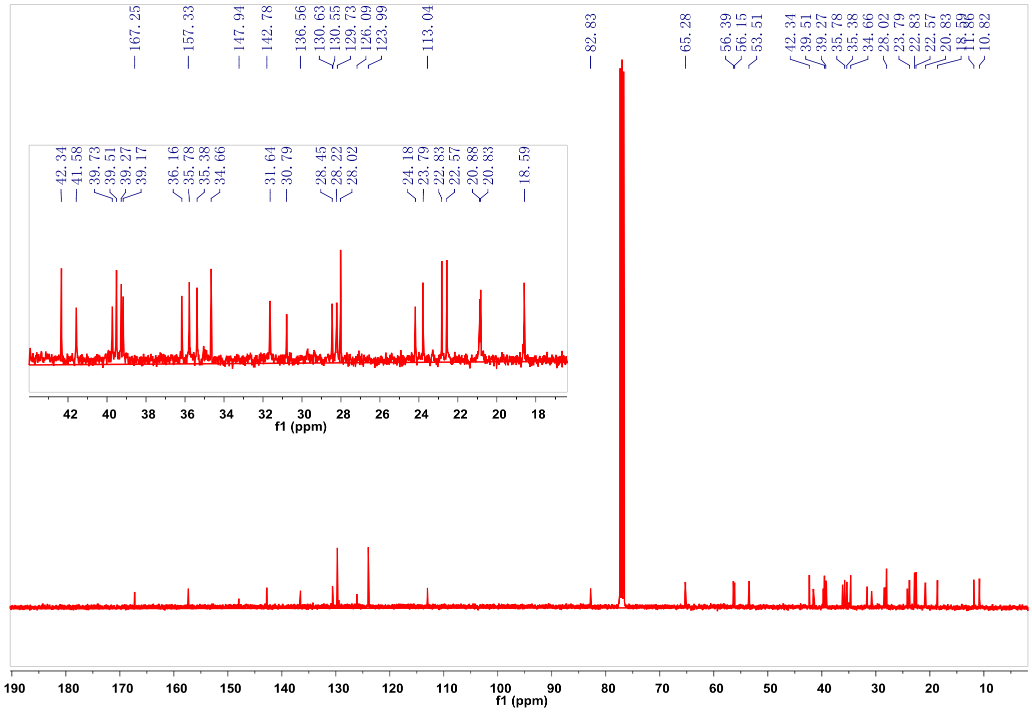

Supplementary Figure 64. ^{1}H, ^{13}C-NMR spectra of product **16**

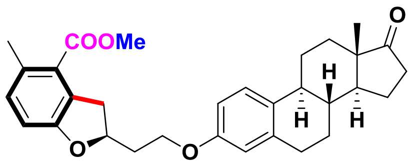

16

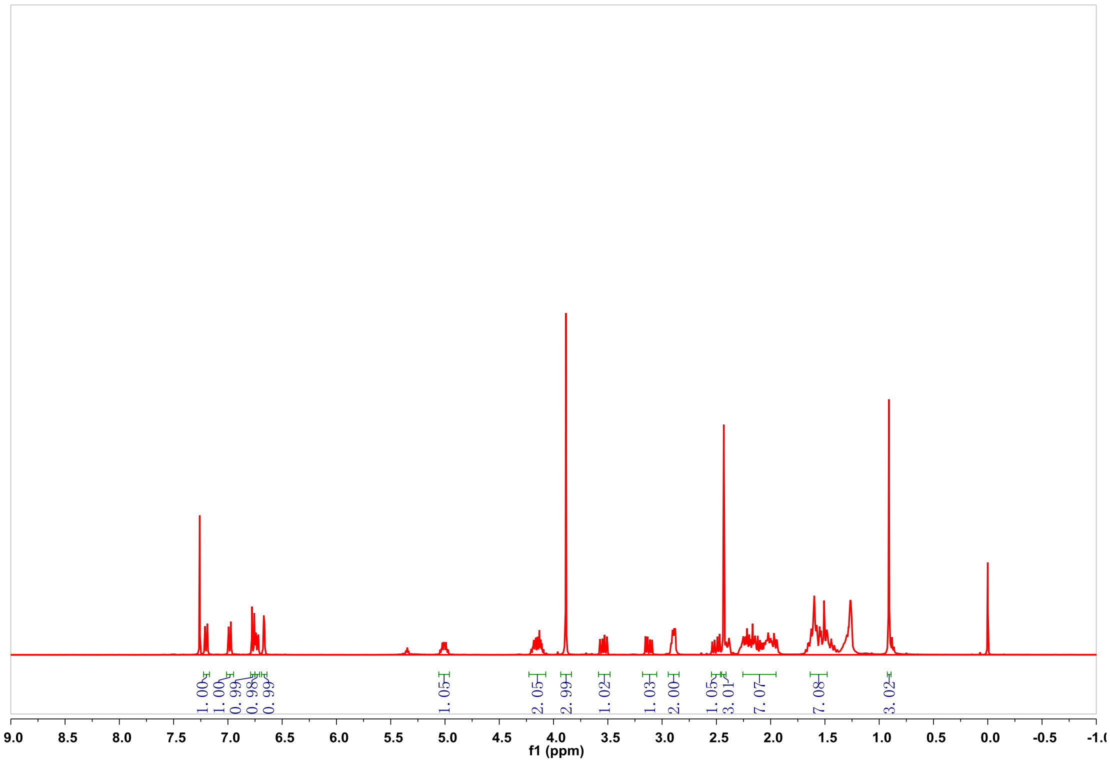

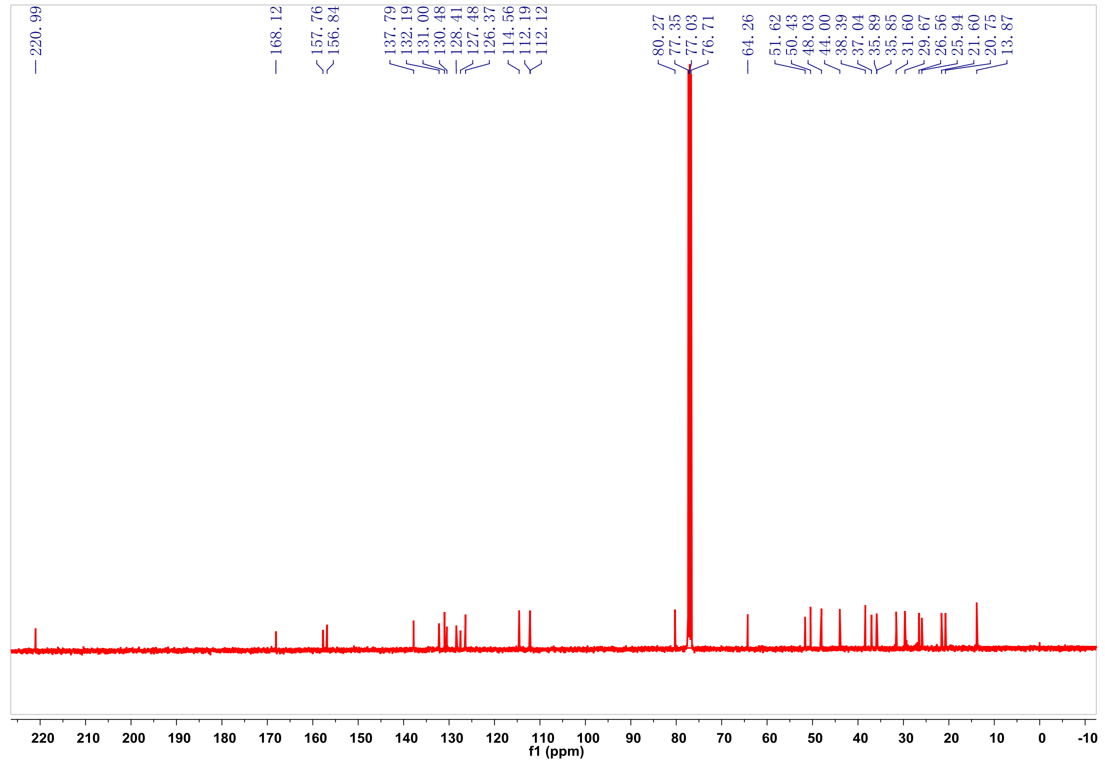

Supplementary Methods

General Information

Unless otherwise noted, materials and solvents were purchased from commercial suppliers and used without further purification. Analytical thin-layer chromatography (TLC) was performed on 0.25 mm silica gel 60-F254.. Visualization was performed using ultraviolet light (254 nm) and Vogel's permanganate. ^{1}H NMR data were obtained on Bruker-400 (400 MHz) spectrometer. Chemical shifts are reported in ppm with the tetramethylsilane (TMS) as the internal standard (TMS: 0.00 ppm). Data are reported as follows: chemical shift, integration, multiplicity (s = singlet, d = doublet, t = triplet, q = quartet, br = broad, m = multiplet), and coupling constants (Hz). ^{13}C NMR spectra were recorded on Bruker-400 (100 MHz) spectrometer with complete proton decoupling. Chemical shifts are reported in ppm with the solvent resonance as the internal standard ($CDCl_3$: 77.0 ppm). ESI-MS analysis was performed by Analytical Instrumentation Center, Peking University.

List of Abbreviations

BQ = 1, 4-Benzoquinone
DCM = dichloromethane
DDQ = 2, 3-Dichloro-5,6-dicyano-1,4-benzoquinone
DIAD = Diisopropyl azodicarboxylate
DMF = *N*,*N*-dimethylformamide
DMSO = Dimethyl sulfoxide
DPPA = Diphenylphosphoryl azide
EtOAc = ethyl acetate
MeCN = acetonitrile
^{t}AmylOH = 2-Methyl-2-butanol
TEA = triethylamine
THF = tetrahydrofuran
TBAF = Tetrabutylammonium fluoride
TIPSCl = Triisopropylsilyl Cloride
PE = petroleum ether

Starting Materials Preparation

General Procedure for the Synthesis of Compound 1a and 1y

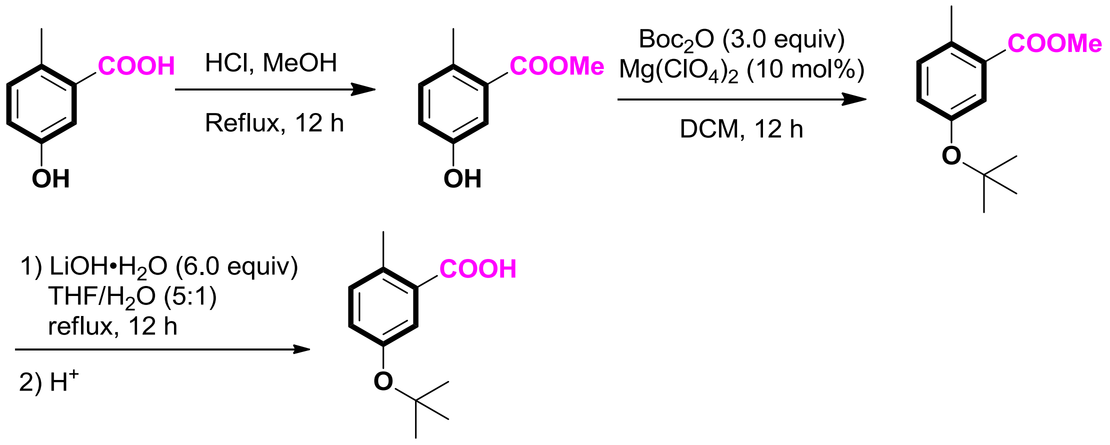

1a

Step 1: To a 100 mL round-bottom flask was added the 5-hydroxy-2-methylbenzoic acid (7.6 g, 50.0 mmol), HCl (5.0 mL), and MeOH (30.0 mL). The reaction mixture was refluxed overnight. After the reaction was completed, the solvent was removed under reduced pressure. Then the residue was purified by flash chromatography (EtOAc:PE = 1:8) to afford methyl 5-hydroxy-2-methylbenzoate (8.25 g, 99% isolated yield).

Step 2: Following a previously reported procedure[1], to a 100 mL round-bottom flask was added 5-hydroxy-2-methylbenzoate (3.32 g, 20.0 mmol), Boc_2O (21.0 mL, 4.6 equiv), $Mg(ClO_4)_2$ (893.0 mg, 0.2 equiv), and DCM (50.0 mL). The reaction mixture was refluxed for 12 h. After the reaction completed, the solvent was removed under reduced pressure. Then the reaction mixture was purified by flash chromatography (EtOAc:PE = 1:15) to afford methyl 5-(*tert*-butoxy)-2-methylbenzoate (3.35 g, 75% isolated yield).

Step 3: To a 100 mL round-bottom flask was added methyl 5-(tert-butoxy)-2-methylbenzoate (2.22 g, 10.0 mmol), $LiOH^{\cdot}H_2O$ (2.52 g, 6.0 equiv), and THF/H_2O (18.0 mL, 5:1). The reaction mixture was refluxed for 12 h, and then acidified with 2.0 N HCl (50 mL). The organic layer was separated and the aqueous layer was extracted with EtOAc (50.0 mL x 2), and dried over anhydrous Na_2SO_4. The solvent was removed under reduced pressure. Finally, the crude residue was purified by flash chromatography (MeOH:DCM = 1:50) to afford 5-(*tert*-butoxy)-2-methylbenzoic acid (**1a**, 2.0 g, 96% isolated yield).

Compound **1y** was prepared though the same procedure in smaller scales with 3,5-dihydroxybenzoic acid. The final carboxylic acid products were purified by column chromatography (MeOH:DCM = 1:50).

5-(tert-butoxy)-2-methylbenzoic acid (1a)

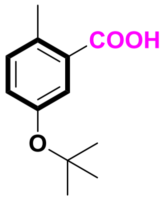

1a

^{1}H NMR (400 MHz, $CDCl_3$) δ 7.71 (d, *J* = 2.3 Hz, 1H), 7.17 (d, *J* = 8.3 Hz, 1H), 7.10 (dd, *J* = 8.2, 2.4 Hz, 1H), 2.60 (s, 3H), 1.35 (s, 9H). ^{13}C NMR (100 MHz, $CDCl_3$) δ 172.53, 153.10, 136.31, 132.43, 129.14, 128.50, 126.92, 78.90, 28.77, 21.46. HRMS (ESI) Calcd for $C_{12}H_{16}NaO_3$ $[M+H]^+$: 231.0995; found: 231.0992.

3, 5-di-tert-butoxybenzoic acid (1y)

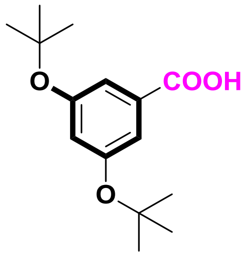

1y

^{1}H NMR (400 MHz, $CDCl_3$) δ 7.48 (d, *J* = 2.2 Hz, 2H), 6.89 (t, *J* = 2.2 Hz, 1H), 1.37 (d, *J* = 4.3 Hz, 18H). ^{13}C NMR (100 MHz, $CDCl_3$) δ 171.65, 155.98, 129.99, 125.67, 120.70, 79.40, 28.82. HRMS (ESI) Calcd for $C_{15}H_{23}O_4$ $[M+H]^+$: 267.1589; found: 267.1591.

General Procedure for the Synthesis of Compound 1b-c, 1e, 1l, 1r-s and 1u-x

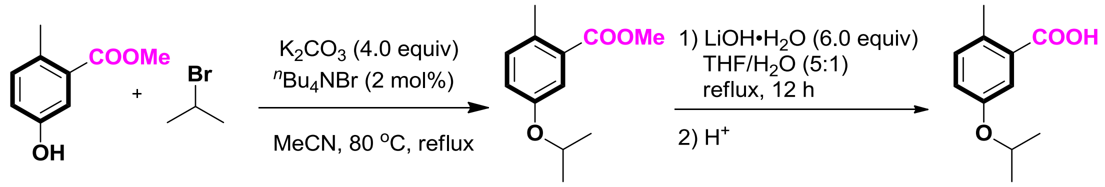

1b

Step 1: Following a previously reported procedure[2], to a 100 mL round-bottom flask was added methyl 5-hydroxy-2-methylbenzoate (1.66 g, 10.0 mmol), 2-bromopropane (1.23 g, 1.0 equiv), K_2CO_3 (5.53 g, 4.0 equiv), $^nBu_4NBr$ (65.0 mg, 2 mol%), and MeCN (20.0 mL). The reaction mixture was refluxed for 12 h. After the reaction completed, the solvent was removed under reduced pressure. Then the reaction mixture was purified by flash chromatography (EtOAc:PE = 1:10) to afford methyl 5-isopropoxy-2-methylbenzoate (1.85g, 89% isolated yield).

Step 2: To a 100 mL round-bottom flask was added 5-isopropoxy-2-methylbenzoate (0.416 g, 2.0 mmol), $LiOH{\cdot}H_2O$ (0.49 g, 6.0 equiv), and THF/H_2O (5.0 mL, 5:1). The reaction mixture was refluxed for 12 h, and then acidified with 2.0 N HCl (10.0 mL). The organic layer was separated and the aqueous layer was extracted with EtOAc (10.0 mL x 2). The combined organic layer dried over anhydrous Na_2SO_4. The solvent

was removed under reduced pressure. Finally, the crude residue was purified by flash chromatography (MeOH:DCM = 1:50) to afford 5-isopropoxy-2-methylbenzoic acid (**1b**, 0.38 g, 98% isolated yield).

Compound **1c, 1e, 1l, 1r-s and 1u-x** were prepared though the same procedure in smaller scales. The final carboxylic acid products were purified by column chromatography (MeOH:DCM = 1:50).

5-isopropoxy-2-methylbenzoic acid (1b)

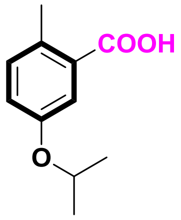

1b

^{1}H NMR (400 MHz, $CDCl_3$) δ 7.60 (d, J = 2.7 Hz, 1H), 7.16 (d, J = 8.4 Hz, 1H), 7.04 – 6.97 (m, 1H), 4.64 – 4.50 (m, 1H), 2.58 (s, 3H), 1.42 – 1.26 (m, 6H). ^{13}C NMR (100 MHz, $CDCl_3$) δ 173.17, 155.77, 133.29, 132.96, 128.91, 121.55, 118.06, 70.35, 21.99, 21.20. HRMS (ESI) Calcd for $C_{11}H_{15}O_3$ $[M+H]^+$: 195.1013; found: 195.1016.

5-(sec-butoxy)-2-methylbenzoic acid (1c)

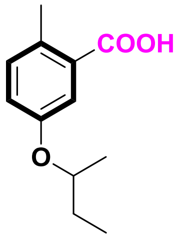

1c

^{1}H NMR (400 MHz, $CDCl_3$) δ 7.59 (d, J = 2.7 Hz, 1H), 7.16 (d, J = 8.4 Hz, 1H), 7.00 (dd, J = 8.4, 2.7 Hz, 1H), 4.37 – 4.25 (m, 1H), 2.54 (d, J = 25.0 Hz, 3H), 1.83 – 1.69 (m, 1H), 1.69 – 1.56 (m, 1H), 1.28 (t, J = 10.0 Hz, 3H), 0.98 (t, J = 7.4 Hz, 3H). ^{13}C NMR (100 MHz, $CDCl_3$) δ 172.36, 156.10, 133.20, 132.95, 128.78, 121.47, 118.05, 75.48, 29.14, 21.22, 19.19, 9.79. HRMS (ESI) Calcd for $C_{12}H_{17}O_3$ $[M+H]^+$: 209.1171; found: 209.1172.

2-methyl-5-(nonan-2-yloxy)benzoic acid (1e)

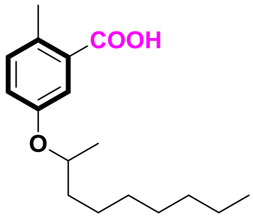

1e

^{1}H NMR (400 MHz, $CDCl_3$) δ 7.59 (d, J = 1.9 Hz, 1H), 7.16 (d, J = 8.4 Hz, 1H), 7.00 (dd, J = 8.3, 2.1 Hz, 1H), 4.36 (dt, J = 12.0, 6.0 Hz, 1H), 2.57 (s, 3H), 1.82 – 1.67 (m, 1H), 1.65 – 1.52 (m, 1H), 1.49 – 1.20 (m, 10H), 0.88 (t, J = 6.3 Hz, 3H). ^{13}C NMR

(100 MHz, $CDCl_3$) δ 172.65, 156.11, 133.18, 132.96, 128.83, 121.45, 118.02, 74.34, 36.43, 31.82, 29.58, 29.27, 25.53, 22.67, 21.22, 19.67, 14.12. HRMS (ESI) Calcd for $C_{17}H_{27}O_3$ $[M+H]^+$: 279.1951; found: 279.1955.

2-methyl-5-((4-phenylbutan-2-yl)oxy)benzoic acid (1l)

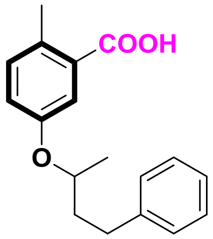

1l

^1H NMR (400 MHz, $CDCl_3$) δ 7.57 (d, J = 2.7 Hz, 1H), 7.27 (dd, J = 12.3, 5.1 Hz, 2H), 7.18 (q, J = 7.4 Hz, 4H), 6.97 (dd, J = 8.4, 2.7 Hz, 1H), 4.45 – 4.32 (m, 1H), 2.87 – 2.66 (m, 2H), 2.55 (d, J = 23.4 Hz, 3H), 2.07 (ddd, J = 13.6, 9.1, 6.8 Hz, 1H), 1.96 – 1.81 (m, 1H), 1.33 (d, J = 6.0 Hz, 3H). ^{13}C NMR (100 MHz, $CDCl_3$) δ 172.71, 155.96, 141.74, 133.32, 133.00, 128.90, 128.49, 128.45, 125.92, 121.33, 118.12, 73.26, 38.17, 31.78, 21.23, 19.64. HRMS (ESI) Calcd for $C_{18}H_{21}O_3$ $[M+H]^+$: 285.1478; found: 285.1485.

5-ethoxy-2-methylbenzoic acid (1r)

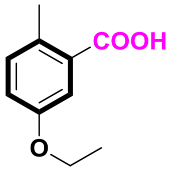

1r

^1H NMR (400 MHz, CDCl3) δ 7.60 (d, J = 2.5 Hz, 1H), 7.17 (d, J = 8.4 Hz, 1H), 7.02 (dd, J = 8.4, 2.8 Hz, 1H), 4.06 (q, J = 7.0 Hz, 2H), 2.58 (s, 3H), 1.43 (t, J = 7.0 Hz, 3H). ^{13}C NMR (100 MHz, $CDCl_3$) δ 173.41, 156.82, 133.33, 132.93, 128.88, 120.19, 116.37, 63.74, 21.23, 14.79. HRMS (ESI) Calcd for $C_{10}H_{13}O_3$ $[M+H]^+$: 181.0854; found: 181.0859.

5-(cyclohexyloxy)-2-methylbenzoic acid (1s)

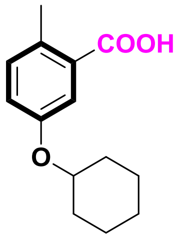

1s

^1H NMR (400 MHz, $CDCl_3$) δ 7.61 (d, J = 1.9 Hz, 1H), 7.19 – 7.13 (m, 1H), 7.02 (dd, J = 8.4, 2.7 Hz, 1H), 4.36 – 4.19 (m, 1H), 2.54 (d, J = 27.1 Hz, 3H), 2.06 – 1.91 (m, 2H), 1.89 – 1.74 (m, 2H), 1.65 – 1.46 (m, 3H), 1.46 – 1.25 (m, 3H). ^{13}C NMR (100 MHz, $CDCl_3$) δ 173.26, 155.65, 133.27, 132.92, 128.92, 121.71, 118.33, 75.82, 31.73, 25.62, 23.70, 21.22. HRMS (ESI) Calcd for $C_{14}H_{19}O_3$ $[M+H]^+$: 235.1322; found:

235.1329.

2-ethoxy-5-isopropoxybenzoic acid (1u)

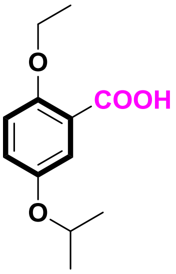

1u

^{1}H NMR (400 MHz, $CDCl_3$) δ 11.25 (s, 1H), 7.69 (d, *J* = 3.1 Hz, 1H), 7.08 (dd, *J* = 9.0, 3.1 Hz, 1H), 6.97 (d, *J* = 9.0 Hz, 1H), 4.60 – 4.50 (m, 1H), 4.29 (q, *J* = 7.0 Hz, 2H), 1.54 (t, *J* = 7.0 Hz, 3H), 1.32 (d, *J* = 6.0 Hz, 6H). ^{13}C NMR (100 MHz, $CDCl_3$) δ 165.38, 152.66, 151.49, 124.05, 118.67, 118.33, 114.35, 70.90, 66.58, 21.94, 14.77. HRMS (ESI) Calcd for $C_{12}H_{17}O_4$ $[M+H]^+$: 225.1126; found: 225.1121.

2, 5-diisopropoxybenzoic acid (1v)

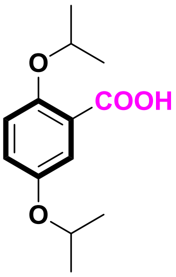

1v

^{1}H NMR (400 MHz, $CDCl_3$) δ 11.51 (s, 1H), 7.67 (d, *J* = 3.0 Hz, 1H), 7.08 (dd, *J* = 9.0, 3.0 Hz, 1H), 6.99 (d, *J* = 9.0 Hz, 1H), 4.77 (hept, *J* = 6.0 Hz, 1H), 4.55 (hept, *J* = 6.0 Hz, 1H), 1.46 (d, *J* = 6.0 Hz, 6H), 1.33 (d, *J* = 6.0 Hz, 6H). ^{13}C NMR (100 MHz, $CDCl_3$) δ 165.56, 152.69, 150.33, 124.11, 119.38, 118.20, 116.18, 74.72, 70.79, 22.07, 21.95. HRMS (ESI) Calcd for $C_{13}H_{19}O_4$ $[M+H]^+$: 239.1283; found: 239.1278.

2-fluoro-5-isopropoxybenzoic acid (1w)

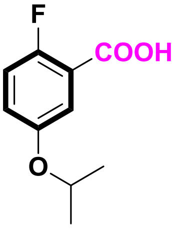

1w

^{1}H NMR (400 MHz, $CDCl_3$) δ 7.50 (dd, *J* = 10.4, 5.9 Hz, 1H), 7.08 (dd, *J* = 9.4, 6.1 Hz, 2H), 4.59 – 4.47 (m, 1H), 1.36 (t, *J* = 11.5 Hz, 6H). ^{13}C NMR (100 MHz, $CDCl_3$) δ 169.42, 169.38, 158.17, 155.64, 153.64, 153.62, 124.01, 123.93, 118.12, 118.01, 117.88, 117.58, 117.47, 71.08, 21.89. HRMS (ESI) Calcd for $C_{10}H_{12}FO_3$ $[M+H]^+$: 199.0761; found: 199.0765.

2-chloro-5-isopropoxybenzoic acid (1x)

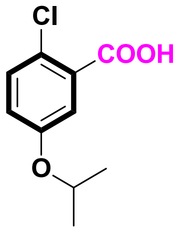

1x

^{1}H NMR (400 MHz, $CDCl_3$) δ 7.51 (d, J = 3.0 Hz, 1H), 7.36 (d, J = 8.8 Hz, 1H), 7.00 (dd, J = 8.8, 3.0 Hz, 1H), 4.62 – 4.50 (m, 1H), 1.35 (d, J = 6.0 Hz, 6H). ^{13}C NMR (100 MHz, $CDCl_3$) δ 170.13, 156.32, 132.32, 128.75, 125.63, 121.82, 118.80, 70.72, 21.85. HRMS (ESI) Calcd for $C_{10}H_{12}ClO_3$ $[M+H]^+$: 215.0469; found: 215.0469.

General Procedure for the Synthesis of Compound 1d, 1f, 1k, 1t and 1z

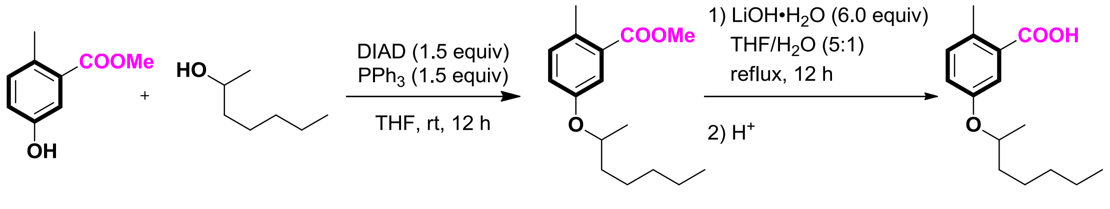

Step 1: Following a previously reported procedure[3], to a 20 mL oven-dried glass tube was added methyl 5-hydroxy-2-methylbenzoate (0.274 g, 1.65 mmol) , heptan-2-ol (0.192 g, 1.0 equiv), PPh_3 (0.649 g, 1.5 equiv) and THF (2.0 mL) under N_2 atmosphere. DIAD (0.5 g, 1.5 equiv) was added dropwisely to this solution at 0 ^{o}C. The reaction mixture warmed up to room temperature and stirred for 16 h. After the reaction completed, the solvent was removed under reduced pressure. Then the reaction mixture was purified by flash chromatography (EtOAc/PE = 1:10) to afford methyl 5-(heptan-2-yloxy)-2-methylbenzoate (0.4 g, 92% isolated yield).

Step 2: To a 100 mL round-bottom flask was added methyl 5-(heptan-2-yloxy)-2-methylbenzoate methyl (0.5 g, 1.5 mmol), $LiOH{\cdot}H_2O$ (0.377 g, 6.0 equiv), and THF/H_2O (5.0 mL, 5:1). The reaction mixture was refluxed for 12 h, and then acidified with 2.0 N HCl (10.0 mL). The organic layer was separated and the aqueous layer was extracted with EtOAc (10.0 mL x 2). The combined organic layer dried over anhydrous Na_2SO_4. The solvent was removed under reduced pressure. Finally, the crude residue was purified by flash chromatography (MeOH:DCM = 1:50) to afford 5-(heptan-2-yloxy)-2-methylbenzoic acid (**1d**, 0.356 g, 95% isolated yield).

Compound **1f, 1k, 1t and 1z** were prepared though the same procedure. The final carboxylic acid products were purified by column chromatography (MeOH:DCM = 1:50).

5-(heptan-2-yloxy)-2-methylbenzoic acid (1d)

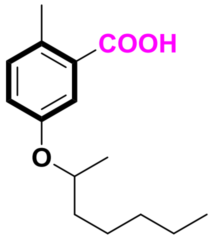

1d

^{1}H NMR (400 MHz, $CDCl_3$) δ 7.59 (d, *J* = 2.8 Hz, 1H), 7.15 (t, *J* = 6.8 Hz, 1H), 7.00 (dd, *J* = 8.4, 2.8 Hz, 1H), 4.44 – 4.29 (m, 1H), 2.56 (d, *J* = 11.6 Hz, 3H), 1.79 – 1.66 (m, 1H), 1.64 – 1.51 (m, 1H), 1.51 (s, 9H), 0.89 (dd, *J* = 9.0, 5.0 Hz, 3H). ^{13}C NMR (100 MHz, $CDCl_3$) δ 172.82, 156.12, 133.15, 132.93, 128.93, 121.43, 118.05, 74.36, 36.40, 31.80, 25.18, 22.61, 21.18, 19.66, 14.02.HRMS (ESI) Calcd for $C_{16}H_{25}O_3$ $[M+H]^+$: 265.18013; found: 265.1798.

2-methyl-5-((5-methylhexan-2-yl)oxy)benzoic acid (1f)

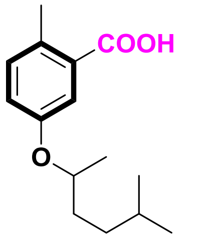

1f

^{1}H NMR (400 MHz, $CDCl_3$) δ 7.61 (d, *J* = 2.7 Hz, 1H), 7.17 (d, *J* = 8.4 Hz, 1H), 7.01 (dd, *J* = 8.4, 2.8 Hz, 1H), 4.35 (dt, *J* = 12.0, 6.0 Hz, 1H), 2.59 (s, 3H), 1.75 (tdd, *J* = 11.4, 6.3, 5.1 Hz, 1H), 1.68 – 1.51 (m, 2H), 1.45 – 1.18 (m, 5H), 0.91 (dd, *J* = 6.6, 1.7 Hz, 6H). ^{13}C NMR (100 MHz, $CDCl_3$) δ 173.42, 156.12, 133.21, 132.96, 128.96, 121.48, 118.10, 74.67, 34.63, 34.29, 28.07, 22.61, 22.59, 21.22, 19.68. HRMS (ESI) Calcd for $C_{16}H_{25}O_3$; $[M+H]^+$: 265.1804; found: 265.1798.

2-methyl-5-((1-phenylpropan-2-yl)oxy)benzoic acid (1k)

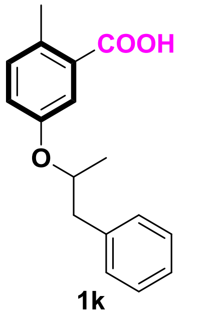

1k

^{1}H NMR (400 MHz, $CDCl_3$) δ 7.71 – 7.58 (m, 1H), 7.40 – 7.20 (m, 5H), 7.20 – 7.13 (m, 1H), 7.07 – 6.94 (m, 1H), 4.74 – 4.49 (m, 1H), 3.23 – 3.02 (m, 1H), 2.93 – 2.78 (m, 1H), 2.65 – 2.52 (m, 3H), 1.45 – 1.25 (m, 3H). ^{13}C NMR (100 MHz, $CDCl_3$) δ 173.32, 155.78, 138.06, 133.52, 133.03, 129.55, 129.00, 128.41, 126.45, 121.58, 118.19, 75.20, 42.63, 21.24, 19.36. HRMS (ESI) Calcd for $C_{18}H_{21}O_3$ $[M+H]^+$: 285.1482; found: 285.1485.

2-methyl-5-((4-methyl-1-phenylpentan-2-yl)oxy)benzoic acid (1t)

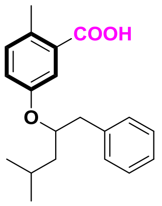

1t

^{1}H NMR (400 MHz, $CDCl_3$) δ 7.61 (d, *J* = 2.6 Hz, 1H), 7.31 – 7.24 (m, 2H), 7.24 – 7.18 (m, 3H), 7.14 (d, *J* = 8.4 Hz, 1H), 6.97 (dd, *J* = 8.4, 2.7 Hz, 1H), 4.52 (dd, *J* = 7.8, 5.1 Hz, 1H), 2.93 (dd, *J* = 34.9, 5.9 Hz, 2H), 2.57 (s, 3H), 1.88 – 1.74 (m, 1H), 1.64 (s, 1H), 1.34 (d, *J* = 6.1 Hz, 2H), 0.93 (d, *J* = 6.7 Hz, 3H), 0.84 (d, *J* = 6.6 Hz, 3H). ^{13}C NMR (100 MHz, $CDCl_3$) δ 172.78, 156.25, 137.99, 133.34, 133.02, 129.60, 128.95, 128.37, 126.36, 121.23, 118.05, 42.92, 40.51, 24.69, 23.19, 22.31, 21.19. HRMS (ESI) Calcd for $C_{20}H_{25}O_3$ $[M+H]^+$: 313.1800; found: 313.1798.

5-(((3R,5S,8R,9S,10S,13R,14S,17R)-10,13-dimethyl-17-((R)-6-methylheptan-2-yl) hexadecahydro-1H-cyclopenta[a]phenanthren-3-yl)oxy)-2-methylbenzoic acid (1z)

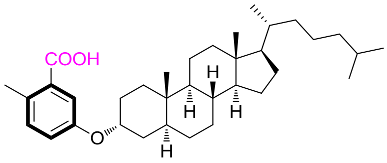

1z

^{1}H NMR (400 MHz, $CDCl_3$) δ 7.59 (d, *J* = 2.7 Hz, 1H), 7.16 (d, *J* = 8.5 Hz, 1H), 7.02 (dd, *J* = 8.4, 2.7 Hz, 1H), 4.57 – 4.52 (m, 1H), 2.57 (s, 3H), 1.99 – 1.78 (m, 3H), 1.68 – 1.44 (m, 9H), 1.39 – 0.97 (m, 19H), 0.92 – 0.80 (m, 13H), 0.66 (s, 3H). ^{13}C NMR (100 MHz, $CDCl_3$) δ 172.78, 155.62, 133.01, 132.89, 128.92, 121.49, 118.46, 72.61, 56.54, 56.30, 54.18, 42.62, 40.05, 39.58, 39.53, 36.20, 35.84, 35.82, 35.53, 32.67, 32.62, 31.97, 28.49, 28.28, 28.03, 25.56, 24.20, 23.89, 22.84, 22.58, 21.21, 20.83, 18.69, 12.10, 11.46. HRMS (ESI) Calcd for $C_{35}H_{55}O_3$ $[M+H]^+$: 523.4151; found: 523.4141.

General Procedure for the Synthesis of Compound 1m-q

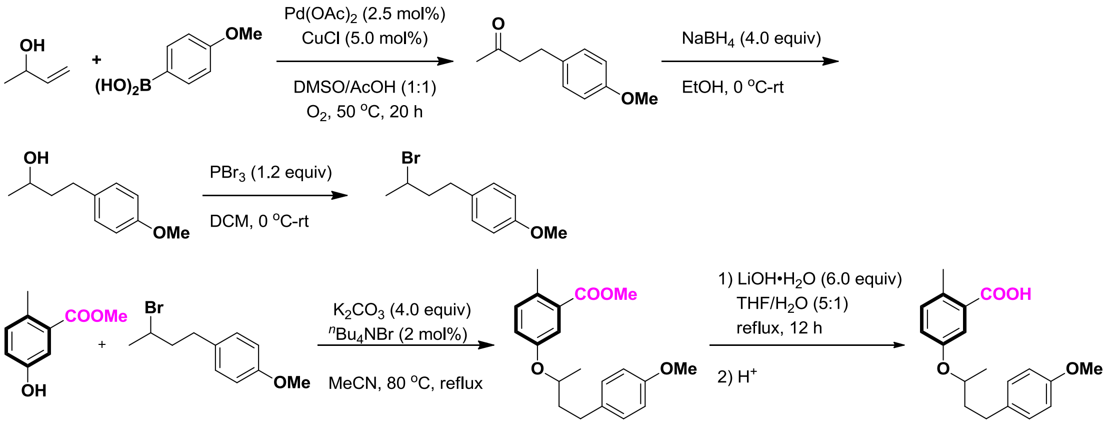

Step 1: Following a previously reported procedure[4], to a 20 mL oven-dried glass tube was added methyl (4-methoxyphenyl)boronic acid (1.52 g, 10.0 mmol) , but-3-en-2-ol

(0.865 g, 1.2 equiv), $Pd(OAc)_2$ (56 mg, 2.5 mol%), CuCl (50 mg, 5 mol%) and solvent (DMSO/AcOH = 1:1, v/v, 2.0 mL). Subsequently, the tube was purged with a balloon pressure of oxygen for 3 times. The reaction mixture was stirred at 50 ^{o}C for 20 h. After the reaction completed, the solution was diluted with 5 mL saturated NaCl solution. The mixture was extracted with Et_2O (150 mL), washed with saturated $NaHCO_3$ (3 x 50 mL), saturated NaCl (2 x 10 mL) and dried over anhydrous Na_2SO_4. The solvent was removed under reduced pressure. Then the reaction mixture was purified by flash chromatography (EtOAc/PE = 1:10) to afford 4-(4-methoxyphenyl)butan-2-one (1.62 g, 91% isolated yield).

Step 2: To a 50 mL round-bottom flask was added 4-(4-methoxyphenyl)butan-2-one (0.891 mg, 5.0 mmol) and EtOH (10 mL). $NaBH_4$ (0.756 g, 4.0 equiv) was slowly added to the mixture at 0 ℃. The reaction mixture was warmed up slowly to room temperature and stirred for 4 h. After the reaction was completed, the reaction was quenched with saturated NH_4Cl (10.0 mL). The mixture was extracted with DCM (3 x 10 mL) and dried over anhydrous Na_2SO_4. The solvent was removed under reduced pressure. The crude product was obtained and carried on to the next synthetic transformation without purification.

Step 3: To a 50 mL round-bottom flask was added 4-(4-methoxyphenyl)butan-2-ol (5.0 mmol based on a theoretical yield of 100%) and DCM (10 mL). PBr_3 (1.62 g, 1.2 equiv) was added dropwisely to this solution at 0 ℃. The reaction mixture was warmed up slowly to room temperature and stirred for 5 h. After the reaction was completed, the reaction was quenched with saturated $NaHCO_3$ (10.0 mL). The mixture was extracted with Et_2O (3 x 10 mL) and dried over anhydrous Na_2SO_4. The solvent was removed under reduced pressure. The crude product was obtained and carried on to the next synthetic transformation without purification.

Step 4: The etherification and hydrogenation were carried out *via* the procedure described above to afford the compound **1m** (0.65g, 41% isolated yield over four steps).

Compound **1n-q** were prepared though the same procedure with corresponding secondary alcohols. The final carboxylic acid products were purified by column chromatography (MeOH:DCM = 1:50).

5-((4-(4-methoxyphenyl)butan-2-yl)oxy)-2-methylbenzoic acid (1m)

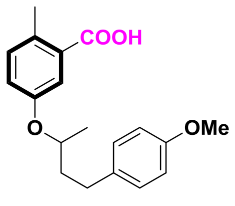

1m

^{1}H NMR (400 MHz, $CDCl_3$) δ 7.59 (d, J = 5.3 Hz, 1H), 7.17 (d, J = 8.4 Hz, 1H), 7.14 – 7.06 (m, 2H), 7.02 – 6.95 (m, 1H), 6.89 – 6.80 (m, 2H), 4.38 (d, J = 5.1 Hz, 1H), 3.80 (s, 3H), 2.83 – 2.63 (m, 2H), 2.59 (d, J = 4.3 Hz, 3H), 2.07 (dd, J = 14.9, 11.5 Hz, 1H), 1.88 (t, J = 9.8 Hz, 1H), 1.32 (dd, J = 11.3, 7.6 Hz, 3H). ^{13}C NMR (100 MHz, $CDCl_3$) δ 173.30, 172.86, 157.83, 156.00, 133.76, 133.32, 133.01, 129.38, 128.99, 128.92, 121.39, 118.16, 113.87, 73.23, 55.28, 38.40, 30.85, 21.26, 19.65. HRMS (ESI) Calcd for $C_{19}H_{23}O_4$ $[M+H]^+$: 315.1582; found: 315.1591.

5-((4-(4-fluorophenyl)butan-2-yl)oxy)-2-methylbenzoic acid (1n)

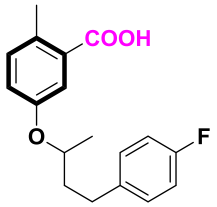

1n

^{1}H NMR (400 MHz, $CDCl_3$) δ 12.14 (s, 1H), 7.57 (d, *J* = 2.8 Hz, 1H), 7.20 – 7.08 (m, 3H), 7.01 – 6.89 (m, 3H), 4.44 – 4.28 (m, 1H), 2.82 – 2.66 (m, 2H), 2.58 (s, 3H), 2.11 – 1.96 (m, 1H), 1.91 – 1.81 (m, 1H), 1.32 (d, *J* = 6.1 Hz, 3H). ^{13}C NMR (100 MHz, $CDCl_3$) δ 172.75, 161.31 (d, *J* = 243.4 Hz), 155.90, 137.29 (d, *J* = 3.2 Hz), 133.41, 133.03, 129.78 (d, *J* = 7.8 Hz), 128.92, 121.33, 118.05, 115.16 (d, *J* = 21.1 Hz), 73.09, 38.28, 30.96, 21.22, 19.63. HRMS (ESI) Calcd for $C_{18}H_{20}FO_3$ $[M+H]^+$: 303.1387; found: 303.1391.

5-((4-(4-chlorophenyl)butan-2-yl)oxy)-2-methylbenzoic acid (1o)

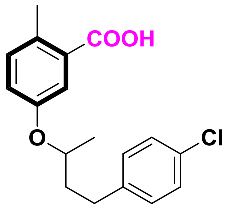

1o

^{1}H NMR (400 MHz, $CDCl_3$) δ 12.17 (s, 1H), 7.58 (s, 1H), 7.24 (d, *J* = 7.4 Hz, 2H), 7.16 (d, *J* = 7.6 Hz, 1H), 7.10 (d, *J* = 6.9 Hz, 2H), 6.97 (d, *J* = 6.5 Hz, 1H), 4.36 (s, 1H), 2.84 – 2.66 (m, 2H), 2.59 (s, 3H), 2.03 (s, 1H), 1.87 (s, 1H), 1.32 (d, *J* = 4.5 Hz, 3H). ^{13}C NMR (100 MHz, $CDCl_3$) δ 173.31, 155.88, 140.17, 133.49, 133.08, 131.64, 129.85, 129.01, 128.55, 121.37, 118.07, 73.07, 38.08, 31.14, 21.29, 19.64. HRMS (ESI) Calcd for $C_{18}H_{20}ClO_3$ $[M+H]^+$: 319.1100; found: 319.1095.

5-((4-(4-bromophenyl)butan-2-yl)oxy)-2-methylbenzoic acid (1p)

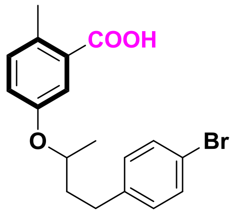

1p

^{1}H NMR (400 MHz, $CDCl_3$) δ 11.75 (s, 1H), 7.56 (d, *J* = 2.8 Hz, 1H), 7.39 (d, *J* = 8.3 Hz, 2H), 7.17 (d, *J* = 8.4 Hz, 1H), 7.05 (d, *J* = 8.3 Hz, 2H), 6.97 (dd, *J* = 8.4, 2.8 Hz, 1H), 4.41 – 4.30 (m, 1H), 2.80 – 2.66 (m, 2H), 2.58 (s, 3H), 2.08 – 2.00 (m, 1H), 1.90 – 1.82 (m, 1H), 1.32 (d, *J* = 6.1 Hz, 3H). ^{13}C NMR (100 MHz, $CDCl_3$) δ 172.98, 155.86, 140.69, 133.48, 133.06, 131.49, 130.26, 128.95, 121.34, 119.66, 118.07, 73.06, 38.02, 31.21, 21.27, 19.64. HRMS (ESI) Calcd for $C_{18}H_{20}BrO_3$ $[M+H]^+$: 363.0581; found: 363.0590.

2-methyl-5-((4-(4-(trifluoromethyl)phenyl)butan-2-yl)oxy)benzoic acid (1q)

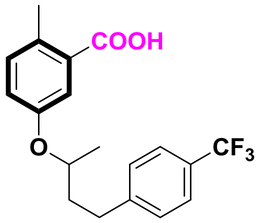

1q

^{1}H NMR (400 MHz, $CDCl_3$) δ 7.57 (d, J = 1.7 Hz, 1H), 7.53 (d, J = 7.9 Hz, 2H), 7.28 (d, J = 7.8 Hz, 2H), 7.17 (d, J = 8.3 Hz, 1H), 7.01 – 6.94 (m, 1H), 4.48 – 4.28 (m, 1H), 2.91 – 2.75 (m, 2H), 2.59 (s, 3H), 2.14 – 2.01 (m, 1H), 1.96 – 1.85 (m, 1H), 1.33 (d, J = 5.9 Hz, 3H). ^{13}C NMR (100 MHz, $CDCl_3$) δ 173.27, 155.83, 145.90, 133.57, 133.09, 129.02, 128.79, 128.32 (q, J = 32.3 Hz), 125.37 (q, J = 3.7 Hz), 124.36 (q, J = 271.7 Hz), 121.40, 118.04, 73.09, 37.87, 31.64, 21.24, 19.62. HRMS (ESI) Calcd for $C_{19}H_{19}F_3NaO_3$ $[M+Na]^+$: 375.1189; found: 375.1179.

General Procedure for the Synthesis of Compound 1i

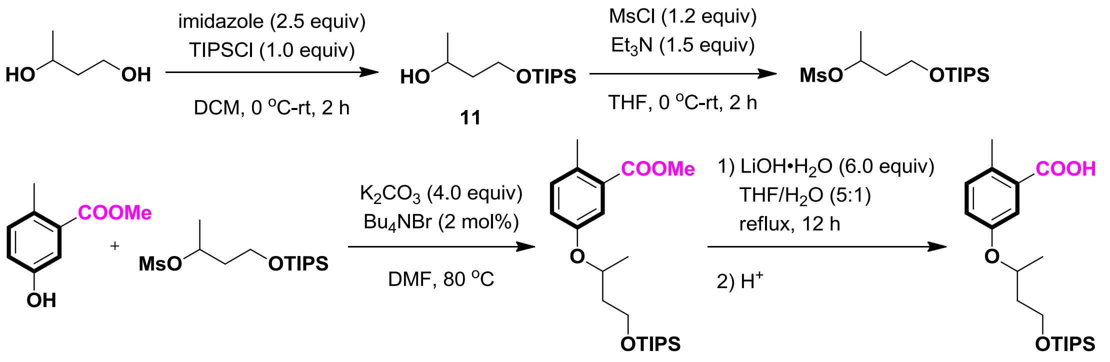

1i

Step 1: To a 100 mL round-bottom flask was added 1,3-Butanediol (1.0 g, 11.0 mmol), imidazol (1.88 g, 2.5 equiv), and DCM (10.0 mL). TIPSCl (2.1 g, 1.0 equiv) was added dropwisely to this solution at 0 ℃. The reaction mixture warmed up to room temperature and stirred for 2 h. After the reaction completed, the crude reaction mixture was diluted with saturated brine (50.0 mL). The organic layer was separated and the aqueous layer was washed with EtOAc (30 mL x 2). The combined organic layer dried over anhydrous Na_2SO_4. The solvent was removed under reduced pressure. Finally, the crude residue was purified by flash chromatography (EtOAc:PE = 1:10) to afford 4-((triisopropylsilyl)oxy)butan-2-ol (**11**, 2.52 g, 92% isolated yield).
Step 2: To a 100 mL round-bottom flask was added **11** (1.0 g, 4.0 mmol), Et_3N(0.5 g, 1.5 equiv), and THF (80.0 mL). MsCl (0.7 g, 1.2 equiv) was added dropwisely to this solution at 0 ℃. The reaction mixture warmed up to room temperature and stirred for 2 h. After the reaction completed, the crude reaction mixture was diluted with saturated brine (50.0 mL). The organic layer was separated and the aqueous layer was washed with EtOAc (30 mL x 2). The combined organic layer dried over anhydrous Na_2SO_4. The solvent was removed under reduced pressure. The crude product was obtained and carried on to the next synthetic transformation without purification.
Step 3: To a 100 mL round-bottom flask was added

4-((triisopropylsilyl)oxy)butan-2-yl methanesulfonate (4.0 mmol based on a theoretical yield of 100%), methyl 5-hydroxy-2-methylbenzoate (0.664 g, 4.0 mmol), K_2CO_3 (2.21 g, 4.0 equiv), ^{n}Bu_4NBr (26.0 mg, 2 mol%), and DMF (8.0 mL). The reaction mixture was refluxed for 12 h. After the reaction completed, the crude reaction mixture was diluted with water (10.0 mL). The organic mixture was extracted with Et_2O (20 mL x 2). The combined organic layer dried over anhydrous Na_2SO_4. The solvent was removed under reduced pressure. Then the reaction mixture was purified by flash chromatography (EtOAc:PE = 1:30) to afford methyl 2-methyl-5-((4-((triisopropylsilyl)oxy)butan-2-yl)oxy)benzoate (1.5g, 95% isolated yield over two steps).

Step 4: The hydrogenation was carried out *via* the procedure described above to afford the compound **1i** (0.833g, 86% isolated yield based on 1.0 g (2.53 mmol) carboxylic ester).

2-methyl-5-((4-((triisopropylsilyl)oxy)butan-2-yl)oxy)benzoic acid (1i)

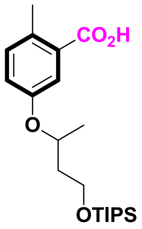

1i

^{1}H NMR (400 MHz, $CDCl_3$) δ 7.60 (d, J = 2.3 Hz, 1H), 7.15 (d, J = 8.4 Hz, 1H), 7.03 (dd, J = 8.4, 2.7 Hz, 1H), 4.64 (dd, J = 12.3, 6.2 Hz, 1H), 3.92 – 3.75 (m, 2H), 2.56 (s, 3H), 2.17 (s, 2H), 1.33 (d, J = 6.1 Hz, 3H), 1.16 – 0.96 (m, 21H); ^{13}C NMR (100 MHz, $CDCl_3$) δ 173.45, 156.14, 133.17, 132.87, 129.05, 121.22, 118.44, 71.20, 59.77, 39.74, 21.22, 19.90, 18.02, 11.98. HRMS (ESI) Calcd for $C_{21}H_{37}O_4Si$ $[M+H]^+$: 381.2449; found: 381.2456.

General Procedure for the Synthesis of Compound 1g

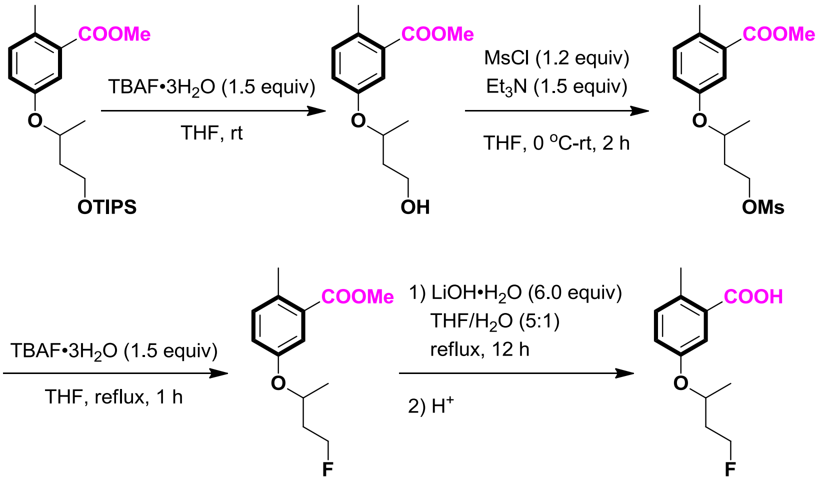

1g

Step 1: To a 100 mL round-bottom flask was added methyl 2-methyl-5-((4-((triisopropylsilyl)oxy)butan-2-yl)oxy)benzoate (2.36 g, 6.0 mmol), TBAF·$3H_2O$ (2.35 g, 1.5 equiv) and THF (12.0 mL). The reaction mixture was stirred

at room temperature for 3 h. After the reaction completed, the solvent was removed under reduced pressure. Then the reaction mixture was purified by flash chromatography (EtOAc:PE = 1:3) to afford methyl 5-((4-hydroxybutan-2-yl)oxy)-2-methylbenzoate (1.3g, 91% isolated yield).

Step 2: The methanesulfonylation of methyl 5-((4-hydroxybutan-2-yl)oxy)-2-methylbenzoate (0.714 g, 3.0 mmol) was carried out *via* the procedure described above to afford the product and carried on to the next synthetic transformation without purification.

Step 3: To a 100 mL round-bottom flask was added methyl 2-methyl-5-((4-((methylsulfonyl)oxy)butan-2-yl)oxy)benzoate (3.0 mmol based on a theoretical yield of 100%), TBAF·$3H_2O$ (1.4 g, 1.5 equiv) and THF (12.0 mL). The reaction mixture was refluxed for 3 h. After the reaction completed, the solvent was removed under reduced pressure. Then the reaction mixture was purified by flash chromatography (EtOAc:PE = 1:30) to afford methyl 5-((4-fluorobutan-2-yl)oxy)-2-methylbenzoate (0.32 g, 44% isolated yield over two steps).

Step 4: The hydrogenation was carried out *via* the procedure described above to afford the compound **1g** (0.28 g, 93% isolated yield).

5-((4-acetoxybutan-2-yl)oxy)-2-methylbenzoic acid (1g)

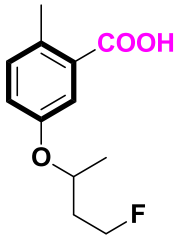

1g

^{1}H NMR (400 MHz, $CDCl_3$) δ 7.62 (d, *J* = 2.6 Hz, 1H), 7.18 (d, *J* = 8.4 Hz, 1H), 7.02 (dd, *J* = 8.4, 2.8 Hz, 1H), 4.75 – 4.49 (m, 3H), 2.58 (s, 3H), 2.19 – 1.91 (m, 2H), 1.35 (dd, *J* = 8.8, 6.2 Hz, 3H). ^{13}C NMR (100 MHz, $CDCl_3$) δ 173.32, 155.76, 133.72, 133.07, 129.09, 121.38, 118.36, 80.70 (d, *J* = 163.9 Hz), 70.58 (d, *J* = 4.3 Hz), 37.42 (d, *J* = 19.5 Hz), 21.23, 19.77. HRMS (ESI) Calcd for $C_{12}H_{16}FO_3$ $[M+H]^+$: 227.1073; found: 227.1078.

General Procedure for the Synthesis of Compound 1h

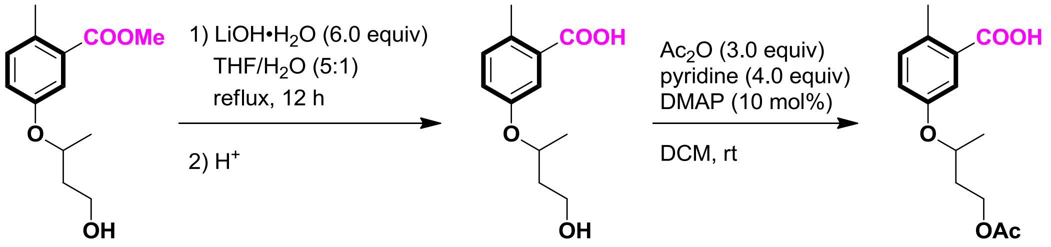

1h

Step 1: The hydrogenation was carried out *via* the procedure described above to afford the 5-((4-hydroxybutan-2-yl)oxy)-2-methylbenzoic acid.

Step 2: To a 20 mL oven-dried glass tube was added 5-((4-hydroxybutan-2-yl)oxy)-2-methylbenzoic acid (1.2 g, 5.35 mmol), pyridine (1.64 g, 4.0 equiv) and DMAP (65 mg, 10 mol%). Ac_2O (1.64 g, 3.0 equiv) was added dropwisely to this solution at room temperature. The reaction mixture was stirred for 3 h. After the reaction completed, the reaction was diluted with 2.0 N HCl (10 mL). The organic layer was separated and the aqueous layer was washed with DCM (30 mL x 2). The combined organic layer dried over anhydrous Na_2SO_4. The solvent was removed under reduced pressure. Finally, the crude residue was purified by flash chromatography (MeOH:DCM = 1:20) to afford 5-((4-acetoxybutan-2-yl)oxy)-2-methylbenzoic acid (**1h**, 0.36 g, 25% isolated yield).

5-((4-acetoxybutan-2-yl)oxy)-2-methylbenzoic acid (1h)

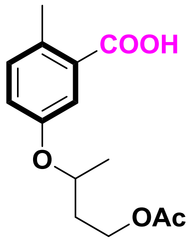

1h

^{1}H NMR (400 MHz, $CDCl_3$) δ 7.60 (d, *J* = 2.6 Hz, 1H), 7.17 (d, *J* = 8.4 Hz, 1H), 7.00 (dd, *J* = 8.4, 2.6 Hz, 1H), 4.53 (dq, *J* = 12.4, 6.0 Hz, 1H), 4.33 – 4.14 (m, 2H), 2.58 (s, 3H), 2.15 – 2.01 (m, 4H), 1.94 (dt, *J* = 13.7, 6.4 Hz, 1H), 1.34 (d, *J* = 6.1 Hz, 3H). ^{13}C NMR (100 MHz, $CDCl_3$) δ 173.09, 171.23, 155.73, 133.66, 133.08, 128.98, 121.57, 117.85, 71.10, 61.18, 35.51, 21.26, 20.99, 19.76. HRMS (ESI) Calcd for $C_{14}H_{19}O_5$ $[M+H]^+$: 267.1222; found: 267.1227.

General Procedure for the Synthesis of Compound 1j

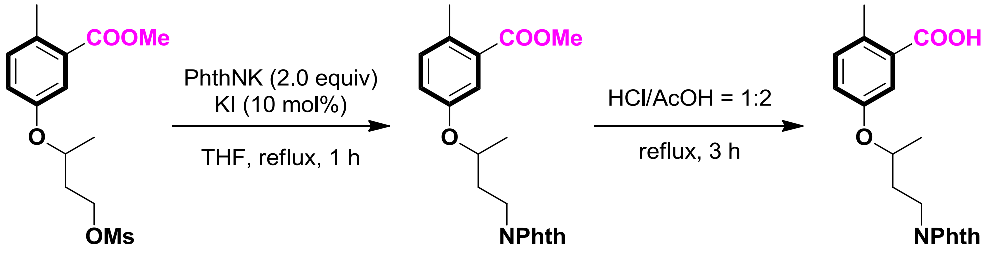

1j

Step 1: To a 100 mL round-bottom flask was added methyl 2-methyl-5-((4-((methylsulfonyl)oxy)butan-2-yl)oxy)benzoate (1.38 g, 4.36 mmol), PhthNK (1.62 g, 2.0 equiv), KI (72 mg, 10 mol%) and DMF (20.0 mL). The reaction mixture was stirred at 80 ^oC for 3 h. After the reaction completed, the solvent was removed under reduced pressure. Then the reaction mixture was purified by flash chromatography (EtOAc:PE = 1:4) to afford methyl 5-((4-(1,3-dioxoisoindolin-2-yl)butan-2-yl)oxy)-2-methylbenzoate (1.27 g, 79% isolated yield).

Step 2: To a 50 mL round-bottom flask was added methyl 5-((4-(1,3-dioxoisoindolin-2-yl)butan-2-yl)oxy)-2-methylbenzoate (1.27 g, 3.46 mmol), HCl (5.0 mL) and AcOH (10.0 mL). The reaction mixture was refluxed for 3

h. After the reaction completed, the mixture was poured into water (70 mL). The precipitated product was collected by filtration and washed with water. Then the crude product was purified by flash chromatography (MeOH:DCM = 1:20) to afford 5-((4-(1,3-dioxoisoindolin-2-yl)butan-2-yl)oxy)-2-methylbenzoic acid (**1j**, 0.63 g, 50% isolated yield).

5-((4-(1,3-dioxoisoindolin-2-yl)butan-2-yl)oxy)-2-methylbenzoic acid (1j)

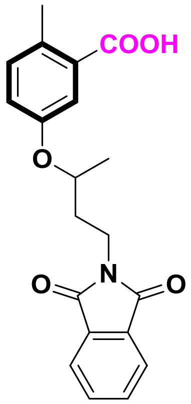

1j

^{1}H NMR (400 MHz, $CDCl_3$) δ 7.86 – 7.78 (m, 2H), 7.73 – 7.66 (m, 2H), 7.49 (d, *J* = 2.2 Hz, 1H), 7.12 (d, *J* = 8.4 Hz, 1H), 6.94 (dd, *J* = 8.2, 2.1 Hz, 1H), 4.59 – 4.39 (m, 1H), 3.97 – 3.82 (m, 2H), 2.56 (s, 3H), 2.19 – 2.10 (m, 1H), 2.05 – 1.95 (m, 1H), 1.35 (d, *J* = 6.0 Hz, 3H). ^{13}C NMR (100 MHz, $CDCl_3$) δ 172.80, 168.41, 155.49, 133.94, 133.48, 132.95, 132.13, 128.90, 123.25, 121.21, 118.18, 72.18, 35.02, 34.95, 21.25, 19.50. HRMS (ESI) Calcd for $C_{20}H_{20}NO_5$ $[M+H]^+$: 354.1330; found: 354.1336.

The synthesis of chiral substrates

General Procedure for the Synthesis of *R*-1i (path 1)

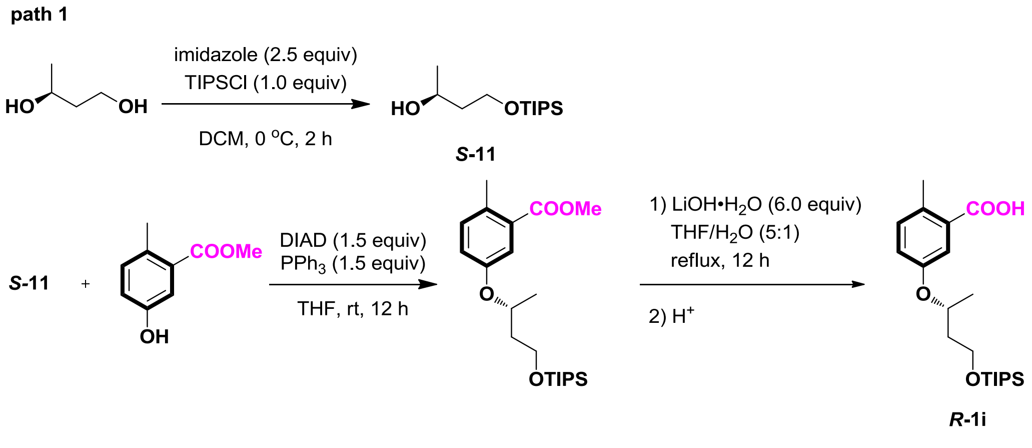

Step 1: The synthesis of ***S*-11** was carried out *via* the procedure described above.
Step 2: The etherification and hydrogenation were carried out *via* the procedure described above to afford the compound ***R*-1i**.

General Procedure for the Synthesis of *S*-1i (path 2)

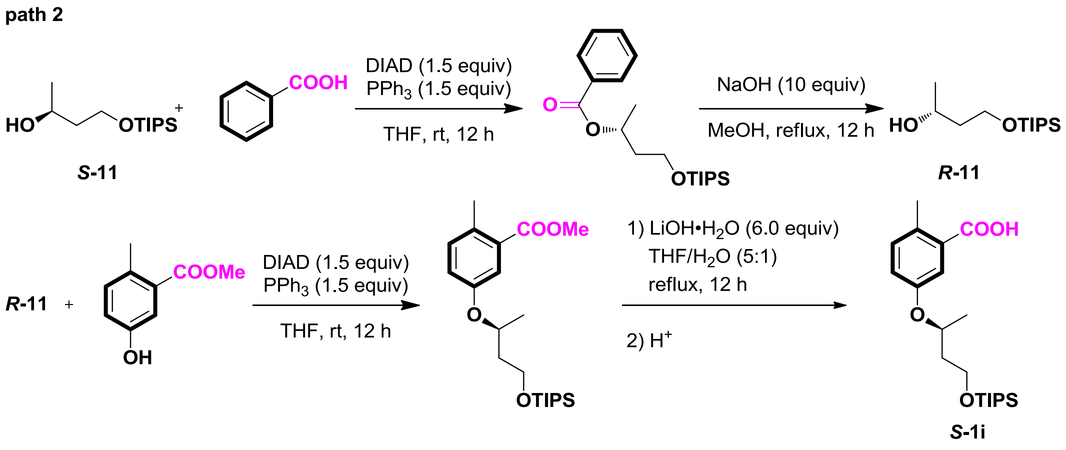

Step 1: The etherification was carried out *via* the mitsunobu reaction procedure described above to afford the compound (*R*)-4-((triisopropylsilyl)oxy)butan-2-yl benzoate.

Step2: To a 50 mL oven-dried glass tube was added (*R*)-4-((triisopropylsilyl)oxy)butan-2-yl benzoate (1.05 g, 3.0 mmol), NaOH (1.2 g, 10.0 equiv) and MeOH (30 mL). The reaction mixture was refluxed for 12 h. The organic layer was separated and the aqueous layer was extracted with EtOAc (20.0 mL x 2). The combined organic layer dried over anhydrous Na_2SO_4. The solvent was removed under reduced pressure. Finally, the crude residue was purified by flash chromatography (EtOAc/PE = 1:10) to afford (*R*)-4-((triisopropylsilyl)oxy)butan-2-ol (***R*-11**, 0.7 g, 95% isolated yield).

Step 3: The etherification and hydrogenation were carried out *via* the procedure described above to afford the compound ***S*-1i**.

General Procedure for the Synthesis of 15a

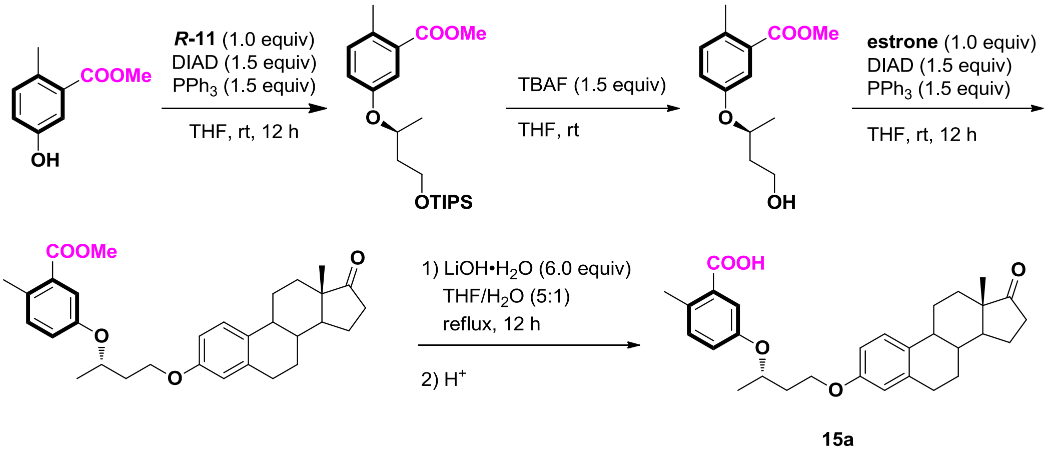

The synthesis of **15a** was carried out *via* two steps mitsunobu reaction procedure described above to afford the compound corresponding product. And the hydrogenation was carried out *via* the procedure described above to afford the compound **15a**.

2-methyl-5-(((2S)-4-(((13S)-13-methyl-17-oxo-7,8,9,11,12,13,14,15,16,17-decahydro-6H-cyclopenta[a]phenanthren-3-yl)oxy)butan-2-yl)oxy)benzoic acid (15a)

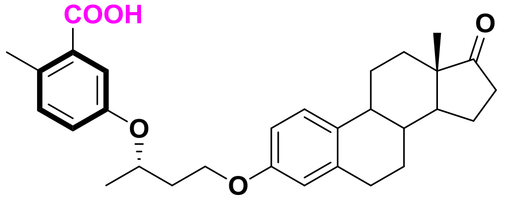

15a

^{1}H NMR (400 MHz, $CDCl_3$) δ 7.62 (d, *J* = 2.7 Hz, 1H), 7.16 (t, *J* = 9.0 Hz, 2H), 7.01 (dd, *J* = 8.4, 2.8 Hz, 1H), 6.71 (dd, *J* = 8.6, 2.6 Hz, 1H), 6.64 (d, *J* = 2.5 Hz, 1H), 4.73 – 4.63 (m, 1H), 4.16 – 4.02 (m, 2H), 2.92 – 2.79 (m, 2H), 2.56 (s, 3H), 2.49 (dd, *J* = 18.9, 8.5 Hz, 1H), 2.38 (dd, *J* = 10.3, 5.9 Hz, 1H), 2.24 – 1.92 (m, 7H), 1.66 – 1.40 (m, 6H), 1.37 (d, *J* = 6.1 Hz, 3H), 0.89 (s, 3H). ^{13}C NMR (100 MHz, $CDCl_3$) δ 221.14, 172.57, 156.86, 155.90, 137.75, 133.42, 132.97, 132.10, 129.01, 126.33, 121.32, 118.31, 114.54, 112.24, 71.25, 64.16, 50.42, 48.04, 43.98, 38.36, 36.40, 35.89, 31.58, 29.64, 26.55, 25.92, 21.59, 21.20, 19.87, 13.86. HRMS (ESI) Calcd for $C_{30}H_{37}O_5$ $[M+H]^+$: 477.2641; found: 477.2636.

Supplementary Tables

Supplementary Table 1. Optimization of Reaction Parameters [a]

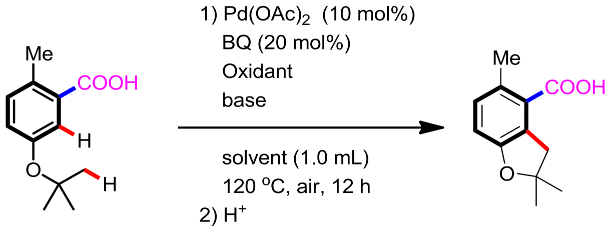

1a, 0.1 mmol — **2aa**

entry	oxidants (2.0 eq.)	bases (2.0 eq.)	solvents	results[b]
**1**	**Ag_2CO_3**	**$KHCO_3$**	**^{t}AmylOH**	**P (26%) + STM (40%)**
2	AgOAc	$KHCO_3$	^{t}AmylOH	P (20%) + STM (40%)
3	AgOTFA	$KHCO_3$	^{t}AmylOH	P (26%) + STM (30%)
4	$AgNO_3$	$KHCO_3$	^{t}AmylOH	P (18%) + STM (33%)
5	AgF	$KHCO_3$	^{t}AmylOH	P (17%) + STM (59%)
6	Ag_3PO_4	$KHCO_3$	^{t}AmylOH	P (11%) + STM (66%)
7	Ag_2O	$KHCO_3$	^{t}AmylOH	P (3%) + STM (74%)
8	Ag_2CO_3 (1.0 eq.)	$KHCO_3$	^{t}AmylOH	P (19%) + STM (57%)
9	Ag_2CO_3 (1.5 eq.)	$KHCO_3$	^{t}AmylOH	P (12%) + STM (28%)
10	Ag_2CO_3 (2.5 eq.)	$KHCO_3$	^{t}AmylOH	P (19%) + STM (50%)
11	Ag_2CO_3	$KHCO_3$	^{t}BuOH	P (9%) + STM (83%)
12	Ag_2CO_3	$KHCO_3$	^{i}PrOH	P (0%) + STM (64%)
13	Ag_2CO_3	$KHCO_3$	MeOH	P (0%) + STM (78%)
14	Ag_2CO_3	$KHCO_3$	$CF_3CH(OH)CF_3$	P (4%) + STM (73%)
15	Ag_2CO_3	$KHCO_3$	DCE	P (22%) + STM (30%)
16	Ag_2CO_3	$KHCO_3$	toluene	P (22%) + STM (18%)
17	Ag_2CO_3	$KHCO_3$	THF	P (8%) + STM (66%)
18	Ag_2CO_3	$KHCO_3$	DMF	P (4%) + STM (77%)
19	Ag_2CO_3	$KHCO_3$	^{t}AmylOH	P (24%) + STM (58%)
20	Ag_2CO_3	K_2CO_3	^{t}AmylOH	P (6%) + STM (74%)
21	Ag_2CO_3	$NaHCO_3$	^{t}AmylOH	P (27%) + STM (24%)
22	Ag_2CO_3	Na_2CO_3	^{t}AmylOH	P (31%) + STM (29%)
23	Ag_2CO_3	KOAc	^{t}AmylOH	P (20%) + STM (54%)
**24**	**Ag_2CO_3**	**NaOAc**	**^{t}AmylOH**	**P (35%) + STM (18%)**
25	Ag_2CO_3	K_2HPO_4	^{t}AmylOH	P (19%) + STM (37%)
**26**	**Ag_2CO_3**	**KH_2PO_4**	**^{t}AmylOH**	**P (36%) + STM (33%)**
27	Ag_2CO_3	K_3PO_4	^{t}AmylOH	P (0%) + STM (94%)
28	Ag_2CO_3	NaH_2PO_4	^{t}AmylOH	P (16%) + STM (74%)
29	Ag_2CO_3	Na_2HPO_4	^{t}AmylOH	P (26%) + STM (48%)
30	Ag_2CO_3	Na_3PO_4	^{t}AmylOH	P (25%) + STM (41%)
31	Ag_2CO_3	Li_2CO_3	^{t}AmylOH	P (14%) + STM (74%)
32	Ag_2CO_3	LiOAc	^{t}AmylOH	P (30%) + STM (40%)
33	Ag_2CO_3	CsOAc	^{t}AmylOH	P (33%) + STM (47%)
34	Ag_2CO_3	CsOPiv	^{t}AmylOH	P (14%) + STM (25%)
**35**	**Ag_2CO_3**	**KH_2PO_4 (1.0 eq.) + NaOAc (1.0 eq.)**	**^{t}AmylOH**	**P (42%) + STM (20%)**
36	Ag_2CO_3	KH_2PO_4 (1.5 eq.) +NaOAc (1.0 eq.)	^{t}AmylOH	P (48%) + STM (13%)
37	Ag_2CO_3	KH_2PO_4 (0.5 eq.) +NaOAc (1.0 eq.)	^{t}AmylOH	P (41%) + STM (16%)
**38[c]**	**Ag_2CO_3**	**KH_2PO_4 (1.0 eq.) +NaOAc (1.5 eq.)**	**^{t}AmylOH**	**P (48%) + STM (27%)**
39	Ag_2CO_3	KH_2PO_4 (1.0 eq.) +NaOAc (0.5 eq.)	^{t}AmylOH	P (48%) + STM (21%)
40	Ag_2CO_3	KH_2PO_4 (2.0 eq.) +NaOAc (2.0 eq.)	^{t}AmylOH	P (45%) + STM (22%)

[a] Reaction conditions: **1a** (0.1 mmol), $Pd(OAc)_2$ (10 mol%), BQ (20 mol%), Oxidant (2.0 equiv), base (2.0 equiv) in solvent (1 mL) at 120 °C for 12h. [b] Yield of **2aa** was determined by ^{1}H NMR using CH_2Br_2 and $Cl_2CHCHCl_2$ as internal standard. [c] with 10 mol% $Pd(OAc)_2$ and 20 mol% BQ at 140 °C for 12 h.

Supplementary Table 2. Screening temperature [a]

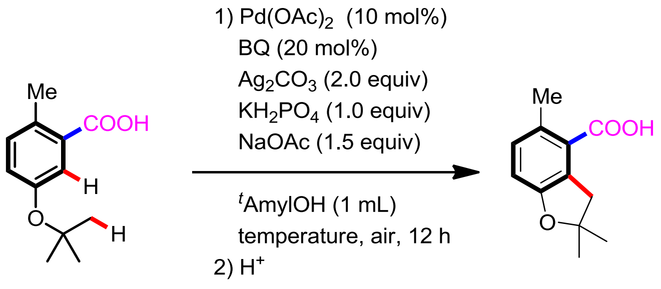

1a, 0.1 mmol **2aa**

entry	temperature (°C)	results^{b}
1	100	43%
2	120	32%
3	140	50%

^{a} Reaction conditions: **1a** (0.1 mmol), $Pd(OAc)_2$ (10 mol%), BQ (20 mol%), Ag_2CO_3 (2.0 equiv), KH_2PO_4 (1.0 equiv), NaOAc (1.5 equiv) in ^{t}AmylOH (1 mL) for 12h. ^{b} Yield of **2aa** was determined by ^{1}H NMR using CH_2Br_2 and $Cl_2CHCHCl_2$ as internal standard.

Supplementary Table 3. Screening quantity of solvents [a]

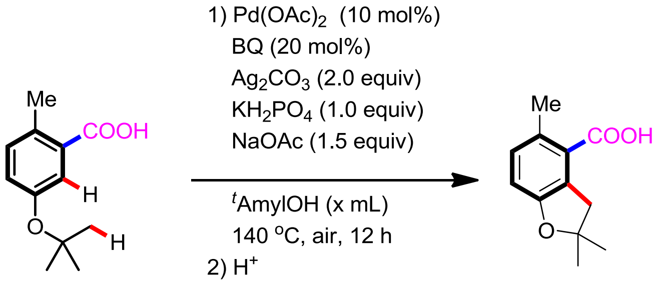

1a, 0.1 mmol **2aa**

entry	solvent (mL)	results^{b}
1	0.5	8%
2	1.0	50%
3	2.0	40%

^{a} Reaction conditions: **1a** (0.1 mmol), $Pd(OAc)_2$ (10 mol%), BQ (20 mol%), Ag_2CO_3 (2.0 equiv), KH_2PO_4 (1.0 equiv), NaOAc (1.5 equiv) in ^{t}AmylOH at 140 °C for 12h. ^{b} Yield of **2aa** was determined by ^{1}H NMR using CH_2Br_2 and $Cl_2CHCHCl_2$ as internal standard.

Supplementary Figure 65. Screening ligands [a, b]

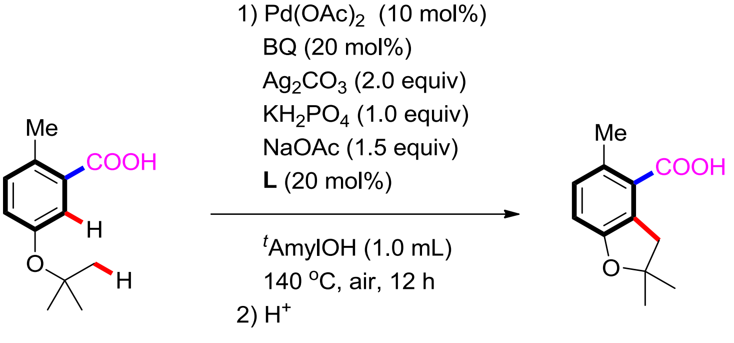

1a, 0.1 mmol **2aa**

Ac-Ala-OH	Boc-Gly-OH	Boc-Leu-OH	Boc-iLeu-OH	Boc-Nva-OH
6%	5%	14%	12%	8%
Boc-Val-OH	Boc-Phe-OH	Boc-Pro-OH	Ac-Pro-OH	Cbz-Pro-OH
11%	24%	50%	45%	38%

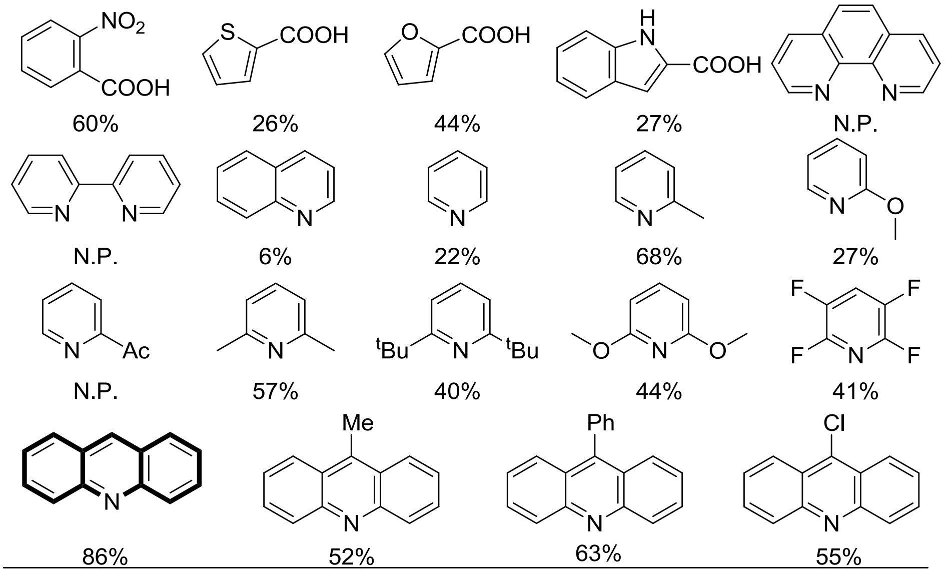

[a] Reaction conditions: **1a** (0.1 mmol), $Pd(OAc)_2$ (10 mol%), BQ (20 mol%), Ag_2CO_3 (2.0 equiv), KH_2PO_4 (1.0 equiv), NaOAc (1.5 equiv), **L** (20 mol%) in *t*AmylOH (1.0 mL) for 12h. [b] Yield of **2aa** was determined by ^1H NMR using CH_2Br_2 and $Cl_2CHCHCl_2$ as internal standard.

Standard Procedure for Oxidative Coupling to Construct Dihydrobenzofurans

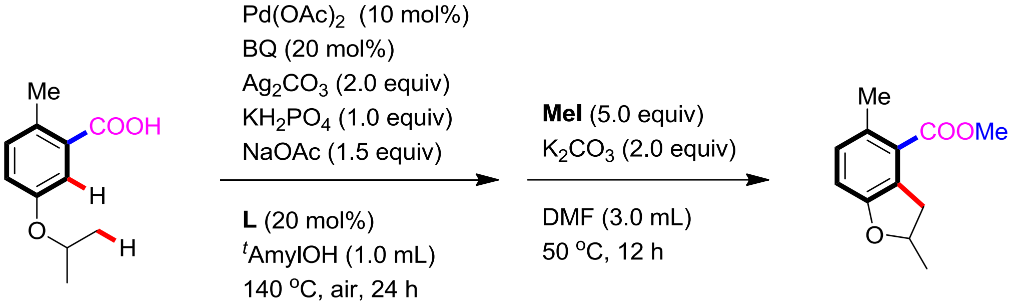

1a, 0.3 mmol **2a**

General Procedure: To a 20 mL oven-dried glass tube was added **1a** (58.3 mg, 0.3 mmol), $Pd(OAc)_2$ (6.7 mg, 10 mol%), BQ (6.6 mg, 20 mol%), Ag_2CO_3 (165.5 mg, 2.0 equiv), acridine (10.8 mg, 20 mol%), KH_2PO_4 (40.8 mg, 1.0 equiv), NaOAc (37.0 mg, 1.5 equiv) and ^{t}AmylOH (2.0 mL). The tube was sealed and the reaction mixture was stirred at 140 ℃ for 24 h under an air atmosphere. The mixture was cooled to rt. After removal of the solvent, MeI (213.0 mg, 5.0 equiv), K_2CO_3 (83.0 mg, 2.0 equiv), and DMF (3.0 mL) were added into the Schlenk tube. The mixture was stirred at 50 °C for 12 h. Then the suspension was filtered through a celite pad and washed with EtOAc. The **2a** was obtained in 85% yields after purification by flash chromatography on silica gel (PE/EtOAc = 100:1 to PE/EtOAc = 30:1). When substrate **1** is 0.3 mmol, the standard reaction performs a better repeatable results than **1** is 0.1 mmol.

General Procedures for Further Transformations to Corresponding Products

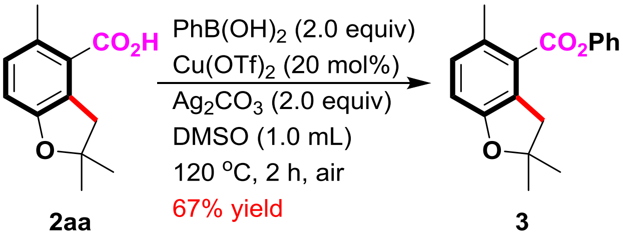

2aa **3**

Compound **3** was synthesized according to the following procedure.[5] To a 10 mL oven-dried glass tube was added **2aa** (41.2 mg, 0.2 mmol), $PhB(OH)_2$ (48.8 mg, 2.0 equiv), $Cu(OTf)_2$ (14.5 mg, 20 mol%), Ag_2CO_3 (110.3 mg, 2.0 equiv) and DMSO (1.0 mL). The vial was sealed and stirred at 120±5 ℃ for 2 h. Upon completion of the reaction, the mixture was cooled to rt and diluted with diethyl ether (30.0 mL). The mixture was then filtered through a short silica column to remove the deposition. The organic layers were washed with brine (50.0 mL x 3), dried over $MgSO_4$, and filtered. The solvent was removed under reduced pressure. Finally, the crude residue was purified by flash chromatography (EtOAc/PE = 1:20) to afford compound **3** (38.0 mg, 67%).

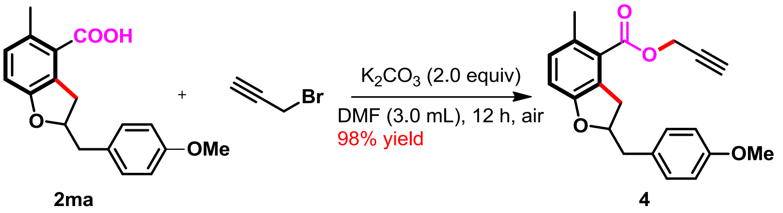

Compound **4** was synthesized according to the following procedure. To a 10 mL oven-dried glass tube was added **2ma** (89.4 mg, 0.3 mmol), K_2CO_3 (82.9 mg, 2.0 equiv) and 3-bromoprop-1-yne (178.4 mg, 5.0 equiv) and DMF (3.0 mL). The vial was sealed and stirred at 25 ℃ for 12 h. Upon completion of the reaction, the mixture was cooled to rt and diluted with diethyl ether (30.0 mL). The mixture was then filtered through a short silica column to remove the deposition. The organic layers were washed with brine (50.0 mL × 2), dried over $MgSO_4$, and filtered. The solvent was removed under reduced pressure. Finally, the crude residue was purified by flash chromatography (EtOAc/PE = 1:10) to afford compound **4** (98.8 mg, 98%).

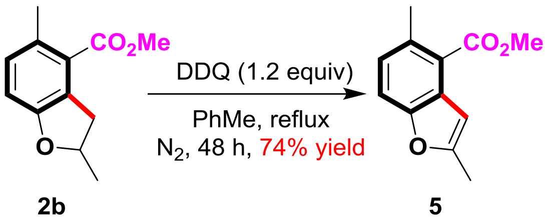

Compound **5** was synthesized according to the following procedure.[6] Under N_2 atmosphere, to a 10 mL oven-dried glass tube was added **2b** (41.2 mg, 0.2 mmol), DDQ (54.5 mg, 1.2 equiv) and DMF (3.0 mL). The vial was sealed and stirred at 120 ℃ for 48 h. Upon completion of the reaction, the mixture was cooled to rt and diluted with ethyl acetate (30.0 mL). The solvent was removed under reduced pressure. Finally, the crude residue was purified by flash chromatography (EtOAc/PE = 1:20) to afford compound **5** (29.9 mg, 74%).

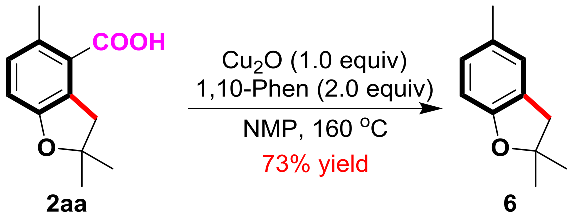

Compound **6** was synthesized according to the following procedure.[7,8] To a 10 mL oven-dried glass tube was added **2aa** (41.2 mg, 0.2 mmol), Cu_2O (28.6 mg, 1.0 equiv) and 1, 10-Phen (72.1 mg, 2.0 equiv) and NMP (3.0 ml) The vial was sealed and stirred at 160 ℃ for 36 h. Upon completion of the reaction, the mixture was cooled to rt and diluted with brine (20.0 mL). The resulting mixture was extracted with ethyl acetate (20.0 mL x 3). The combined organic layers were dried over Na_2SO_4, filtered. The solvent was removed under reduced pressure. Finally, the crude residue was purified by flash chromatography (EtOAc/PE = 1:10) to afford compound **6** (23.7mg,

73%).

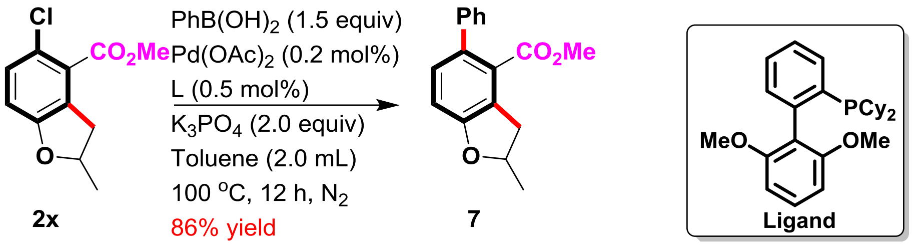

2x **7**

Compound **7** was synthesized according to the following procedure.[9] Under N_2 atmosphere, to a 10 mL oven-dried glass tube was added $PhB(OH)_2$ (54.9 mg, 1.5 equiv), $Pd(OAc)_2$ (1.4 mg, 0.2 mol%), Ligand (6.0 mg, 0.5 mol%), K_3PO_4 (127.4 mg, 2.0 equiv) and dry toluene (2.0 mL). The resulting mixture was stirred at room temperature for 2 min. Then **2x** (68.0 mg, 0.3 mmol in 1.0 mL toluene) was added dropwise by syringe through the septum. The reaction mixture was heated at 100 ℃ for 12 h. The reaction mixture was cooled to rt and diluted with diethyl ether (10.0 mL). The mixture was then filtered through a short silica column to remove the deposition. The solvent was removed under reduced pressure. Finally, the crude residue was purified by flash chromatography (EtOAc/PE = 1:20) to afford compound **7** (69.2 mg, 86%).

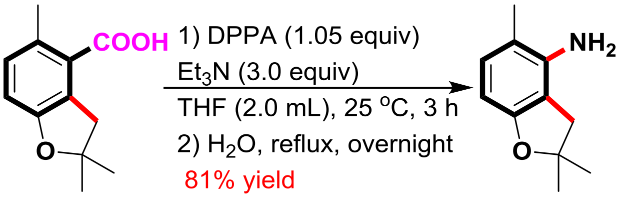

2aa **8**

Compound **8** was synthesized according to the procedure of Curtis rearrangement.[10] To a 10 mL oven-dried glass tube was added **2aa** (62.0 mg, 0.3 mmol), Et_3N (91.1 mg, 3.0 equiv) and THF (3.0 mL). DPPA (86.7 mg, 1.05 equiv) was added dropwise and the reaction mixture was stirred at 25 ℃ for 3 h. H_2O (2 mL) was added and the reaction mixture refluxed for 2 h. The solvent was removed under reduced pressure and the residue was treated with saturated aqueous K_2CO_3 solution(10.0 mL), and the mixture was extracted with EtOAc (15.0 mL x 2). The combined organic were washed with brine (20.0 mL), dried over Na_2SO_4. The solvent was removed under reduced pressure. Finally, the crude residue was purified by flash chromatography (EtOAc/PE = 1:4) to afford compound **8** (43.0 mg, 81%).

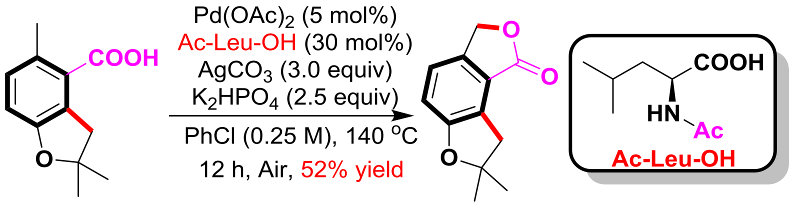

2aa **9**

Compound **9** was synthesized according to the following procedure.[11] Under N_2 atmosphere, to a 10 mL oven-dried glass tube was added **2aa** (41.2 mg, 0.2 mmol), $Pd(OAc)_2$ (2.3 mg, 5 mol%), Ac-Leu-OH (10.4 mg, 30 mol%), Ag_2CO_3 (165.5 mg, 3.0 equiv) and K_2HPO_4 (78.1 mg, 2.5 equiv) and PhCl (2.0 mL). The mixture was stirred at 140 ℃ for 14 h. The mixture was cooled to rt, diluted with ethyl acetate (5.0 mL). The mixture was then filtered through a short silica column to remove the deposition. The filtrate was removed under reduced pressure. Finally, the crude residue was purified by flash chromatography (EtOAc/PE = 1:10) to afford compound **9** (21.5 mg, 52%).

Methyl-2-(2-hydroxyethyl)-5-methyl-2,3-dihydrobenzofuran-4-carboxylate

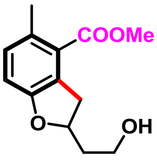

(*rac*)-2i-1

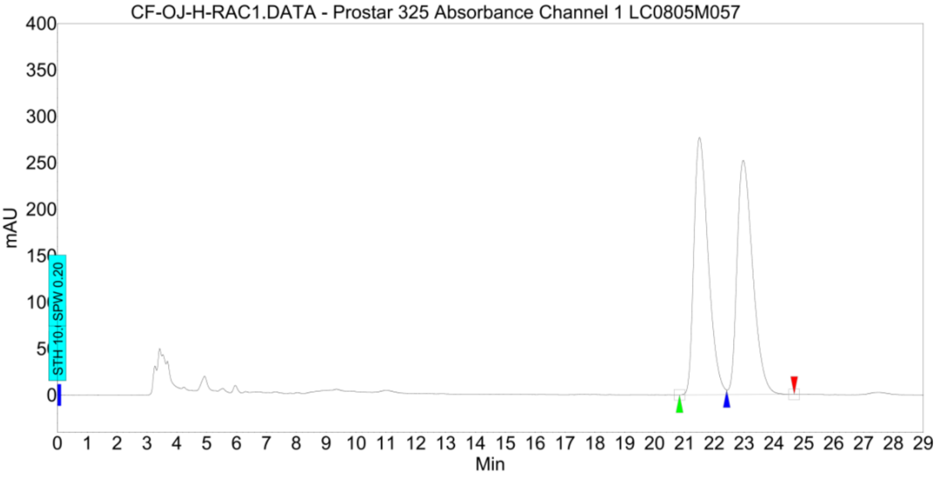

Peak results :

Index	文件名	时间 [Min]	数量 [% 面积]	高度 [mAU]	Area [mAU.Min]	Area % [%]
1	未知	21.51	49.89	276.8	153.6	49.894
2	未知	22.97	50.11	252.1	154.3	50.106
Total			100.00	528.9	307.8	100.000

Methyl (R)-2-(2-hydroxyethyl)-5-methyl-2,3-dihydrobenzofuran-4-carboxylate

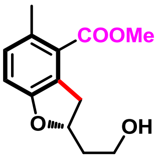

(*R*)-2i-1

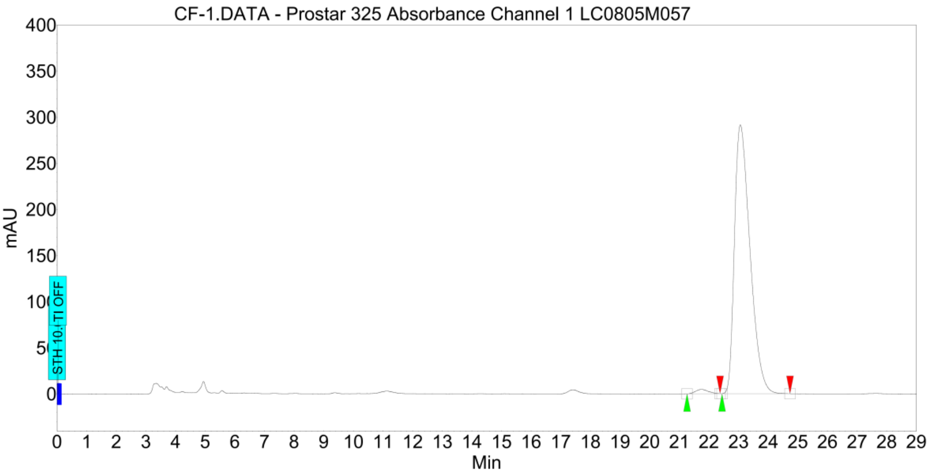

Peak results :

Index	文件名	时间 [Min]	数量 [% 面积]	高度 [mAU]	Area [mAU.Min]	Area % [%]
1	未知	21.75	1.28	4.7	2.3	1.283
2	未知	23.06	98.72	291.4	177.2	98.717
Total			100.00	296.1	179.5	100.000

Methyl (S)-2-(2-hydroxyethyl)-5-methyl-2,3-dihydrobenzofuran-4-carboxylate

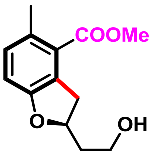

(*S*)-2i-1

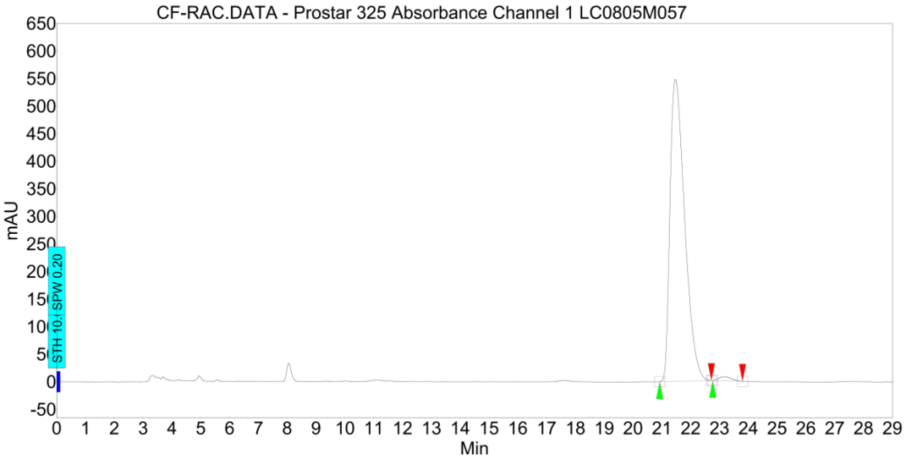

Peak results :

Index	文件名	时间 [Min]	数量 [% 面积]	高度 [mAU]	Area [mAU.Min]	Area % [%]
1	未知	21.47	98.86	547.7	322.7	98.861
2	未知	23.17	1.14	7.5	3.7	1.139
Total			100.00	555.3	326.4	100.000

Analytical Data of Products

2,2,5-trimethyl-2,3-dihydrobenzofuran-4-carboxylic acid

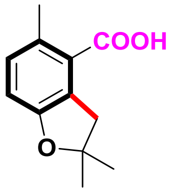

2aa

2aa was obtained from **2a** by hydrolysis. 1H NMR (400 MHz, CDCl3) δ 7.02 (d, *J* = 8.1 Hz, 1H), 6.80 (d, *J* = 8.1 Hz, 1H), 3.32 (s, 2H), 2.52 (s, 3H), 1.47 (s, 6H). ^{13}C NMR (100 MHz, CDCl3) δ 173.30, 157.43, 131.64, 131.33, 130.35, 125.99, 113.33, 86.82, 44.96, 28.25, 21.40. HRMS (ESI) Calcd for $C_{12}H_{15}O_3$ $[M+H]^+$: 207.1012; found: 207.1016.

Corresponding products NMR Data

Methyl 2, 2, 5-trimethyl-2, 3-dihydrobenzofuran-4-carboxylate

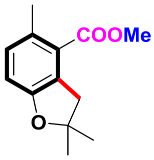

2a

2a was obtained as a yellow oil (56.1 mg, 85%). ^{1}H NMR (400 MHz, $CDCl_3$) δ 7.01 (t, *J* = 7.1 Hz, 1H), 6.81 (d, *J* = 8.1 Hz, 1H), 3.34 (s, 2H), 2.53 (s, 3H), 1.48 (s, 6H). ^{13}C NMR (100 MHz, CDCl3) δ 168.23, 157.25, 130.94, 130.03, 128.72, 127.58, 112.28, 86.69, 51.54, 44.34, 28.21, 20.72 ppm; HRMS (ESI) Calcd for $C_{13}H_{17}O_3$ $[M+H]^+$: 221.1179; found: 221.1172.

Methyl 2, 5-dimethyl-2, 3-dihydrobenzofuran-4-carboxylate

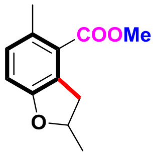

2b

2b was obtained as a yellow oil (47.0 mg, 76%). ^{1}H NMR (400 MHz, $CDCl_3$) δ 6.98 (d, *J* = 8.1 Hz, 1H), 6.76 (d, *J* = 8.1 Hz, 1H), 4.98 – 4.82 (m, 1H), 3.88 (s, 3H), 3.50 (dd, *J* = 16.7, 8.8 Hz, 1H), 2.99 (dd, *J* = 16.7, 7.7 Hz, 1H), 2.43 (s, 3H), 1.45 (d, *J* = 6.2 Hz, 3H). ^{13}C NMR (100 MHz, $CDCl_3$) δ 168.17, 157.92, 130.91, 130.26, 128.73, 127.45, 112.03, 79.72, 51.58, 38.58, 21.76, 20.74. HRMS (ESI) Calcd for $C_{12}H_{15}O_3$ $[M+H]^+$: 207.1016; found: 207.1016.

Methyl 2-ethyl-5-methyl-2, 3-dihydrobenzofuran-4-carboxylate

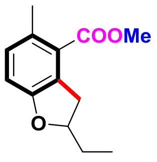

2c

2c was obtained as a yellow oil (41.6 mg, 63%). ^{1}H NMR (400 MHz, $CDCl_3$) δ 6.97 (d, J = 8.1 Hz, 1H), 6.76 (d, J = 8.1 Hz, 1H), 4.77 – 4.63 (m, 1H), 3.88 (s, 3H), 3.45 (dd, J = 16.8, 9.0 Hz, 1H), 3.03 (dd, J = 16.8, 7.9 Hz, 1H), 2.42 (s, 3H), 1.81 (dd, J = 14.1, 7.0 Hz, 1H), 1.71 (dd, J = 13.9, 7.0 Hz, 1H), 1.02 (t, J = 7.4 Hz, 3H). ^{13}C NMR (100 MHz, $CDCl_3$) δ 168.26, 158.08, 130.88, 130.13, 128.70, 127.39, 111.93, 84.79, 51.63, 36.46, 28.96, 20.79, 9.59. HRMS (ESI) Calcd for $C_{13}H_{17}O_3$ $[M+H]^+$: 221.1168; found: 221.1172.

Methyl 5-methyl-2-pentyl-2,3-dihydrobenzofuran-4-carboxylate

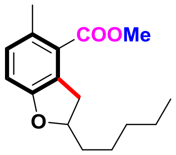

2d

2d was obtained as a yellow oil (51.3 mg, 65%). ^{1}H NMR (400 MHz, $CDCl_3$) δ 6.97 (d, J = 8.1 Hz, 1H), 6.76 (d, J = 8.1 Hz, 1H), 4.80 – 4.69 (m, 1H), 3.88 (s, 3H), 3.45 (dd, J = 16.8, 8.9 Hz, 1H), 3.02 (dd, J = 16.8, 8.0 Hz, 1H), 2.42 (s, 3H), 1.88 – 1.74 (m, 1H), 1.65 (ddd, J = 13.5, 10.8, 5.2 Hz, 1H), 1.56 – 1.39 (m, 2H), 1.33 (dd, J = 6.9, 3.7 Hz, 4H), 0.90 (t, J = 6.9 Hz, 3H) ppm. ^{13}C NMR (100 MHz, $CDCl_3$) δ 168.24, 158.05, 130.84, 130.06, 128.69, 127.44, 111.93, 83.65, 51.56, 36.89, 36.07, 31.73, 25.04, 22.59, 20.70, 14.02. HRMS (ESI) Calcd for $C_{16}H_{23}O_3$ $[M+H]^+$: 263.1637; found: 263.1642.

Methyl 2-heptyl-5-methyl-2, 3-dihydrobenzofuran-4-carboxylate

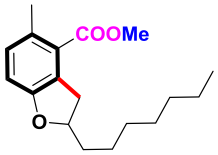

2e

2e was obtained as a yellow oil (52.2 mg, 60%). ^{1}H NMR (400 MHz, $CDCl_3$) δ 6.97 (d, J = 8.1 Hz, 1H), 6.76 (d, J = 8.1 Hz, 1H), 4.84 – 4.68 (m, 1H), 3.88 (s, 3H), 3.45 (dd, J = 16.8, 8.9 Hz, 1H), 3.02 (dd, J = 16.8, 8.0 Hz, 1H), 2.42 (s, 3H), 1.80 (dd, J = 9.9, 5.9 Hz, 1H), 1.72 – 1.60 (m, 1H), 1.54 – 1.18 (m, 10H), 0.88 (t, J = 6.6 Hz, 3H). ^{13}C NMR (100 MHz, $CDCl_3$) δ 168.26, 158.04, 130.86, 130.11, 128.73, 127.40, 111.95, 83.66, 51.62, 36.91, 36.12, 31.82, 29.53, 29.25, 25.39, 22.68, 20.77, 14.13. HRMS (ESI) Calcd for $C_{19}H_{15}O_3$ $[M+H]^+$: 291.1006; found: 291.1016.

Methyl 2-isopentyl-5-methyl-2, 3-dihydrobenzofuran-4-carboxylate

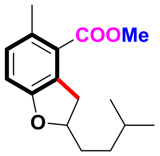

2f

2f was obtained as a yellow oil (51.9 mg, 66%). ^{1}H NMR (400 MHz, $CDCl_3$) δ 6.97

(d, J = 8.1 Hz, 1H), 6.76 (d, J = 8.1 Hz, 1H), 4.80 – 4.64 (m, 1H), 3.93 – 3.84 (m, 3H), 3.45 (dd, J = 16.8, 8.9 Hz, 1H), 3.02 (dd, J = 16.8, 8.0 Hz, 1H), 2.45 – 2.38 (m, 3H), 1.89 – 1.75 (m, 1H), 1.74 – 1.61 (m, 1H), 1.47 – 1.22 (m, 3H), 0.96 – 0.88 (m, 6 H). ^{13}C NMR (100 MHz, $CDCl_3$) δ 168.23, 158.05, 130.85, 130.07, 128.67, 127.45, 111.92, 83.91, 51.54, 36.89, 34.35, 33.95, 28.03, 22.53, 20.69. HRMS (ESI) Calcd for $C_{16}H_{23}O_3$ [M+H]+: 263.1638; found: 263.1642.1287.

Methyl 2-(2-fluoroethyl)-5-methyl-2, 3-dihydrobenzofuran-4-carboxylate

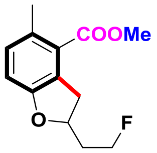

2g

2g was obtained as a yellow oil (51.4 mg, 72%). ^{1}H NMR (400 MHz, $CDCl_3$) δ 6.99 (d, J = 8.1 Hz, 1H), 6.78 (d, J = 8.1 Hz, 1H), 5.01 – 4.87 (m, 1H), 4.81 – 4.51 (m, 2H), 3.88 (s, 3H), 3.55 (dd, J = 16.8, 9.1 Hz, 1H), 3.09 (dd, J = 16.8, 7.6 Hz, 1H), 2.43 (s, 3H), 2.20 – 2.02 (m, 2H). ^{13}C NMR (100 MHz, $CDCl_3$) δ 168.03, 157.64, 131.06, 130.62, 128.17, 127.51, 112.15, 80.74 (d, J = 164.7 Hz), 79.47 (d, J = 4.7 Hz), 51.59, 36.99, 36.85 (d, J = 19.4 Hz), 20.71. HRMS (ESI) Calcd for $C_{13}H_{16}FO_3$ $[M+H]^+$: 239.1076; found: 239.1078.

Methyl 2-(2-acetoxyethyl)-5-methyl-2, 3-dihydrobenzofuran-4-carboxylate

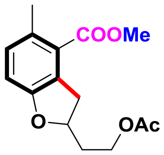

2h

2h was obtained as a yellow oil (57.0 mg, 72%). ^{1}H NMR (400 MHz, $CDCl_3$) δ 6.91 (d, J = 8.1 Hz, 1H), 6.70 (d, J = 8.1 Hz, 1H), 4.86 – 4.73 (m, 1H), 4.19 m, 2H), 3.81 (s, 3H), 3.45 (dd, J = 16.8, 9.1 Hz, 1H), 3.00 (dd, J = 16.8, 7.5 Hz, 1H), 2.36 (s, 3H), 2.05 – 1.89 (m, 5H). ^{13}C NMR (100 MHz, $CDCl_3$) δ 171.04, 168.06, 157.66, 131.09, 130.62, 128.16, 127.41, 112.20, 79.97, 61.07, 51.64, 37.00, 35.08, 20.98, 20.79. HRMS (ESI) Calcd for $C_{14}H_{17}O_5$ $[M+H]^+$: 265.1075; found: 265.1071.

Methyl-5-methyl-2-(2-((triisopropylsilyl)oxy)ethyl)-2,3-dihydrobenzofuran-4-carboxylate

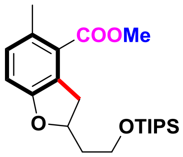

2i

2i was obtained as a yellow oil (73.0 mg, 62%). ^{1}H NMR (400 MHz, $CDCl_3$) δ 6.97 (d, J = 8.1 Hz, 1H), 6.76 (d, J = 8.1 Hz, 1H), 5.04 – 4.87 (m, 1H), 3.94 – 3.83 (m, 5H), 3.50 (dd, J = 16.8, 9.0 Hz, 1H), 3.10 (dd, J = 16.8, 7.8 Hz, 1H), 2.47 – 2.38 (m, 3H), 2.04 (ddt, J = 12.9, 7.5, 5.3 Hz, 1H), 1.98 – 1.83 (m, 1H), 1.19 – 0.96 (m, 21H). ^{13}C NMR (100 MHz, $CDCl_3$) δ 168.17, 157.93, 130.82, 130.22, 128.71, 127.45, 112.01, 80.80, 59.94, 51.54, 39.30, 37.14, 20.69, 18.02, 11.96. HRMS (ESI) Calcd for $C_{22}H_{37}O_4Si$ $[M+H]^+$: 393.2449; found: 393.2456.

Methyl2-(2-(1,3-dioxoisoindolin-2-yl)ethyl)-5-methyl-2,3-dihydrobenzofuran-4-

carboxylate

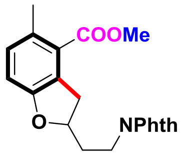

2j

2j was obtained as a yellow oil (82.4 mg, 78%).^{1}H NMR (400 MHz, $CDCl_3$) δ 7.86 (dd, *J* = 5.3, 3.1 Hz, 2H), 7.73 (dd, *J* = 5.4, 3.1 Hz, 2H), 6.95 (d, *J* = 8.1 Hz, 1H), 6.69 (d, *J* = 8.1 Hz, 1H), 4.82 (qd, *J* = 8.3, 4.7 Hz, 1H), 3.96 – 3.88 (m, 2H), 3.86 (s, 3H), 3.54 (dd, *J* = 16.9, 9.1 Hz, 1H), 3.08 (dd, *J* = 16.9, 7.5 Hz, 1H), 2.42 (s, 3H), 2.18 (td, *J* = 14.6, 7.6 Hz, 1H), 2.05 (dt, *J* = 13.7, 6.1 Hz, 1H). ^{13}C NMR (100 MHz, $CDCl_3$) δ 168.35, 168.02, 157.70, 133.98, 132.16, 131.05, 130.62, 128.09, 127.28, 123.27, 112.28, 81.02, 51.61, 37.06, 34.89, 34.73, 20.81. HRMS (ESI) Calcd for $C_{21}H_{20}NO_5$ [M+H]+: 366.1333; found: 366.1336.

Methyl 2-benzyl-5-methyl-2, 3-dihydrobenzofuran-4-carboxylate

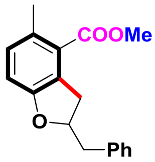

2k

2k was obtained as a yellow oil (46.6 mg, 55%). ^{1}H NMR (400 MHz, $CDCl_3$) δ 7.37 – 7.21 (m, 5H), 6.98 (d, *J* = 8.1 Hz, 1H), 6.78 (d, *J* = 8.1 Hz, 1H), 5.02 (tt, *J* = 14.8, 7.6 Hz, 1H), 3.86 (s, 3H), 3.42 (dd, *J* = 16.9, 8.9 Hz, 1H), 3.21 – 3.08 (m, 2H), 2.93 (dd, *J* = 13.9, 6.4 Hz, 1H), 2.42 (s, 3H). ^{13}C NMR (100 MHz, $CDCl_3$) δ 168.15, 157.85, 137.39, 131.00, 130.39, 129.38, 128.57, 128.40, 127.51, 126.65, 112.19, 83.81, 51.64, 42.07, 36.48, 20.79. HRMS (ESI) Calcd for $C_{18}H_{19}O_3$ $[M+H]^+$: 283.1331; found: 283.1329.

Methyl 5-methyl-2-phenethyl-2,3-dihydrobenzofuran-4-carboxylate

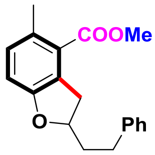

2l

2l was obtained as a yellow oil (68.4 mg, 77%). ^{1}H NMR (400 MHz, $CDCl_3$) δ 7.34 – 7.16 (m, 5H), 6.99 (d, *J* = 8.1 Hz, 1H), 6.79 (d, *J* = 8.1 Hz, 1H), 4.83 – 4.69 (m, 1H), 3.88 (s, 3H), 3.46 (dt, *J* = 14.8, 7.4 Hz, 1H), 3.05 (dd, *J* = 16.8, 7.7 Hz, 1H), 2.92 – 2.69 (m, 2H), 2.43 (s, 3H), 2.23 – 2.06 (m, 1H), 2.06 – 1.91 (m, 1H). ^{13}C NMR (100 MHz, $CDCl_3$) δ 168.19, 157.97, 141.46, 130.98, 130.33, 128.59, 128.50, 128.47, 127.41, 126.00, 112.09, 82.61, 51.64, 37.80, 36.92, 31.71, 20.82. HRMS (ESI) Calcd for $C_{19}H_{21}O_3$ $[M+H]^+$: 297.1476; found: 297.1485.

Methyl2-(4-methoxyphenethyl)-5-methyl-2,3-dihydrobenzofuran-4-carboxylate

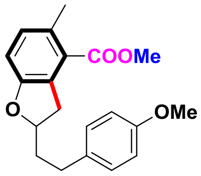

2m

2m was obtained as a yellow oil (54.8 mg, 56%). ^{1}H NMR (400 MHz, $CDCl_3$) δ 7.15 (d, *J* = 8.4 Hz, 2H), 6.99 (d, *J* = 8.1 Hz, 1H), 6.84 (d, *J* = 8.3 Hz, 2H), 6.80 (d, *J* = 8.1 Hz, 1H), 4.83 – 4.70 (m, 1H), 3.88 (s, 3H), 3.79 (s, 3H), 3.47 (dd, *J* = 16.8, 9.0 Hz, 1H), 3.05 (dd, *J* = 16.8, 7.8 Hz, 1H), 2.84 – 2.66 (m, 2H), 2.44 (s, 3H), 2.10 (dd, *J* = 14.1, 5.6 Hz, 1H), 1.94 (ddd, *J* = 14.4, 9.0, 5.0 Hz, 1H). ^{13}C NMR (100 MHz, $CDCl_3$) δ 168.20, 157.98, 157.88, 133.47, 130.96, 130.29, 129.38, 128.62, 127.41, 113.85, 112.07, 82.60, 55.28, 51.63, 38.04, 36.92, 30.77, 20.82. HRMS (ESI) Calcd for $C_{20}H_{23}O_4$ $[M+H]^+$: 327.1586; found: 327.1591.

2-(4-methoxyphenethyl)-5-methyl-2,3-dihydrobenzofuran-4-carboxylic acid

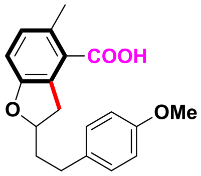

2ma

2ma was obtained from **2m** by hydrolysis. ^{1}H NMR (400 MHz, CDCl3) δ 7.15 (t, *J* = 5.8 Hz, 2H), 7.03 (d, *J* = 8.1 Hz, 1H), 6.88 – 6.82 (m, 3H), 4.78 (qd, *J* = 7.8, 5.4 Hz, 1H), 3.79 (s, 3H), 3.61 (dd, *J* = 17.2, 9.0 Hz, 1H), 3.18 (dd, *J* = 17.2, 7.8 Hz, 1H), 2.86 – 2.67 (m, 2H), 2.53 (s, 3H), 2.16 – 2.03 (m, 1H), 1.96 (ddd, *J* = 13.8, 8.5, 4.7 Hz, 1H). ^{13}C NMR (100 MHz, CDCl3) δ 172.70, 158.18, 157.89, 133.47, 131.82, 131.32, 130.10, 129.36, 125.85, 113.88, 113.06, 82.66, 55.29, 38.04, 37.47, 30.71, 21.37. HRMS (ESI) Calcd for $C_{19}H_{20}NaO_4$ $[M+Na]^+$: 335.1248; found: 335.1254. CCDC 1489220.

Supplementary Figure 66. X-ray for the compound 2ma

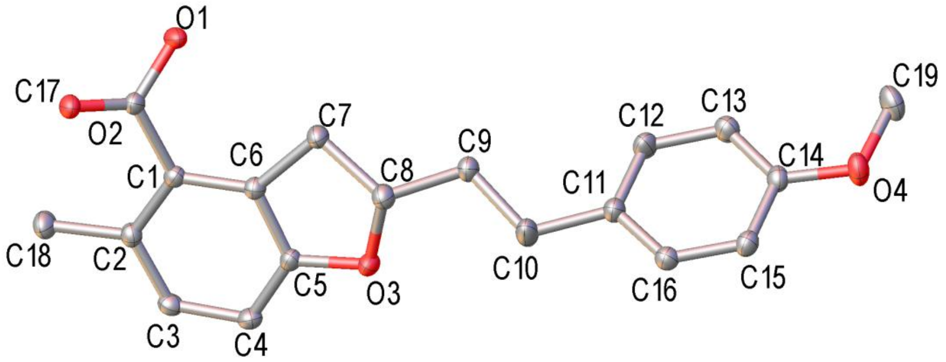

Supplementary Table 4. Crystal data and structure refinement for sa4680.

Identification code	sa4680
Empirical formula	C19 H20 O4
Formula weight	312.35
Temperature	173.1500 K
Wavelength	0.71073 Å

Crystal system	Triclinic
Space group	P -1
Unit cell dimensions	a = 7.2499(17) Å = 92.521(4)°.
	b = 14.162(3) Å = 92.121(5)°.
	c = 15.540(3) Å = 98.975(4)°.
Volume	1572.9(6) $Å^3$
Z	4
Density (calculated)	1.319 Mg/m^3
Absorption coefficient	0.092 mm^{-1}
F(000)	664
Crystal size	0.685 x 0.267 x 0.11 mm^3
Theta range for data collection	2.847 to 27.488°.
Index ranges	-9<=h<=9, -18<=k<=18, -19<=l<=20
Reflections collected	18376
Independent reflections	7166 [R(int) = 0.0381]
Completeness to theta = 26.000°	99.4 %
Absorption correction	Semi-empirical from equivalents
Max. and min. transmission	1.0000 and 0.8518
Refinement method	Full-matrix least-squares on F^2
Data / restraints / parameters	7166 / 142 / 467
Goodness-of-fit on F^2	1.134
Final R indices [I>2sigma(I)]	R1 = 0.0698, wR2 = 0.1464
R indices (all data)	R1 = 0.0825, wR2 = 0.1535
Extinction coefficient	n/a
Largest diff. peak and hole	0.334 and -0.245 e.$Å^{-3}$

Supplementary Table 5. Atomic coordinates (x 10^4) and equivalent isotropic displacement parameters ($Å^2$x 10^3) for sa4680. U(eq) is defined as one third of the trace of the orthogonalized U^{ij} tensor.

	x	y	z	U(eq)
O1	4302(2)	9097(1)	5770(1)	36(1)
O2	6990(2)	9582(1)	5140(1)	34(1)
O3	5355(2)	5749(1)	6287(1)	34(1)
O4	-4013(2)	1354(1)	6421(1)	45(1)
C1	6824(3)	8271(1)	6056(1)	27(1)
C2	8644(3)	8430(1)	6434(1)	29(1)
C3	9337(3)	7656(1)	6773(1)	31(1)

C4	8319(3)	6735(1)	6734(1)	32(1)
C5	6541(3)	6607(1)	6364(1)	28(1)
C6	5756(3)	7362(1)	6039(1)	27(1)
C7	3768(3)	6976(1)	5745(1)	32(1)
C8	3772(3)	5898(2)	5715(1)	35(1)
C9	2030(3)	5272(1)	5982(1)	34(1)
C10	2178(3)	4222(2)	5879(2)	49(1)
C11	456(3)	3511(1)	6043(1)	35(1)
C12	-1030(3)	3727(2)	6503(2)	41(1)
C13	-2568(3)	3028(2)	6649(1)	38(1)
C14	-2599(3)	2100(2)	6326(1)	33(1)
C15	-1118(3)	1873(1)	5857(1)	33(1)
C16	375(3)	2573(1)	5723(1)	32(1)
C17	6050(3)	9052(1)	5617(1)	28(1)
C18	9849(3)	9404(2)	6494(2)	40(1)
C19	-5570(3)	1553(2)	6885(2)	52(1)
O1A	9244(2)	9012(1)	9203(1)	42(1)
O2A	12002(2)	9625(1)	9850(1)	39(1)
O3A	10608(3)	5716(1)	8480(1)	52(1)
O4A	973(3)	1366(1)	8724(1)	69(1)
C1A	11846(3)	8262(1)	8886(1)	30(1)
C2A	13610(3)	8462(2)	8528(1)	32(1)
C3A	14357(3)	7705(2)	8153(1)	38(1)
C4A	13439(3)	6767(2)	8131(2)	40(1)
C5A	11721(3)	6602(2)	8477(1)	37(1)
C6A	10880(3)	7330(2)	8841(1)	32(1)
C7A	8973(3)	6892(2)	9098(2)	46(1)
C8A	9122(4)	5832(2)	9057(2)	63(1)
C9A	7173(9)	5246(4)	8855(5)	46(2)
C10A	7295(7)	4204(3)	8970(4)	60(2)
C11A	5486(6)	3496(3)	8878(3)	39(1)
C12A	5452(9)	2658(5)	9300(5)	41(1)
C13A	3924(3)	2079(2)	9265(1)	45(1)
C14A	2305(3)	2159(2)	8788(1)	36(1)
C15A	2137(4)	3003(2)	8420(2)	50(1)
C16A	3908(8)	3648(4)	8414(4)	45(1)
C17A	11041(3)	9036(1)	9355(1)	31(1)
C18A	14674(3)	9463(2)	8506(2)	44(1)
C19A	-725(4)	1429(3)	8262(2)	81(1)
C10B	6221(7)	4821(4)	9373(4)	47(1)
C11B	4873(7)	3889(4)	9169(3)	40(1)
C12B	5180(9)	2989(4)	9443(5)	39(2)
C16B	3241(9)	3877(4)	8679(4)	46(1)
C9B	7705(11)	4979(5)	8717(5)	44(2)

Supplementary Table 6. Bond lengths [Å] and angles [°] for sa4680.

O1-H1	0.8200
O1-C17	1.309(2)
O2-C17	1.226(2)
O3-C5	1.373(2)
O3-C8	1.472(2)
O4-C14	1.369(2)
O4-C19	1.419(3)
C1-C2	1.406(3)
C1-C6	1.394(3)
C1-C17	1.494(3)
C2-C3	1.390(3)
C2-C18	1.510(3)
C3-H3	0.9300
C3-C4	1.393(3)
C4-H4	0.9300
C4-C5	1.374(3)
C5-C6	1.392(3)
C6-C7	1.506(3)
C7-H7A	0.9700
C7-H7B	0.9700
C7-C8	1.526(3)
C8-H8	0.9800
C8-C9	1.508(3)
C9-H9A	0.9700
C9-H9B	0.9700
C9-C10	1.509(3)
C10-H10E	0.9700
C10-H10F	0.9700
C10-C11	1.514(3)
C11-C12	1.380(3)
C11-C16	1.391(3)
C12-H12	0.9300
C12-C13	1.403(3)
C13-H13	0.9300
C13-C14	1.382(3)
C14-C15	1.390(3)
C15-H15	0.9300
C15-C16	1.378(3)
C16-H16	0.9300
C18-H18D	0.9600
C18-H18E	0.9600
C18-H18F	0.9600

C19-H19D	0.9600
C19-H19E	0.9600
C19-H19F	0.9600
O1A-H1A	0.8200
O1A-C17A	1.310(2)
O2A-C17A	1.224(2)
O3A-C5A	1.382(3)
O3A-C8A	1.451(4)
O4A-C14A	1.360(3)
O4A-C19A	1.419(4)
C1A-C2A	1.406(3)
C1A-C6A	1.392(3)
C1A-C17A	1.495(3)
C2A-C3A	1.391(3)
C2A-C18A	1.507(3)
C3A-H3A	0.9300
C3A-C4A	1.388(3)
C4A-H4A	0.9300
C4A-C5A	1.366(3)
C5A-C6A	1.388(3)
C6A-C7A	1.501(3)
C7A-H7AA	0.9700
C7A-H7AB	0.9700
C7A-C8A	1.520(4)
C8A-H8A	0.9800
C8A-H8AA	0.9800
C8A-C9A	1.537(7)
C8A-C9B	1.520(8)
C9A-H9AA	0.9700
C9A-H9AB	0.9700
C9A-C10A	1.510(8)
C10A-H10A	0.9700
C10A-H10B	0.9700
C10A-C11A	1.520(6)
C11A-C12A	1.378(8)
C11A-C16A	1.380(7)
C12A-H12A	0.9300
C12A-C13A	1.269(8)
C13A-H13A	0.9300
C13A-H13B	0.9300
C13A-C14A	1.388(3)
C13A-C12B	1.467(7)
C14A-C15A	1.368(3)
C15A-H15A	0.9300
C15A-H15B	0.9300
C15A-C16A	1.456(6)

C15A-C16B	1.399(5)
C16A-H16A	0.9300
C18A-H18A	0.9600
C18A-H18B	0.9600
C18A-H18C	0.9600
C19A-H19A	0.9600
C19A-H19B	0.9600
C19A-H19C	0.9600
C10B-H10C	0.9700
C10B-H10D	0.9700
C10B-C11B	1.529(7)
C10B-C9B	1.508(9)
C11B-C12B	1.408(6)
C11B-C16B	1.380(6)
C12B-H12B	0.9300
C16B-H16B	0.9300
C9B-H9BA	0.9700
C9B-H9BB	0.9700

C17-O1-H1	109.5
C5-O3-C8	106.31(14)
C14-O4-C19	117.27(18)
C2-C1-C17	120.83(17)
C6-C1-C2	120.08(17)
C6-C1-C17	118.98(17)
C1-C2-C18	122.55(18)
C3-C2-C1	118.10(17)
C3-C2-C18	119.35(18)
C2-C3-H3	118.6
C2-C3-C4	122.70(18)
C4-C3-H3	118.6
C3-C4-H4	121.2
C5-C4-C3	117.60(18)
C5-C4-H4	121.2
O3-C5-C4	124.65(17)
O3-C5-C6	113.23(17)
C4-C5-C6	122.12(18)
C1-C6-C7	133.13(17)
C5-C6-C1	119.33(17)
C5-C6-C7	107.53(16)
C6-C7-H7A	111.4
C6-C7-H7B	111.4
C6-C7-C8	101.93(15)
H7A-C7-H7B	109.2
C8-C7-H7A	111.4
C8-C7-H7B	111.4

O3-C8-C7	105.97(16)
O3-C8-H8	108.2
O3-C8-C9	108.77(16)
C7-C8-H8	108.2
C9-C8-C7	117.26(18)
C9-C8-H8	108.2
C8-C9-H9A	109.2
C8-C9-H9B	109.2
C8-C9-C10	111.91(18)
H9A-C9-H9B	107.9
C10-C9-H9A	109.2
C10-C9-H9B	109.2
C9-C10-H10E	108.0
C9-C10-H10F	108.0
C9-C10-C11	117.36(19)
H10E-C10-H10F	107.2
C11-C10-H10E	108.0
C11-C10-H10F	108.0
C12-C11-C10	124.5(2)
C12-C11-C16	117.57(19)
C16-C11-C10	117.95(19)
C11-C12-H12	119.2
C11-C12-C13	121.6(2)
C13-C12-H12	119.2
C12-C13-H13	120.3
C14-C13-C12	119.3(2)
C14-C13-H13	120.3
O4-C14-C13	125.14(19)
O4-C14-C15	115.12(19)
C13-C14-C15	119.73(19)
C14-C15-H15	120.1
C16-C15-C14	119.76(19)
C16-C15-H15	120.1
C11-C16-H16	119.0
C15-C16-C11	121.98(19)
C15-C16-H16	119.0
O1-C17-C1	114.38(16)
O2-C17-O1	123.95(18)
O2-C17-C1	121.64(17)
C2-C18-H18D	109.5
C2-C18-H18E	109.5
C2-C18-H18F	109.5
H18D-C18-H18E	109.5
H18D-C18-H18F	109.5
H18E-C18-H18F	109.5
O4-C19-H19D	109.5

O4-C19-H19E	109.5
O4-C19-H19F	109.5
H19D-C19-H19E	109.5
H19D-C19-H19F	109.5
H19E-C19-H19F	109.5
C17A-O1A-H1A	109.5
C5A-O3A-C8A	106.02(18)
C14A-O4A-C19A	117.5(2)
C2A-C1A-C17A	120.39(18)
C6A-C1A-C2A	120.00(19)
C6A-C1A-C17A	119.53(18)
C1A-C2A-C18A	122.63(19)
C3A-C2A-C1A	118.18(19)
C3A-C2A-C18A	119.13(19)
C2A-C3A-H3A	118.8
C4A-C3A-C2A	122.4(2)
C4A-C3A-H3A	118.8
C3A-C4A-H4A	121.1
C5A-C4A-C3A	117.7(2)
C5A-C4A-H4A	121.1
O3A-C5A-C6A	112.7(2)
C4A-C5A-O3A	124.7(2)
C4A-C5A-C6A	122.6(2)
C1A-C6A-C7A	133.4(2)
C5A-C6A-C1A	119.05(19)
C5A-C6A-C7A	107.52(19)
C6A-C7A-H7AA	111.4
C6A-C7A-H7AB	111.4
C6A-C7A-C8A	101.7(2)
H7AA-C7A-H7AB	109.3
C8A-C7A-H7AA	111.4
C8A-C7A-H7AB	111.4
O3A-C8A-C7A	106.38(19)
O3A-C8A-H8A	107.3
O3A-C8A-H8AA	106.8
O3A-C8A-C9A	118.7(3)
O3A-C8A-C9B	98.4(3)
C7A-C8A-H8A	107.3
C7A-C8A-H8AA	106.8
C7A-C8A-C9A	109.3(3)
C9A-C8A-H8A	107.3
C9B-C8A-C7A	129.8(4)
C9B-C8A-H8AA	106.8
C8A-C9A-H9AA	110.0
C8A-C9A-H9AB	110.0
H9AA-C9A-H9AB	108.4

C10A-C9A-C8A	108.3(5)
C10A-C9A-H9AA	110.0
C10A-C9A-H9AB	110.0
C9A-C10A-H10A	107.9
C9A-C10A-H10B	107.9
C9A-C10A-C11A	117.5(5)
H10A-C10A-H10B	107.2
C11A-C10A-H10A	107.9
C11A-C10A-H10B	107.9
C12A-C11A-C10A	116.5(5)
C12A-C11A-C16A	119.2(5)
C16A-C11A-C10A	124.3(4)
C11A-C12A-H12A	121.3
C13A-C12A-C11A	117.4(6)
C13A-C12A-H12A	121.3
C12A-C13A-H13A	116.6
C12A-C13A-C14A	126.9(4)
C14A-C13A-H13A	116.6
C14A-C13A-H13B	123.0
C14A-C13A-C12B	114.0(3)
C12B-C13A-H13B	123.0
O4A-C14A-C13A	115.9(2)
O4A-C14A-C15A	124.5(2)
C15A-C14A-C13A	119.6(2)
C14A-C15A-H15A	123.6
C14A-C15A-H15B	118.3
C14A-C15A-C16A	112.7(3)
C14A-C15A-C16B	123.4(3)
C16A-C15A-H15A	123.6
C16B-C15A-H15B	118.3
C11A-C16A-C15A	122.6(4)
C11A-C16A-H16A	118.7
C15A-C16A-H16A	118.7
O1A-C17A-C1A	114.52(17)
O2A-C17A-O1A	123.82(19)
O2A-C17A-C1A	121.65(18)
C2A-C18A-H18A	109.5
C2A-C18A-H18B	109.5
C2A-C18A-H18C	109.5
H18A-C18A-H18B	109.5
H18A-C18A-H18C	109.5
H18B-C18A-H18C	109.5
O4A-C19A-H19A	109.5
O4A-C19A-H19B	109.5
O4A-C19A-H19C	109.5
H19A-C19A-H19B	109.5

H19A-C19A-H19C	109.5
H19B-C19A-H19C	109.5
H10C-C10B-H10D	108.0
C11B-C10B-H10C	109.3
C11B-C10B-H10D	109.3
C9B-C10B-H10C	109.3
C9B-C10B-H10D	109.3
C9B-C10B-C11B	111.5(5)
C12B-C11B-C10B	124.1(5)
C16B-C11B-C10B	121.0(5)
C16B-C11B-C12B	114.9(5)
C13A-C12B-H12B	117.0
C11B-C12B-C13A	125.9(6)
C11B-C12B-H12B	117.0
C15A-C16B-H16B	120.1
C11B-C16B-C15A	119.9(5)
C11B-C16B-H16B	120.1
C8A-C9B-H9BA	110.4
C8A-C9B-H9BB	110.4
C10B-C9B-C8A	106.9(5)
C10B-C9B-H9BA	110.4
C10B-C9B-H9BB	110.4
H9BA-C9B-H9BB	108.6

Symmetry transformations used to generate equivalent atoms:

Supplementary Table Supplementary Table 7. Anisotropic displacement parameters ($Å^2$ x 10^3) for sa4680. The anisotropic displacement factor exponent takes the form: -2 ^{2}[h^2 $a^{*2}U^{11}$ + ... + 2 h k a* b* U^{12}]

	U^{11}	U^{22}	U^{33}	U^{23}	U^{13}	U^{12}
O1	26(1)	32(1)	51(1)	15(1)	7(1)	9(1)
O2	28(1)	29(1)	45(1)	11(1)	5(1)	3(1)
O3	30(1)	23(1)	48(1)	5(1)	-2(1)	3(1)
O4	39(1)	38(1)	54(1)	2(1)	12(1)	-9(1)
C1	26(1)	25(1)	30(1)	3(1)	4(1)	5(1)
C2	28(1)	28(1)	31(1)	2(1)	4(1)	1(1)
C3	25(1)	34(1)	34(1)	1(1)	-2(1)	6(1)
C4	30(1)	30(1)	39(1)	5(1)	2(1)	9(1)
C5	28(1)	22(1)	36(1)	2(1)	5(1)	4(1)

C6	26(1)	26(1)	28(1)	2(1)	1(1)	4(1)
C7	27(1)	28(1)	41(1)	6(1)	-3(1)	2(1)
C8	30(1)	32(1)	41(1)	3(1)	-2(1)	1(1)
C9	30(1)	27(1)	42(1)	3(1)	2(1)	1(1)
C10	36(1)	29(1)	80(2)	-1(1)	16(1)	0(1)
C11	31(1)	27(1)	47(1)	3(1)	4(1)	2(1)
C12	39(1)	26(1)	59(1)	-4(1)	9(1)	4(1)
C13	35(1)	36(1)	43(1)	1(1)	9(1)	5(1)
C14	31(1)	31(1)	35(1)	7(1)	1(1)	-1(1)
C15	36(1)	26(1)	37(1)	1(1)	1(1)	2(1)
C16	29(1)	30(1)	36(1)	1(1)	3(1)	5(1)
C17	26(1)	23(1)	36(1)	1(1)	1(1)	2(1)
C18	33(1)	33(1)	52(1)	3(1)	-6(1)	-3(1)
C19	39(1)	57(2)	56(2)	6(1)	14(1)	-8(1)
O1A	31(1)	44(1)	52(1)	-11(1)	-2(1)	10(1)
O2A	32(1)	38(1)	46(1)	-9(1)	-1(1)	7(1)
O3A	62(1)	27(1)	65(1)	6(1)	-14(1)	-3(1)
O4A	82(1)	51(1)	63(1)	23(1)	-29(1)	-24(1)
C1A	29(1)	30(1)	29(1)	0(1)	-3(1)	4(1)
C2A	29(1)	33(1)	34(1)	2(1)	-1(1)	3(1)
C3A	31(1)	44(1)	38(1)	-3(1)	0(1)	10(1)
C4A	40(1)	37(1)	47(1)	-6(1)	-6(1)	16(1)
C5A	42(1)	26(1)	42(1)	3(1)	-12(1)	4(1)
C6A	31(1)	34(1)	31(1)	4(1)	-3(1)	2(1)
C7A	40(1)	48(1)	47(1)	8(1)	2(1)	-8(1)
C8A	74(2)	53(2)	51(2)	15(1)	-5(1)	-23(1)
C9A	39(3)	28(3)	70(4)	4(3)	3(3)	3(2)
C10A	39(3)	35(3)	103(4)	7(3)	-4(3)	0(2)
C11A	34(2)	28(2)	55(3)	-8(2)	9(2)	6(2)
C12A	32(3)	53(4)	43(3)	5(3)	3(2)	16(2)
C13A	47(1)	58(2)	34(1)	10(1)	4(1)	15(1)
C14A	37(1)	38(1)	32(1)	4(1)	3(1)	2(1)
C15A	48(1)	42(1)	57(2)	12(1)	-12(1)	4(1)
C16A	50(3)	30(3)	58(3)	10(2)	10(2)	11(2)
C17A	28(1)	31(1)	34(1)	0(1)	1(1)	3(1)
C18A	38(1)	39(1)	51(1)	0(1)	5(1)	-3(1)
C19A	68(2)	95(3)	64(2)	21(2)	-24(2)	-37(2)
C10B	47(3)	42(3)	52(3)	-2(2)	8(2)	6(2)
C11B	42(3)	39(3)	40(3)	6(2)	8(2)	1(2)
C12B	23(3)	57(4)	41(3)	12(3)	6(2)	14(3)
C16B	51(4)	32(3)	57(4)	11(2)	-1(3)	9(2)
C9B	53(4)	30(4)	47(3)	3(3)	8(3)	0(3)

Supplementary Table 8. Hydrogen coordinates (x 10^4) and isotropic

displacement parameters (Å^{2}x 10 ^{3})
for sa4680.

	x	y	z	U(eq)
H1	3939	9508	5480	53
H3	10529	7758	7036	38
H4	8825	6225	6951	39
H7A	3466	7193	5181	39
H7B	2891	7160	6153	39
H8	4023	5693	5126	42
H9A	966	5399	5635	40
H9B	1817	5431	6580	40
H10E	3185	4100	6266	58
H10F	2539	4094	5296	58
H12	-1011	4350	6722	50
H13	-3557	3187	6960	45
H15	-1135	1251	5636	40
H16	1358	2413	5408	38
H18D	10280	9563	5934	61
H18E	10903	9394	6884	61
H18F	9129	9874	6702	61
H19D	-6114	2046	6613	78
H19E	-5166	1763	7467	78
H19F	-6482	984	6888	78
H1A	8889	9443	9490	63
H3A	15514	7832	7907	45
H4A	13976	6268	7890	49
H7AA	8004	7031	8698	56
H7AB	8724	7113	9676	56
H8A	9536	5672	9632	75
H8AA	9556	5681	9632	75
H9AA	6280	5456	9241	55
H9AB	6761	5335	8267	55
H10A	7857	4151	9538	71
H10B	8141	4008	8553	71
H12A	6507	2522	9598	50
H13A	3885	1544	9595	55
H13B	4189	1499	9456	55
H15A	1012	3149	8197	60
H15B	1241	2996	7974	60
H16A	3979	4184	8084	54

H18A	13828	9893	8352	65
H18B	15618	9476	8087	65
H18C	15253	9659	9064	65
H19A	-466	1592	7681	122
H19B	-1323	1914	8537	122
H19C	-1536	824	8256	122
H10C	5525	5354	9383	56
H10D	6812	4802	9941	56
H12B	6279	2971	9765	47
H16B	2877	4449	8521	55
H9BA	7157	5108	8164	52
H9BB	8302	4415	8644	52

Supplementary Table 9. Torsion angles [°] for sa4680.

O3-C5-C6-C1	-178.37(16)
O3-C5-C6-C7	3.0(2)
O3-C8-C9-C10	-62.9(2)
O4-C14-C15-C16	179.84(18)
C1-C2-C3-C4	1.4(3)
C1-C6-C7-C8	166.2(2)
C2-C1-C6-C5	-3.0(3)
C2-C1-C6-C7	175.3(2)
C2-C1-C17-O1	-138.99(19)
C2-C1-C17-O2	42.8(3)
C2-C3-C4-C5	-1.8(3)
C3-C4-C5-O3	-179.13(18)
C3-C4-C5-C6	-0.3(3)
C4-C5-C6-C1	2.7(3)
C4-C5-C6-C7	-176.01(18)
C5-O3-C8-C7	-21.0(2)
C5-O3-C8-C9	-147.88(17)
C5-C6-C7-C8	-15.4(2)
C6-C1-C2-C3	1.0(3)
C6-C1-C2-C18	-178.23(19)
C6-C1-C17-O1	44.9(2)
C6-C1-C17-O2	-133.4(2)
C6-C7-C8-O3	21.8(2)
C6-C7-C8-C9	143.42(18)
C7-C8-C9-C10	177.0(2)
C8-O3-C5-C4	-169.51(19)
C8-O3-C5-C6	11.5(2)
C8-C9-C10-C11	-174.3(2)
C9-C10-C11-C12	-19.1(4)
C9-C10-C11-C16	162.0(2)

C10-C11-C12-C13	-178.5(2)
C10-C11-C16-C15	178.6(2)
C11-C12-C13-C14	0.1(4)
C12-C11-C16-C15	-0.3(3)
C12-C13-C14-O4	-179.8(2)
C12-C13-C14-C15	-0.4(3)
C13-C14-C15-C16	0.4(3)
C14-C15-C16-C11	0.0(3)
C16-C11-C12-C13	0.3(3)
C17-C1-C2-C3	-175.07(18)
C17-C1-C2-C18	5.7(3)
C17-C1-C6-C5	173.16(17)
C17-C1-C6-C7	-8.6(3)
C18-C2-C3-C4	-179.29(19)
C19-O4-C14-C13	0.2(3)
C19-O4-C14-C15	-179.2(2)
O3A-C5A-C6A-C1A	179.64(17)
O3A-C5A-C6A-C7A	-1.5(2)
O3A-C8A-C9A-C10A	67.6(6)
O3A-C8A-C9B-C10B	166.2(4)
O4A-C14A-C15A-C16A	-166.3(3)
O4A-C14A-C15A-C16B	161.4(4)
C1A-C2A-C3A-C4A	-1.0(3)
C1A-C6A-C7A-C8A	-166.4(2)
C2A-C1A-C6A-C5A	3.1(3)
C2A-C1A-C6A-C7A	-175.5(2)
C2A-C1A-C17A-O1A	139.9(2)
C2A-C1A-C17A-O2A	-41.3(3)
C2A-C3A-C4A-C5A	1.6(3)
C3A-C4A-C5A-O3A	177.8(2)
C3A-C4A-C5A-C6A	0.1(3)
C4A-C5A-C6A-C1A	-2.4(3)
C4A-C5A-C6A-C7A	176.5(2)
C5A-O3A-C8A-C7A	22.8(3)
C5A-O3A-C8A-C9A	146.4(4)
C5A-O3A-C8A-C9B	158.6(3)
C5A-C6A-C7A-C8A	14.9(2)
C6A-C1A-C2A-C3A	-1.4(3)
C6A-C1A-C2A-C18A	176.05(19)
C6A-C1A-C17A-O1A	-43.3(3)
C6A-C1A-C17A-O2A	135.5(2)
C6A-C7A-C8A-O3A	-22.8(3)
C6A-C7A-C8A-C9A	-152.1(4)
C6A-C7A-C8A-C9B	-139.1(4)
C7A-C8A-C9A-C10A	-170.2(4)
C7A-C8A-C9B-C10B	-74.2(6)

C8A-O3A-C5A-C4A	168.5(2)
C8A-O3A-C5A-C6A	-13.6(2)
C8A-C9A-C10A-C11A	174.9(5)
C9A-C10A-C11A-C12A	-155.1(6)
C9A-C10A-C11A-C16A	24.2(9)
C10A-C11A-C12A-C13A	176.7(5)
C10A-C11A-C16A-C15A	-173.0(5)
C11A-C12A-C13A-C14A	5.7(7)
C12A-C11A-C16A-C15A	6.4(8)
C12A-C13A-C14A-O4A	167.9(4)
C12A-C13A-C14A-C15A	-12.2(5)
C13A-C14A-C15A-C16A	13.8(4)
C13A-C14A-C15A-C16B	-18.5(5)
C14A-C13A-C12B-C11B	-0.5(8)
C14A-C15A-C16A-C11A	-11.8(6)
C14A-C15A-C16B-C11B	16.7(8)
C16A-C11A-C12A-C13A	-2.7(8)
C17A-C1A-C2A-C3A	175.42(18)
C17A-C1A-C2A-C18A	-7.1(3)
C17A-C1A-C6A-C5A	-173.80(18)
C17A-C1A-C6A-C7A	7.6(3)
C18A-C2A-C3A-C4A	-178.5(2)
C19A-O4A-C14A-C13A	178.2(3)
C19A-O4A-C14A-C15A	-1.7(4)
C10B-C11B-C12B-C13A	179.7(5)
C10B-C11B-C16B-C15A	172.9(5)
C11B-C10B-C9B-C8A	-171.4(4)
C12B-C13A-C14A-O4A	-170.1(4)
C12B-C13A-C14A-C15A	9.8(4)
C12B-C11B-C16B-C15A	-6.3(9)
C16B-C11B-C12B-C13A	-1.1(9)
C9B-C10B-C11B-C12B	86.7(7)
C9B-C10B-C11B-C16B	-92.4(7)

Symmetry transformations used to generate equivalent atoms:

Supplementary Table 10. Hydrogen bonds for sa4680 [Å and °].

D-H...A	d(D-H)	d(H...A)	d(D...A)	<(DHA)
O1-H1...O2#1	0.82	1.84	2.657(2)	177.0
O1A-H1A...O2A#2	0.82	1.85	2.665(2)	177.4

Symmetry transformations used to generate equivalent atoms:

#1 -x+1,-y+2,-z+1 #2 -x+2,-y+2,-z+2

Methyl2-(4-fluorophenethyl)-5-methyl-2,3-dihydrobenzofuran-4-carboxylate

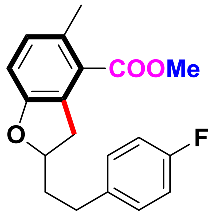

2n

2n was obtained as a yellow oil (57.5 mg, 61%). ^{1}H NMR (400 MHz, $CDCl_3$) δ 7.17 (dd, J = 8.1, 5.6 Hz, 2H), 7.02 – 6.93 (m, 3H), 6.79 (d, J = 8.1 Hz, 1H), 4.80 – 4.69 (m, 1H), 3.88 (s, 3H), 3.47 (dd, J = 16.8, 9.0 Hz, 1H), 3.05 (dd, J = 16.8, 7.7 Hz, 1H), 2.86 – 2.69 (m, 2H), 2.43 (s, 3H), 2.14 – 2.03 (m, 1H), 1.99 – 1.88 (m, 1H). ^{13}C NMR (100 MHz, $CDCl_3$) δ 168.17, 161.34 (d, J = 243.6 Hz), 157.90, 137.04 (d, J = 3.3 Hz), 131.02, 130.39, 129.81 (d, J = 7.8 Hz), 128.51, 127.41, 115.19 (d, J = 21.1 Hz), 112.09, 82.40, 51.65, 37.93, 36.91, 30.90, 20.83. HRMS (ESI-TOF) Calcd for $C_{19}H_{19}FO_3$ $[M]^+$: 314.1318; found: 314.1315.

Methyl2-(4-chlorophenethyl)-5-methyl-2,3-dihydrobenzofuran-4-carboxylate

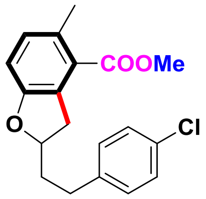

2o

2o was obtained as a yellow oil (55.5 mg, 56%). ^{1}H NMR (400 MHz, $CDCl_3$) δ 7.29 – 7.23 (m, 2H), 7.15 (d, J = 8.2 Hz, 2H), 6.99 (d, J = 8.1 Hz, 1H), 6.79 (d, J = 8.1 Hz, 1H), 4.80 – 4.68 (m, 1H), 3.89 (d, J = 10.9 Hz, 3H), 3.47 (dd, J = 16.8, 9.0 Hz, 1H), 3.04 (dd, J = 16.8, 7.7 Hz, 1H), 2.87 – 2.67 (m, 2H), 2.43 (s, 3H), 2.16 – 2.03 (m, 1H), 2.00 – 1.86 (m, 1H). ^{13}C NMR (100 MHz, $CDCl_3$) δ 168.14, 157.87, 139.88, 131.72, 131.03, 130.42, 129.84, 128.55, 128.46, 127.42, 112.09, 82.33, 51.64, 37.69, 36.90, 31.05, 20.81. HRMS (ESI) Calcd for $C_{19}H_{20}ClO_3$ $[M+H]^+$: 331.1091; found: 331.1095.

Methyl2-(4-bromophenethyl)-5-methyl-2,3-dihydrobenzofuran-4-carboxylate

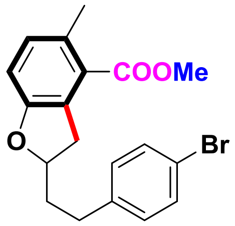

2p

2p was obtained as a yellow oil (64.8 mg, 60%). ^{1}H NMR (400 MHz, $CDCl_3$) δ 7.40 (d, J = 8.3 Hz, 2H), 7.09 (d, J = 8.3 Hz, 2H), 6.98 (d, J = 8.1 Hz, 1H), 6.78 (d, J = 8.1 Hz, 1H), 4.74 (qd, J = 7.9, 5.1 Hz, 1H), 3.88 (d, J = 4.4 Hz, 3H), 3.50 – 3.41 (m, 1H), 3.08 – 2.99 (m, 1H), 2.84 – 2.68 (m, 2H), 2.43 (s, 3H), 2.13 – 2.03 (m, 1H),

1.98 – 1.87 (m, 1H). ^{13}C NMR (100 MHz, $CDCl_3$) δ 168.14, 157.88, 140.42, 131.50, 131.04, 130.44, 130.27, 128.47, 127.41, 119.74, 112.10, 82.32, 51.65, 37.63, 36.92, 31.13, 20.84. HRMS (ESI) Calcd for $C_{18}H_{18}BrO_3$ $[M+H]^+$: 361.0442; found: 361.0434.

Methyl5-methyl-2-(4-(trifluoromethyl)phenethyl)-2,3-dihydrobenzofuran-4-carboxylate

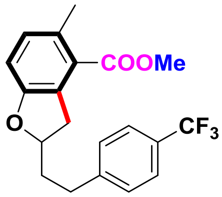

2q

2q was obtained as a yellow oil (77.6 mg, 71%). ^{1}H NMR (400 MHz, $CDCl_3$) δ 7.55 (d, J = 8.0 Hz, 2H), 7.34 (d, J = 7.9 Hz, 2H), 7.00 (d, J = 8.1 Hz, 1H), 6.80 (d, J = 8.1 Hz, 1H), 4.82 – 4.69 (m, 1H), 3.88 (s, 3H), 3.49 (dd, J = 16.8, 9.0 Hz, 1H), 3.06 (dd, J = 16.8, 7.6 Hz, 1H), 2.98 – 2.77 (m, 2H), 2.44 (s, 3H), 2.12 (td, J = 14.0, 8.7 Hz, 1H), 2.05 – 1.91 (m, 1H). ^{13}C NMR (100 MHz, $CDCl_3$) δ 168.12, 157.84, 145.61, 131.07, 130.49, 128.80, 128.39 (dd, J = 64.6, 32.3 Hz), 128.39, 127.43, 125.38 (q, J = 3.8 Hz), 124.33 (q, J = 3.0 Hz), 112.11, 82.24, 51.63, 37.47, 36.90, 31.56 , 20.81. HRMS (ESI-TOF) Calcd for $C_{20}H_{19}F_3O_3$ $[M]^+$: 364.1286; found: 364.1287.

Methyl 5-methyl-2,3-dihydrobenzofuran-4-carboxylate

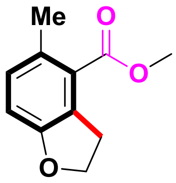

2r

2r was obtained as a yellow oil (9.8 mg, 17%). ^{1}H NMR (400 MHz, $CDCl_3$) δ 6.98 (d, J = 8.1 Hz, 1H), 6.79 (d, J = 8.1 Hz, 1H), 4.55 (t, J = 8.8 Hz, 2H), 3.89 (s, 3H), 3.39 (t, J = 8.7 Hz, 2H), 2.43 (s, 3H). ^{13}C NMR (100 MHz, $CDCl_3$) δ 168.17, 158.47, 130.89, 130.41, 128.60, 127.48, 112.02, 71.34, 51.59, 31.21, 20.69. HRMS (ESI) Calcd for $C_{11}H_{13}O_3$ $[M+H]^+$: 193.0854; found: 193.0859.

4-nitrobenzyl(9aR)-2-methyl-5a,6,7,8,9,9a-hexahydrodibenzo[*b*,*d*]furan-1-carboxylate

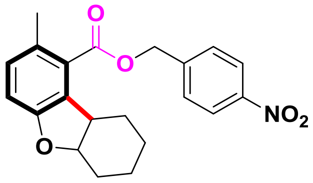

2s

2s was obtained as a yellow oil (35.2 mg, 32%). ^{1}H NMR (400 MHz, $CDCl_3$) δ 8.33 – 8.19 (m, 2H), 7.62 (d, J = 8.7 Hz, 2H), 6.99 (d, J = 8.1 Hz, 1H), 6.85 (d, J = 8.1 Hz, 1H), 5.43 (s, 2H), 4.55 (dd, J = 5.6, 3.1 Hz, 1H), 3.25 (dt, J = 11.9, 6.2 Hz, 1H), 2.40 (s, 3H), 2.25 (d, J = 16.1 Hz, 1H), 1.88 – 1.39 (m, 6H), 1.18 – 0.95 (m, 2H). ^{13}C NMR (100 MHz, $CDCl_3$) δ 167.33, 157.73, 147.88, 142.92, 136.09, 130.63, 130.13, 129.07, 126.33, 123.90, 113.11, 82.85, 77.35, 77.03, 76.71, 65.27, 41.58, 29.10, 27.22, 22.93,

20.78, 20.26. HRMS (ESI) Calcd for $C_{21}H_{22}NO_5$ $[M+H]^+$: 368.1483; found: 368.1492.

Methyl(3R)-2-isobutyl-5-methyl-3-phenyl-2,3-dihydrobenzofuran-4-carboxylate

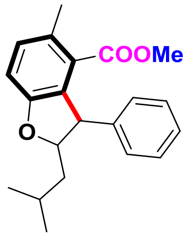

2t

2t was obtained as a yellow oil (83.6 mg, 86%). ^{1}H NMR (400 MHz, $CDCl_3$) δ 7.31 – 7.14 (m, 2H), 7.07 (dt, *J* = 15.8, 6.9 Hz, 1H), 6.93 – 6.81 (m, 1H), 3.94 – 3.84 (m, 0H), 3.53 (s, 1H), 3.34 (s, 1H), 2.36 (s, 1H), 2.31 (s, 1H), 2.00 – 1.70 (m, 1H), 1.62 – 1.49 (m, 1H), 1.37 – 1.19 (m, 1H), 0.96 – 0.79 (m, 4H). ^{13}C NMR (100 MHz, $CDCl_3$) δ 168.27, 167.79, 167.69, 158.20, 158.18, 156.61, 152.51, 142.57, 139.28, 133.32, 132.33, 131.37, 131.21, 130.47, 130.33, 130.06, 129.70, 129.58, 129.29, 129.03, 128.84, 128.60, 128.45, 128.38, 128.11, 127.88, 127.08, 126.86, 126.78, 126.46, 125.73, 117.56, 112.25, 111.77, 90.77, 85.66, 55.57, 52.29, 51.28, 51.21, 51.02, 44.41, 40.20, 35.42, 29.72, 27.97, 25.21, 24.93, 24.67, 23.28, 23.23, 22.44, 22.30, 22.10, 22.06, 20.18, 19.57, 19.15. HRMS (ESI) Calcd for $C_{21}H_{25}O_3$ $[M+H]^+$: 325.1799; found: 325.1798.

Methyl 5-ethoxy-2-methyl-2, 3-dihydrobenzofuran-4-carboxylate

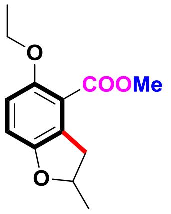

2u

2u was obtained as a yellow oil (44.1 mg, 62%). ^{1}H NMR (400 MHz, $CDCl_3$) δ 6.77 (d, *J* = 8.7 Hz, 1H), 6.73 (d, *J* = 8.7 Hz, 1H), 4.97 – 4.84 (m, 1H), 4.01 (q, *J* = 6.9 Hz, 2H), 3.89 (s, 3H), 3.45 (dd, *J* = 16.7, 8.7 Hz, 1H), 2.94 (dd, *J* = 16.7, 7.8 Hz, 1H), 1.45 (d, *J* = 6.2 Hz, 3H), 1.38 (t, *J* = 7.0 Hz, 3H). ^{13}C NMR (100 MHz, $CDCl_3$) δ 166.81, 153.78, 151.90, 129.10, 119.46, 114.39, 112.05, 79.93, 66.37, 51.84, 38.08, 21.70, 14.98. HRMS (ESI) Calcd for $C_{13}H_{17}O_4$ [M+H]+: 237.1124; found: 237.1121.

Methyl5-isopropoxy-2-methyl-2, 3-dihydrobenzofuran-4-carboxylate

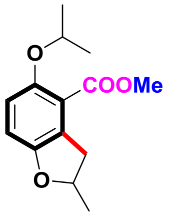

2v

2v was obtained as a yellow oil (53.3 mg, 71%). ^{1}H NMR (400 MHz, $CDCl_3$) δ 6.76 (s, 2H), 4.97 – 4.84 (m, 1H), 4.32 (m, *J* = 6.0 Hz, 1H), 3.44 (dd, *J* = 16.6, 8.8 Hz, 1H), 2.94 (dd, *J* = 16.6, 7.9 Hz, 1H), 1.45 (d, *J* = 6.2 Hz, 3H), 1.29 (d, *J* = 6.1 Hz, 6H). ^{13}C NMR (100 MHz, $CDCl_3$) δ 167.00, 154.09, 150.61, 128.82, 121.13, 117.75, 112.02,

80.00, 74.01, 51.81, 38.00, 22.18, 21.73. HRMS (ESI) Calcd for $C_{14}H_{19}O_4$ $[M+H]^+$: 251.1271; found: 251.1278.

Methyl 5-fluoro-2-methyl-2, 3-dihydrobenzofuran-4-carboxylate

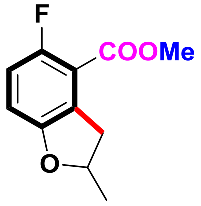

2w

2w was obtained as a yellow oil (47.3 mg, 75%). ^{1}H NMR (400 MHz, $CDCl_3$) δ 6.88 (dd, *J* = 10.6, 8.8 Hz, 1H), 6.80 (dd, *J* = 8.7, 3.9 Hz, 1H), 4.96 (ddd, *J* = 8.8, 7.8, 6.3 Hz, 1H), 3.91 (s, 3H), 3.58 (dd, *J* = 17.3, 8.9 Hz, 1H), 3.06 (dd, *J* = 17.3, 7.7 Hz, 1H), 1.46 (d, *J* = 6.3 Hz, 3H). ^{13}C NMR (100 MHz, $CDCl_3$) δ 164.96, 164.93, 157.21, 155.69, 155.67, 154.73, 130.63, 130.61, 115.87, 115.61, 113.35, 113.26, 80.54, 52.15, 38.54, 38.52, 21.74. HRMS (ESI) Calcd for $C_{11}H_{12}FO_3$ $[M+H]^+$: 211.0765; found: 211.0765.

Methyl 5-chloro-2-methyl-2, 3-dihydrobenzofuran-4-carboxylate

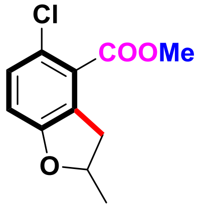

2x

2x was obtained as a yellow oil (50.9 mg, 75%). ^{1}H NMR (400 MHz, $CDCl_3$) δ 7.16 (d, *J* = 8.5 Hz, 1H), 6.75 (d, *J* = 8.5 Hz, 1H), 4.96 (dd, *J* = 14.2, 7.7 Hz, 1H), 3.92 (s, 3H), 3.44 (dd, *J* = 16.6, 8.9 Hz, 1H), 2.94 (dd, *J* = 16.6, 7.7 Hz, 1H), 1.46 (d, *J* = 6.2 Hz, 3H). ^{13}C NMR (100 MHz, $CDCl_3$) δ 166.20, 158.41, 130.07, 129.47, 127.94, 122.98, 112.46, 80.56, 52.31, 37.64, 21.70. HRMS (ESI) Calcd for $C_{11}H_{12}ClO_3$ $[M+H]^+$: 227.0468; found: 227.0469.

Methyl2,2,6,6-tetramethyl-2,3,5,6-tetrahydrobenzo[1,2-b:5,4-b']difuran-4-carboxylate

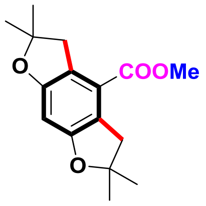

2y

2y was obtained as a yellow oil (58.8 mg, 71%). ^{1}H NMR (400 MHz, $CDCl_3$) δ 6.28 (s, 1H), 3.78 (s, 3H), 3.15 (s, 4H), 1.38 (s, 12H). ^{13}C NMR (100 MHz, $CDCl_3$) δ 156.73, 129.17, 128.28, 127.10, 125.77, 109.05, 86.41, 42.96, 28.21, 20.78. HRMS (ESI) Calcd for $C_{16}H_{21}O_4$ $[M+H]^+$: 277.1437; found: 277.1434.

(1R,3aS,3bR,6aS,12aS,12bS,14aR)-4-nitrobenzyl 10,12a,14a-trimethyl-1-((R)-6-methylheptan-2-yl)-2,3,3a,3b,4,5,5a,6,6a,11b,12,12a,12b,13,14,14a-hexadecahydro-1H-cyclopenta[7,8]phenanthro[2,3-b]benzofuran-11-carboxylate

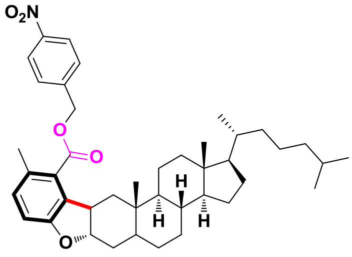

13

13 was obtained as a yellow oil (44% NMR yield. The reaction was performed according to the standard procedure for 24 h, and another bath of $Pd(OAc)_2$ (6.7 mg, 10 mol%) was added into the above mixture. The reaction was stirred at 140 ^oC for another 24 h.), ^1H NMR (400 MHz, $CDCl_3$) δ 8.26 (d, *J* = 8.7 Hz, 2H), 7.65 (d, *J* = 8.7 Hz, 2H), 6.99 (d, *J* = 8.2 Hz, 1H), 6.83 (d, *J* = 8.1 Hz, 1H), 5.50 – 5.33 (m, 2H), 4.52 (t, *J* = 4.0 Hz, 1H), 3.38 – 3.23 (m, 1H), 2.43 (s, 3H), 1.98 – 1.89 (m, 1H), 1.84 – 1.60 (m, 5H), 1.55 – 1.47 (m, 2H), 1.44 – 0.95 (m, 19H), 0.90 (d, *J* = 6.6 Hz, 4H), 0.86 (dd, *J* = 6.6, 1.4 Hz, 6H), 0.60 (d, *J* = 3.1 Hz, 6H), 0.53 – 0.44 (m, 1H). ^{13}C NMR (100 MHz, $CDCl_3$) δ 167.25, 157.33, 147.94, 142.78, 136.56, 130.63, 130.55, 129.73, 126.09, 123.99, 113.04, 82.83, 65.28, 56.39, 56.15, 53.51, 42.34, 41.58, 39.73, 39.51, 39.27, 39.17, 36.16, 35.78, 35.38, 34.66, 31.64, 30.79, 28.45, 28.22, 28.02, 24.18, 23.79, 22.83, 22.57, 20.88, 20.83, 18.59, 11.86, 10.82. HRMS (ESI) Calcd for $C_{42}H_{58}NO_5$ $[M+H]^+$: 656.4315; found: 656.4323.

(S)-methyl 5-methyl-2-(2-(((8R,9S,13S,14S)-13-methyl-17-oxo-7,8,9,11,12,13,14,15,16,17-decahydro-6H-cyclopenta[a]phenanthren-3-yl)oxy)ethyl)-2,3-dihydrobenzofuran-4-carboxylate

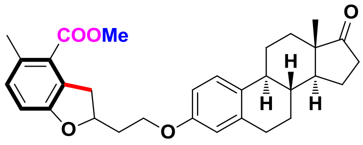

16

16 was obtained as a yellow oil (52% NMR yield. With 20 mol% $Pd(OAc)_2$). ^1H NMR (400 MHz, $CDCl_3$) δ 7.20 (d, *J* = 8.6 Hz, 1H), 6.98 (d, *J* = 8.2 Hz, 1H), 6.77 (d, *J* = 8.1 Hz, 1H), 6.75 – 6.71 (m, 1H), 6.67 (d, *J* = 2.6 Hz, 1H), 5.01 (ddd, *J* = 16.8, 8.1, 5.0 Hz, 1H), 4.24 – 4.08 (m, 2H), 3.89 (d, *J* = 3.9 Hz, 3H), 3.57 – 3.48 (m, 1H), 3.12 (dd, *J* = 16.8, 7.7 Hz, 1H), 2.90 (dd, *J* = 10.5, 4.5 Hz, 2H), 2.54 – 2.46 (m, 1H), 2.43 (s, 3H), 2.26 – 1.95 (m, 7H), 1.63 – 1.47 (m, 7H), 0.91 (s, 3H). ^{13}C NMR (100 MHz, $CDCl_3$) δ 220.99, 168.12, 157.76, 156.84, 137.79, 132.19, 131.00, 130.48, 128.41, 127.48, 126.37, 114.56, 112.19, 112.12, 80.27, 64.26, 51.62, 50.43, 48.03, 44.00, 38.39, 37.04, 35.89, 35.85, 31.60, 29.67, 26.56, 25.94, 21.60, 20.75, 13.87. HRMS (ESI) Calcd for $C_{31}H_{37}O_5$ $[M+H]^+$: 489.2641; found: 489.2636.

NMR Data of Further Transformations of Corresponding Products

Phenyl 2, 2, 5-trimethyl-2, 3-dihydrobenzofuran-4-carboxylate

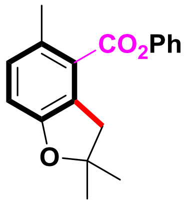

3

3, ^{1}H NMR (400 MHz, $CDCl_3$) δ 7.45 (dd, *J* = 11.0, 4.8 Hz, 2H), 7.29 (d, *J* = 7.5 Hz, 1H), 7.24 – 7.17 (m, 2H), 7.06 (d, *J* = 8.1 Hz, 1H), 6.83 (d, *J* = 8.1 Hz, 1H), 3.35 (s, 2H), 2.54 (s, 3H), 1.49 (s, 6H). ^{13}C NMR (100 MHz, $CDCl_3$) δ 166.01, 157.49, 150.61, 131.32, 131.05, 129.57, 129.41, 126.56, 125.97, 121.75, 113.09, 86.83, 77.35, 77.03, 76.71, 44.71, 28.29, 21.08. HRMS (ESI) Calcd for $C_{18}H_{19}O_3$ $[M+H]^+$: 283.1330; found: 283.1329.

Prop-2-yn-1-yl-2-(4-methoxybenzyl)-5-methyl-2,3-dihydrobenzofuran-4-carboxylate

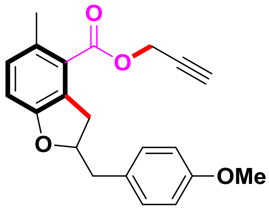

4

4, ^{1}H NMR (400 MHz, $CDCl_3$) δ 7.13 (d, *J* = 8.5 Hz, 2H), 6.97 (d, *J* = 8.1 Hz, 1H), 6.81 (dd, *J* = 11.1, 8.4 Hz, 3H), 4.87 (d, *J* = 2.2 Hz, 2H), 4.81 – 4.68 (m, 1H), 3.76 (s, 3H), 3.50 (dd, *J* = 16.9, 9.0 Hz, 1H), 3.08 (dd, *J* = 16.9, 7.8 Hz, 1H), 2.84 – 2.63 (m, 2H), 2.50 (t, *J* = 2.3 Hz, 1H), 2.45 (s, 3H), 2.14 – 2.01 (m, 1H), 2.00 – 1.83 (m, 1H). ^{13}C NMR (100 MHz, $CDCl_3$) δ 166.67, 158.10, 157.93, 133.47, 131.10, 130.76, 129.38, 129.03, 126.44, 113.90, 112.53, 82.64, 75.06, 55.27, 51.96, 38.05, 37.04, 30.80, 20.85. HRMS (ESI) Calcd for $C_{22}H_{23}O_4$ $[M+H]^+$: 351.1589; found: 351.1591.

Methyl 2, 5-dimethylbenzofuran-4-carboxylate

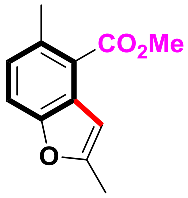

5

5, ^{1}H NMR (400 MHz, $CDCl_3$) δ 7.40 (d, *J* = 8.3 Hz, 1H), 7.07 (d, *J* = 8.3 Hz, 1H), 6.76 – 6.72 (m, 1H), 3.96 (s, 3H), 2.63 (s, 3H), 2.47 (d, *J* = 0.9 Hz, 3H). ^{13}C NMR (100 MHz, $CDCl_3$) δ 168.02, 156.98, 153.36, 134.85, 130.17, 126.44, 121.04, 113.73, 104.24, 51.53, 21.57, 14.12. HRMS (ESI) Calcd for $C_{12}H_{13}O_3$ $[M+H]^+$: 205.0854; found: 205.0859.

2,2,5-trimethyl-2,3-dihydrobenzofuran

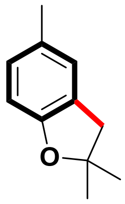

6

6, ^1H NMR (400 MHz, $CDCl_3$) δ 6.96 (s, 1H), 6.91 (d, *J* = 8.1 Hz, 1H), 6.63 (d, *J* = 8.0 Hz, 1H), 2.98 (s, 2H), 2.28 (s, 3H), 1.47 (s, 6H). ^{13}C NMR (100 MHz, $CDCl_3$) δ 156.73, 129.17, 128.28, 127.10, 125.77, 109.05, 86.41, 42.96, 28.21, 20.78. Spectroscopic data for **6** match those previously reported in the literature.[10]

Methyl 2-methyl-5-phenyl-2,3-dihydrobenzofuran-4-carboxylate

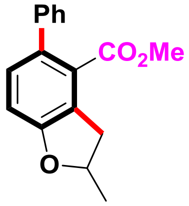

7

7, ^1H NMR (400 MHz, $CDCl_3$) δ 7.39 – 7.31 (m, 2H), 7.32 – 7.24 (m, 3H), 7.14 (d, *J* = 8.2 Hz, 1H), 6.86 (d, *J* = 8.2 Hz, 1H), 5.05 – 4.92 (m, 1H), 3.57 (s, 3H), 3.49 (dd, *J* = 16.5, 8.9 Hz, 1H), 2.98 (dd, *J* = 16.5, 7.7 Hz, 1H), 1.49 (d, *J* = 6.3 Hz, 3H). ^{13}C NMR (100 MHz, $CDCl_3$) δ 168.82, 159.16, 141.36, 133.89, 130.51, 128.39, 128.31, 128.12, 127.94, 126.75, 111.38, 80.34, 51.71, 37.23, 21.83. HRMS (ESI) Calcd for $C_{17}H_{17}O_3$ $[M+H]^+$: 269.1177; found: 269.1172.

2, 2, 5-trimethyl-2, 3-dihydrobenzofuran-4-amine

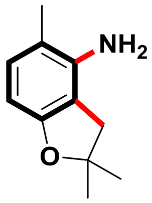

8

8, ^1H NMR (400 MHz, $CDCl_3$) δ 6.83 (dd, *J* = 7.9, 0.6 Hz, 1H), 6.17 (d, *J* = 7.9 Hz, 1H), 3.49 (s, 2H), 2.83 (s, 2H), 2.12 (s, 3H), 1.49 (s, 6H). ^{13}C NMR (100 MHz, $CDCl_3$) δ 158.04, 141.18, 129.67, 113.84, 111.05, 99.70, 86.65, 40.48, 28.51, 16.52. HRMS (ESI) Calcd for $C_{11}H_{16}NO$ $[M+H]^+$: 178.1224; found: 178.1226.

7, 7-dimethyl-7, 8-dihydrobenzo[1, 2 -b:3, 4-c']difuran-1(3H)-one

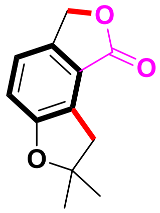

9

9, ^1H NMR (400 MHz, $CDCl_3$) δ 7.19 (d, *J* = 8.1 Hz, 1H), 7.01 (d, *J* = 8.2 Hz, 1H), 5.26 (s, 2H), 3.32 (s, 2H), 1.51 (s, 6H).^{13}C NMR (100 MHz, $CDCl_3$) δ 170.89, 160.38, 137.99, 125.73, 122.75, 121.35, 115.36, 89.01, 70.01, 40.80, 28.18. HRMS

(ESI) Calcd for $C_{12}H_{13}O_3$ $[M+H]^+$: 205.0857; found: 355.1287.

Methyl-2-(2-hydroxyethyl)-5-methyl-2,3-dihydrobenzofuran-4-carboxylate

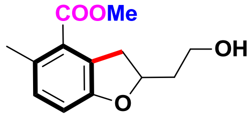

2i-1

2i-1, ^1H NMR (400 MHz, $CDCl_3$) δ 6.98 (d, *J* = 8.1 Hz, 1H), 6.77 (d, *J* = 8.1 Hz, 1H), 4.95 (ddd, *J* = 16.8, 8.7, 4.3 Hz, 1H), 3.93 – 3.83 (m, 5H), 3.52 (dd, *J* = 16.8, 9.0 Hz, 1H), 3.08 (dd, *J* = 16.8, 7.9 Hz, 1H), 2.43 (s, 3H), 2.12 – 1.88 (m, 2H). ^{13}C NMR (100 MHz, $CDCl_3$) δ 168.09, 157.57, 131.02, 130.62, 128.31, 127.49, 112.16, 81.83, 77.35, 77.03, 76.71, 60.17, 51.62, 38.54, 37.20, 20.73. HRMS (ESI) Calcd for $C_{13}H_{17}O_4$ $[M+H]^+$: 237.1117; found: 237.1121.

Methyl (R)-2-(2-hydroxyethyl)-5-methyl-2,3-dihydrobenzofuran-4-carboxylate **2i-1-1**, yellow oil, $[\alpha]_D^{20}$ - 34.4 (DCM, c 0.215) for 97% ee. Methyl (S)-2-(2-hydroxyethyl)-5-methyl-2,3-dihydrobenzofuran-4-carboxylate **2i-1-2**, yellow oil, $[\alpha]_D^{20}$ + 34.2 (DCM, c 0.155) for 98% ee.

Kinetic isotope effect experiments

KIE determined from two parallel reactions

General procedure: Substrate (0.3 mmol), $Pd(OAc)_2$ (6.7 mg, 10 mol%), BQ (6.6 mg, 20 mol%), Ag_2CO_3 (165.5 mg, 2.0 equiv), acridine (10.8 mg, 20 mol%), KH_2PO_4 (40.8 mg, 1.0 equiv), NaOAc (37.0 mg, 1.5 equiv) and ^{t}AmylOH (2.0 mL) were added to the 20 mL oven-dried glass tube. The tube was sealed and the reaction mixture was stirred at 140 ℃ under an air atmosphere. After the reaction stopped, the mixture was cooled to rt and then acidified with 2.0 N HCl (5.0 mL). The organic layer was separated and the aqueous layer was extracted with EtOAc (5.0 mL x 2), and dried over anhydrous Na_2SO_4. The solvent was removed under reduced pressure, and the yield was analyzed by NMR using the dibromomethane (17.4 mg, 0.1 mmol) as the internal standard.

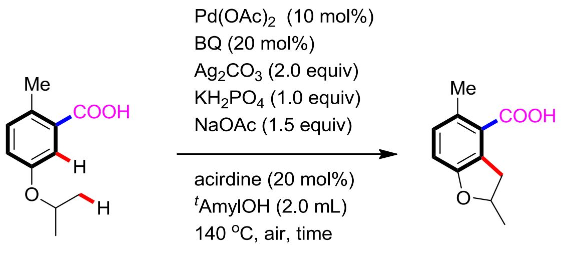

1a, 0.3 mmol

2aa

Entry	Time	Yield
1	0.5 h	6.6%
2	1.0 h	11.8%
3	1.5 h	20.4%
4	2.0 h	29.9%
5	2.5 h	36.7%

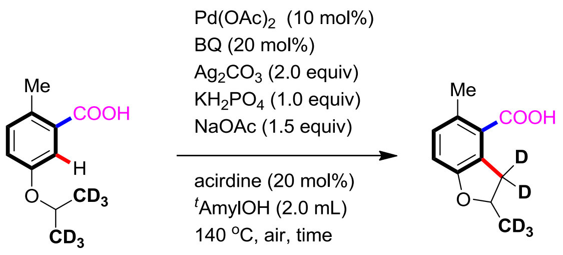

1a-D$_6$, 0.3 mmol

2aa-D$_5$

Entry	Time	Yield
1	1.0 h	3.9%
2	2.0 h	6.0%
3	3.0 h	8.3%
4	4.0 h	10.3%
5	5.0 h	11.9%

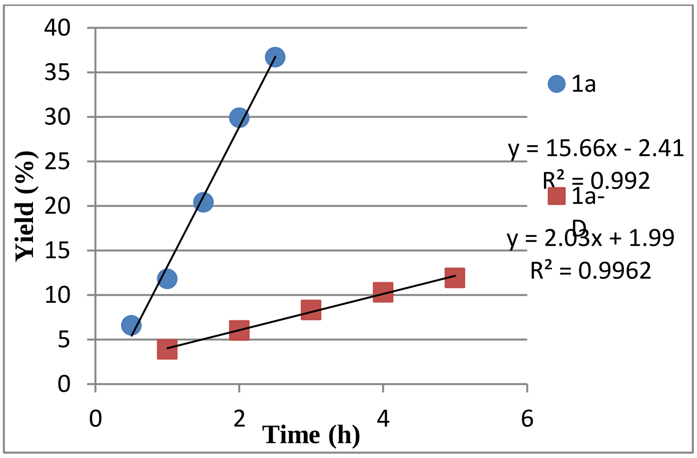

KIE = k_H/k_D = 15.66/2.03 = 7.714

KIE determined from an intermolecular competition

General procedure: To a 20 mL oven-dried glass tube was added **1a** (29 mg, 0.15 mmol),**1a-D$_6$** (30 mg, 0.15 mmol), Pd(OAc)$_2$ (6.7 mg, 10 mol%), BQ (6.6 mg, 20 mol%), Ag_2CO_3 (165.5 mg, 2.0 equiv), acridine (10.8 mg, 20 mol%), KH_2PO_4 (40.8 mg, 1.0 equiv), NaOAc (37.0 mg, 1.5 equiv) and ^{t}AmylOH (2.0 mL) were added to the 20 mL oven-dried glass tube. The tube was sealed and the reaction mixture was stirred at 140 ℃ for 3 h under an air atmosphere. After the reaction stopped, the mixture was cooled to rt and then acidified with 2.0 N HCl (5.0 mL). The organic layer was separated and the aqueous layer was extracted with EtOAc (5.0 mL x 2), and dried over anhydrous Na_2SO_4. The solvent was removed under reduced pressure, and the yield was analyzed by NMR without the internal standard.

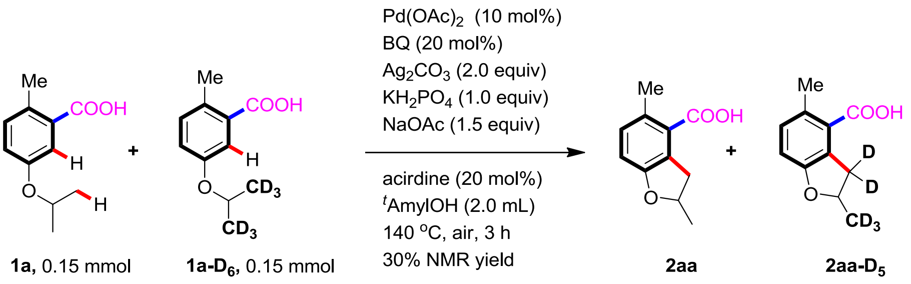

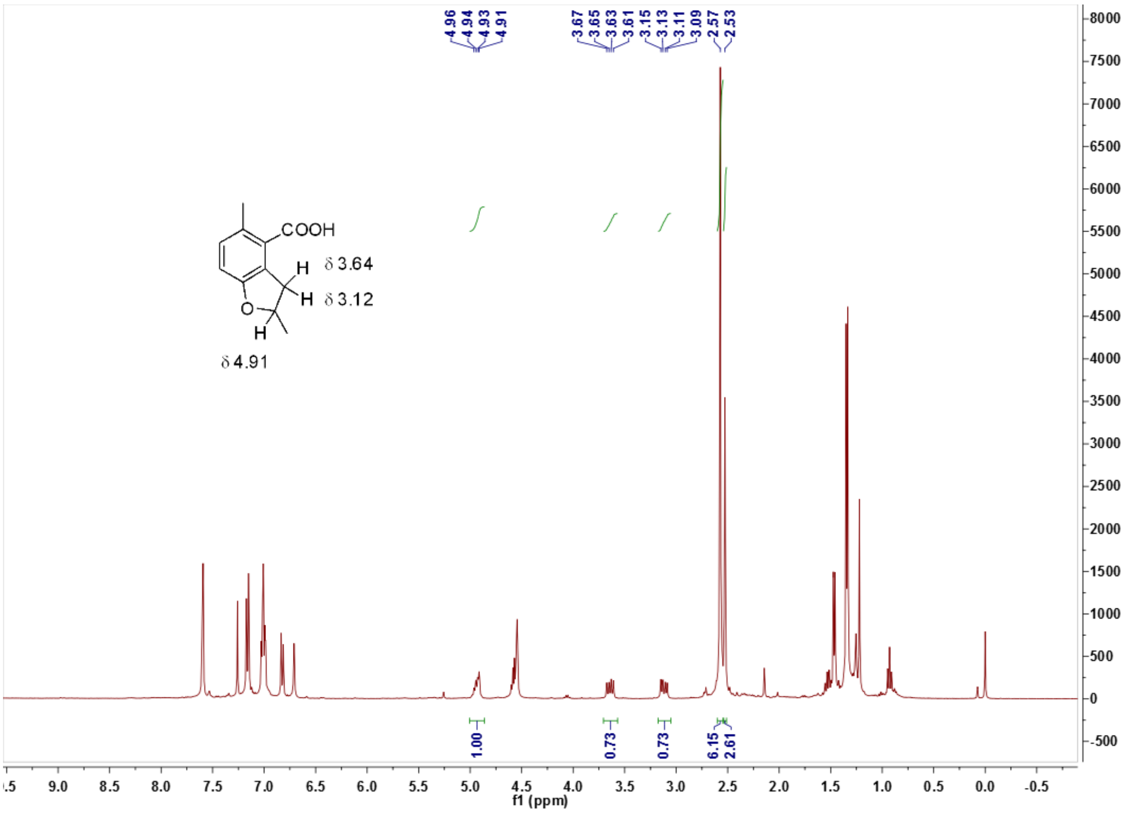

KIE = 0.73/1-0.73 = 2.7

Yield = 2.61/2.61+6.15 = 29.8%

KIE determined from an intramolecular competition

General procedure: To a 20 mL oven-dried glass tube was added **1a-D$_3$** (29 mg, 0.15 mmol),**1a-D$_6$** (30 mg, 0.15 mmol), Pd(OAc)$_2$ (6.7 mg, 10 mol%), BQ (6.6 mg, 20 mol%), Ag_2CO_3 (165.5 mg, 2.0 equiv), acridine (10.8 mg, 20 mol%), KH_2PO_4 (40.8 mg, 1.0 equiv), NaOAc (37.0 mg, 1.5 equiv) and ^{t}AmylOH (2.0 mL) were added to the 20 mL oven-dried glass tube. The tube was sealed and the reaction mixture was stirred at 140 ℃ for 3 h under an air atmosphere. After the reaction stopped, the mixture was cooled to rt and then acidified with 2.0 N HCl (5.0 mL). The organic layer was separated and the aqueous layer was extracted with EtOAc (5.0 mL x 2), and dried over anhydrous Na_2SO_4. The solvent was removed under reduced pressure, and the yield was analyzed by NMR without the internal standard.

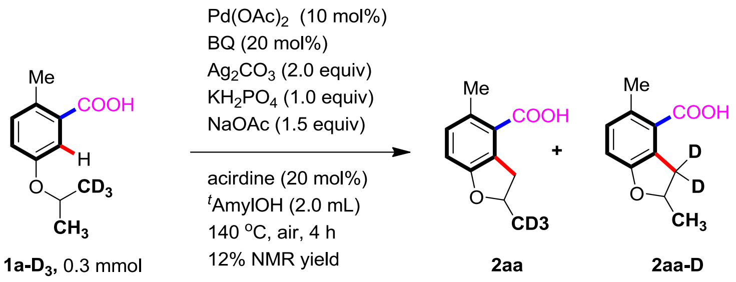

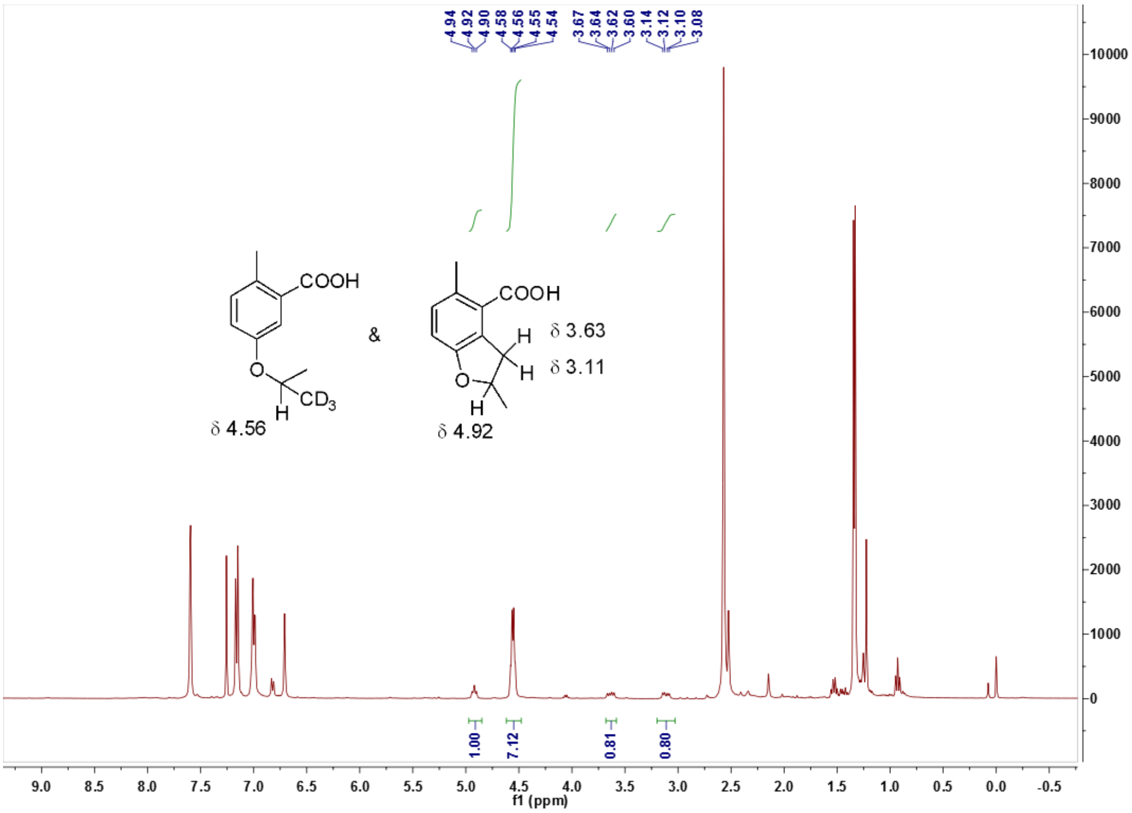

KIE = [(0.81+0.80)/2]/ [1-(0.81+0.80)/2] = 4.13

Yield = 1/1+7.12 = 12.3%

Supplementary References

1. Bartoli, G., Bosco, M., Carlone, A., Dalpozzo, R., Locatelli, M., Melchiorre, P. and Sambri, L. Alcohols and di-tert-butyl dicarbonate: how the nature of the Lewis acid catalyst may address the reaction to the synthesis of tert-butyl ethers. *J. Org. Chem.* **71**, 9580-9588 (2006).

2. Sargent, B. T. and Alexanian, E. J. Palladium-Catalyzed Alkoxycarbonylation of Unactivated Secondary Alkyl Bromides at Low Pressure. *J. Am. Chem. Soc.* **138**, 7520-7523 (2016).

3. Lipshutz, B. H., Chung, D. W., Rich, B. and Corral, R. Simplification of the Mitsunobu Reaction. Di-p-chlorobenzyl Azodicarboxylate: A New Azodicarboxylate. *Org. Lett.* **8**, 5069-5072 (2006).

4. Chen, M., Wang, J., Chai, Z., You, C. and Lei, A. C-X (X=Br, I) Bond-Tolerant

Aerobic Oxidative Cross-Coupling: A Strategy to Selectively Construct β-Aryl Ketones and Aldehydes. *Adv. Synth. Catal*. **354**, 341-346 (2012).
5. Dai, J.-J., Liu, J.-H., Luo, D.-F. and Liu, L. Pd-catalysed decarboxylative Suzuki reactions and orthogonal Cu-based O-arylation of aromatic carboxylic acids. *Chem. Commun.* **47**, 677–679 (2011).
6. Sun, L.-Q., Takaki, K., Chen, J., Iben, L., Knipe, J. O., Pajor, L., Mahle, C. D., Ryan, E. and Xu, C. N-{2-[2-(4-Phenylbutyl)benzofuran-4-yl]cyclopropylmethyl}acetamide: an orally bioavailable melatonin receptor agonist. *Bioorg. Med. Chem. Lett.* **14**, 5157–5160 (2004).
7. Ma, C., Rangasamy, E., Liang, C., Sakamoto, J., More, K. L. and Chi, M. Cover Picture: Excellent Stability of a Lithium-Ion-Conducting Solid Electrolyte upon Reversible Li+/H+ Exchange in Aqueous Solutions (Angew. Chem. Int. Ed. 1/2015). *Angew. Chem. Int. Ed.* **54**, 1-6 (2015).
8. Lafrance, M., Gorelsky, S. I. and Fagnou, K. High-Yielding Palladium-Catalyzed Intramolecular Alkane Arylation: Reaction Development and Mechanistic Studies. *J. Am. Chem. Soc.* **129**, 14570–14571 (2007).
9. Walker, S. D., Barder, T. E., Martinelli, J. R. and Buchwald, S. L. A Rationally Designed Universal Catalyst for Suzuki–Miyaura Coupling Processes. *Angew. Chem. Int. Ed.* **43**, 1871-1876 (2004).
10. Tichenor, M. S., Trzupek, J. D., Kastrinsky, D. B., Shiga, F., Hwang, I. and Boger, D. L. Asymmetric Total Synthesis of (+)- and ent-(−)-Yatakemycin and Duocarmycin SA: Evaluation of Yatakemycin Key Partial Structures and Its Unnatural Enantiomer. *J. Am. Chem. Soc.* **128**, 15683-15696 (2006).
11. Martin, Novák, P., Correa, A., Gallardo-Donaire, J. and Martin, R. Synergistic Palladium-Catalyzed C(sp^3)-H Activation/C(sp^3)-O Bond Formation: A Direct, Step-Economical Route to Benzolactones. *Angew. Chem. Int. Ed.* **50**, 12236-12239 (2011).
