# Supplementary material for: Oxidative coupling of sp2 and sp3 carbon–hydrogen bonds to construct dihydrobenzofurans
Source: Nat Commun. 2017 Aug 10;8:238. doi: 10.1038/s41467-017-00078-6 (PMC5552783; doi:10.1038/s41467-017-00078-6)
Supplement: Supplementary file 2 — Peer Review File [file 41467_2017_78_MOESM2_ESM.pdf]

Editorial Note: this manuscript has been previously reviewed at another journal that is not operating a transparent peer review scheme. This document only contains reviewer comments and rebuttal letters for versions considered at Nature Communications.

Reviewer #1 (Remarks to the Author):

The present manuscript describes the development of an oxidative coupling between phenyl and inert alkyl C-H bonds by under the Pd catalysis. Synthetically valuable products of dihydrobenzofurans were obtained in high efficiency from substituted phenyl alkyl ethers. It is noteworthy that weakly coordinating groups, carboxylic acids, were successfully utilized for this purpose. While the fundamental mechanistic background is already known, the synthetic value of this work is truly high as well demonstrated by the authors. I would like to recommend the publication of this study as it stands.

Reviewer #2 (Remarks to the Author):

The authors have addressed all concerns of the reviewers and should be acceptable in Nat. Commun.
